# Treatment and care received by children hospitalized with COVID-19 in a large hospital network in the United States, February 2020 to September 2021

**DOI:** 10.1371/journal.pone.0288284

**Published:** 2023-07-11

**Authors:** Candace C. Fuller, Austin Cosgrove, Mayura Shinde, Edward Rosen, Katie Haffenreffer, Christian Hague, Laura E. McLean, Jonathan Perlin, Russell E. Poland, Kenneth E. Sands, Natasha Pratt, Patricia Bright, Richard Platt, Noelle M. Cocoros, Sarah K. Dutcher

**Affiliations:** 1 Department of Population Medicine, Harvard Medical School and Harvard Pilgrim Health Care Institute, Boston, Massachusetts, United States of America; 2 HCA Healthcare, Nashville, Tennessee, United States of America; 3 US Food and Drug Administration, Silver Spring, Maryland, United States of America; Bay Area Hospital, North Bend Medical Center, UNITED STATES

## Abstract

We described care received by hospitalized children with COVID-19 or multi-system inflammatory syndrome (MIS-C) prior to the 2021 COVID-19 Omicron variant surge in the US. We identified hospitalized children <18 years of age with a COVID-19 or MIS-C diagnosis (COVID-19 not required), separately, from February 2020-September 2021 (n = 126 hospitals). We described high-risk conditions, inpatient treatments, and complications among these groups. Among 383,083 pediatric hospitalizations, 2,186 had COVID-19 and 395 had MIS-C diagnosis. Less than 1% had both COVID-19 and MIS-C diagnosis (n = 154). Over half were >6 years old (54% COVID-19, 70% MIS-C). High-risk conditions included asthma (14% COVID-19, 11% MIS-C), and obesity (9% COVID-19, 10% MIS-C). Pulmonary complications in children with COVID-19 included viral pneumonia (24%) and acute respiratory failure (11%). In reference to children with COVID-19, those with MIS-C had more hematological disorders (62% vs 34%), sepsis (16% vs 6%), pericarditis (13% vs 2%), myocarditis (8% vs 1%). Few were ventilated or died, but some required oxygen support (38% COVID-19, 45% MIS-C) or intensive care (42% COVID-19, 69% MIS-C). Treatments included: methylprednisolone (34% COVID-19, 75% MIS-C), dexamethasone (25% COVID-19, 15% MIS-C), remdesivir (13% COVID-19, 5% MIS-C). Antibiotics (50% COVID-19, 68% MIS-C) and low-molecular weight heparin (17% COVID-19, 34% MIS-C) were frequently administered. Markers of illness severity among hospitalized children with COVID-19 prior to the 2021 Omicron surge are consistent with previous studies. We report important trends on treatments in hospitalized children with COVID-19 to improve the understanding of real-world treatment patterns in this population.

## Introduction

Globally the COVID-19 pandemic has led to high morbidity and mortality in adults, but most reported SARS-CoV-2 infections in children have been described as mild or asymptomatic [1, 2]. Studies have reported low rates of COVID-19 associated hospitalization in children as compared to adults [3], with increases coinciding with wide circulation of the SARS-CoV-2 Delta variant and Omicron variants [4]. Although COVID-19 vaccines prevent severe consequences of SARS-CoV-2 infections and were first authorized for patients ages 16 years and older in December 2020 [5], they were not available until May 2021 for adolescents ages 12–15 years [6], October 2021 for children ages 5–11 years [7] and June 2022 for use in children ages 6 months through 5 years [8].

Rare but serious illnesses such as multi-system inflammatory syndrome (MIS-C) can occur in children following COVID-19 infection [9], and hospitalized children may require intensive care unit (ICU) support [10–12]. Although illness severity and medication use in hospitalized children has previously been characterized [13–17], less is known about potential COVID-19 treatments such as monoclonal antibodies that were under emergency use authorization through 2021. In addition, the National Institute of Health’s COVID-19 treatment guidelines for children last updated in 2022 bases some recommendations on data for adult patients, in part due to inadequate clinical trial or observational study data on treatment of children with acute COVID-19 [18]. An improved understanding of real-world treatment patterns among children hospitalized with COVID-19 can inform regulators and future clinical trials designed to evaluate pediatric treatments for COVID-19.

The US Food and Drug Administration’s (FDA) Sentinel System [19, 20] is an active post-marketing surveillance system that uses curated, quality checked administrative claims and electronic health record (EHR) data to study medical products, and has implemented an array of public health surveillance activities to respond to the COVID-19 pandemic [21]. This includes partnering with a large US hospital system to allow for near real-time monitoring of hospitalized patients with COVID-19 [21, 22]. As treatment patterns and illness severity may be influenced by both COVID-19 vaccination status and SARS-CoV-2 variant type, our objective was to describe characteristics, complications, and treatments administered to hospitalized children with evidence of COVID-19 or MIS-C diagnosis prior to the Omicron variant surge in December 2021 and widespread vaccination of children less than 12 years.

## Materials and methods

### Data sources and study population

This was a retrospective descriptive study among children <18 years hospitalized from February 20, 2020 through September 30, 2021. We used inpatient EHR data from HCA Healthcare [23] (n = 126 hospitals which provided pediatric care). Hospitals were in 18 states, with Texas, Florida, Virginia, and Colorado heavily represented. The study start date corresponds with the release of initial COVID-19 diagnostic coding guidelines [24].

We identified hospitalizations with a COVID-19 specific diagnosis code (International Classification of Diseases, Tenth Revision, Clinical Modification [ICD-10-CM], U07.1-COVID-19) which became available April 1, 2020, as well as codes for coronavirus infection that were in use prior to April 1, 2020 (ICD-10-CM, B97.29- Other coronavirus as the cause of diseases classified elsewhere; B34.2-Coronavirus infection, unspecified). As MIS-C can present sometime after an initial SARS-CoV-2 infection, we examined children with MIS-C diagnoses separately without requiring a COVID-19 diagnosis (M35.81-Multisystem Inflammatory Disorder, M35.8-Other specific systemic involvement of connective tissue). We included data on patients discharged and with complete billing only.

For reference, we also examined characteristics of all hospitalizations in children captured in the database. See S1 Appendix for a study design diagram.

### Patient characteristics and high-risk conditions

We examined patient demographics on admission and analyzed the frequency of admission by month across the study period. We examined conditions that may increase risk of serious complications from COVID-19, including cancer, chronic lung diseases, chronic cardiovascular disease (e.g., congenital heart disease), liver or renal disorders, immunocompromised state, diabetes, obesity, developmental disorders and smoking [25]. All conditions were identified via ICD-10-CM diagnosis codes documented throughout the hospitalization. We described the frequency of COVID-19 diagnosis among newborns delivered in these hospitals during the study period (i.e., live birth delivery).

We also assessed how often patients had SARS-CoV-2 polymerase chain reaction (PCR) tests performed within the hospital system. See S2 Appendix for applicable code lists.

### Treatments

We identified administrations of therapies for management of COVID-19 during hospitalization, including remdesivir, dexamethasone, and methylprednisolone, and examined the frequency of these administrations among children with COVID-19 or MIS-C diagnosis over time. We also examined use of antibiotics, antithrombotic therapies, and immune globulin. We used brand names, generic names, National Drug Codes (NDC), and ICD-10-CM procedure codes to identify medications (see S3 Appendix for medication search strategy and codes).

### Complications and illness severity markers

We described complications coded during the hospital stay, including pulmonary complications, inflammatory conditions, thromboembolic complications, hematological disorders, myocarditis, pericarditis, and sepsis. As hematological disorders included many conditions, we also examined the top 25 most common hematological diagnoses in this group. We also examined death in the hospital based on discharge disposition (i.e., discharged expired).

As markers of illness severity, we examined receipt of respiratory support during the stay, ICU stays, and length of stay. Respiratory support included supplemental oxygen (e.g., high flow nasal cannula, routine nasal cannula, nonrebreather mask, oxygen conserving device, simple mask), bilevel positive airway pressure (BiPAP), mechanical ventilation (MV), and extracorporeal membrane oxygenation (ECMO). Clinicians reviewed and classified standardized nursing documentation to identify supplemental oxygen and invasive mechanical ventilation. We identified ICU stays with revenue codes, and length of stay was calculated based on admission and discharge dates. See S2 Appendix for applicable code lists.

Data were fully anonymized (no individually identifiable data included) before accessed by a programmer on the study team. This Sentinel activity was a public health surveillance activity conducted under the authority of FDA and, accordingly, was not subject to Institutional Review Board oversight [26–28].

## Results

We observed 2,186 hospitalizations with a COVID-19 diagnosis and 395 with MIS-C diagnosis among 383,083 total pediatric hospitalizations. Thirty-nine percent of children with MIS-C diagnosis also had a COVID-19 diagnosis during the same hospitalization.

**Fig 1:**
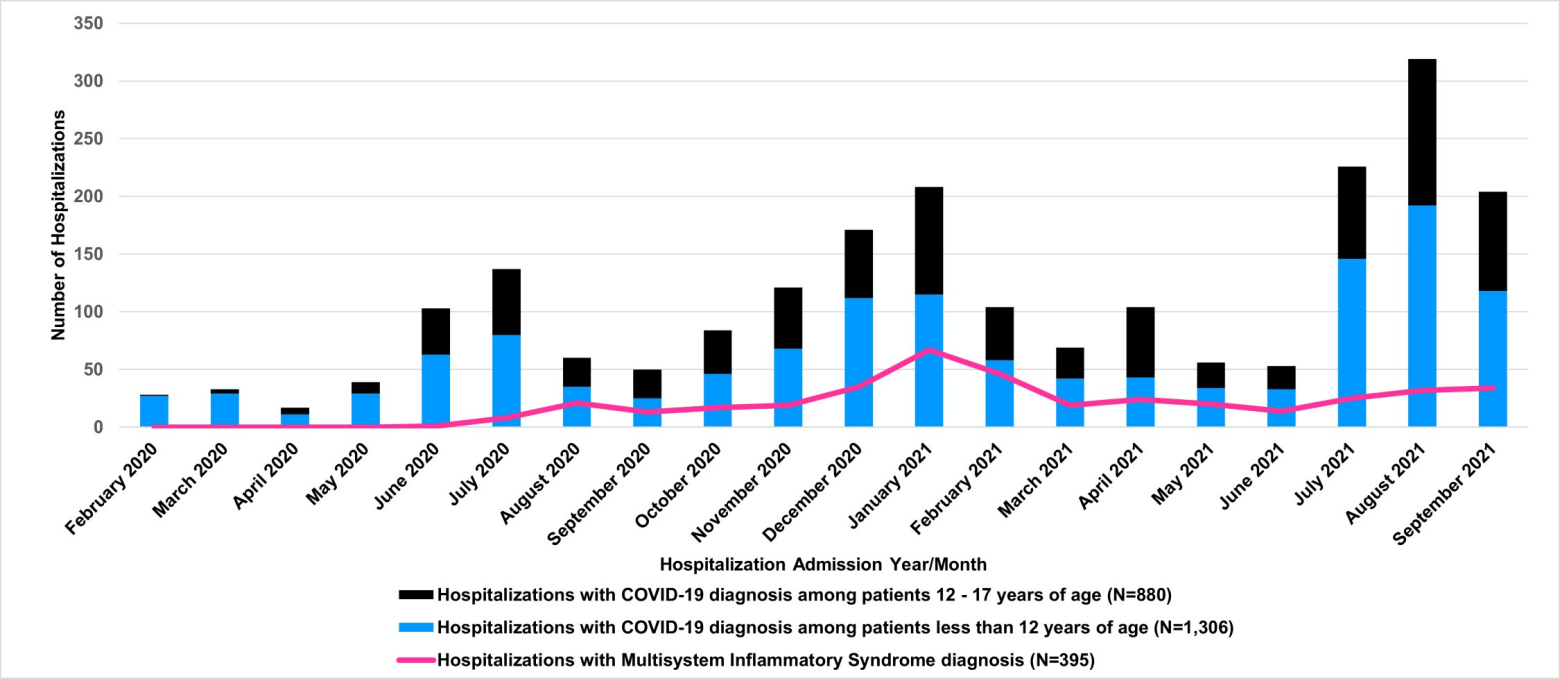
Pediatric hospitalizations with COVID-19 or multi-system inflammatory (MIS-C) diagnosis, February 20, 2020-September 30, 2021.

Fig 1 shows COVID-19 hospitalizations by month. We observed increases in pediatric hospitalization corresponding to known hospitalization surges during the pandemic including higher numbers of hospitalized children during the summer of 2021 [16]. In addition, 60% of hospitalized children with COVID-19 diagnosis in this analysis were <12 years of age. Fifty-eight percent of children with COVID-19 diagnosis had evidence of SARS-CoV-2 testing at the same hospital during their stay, and 85% of those had a positive PCR test. In contrast, just 10% of children with MIS-C diagnosis had a positive PCR test during their stay. Less than 1% of children with a COVID-19 diagnosis also had an MIS-C diagnosis (n = 154).

### Characteristics of hospitalized children with COVID-19 or MIS-C diagnosis

**Table 1** shows the characteristics of hospitalized children diagnosed with COVID-19 or MIS-C. Approximately 49% of children with COVID-19 diagnosis were female, and their median age was 7 years (interquartile range [IQR] 0–15 years). On admission, 60% of children with COVID-19 diagnosis were ages <12 years and 40% were 12–17 years. Among children with a COVID-19 diagnosis, we identified 129 (6%) newborns, which represented <1% of all deliveries in these hospitals during the study. Approximately 53% of children with COVID-19 were white, 17% black, and 24% other race, which was similar to all hospitalizations in the database (56% white, 16% black, 18% other race). Thirty-nine percent of children with COVID-19 diagnosis were of Hispanic ethnicity, while just 18% of all hospitalizations in the database were in children of Hispanic ethnicity. We observed regional variation in accordance with the distribution of the hospital network, with 38% of pediatric COVID-19 hospitalizations in Texas and 19% in Florida. The most recorded high-risk conditions were asthma (14%), cardiovascular diseases (12%), obesity (9%), immune compromising conditions (7%), liver and renal disorders (6%), and diabetes (5%).

**Table 1:** Baseline characteristics of hospitalizations with COVID-19 or MIS-C diagnosis stratified by ICU stays or age, February 20, 2020 and September 30, 2021*.

	All hospitalizations (N = 383,083)	Hospitalizations with COVID-19 diagnosis (N = 2,186)	Hospitalizations with multisystem inflammatory syndrome diagnosis (N = 395)	Hospitalizations with COVID-19 diagnosis AND ICU admission (N = 917)	Hospitalizations with COVID-19 diagnosis among patients less than 12 years of age (N = 1,306)	Hospitalizations with COVID-19 diagnosis among patients 12–17 years of age (N = 880)
**Total Patients**	368,840	2,146	390	910	1,286	860
	N (%)	N (%)	N (%)	N (%)	N (%)	N (%)
**Age at admission**						
<1 year	323,388 (84%)	621 (28%)	26 (7%)	186 (20%)	621 (48%)	-
1-<2 years	5,257 (1%)	155 (7%)	26 (7%)	60 (7%)	155 (12%)	-
2-<6 years	9,374 (2%)	221 (10%)	66 (17%)	100 (11%)	221 (17%)	-
6-< 12 years	10,843 (3%)	309 (14%)	139 (35%)	158 (17%)	309 (24%)	-
12 -<18 years	34,029 (9%)	880 (40%)	138 (35%)	413 (45%)	-	880 (100%)
**Sex**						
Female	188,883 (49%)	1,078 (49%)	175 (44%)	438 (48%)	583 (45%)	495 (56%)
**Race**						
White	215,138 (56%)	1,152 (53%)	198 (50%)	492 (54%)	686 (53%)	466 (53%)
Black	61,198 (16%)	377 (17%)	85 (22%)	163 (18%)	228 (18%)	149 (17%)
American Indian/ Alaska Native/ Hawaiian/ Pacific Islander	1,359 (0%)	11 (1%)	2 (1%)	6 (1%)	3 (0%)	8 (1%)
Asian/Asian Indian	19,883 (5%)	32 (2%)	9 (2%)	13 (1%)	20 (2%)	12 (1%)
Other	69,768 (18%)	527 (24%)	92 (23%)	203 (22%)	323 (25%)	204 (23%)
Unknown	15,565 (4%)	87 (4%)	9 (2%)	40 (4%)	46 (4%)	41 (5%)
**Ethnicity**						
Hispanic or Latino	67,608 (18%)	857 (39%)	136 (34%)	392 (43%)	491 (38%)	366 (42%)
Unknown	157,287 (41%)	241 (11%)	24 (6%)	87 (10%)	178 (14%)	63 (7%)
**State**						
Colorado	24,892 (7%)	116 (5%)	25 (6%)	36 (4%)	64 (5%)	52 (6%)
Florida	63,630 (17%)	409 (19%)	63 (16%)	174 (19%)	220 (17%)	189 (22%)
Other†	99,507 (26%)	646 (30%)	160 (41%)	256 (28%)	410 (31%)	236 (27%)
Tennessee	18,794 (5%)	86 (4%)	16 (4%)	21 (2%)	45 (3%)	41 (5%)
Texas	151,294 (40%)	830 (38%)	123 (31%)	407 (44%)	518 (40%)	312 (36%)
Virginia	24,794 (7%)	99 (5%)	8 (2%)	23 (3%)	49 (4%)	50 (6%)
**Live birth delivery or pregnancy**						
Live birth delivery	306,212 (80%)	129 (6%)	1 (0%)	6 (1%)	129 (10%)	0 (0%)
Pregnancy§	127 (0%)	85 (4%)	-	9 (1%)	-	85 (10%)
**High-risk conditions documented during the stay**						
Asthma	10,360 (3%)	312 (14%)	45 (11%)	186 (20%)	149 (11%)	163 (19%)
Chronic obstructive pulmonary disease	173 (0%)	9 (0%)	2 (1%)	5 (1%)	4 (0%)	5 (1%)
Cystic fibrosis	157 (0%)	1 (0%)	0 (0%)	0 (0%)	0 (0%)	1 (0%)
Tuberculosis	32 (0%)	3 (0%)	0 (0%)	2 (0%)	2 (0%)	1 (0%)
Other chronic respiratory disorders (e.g., chronic respiratory disorders originating in the perinatal period)	1,591 (0%)	21 (1%)	9 (2%)	14 (2%)	12 (1%)	9 (1%)
Diabetes	2,958 (1%)	103 (5%)	7 (2%)	77 (8%)	33 (3%)	70 (8%)
Heart failure	328 (0%)	17 (1%)	17 (4%)	16 (2%)	10 (1%)	7 (1%)
Cardiovascular disease¶	7,355 (2%)	260 (12%)	173 (44%)	184 (20%)	140 (11%)	120 (14%)
Congenital heart disease	16,026 (4%)	96 (4%)	16 (4%)	48 (5%)	73 (6%)	23 (3%)
Sickle cell disease or thalassemia	704 (0%)	29 (1%)	4 (1%)	12 (1%)	18 (1%)	11 (1%)
Mental health conditions (e.g., depression, bi-polar disorder)	13,651 (4%)	159 (7%)	9 (2%)	59 (6%)	9 (1%)	150 (17%)
Down syndrome	1,001 (0%)	28 (1%)	0 (0%)	20 (2%)	12 (1%)	16 (2%)
Liver and renal disorders	4,347 (1%)	138 (6%)	79 (20%)	75 (8%)	75 (6%)	63 (7%)
Obesity	2,838 (1%)	203 (9%)	38 (10%)	134 (15%)	26 (2%)	177 (20%)
Immunocompromised state (e.g., diagnoses of primary immunodeficiency, HIV, or graft versus host related complications)	2,232 (1%)	147 (7%)	33 (8%)	83 (9%)	79 (6%)	68 (8%)
Malignant cancer	1,738 (1%)	30 (1%)	4 (1%)	14 (2%)	20 (2%)	10 (1%)

*Includes discharges for final billed patients only. The analysis does not include inpatient stays for patients still admitted or not completely coded by the data pull date.

† Other includes states with less representation in the network: CA, GA, ID, IN, KS, KY, LA, MO, MS, NH, NV, SC, UT.

§ Defined with ICD-9-CM code O98. 51 (Other viral disease complicating pregnancy, childbirth, or the puerperium)

¶Cardiovascular conditions included hospitalizations with diagnosis codes for myocardial infarction, angina pectoris, atherosclerosis, heart failure, aneurysm, and ischemic heart disease

In children with MIS-C, 44% were female, and the median age was 9 years (IQR 4–13 years). Characteristics were generally similar among children diagnosed with MIS-C compared to children with COVID-19, particularly with respect to underlying conditions, but diagnoses of hematologic disorders (62%), cardiovascular diseases (44%), and liver and renal disorders (20%) were more common. Among children with MIS-C and hematologic disorders, the most frequent diagnoses included thrombocytopenia (45%), decreased white blood cell counts (35%), and anemia (33%).

### Complications and markers of severity among children with COVID-19 or MIS-C diagnosis

**Table 2** summarizes complications among hospitalized children with a COVID-19 or MIS-C diagnosis. The most common pulmonary complications in children with a COVID-19 diagnosis were viral pneumonia (24%) and acute respiratory failure (11%). In reference to children with COVID-19 diagnosis, a higher proportion of some complications was observed in those with MIS-C diagnosis including hematologic conditions (62% vs 34%), sepsis (16% vs. 6%), pericarditis (13% vs. 2%), and myocarditis (8% vs. 1%). Viral pneumonia (9% vs. 24%) was less frequently documented in patients with MIS-C diagnosis than with a COVID-19 diagnosis. Less than 1% of children with a COVID-19 or MIS-C diagnosis had acute bronchitis or lower respiratory infection, and less than 0.2% thromboembolic complications were observed. Among children with hematologic disorders, the most frequently diagnosed conditions included decreased white blood cell counts [e.g., neutropenia, lymphocytopenia] (40%), anemia (35%), and thrombocytopenia (22%).

**Table 2:** Complications among hospitalizations with COVID-19 or MIS-C diagnosis receiving oxygen-related therapies delivery stratified by ICU stays and age, February 20, 2020-September 30, 2021*.

	Hospitalizations with COVID-19 diagnosis (N = 2,186)	Hospitalizations with multisystem inflammatory syndrome diagnosis (N = 395)	Hospitalizations with COVID-19 diagnosis AND ICU admission (N = 917)	Hospitalizations with COVID-19 diagnosis among patients less than 12 years of age (N = 1,306)	Hospitalizations with COVID-19 diagnosis among patients 12–17 years of age (N = 880)
	**%**	**%**	**%**	**%**	**%**
**Pulmonary complications**					
Viral pneumonia	519 (24%)	36 (9%)	307 (33%)	215 (16%)	304 (35%)
Acute respiratory failure	244 (11%)	43 (11%)	205 (22%)	96 (7%)	148 (17%)
Acute respiratory distress syndrome	25 (1%)	10 (3%)	19 (2%)	6 (0%)	19 (2%)
**Oxygen-related therapy**					
Bilevel positive airway pressure (BiPAP)	75 (3%)	25 (6%)	66 (7%)	29 (2%)	46 (5%)
Extracorporeal membrane oxygenation† (ECMO)	1 (0%)	1 (0%)	1 (0%)	0 (0%)	1 (0%)
High flow	309 (14%)	79 (20%)	266 (29%)	143 (11%)	166 (19%)
Nasal cannula	673 (31%)	155 (39%)	408 (44%)	340 (26%)	333 (38%)
Non-rebreather	77 (4%)	14 (4%)	60 (7%)	25 (2%)	52 (6%)
Oxygen conserving device	5 (0%)	0 (0%)	3 (0%)	0 (0%)	5 (1%)
Simple mask	195 (9%)	43 (11%)	105 (11%)	125 (10%)	70 (8%)
**Other complications**					
Sepsis	141 (6%)	65 (16%)	85 (9%)	97 (7%)	45 (5%)
Myocarditis	25 (1%)	33 (8%)	20 (2%)	8 (1%)	17 (2%)
Pericarditis	40 (2%)	51 (13%)	35 (4%)	23 (2%)	17 (2%)
Myocarditis or pericarditis	45 (2%)	57 (14%)	40 (4%)	23 (2%)	22 (3%)
Hematological disorders	419 (34%)	209 (62%)	242 (37%)	215 (34%)	204 (34%)
**Markers of illness severity**					
ICU admission	917 (42%)	272 (69%)	917 (100%)	504 (39%)	413 (47%)
Invasive mechanical ventilation	109 (5%)	36 (9%)	93 (10%)	59 (5%)	50 (6%)
Respiratory support, including supplemental oxygen or BiPAP (High flow or Nasal cannula or Non-rebreather or Oxygen conserving device or Simple mask or Bilevel positive airway pressure)	841 (39%)	180 (46%)	500 (55%)	454 (35%)	387 (44%)
Death	11 (1%)	3 (1%)	8 (1%)	7 (1%)	4 (0%)
**Length of stay (days)**					
Mean (SD)	5.9 (10.8)	6.9 (5.4)	6.7 (7.6)	5.4 (8.6)	6.8 (13.3)
Median (interquartile range)	3 (2–6)	6 (4–8)	5 (3–8)	3 (2–5)	4 (3–7)

*Includes discharges for final billed patients only. The analysis does not include inpatient stays for patients still admitted or not completely coded by the data pull date.

† Please note only 15 of the 126 HCA Healthcare facilities contributing pediatric inpatient data to this manuscript use ECMO machines routinely

Overall, the median length of stay (LOS) among children with a COVID-19 diagnosis (n = 2,185) was 3 days (interquartile range [IQR] 2–6 days), 42% were treated in the ICU and 0.5% were discharged expired. Regarding the receipt of oxygen-related therapy, 5% were mechanically ventilated and 39% were provided respiratory support (supplementary oxygen or BiPAP). Among children with an MIS-C diagnosis (n = 395) the median LOS was 6 days (IQR 4–8 days), 69% were treated in ICU, and 0.7% were discharged expired. A higher proportion of children with an MIS-C diagnosis than those with COVID-19 diagnosis received oxygen related therapy: 9% were mechanically ventilated and 45% were provided respiratory support.

Children with ICU admissions had markers of more severe illness than those without ICU admission (ICU admission: 10% MV, 53% oxygen use, <1% death; no ICU admission: 1% MV, 26% oxygen use, <1% death). Complications were more frequent among patients with ICU stays.

## Medications

**Fig 2:**
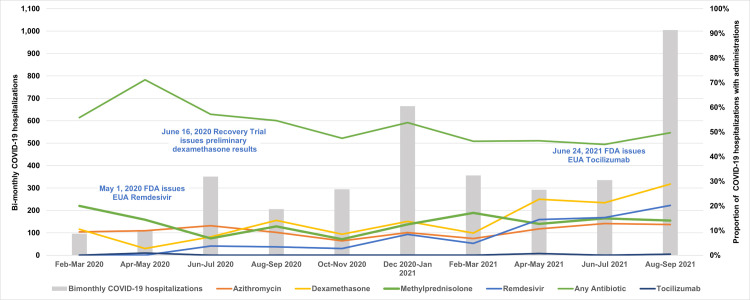
Proportion of pediatric COVID‐19 hospitalizations with administration of select medications, by month, February 20, 2020‐September 30, 2021.

Fig 2 shows the proportion of those diagnosed with COVID-19 or MIS-C over time who received selected medications. The most common agents used to treat hospitalized children with COVID-19 or MIS-C diagnosis during their stay included: methylprednisolone (COVID-19: 34%, MIS-C: 75%), dexamethasone (COVID-19: 25%, MIS-C: 15%), and remdesivir (COVID-19: 13%, MIS-C: 5%). Antibiotic use was also common in both patients with COVID-19 diagnosis (50%) and MIS-C (68%). We observed some utilization of anti-thrombotic medications such as low-molecular weight heparin (COVID-19: 17%, MIS-C: 34%), anti-platelet therapy (COVID-19: 6%, MIS-C: 53%), tissue plasminogen activator (COVID-19: 2%, MIS-C: 6%), and thrombin inhibitors (COVID-19: 2%, MIS-C: 6%). Immune globulin treatment was common in children with MIS-C diagnosis (COVID-19: 7% MIS-C: 67%).

In analyses of hospitalized children with COVID-19 diagnosis stratified by age, we noted higher proportions of children ages 12–17 had evidence of treatments to manage COVID-19 (remdesivir: 6% of those <12 years vs. 24% of those 12–17 years; dexamethasone: 17% in those <12 years vs. 36% in those 12–17 years); the exception was methylprednisolone, which was used more often in younger patients (44% in those <12 years vs. 25% in those 12–17 years) (**Table 3**). Inpatient medication administrations were more frequent in children admitted to the ICU.

**Table 3:** Inpatient medication administrations among hospitalizations with COVID-19 diagnosis stratified by ICU stays and age category, February 20, 2020-September 30, 2021*.

	Hospitalizations with COVID-19 diagnosis (N = 2,186)	Hospitalizations with multisystem Inflammatory syndrome diagnosis (N = 395)	Hospitalizations with COVID-19 diagnosis AND ICU admission (N = 917)	Hospitalizations with COVID-19 diagnosis among patients less than 12 years of age (N = 1,306)	Hospitalizations with COVID-19 diagnosis among patients 12–17 years of age (N = 880)
	**%**	**%**	**%**	**%**	**%**
**Potential COVID-19 treatments†**					
Remdesivir	13.3%	4.8%	22.9%	5.7%	24.4%
Tocilizumab	0.4%	0.8%	0.8%	0.1%	0.8%
Sarilumab	0.0%	0.0%	0.0%	0.0%	0.0%
Eculizumab	0.0%	0.0%	0.1%	0.0%	0.1%
Methylprednisolone	34.4%	75.1%	40.7%	43.5%	24.8%
Lopinavir / Ritonavir	0.0%	0.0%	0.0%	0.0%	0.0%
Norepinephrine	1.1%	8.4%	2.4%	0.8%	1.7%
Dexamethasone	24.6%	14.9%	33.0%	16.9%	36.0%
Remdesivir and Dexamethasone§	11.3%	3.8%	19.4%	3.9%	22.2%
Baricitinib	0.3%	0.3%	0.4%	0.0%	0.8%
Baricitinib and Remdesivir	0.2%	0.0%	0.3%	0.0%	0.6%
Bamlanivimab	0.0%	0.3%	0.1%	0.0%	0.1%
Casirivimab / Imdevimab	0.1%	0.0%	0.1%	0.0%	0.2%
Bamlanivimab and Etesevimab	0.0%	0.0%	0.0%	0.0%	0.0%
**Antibiotics†**					
Any Antibiotic use	49.9%	68.1%	59.7%	47.2%	53.9%
Azithromycin	13.8%	12.9%	18.3%	9.4%	20.2%
Doxycycline	1.0%	8.9%	1.5%	0.7%	1.4%
**Antithrombotic medications†**					
Low molecular weight heparin	17.3%	33.7%	31.2%	5.8%	34.4%
Tissue plasminogen activator	2.0%	5.8%	4.1%	2.2%	1.6%
Vitamin K antagonists	0.0%	0.0%	0.1%	0.1%	0.0%
Factor Xa inhibitors	0.2%	0.3%	0.4%	0.0%	0.5%
Thrombin inhibitors	2.2%	6.1%	4.6%	2.3%	1.9%
Anti-platelet therapy	6.3%	51.4%	10.8%	6.9%	5.5%
**Immune globulin (IVIg)**					
Immune globulin (IVIg)	6.6%	67.1%	11.8%	7.5%	5.2%

*Includes discharges for final billed patients only. The analysis does not include inpatient stays for patients still admitted or not completely coded by the data pull date.

†We defined administration of potential COVID-19 treatments and anti-thrombotic medications by National Drug Codes (NDC) and text string searches for generic and brand name.

Finally, we examined potential COVID-19 treatments under US FDA Emergency Use Authorization (EUA), including monoclonal antibodies and baricitinib and found just two administrations of monoclonal antibodies and seven administrations of baricitinib in hospitalized children with COVID-19 or MIS-C. Baricitinib administrations were first observed in the last week of August 2021.

## Discussion

In this descriptive analysis of children hospitalized in 126 hospitals through September 2021, we found relatively few children with a COVID-19 or MIS-C diagnosis. However, 41% of hospitalized children with COVID-19 and 70% with MIS-C were admitted to an ICU, 5% of those with COVID-19 and 9% with MIS-C were mechanically ventilated, and 38% of children with COVID-19 and 45% with MIS-C required supplemental oxygen or BiPAP. We identified few deaths (<1%) and most children were discharged home (93%). This analysis was conducted prior to eligibility of children <12 years for COVID-19 vaccination, and 60% of hospitalized children with COVID-19 diagnosis in this analysis were <12 years with the highest proportions of hospitalized children observed in the summer of 2021.

Our estimates of markers of illness severity were consistent with other studies [12, 15, 17, 29]. Children with COVID-19 diagnosis admitted to the ICU generally had more high-risk conditions (e.g., asthma,), complications, and respiratory support recorded during their hospitalization than noted in the overall COVID-19 cohort. However, it is possible that some children were admitted to the ICU for reasons other than illness severity as ICU admission decisions are governed by physician discretion and hospital specific practices, particularly related to infection control considerations–i.e., a child may be admitted to the ICU for infection control and not due to severe illness. As we used coded data to identify ICU admissions, we were unable to distinguish specific reasons for ICU admission, but this could be done with detailed medical record review in future studies.

As expected, children with MIS-C diagnosis had evidence of severe illness and longer stays (median LOS 6 days); 69% were admitted to an ICU. As noted in other studies [12, 29–31], children with MIS-C generally had more high-risk conditions than the overall COVID-19 cohort (e.g., liver or renal disorders, cardiovascular disease). However, as conditions were defined with diagnoses during hospitalization, it is possible these included both acute complications and underlying conditions. While many children with COVID-19 or MIS-C had few pre-existing high-risk conditions, some children had asthma or obesity, both factors previously identified as associated with higher risk for prolonged recovery [12].

Viral pneumonia was uncommon among children with MIS-C, but we noted diagnoses such as sepsis (16%), myocarditis or pericarditis (14%). Notably, three quarters of children with MIS-C diagnosis were treated with methylprednisolone, 67% received immune globulin, half received anti-platelet therapy, and a third received low molecular weight heparin. Although understanding of MIS-C and treatments has evolved, observed treatments are consistent with previous reports as well as known complications of MIS-C including cardiac complications, sepsis, and coagulopathy [12, 30–33].

More than half of children with an MIS-C diagnosis did not have a COVID-19 diagnosis or positive test during their stay, this was expected based on the clinical course of disease and coding guidelines [34, 35]. This finding corresponds with recent study which used a data-driven approach to identify features which cluster patients into a group with high likelihood of having MIS-C and identified features such as being older (mean age 7 years) previously healthy and SARS-CoV-2 PCR negative, along with predominately cardiovascular and/or mucocutaneous involvement or high inflammatory biomarkers [30]. A limitation of our study is that tests for inflammatory markers and clinical tests examining cardiovascular or mucocutaneous involvement or function do not exist in dataset we used for this study, but these could be investigated in future studies. Although Feldstein previously reported on medications administered to children hospitalized with COVID-19 or MIS-C through late 2020 [17], we also examined inpatient utilization of antibiotics, and potential COVID-19 treatments such as monoclonal antibodies and baricitinib under EUA at through the end of the study period in late 2021. As expected, treatment patterns differed from those noted in adults [22]. For example, methylprednisolone was used more frequently than dexamethasone, especially in those with MIS-C diagnosis, and antibiotic use was common. In accordance with recent treatment guidelines [34, 35], we observed older children >12 years with COVID-19 were more frequently treated with remdesivir than younger children and those with MIS-C diagnosis were not frequently treated with remdesivir. As of March 2023, remdesivir is the only antiviral drug approved by the FDA for the treatment of COVID-19 in hospitalized children (28 days of age or older and weighing > = 3.0 kg) [36]. Although in July 2021 the FDA reissued an EUA for baricitinib in children > = 2 years hospitalized with COVID-19 requiring oxygen, ventilatory support or ECMO and removed a requirement to use baricitinib with remdesivir [37], we observed very low utilization in our analysis. However, as all baricitinib administrations we observed in children occurred after the EUA update in July 2021, it is possible low utilization could be explained by lack of immediate uptake between the EUA update and the end of our study period in September 2021, or insufficient evidence about benefits and risks in this population [34].

Our analyses characterizing inpatient medication administrations in hospitalized children with COVID-19 are generally consistent with recommendations for supportive care as the mainstay of therapy in children with severe COVID-19 [38–40], and thromboprophylaxis in some instances [41]. However, similar to at least one other US study [42], we observed a high proportion of COVID-19 patients with antibiotic administrations during their stay. In May 2022, NIH treatment guidelines were updated to recommend against empiric broad-spectrum antimicrobial therapy in hospitalized adults with COVID-19 [43]. We did not evaluate indications for antibiotic use, but future studies could investigate this as well as optimal use of antibiotics in hospitalized children with COVID-19.

A strength of this study is the size of the data source. We identified hospitalizations with COVID-19 or MIS-C diagnoses in an inpatient EHR database that includes 126 hospitals and captured over 300,000 pediatric hospitalizations during the COVID-19 pandemic through September 2021. Inpatient EHR data allow for capture and examination of detailed clinical information not routinely available in other electronic sources such as administrative claims. We restricted our analysis to only discharged patients with complete billing information to ensure data completeness [44].

There are several details to consider when interpreting our study results. Due to the nature of inpatient EHR data which captures hospital stay information, we were unable to examine patient characteristics, medication use, or care delivered before or after the hospitalization. We did not have access to the reason for admission, so some patients may have been admitted for other reasons unrelated to COVID-19. We used diagnosis and procedure codes to examine conditions and procedures during the hospitalization and used revenue codes to define ICU stays. We did not have access to SARS-CoV-2 laboratory results from outside the hospital system and used diagnosis codes to identify children with COVID-19 or MIS-C. However, others have shown positive predictive values over 90% for inpatient COVID-19 diagnosis [45, 46].

## Conclusions

Our study provides information on the natural history of COVID-19 and care received by hospitalized children with COVID-19 or MIS-C prior to the 2021 COVID-19 Omicron variant surge in the US. We have described potential COVID-19 therapies administered to children hospitalized with COVID-19 or MIS-C in 126 hospitals located in 18 US states, and report markers of illness severity among hospitalized children with COVID-19 that are consistent with previous studies. Over a third of hospitalized children with COVID-19 were treated with methylprednisolone, 25% with dexamethasone, and 13% were treated with remdesivir. Three quarters of hospitalized children with MIS-C were treated with methylprednisolone, 67% with immune globulin, 51% with antiplatelet therapy, and 33% with low molecular weight heparin. The trends we report on potential COVID-19 therapies administered to hospitalized children with COVID-19 may improve the understanding of real-world treatment patterns in this population and inform regulators and future studies designed to evaluate pediatric treatments for COVID-19.

## Supporting information

S1 Appendix Study design diagram. (PDF)

S2 Appendix List of International Classification of Diseases, Ninth Revision, Clinical Modification (ICD-9-CM), International Classification of Diseases, Tenth Revision, Clinical Modification (ICD-10-CM), International Classification of Diseases, Ninth Revision, Procedure Coding System (ICD-9-PCS) International Classification of Diseases, Tenth Revision, Procedure Coding System (ICD-10-PCS), Current Procedural Terminology, Fourth Edition (CPT-4), and Healthcare Common Procedure Coding System, level II (HCPCS) codes used in the analysis. (PDF)

S3 Appendix Generic and brand names of medical products used in this analysis. (PDF)
