# Supplementary figures and images for: Treatment and care received by children hospitalized with COVID-19 in a large hospital network in the United States, February 2020 to September 2021
Source: PLoS One. 2023 Jul 11;18(7):e0288284. doi: 10.1371/journal.pone.0288284 (PMC10335660; doi:10.1371/journal.pone.0288284)

## S1 Appendix. Study Design Diagram

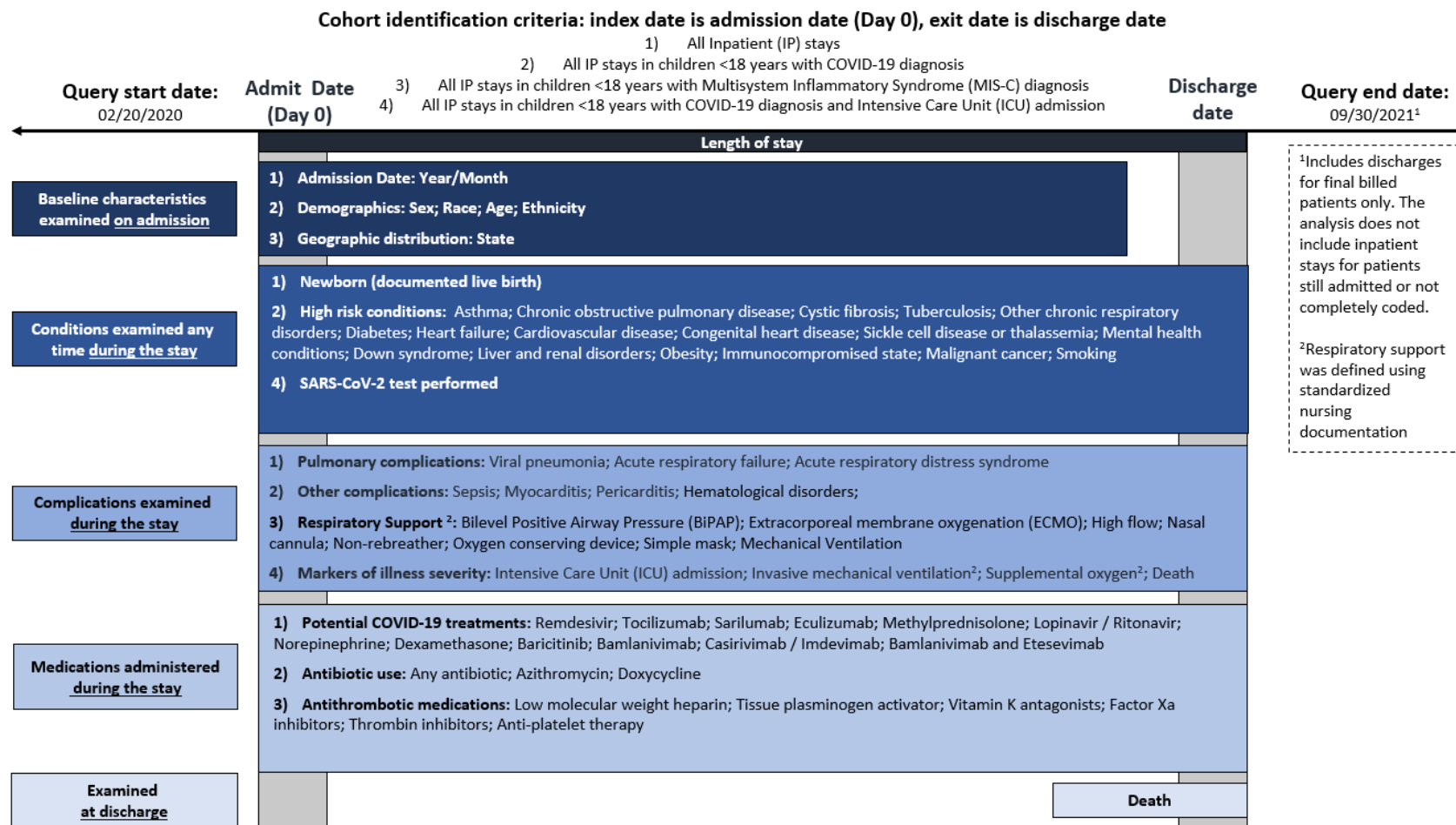

Supplement: S1 Appendix — (PDF) [file pone.0288284.s001.pdf]
