# Supplementary material for: Treatment and care received by children hospitalized with COVID-19 in a large hospital network in the United States, February 2020 to September 2021
Source: PLoS One. 2023 Jul 11;18(7):e0288284. doi: 10.1371/journal.pone.0288284 (PMC10335660; doi:10.1371/journal.pone.0288284)
Supplement: S2 Appendix — (PDF) [file pone.0288284.s002.pdf]

S2 Appendix. List of International Classification of Diseases, Ninth Revision, Clinical Modification (ICD-9-CM), International Classification of Diseases, Tenth Revision, Clinical Modification (ICD-10-CM), International Classification of Diseases, Ninth Revision, Procedure Coding System (ICD-9-PCS) International Classification of Diseases, Tenth Revision, Procedure Coding System (ICD-10-PCS), Current Procedural Terminology, Fourth Edition (CPT-4), and Healthcare Common Procedure Coding System, Level II (HCPCS) Codes Used in the Analysis

Code	Description	Code Category	Code Type
	**Asthma**		
493	Asthma	Diagnosis	ICD-9-CM
493.0	Extrinsic asthma	Diagnosis	ICD-9-CM
493.00	Extrinsic asthma, unspecified	Diagnosis	ICD-9-CM
493.01	Extrinsic asthma with status asthmaticus	Diagnosis	ICD-9-CM
493.02	Extrinsic asthma, with (acute) exacerbation	Diagnosis	ICD-9-CM
493.1	Intrinsic asthma	Diagnosis	ICD-9-CM
493.10	Intrinsic asthma, unspecified	Diagnosis	ICD-9-CM
493.11	Intrinsic asthma with status asthmaticus	Diagnosis	ICD-9-CM
493.12	Intrinsic asthma, with (acute) exacerbation	Diagnosis	ICD-9-CM
493.2	Chronic obstructive asthma	Diagnosis	ICD-9-CM
493.20	Chronic obstructive asthma, unspecified	Diagnosis	ICD-9-CM
493.21	Chronic obstructive asthma with status asthmaticus	Diagnosis	ICD-9-CM
493.22	Chronic obstructive asthma, with (acute) exacerbation	Diagnosis	ICD-9-CM
493.8	Other forms of asthma	Diagnosis	ICD-9-CM
493.81	Exercise induced bronchospasm	Diagnosis	ICD-9-CM
493.82	Cough variant asthma	Diagnosis	ICD-9-CM
493.9	Unspecified asthma	Diagnosis	ICD-9-CM
493.90	Asthma, unspecified, unspecified status	Diagnosis	ICD-9-CM
493.91	Asthma, unspecified with status asthmaticus	Diagnosis	ICD-9-CM
493.92	Asthma, unspecified, with (acute) exacerbation	Diagnosis	ICD-9-CM
J45.20	Mild intermittent asthma, uncomplicated	Diagnosis	ICD-10-CM
J45.21	Mild intermittent asthma with (acute) exacerbation	Diagnosis	ICD-10-CM
J45.22	Mild intermittent asthma with status asthmaticus	Diagnosis	ICD-10-CM
J45.30	Mild persistent asthma, uncomplicated	Diagnosis	ICD-10-CM
J45.31	Mild persistent asthma with (acute) exacerbation	Diagnosis	ICD-10-CM
J45.32	Mild persistent asthma with status asthmaticus	Diagnosis	ICD-10-CM
J45.40	Moderate persistent asthma, uncomplicated	Diagnosis	ICD-10-CM
J45.41	Moderate persistent asthma with (acute) exacerbation	Diagnosis	ICD-10-CM
J45.42	Moderate persistent asthma with status asthmaticus	Diagnosis	ICD-10-CM
J45.50	Severe persistent asthma, uncomplicated	Diagnosis	ICD-10-CM
J45.51	Severe persistent asthma with (acute) exacerbation	Diagnosis	ICD-10-CM
J45.52	Severe persistent asthma with status asthmaticus	Diagnosis	ICD-10-CM
J45.901	Unspecified asthma with (acute) exacerbation	Diagnosis	ICD-10-CM
J45.902	Unspecified asthma with status asthmaticus	Diagnosis	ICD-10-CM
J45.909	Unspecified asthma, uncomplicated	Diagnosis	ICD-10-CM
J45.990	Exercise induced bronchospasm	Diagnosis	ICD-10-CM
J45.991	Cough variant asthma	Diagnosis	ICD-10-CM

Code	Description	Code Category	Code Type
J45.998	Other asthma	Diagnosis	ICD-10-CM
	**Chronic Obstructive Pulmonary Disease**		
490	Bronchitis, not specified as acute or chronic	Diagnosis	ICD-9-CM
491	Chronic bronchitis	Diagnosis	ICD-9-CM
491.0	Simple chronic bronchitis	Diagnosis	ICD-9-CM
491.1	Mucopurulent chronic bronchitis	Diagnosis	ICD-9-CM
491.2	Obstructive chronic bronchitis	Diagnosis	ICD-9-CM
491.20	Obstructive chronic bronchitis, without exacerbation	Diagnosis	ICD-9-CM
491.21	Obstructive chronic bronchitis, with (acute) exacerbation	Diagnosis	ICD-9-CM
491.22	Obstructive chronic bronchitis with acute bronchitis	Diagnosis	ICD-9-CM
491.8	Other chronic bronchitis	Diagnosis	ICD-9-CM
491.9	Unspecified chronic bronchitis	Diagnosis	ICD-9-CM
492	Emphysema	Diagnosis	ICD-9-CM
492.0	Emphysematous bleb	Diagnosis	ICD-9-CM
492.8	Other emphysema	Diagnosis	ICD-9-CM
494	Bronchiectasis	Diagnosis	ICD-9-CM
494.0	Bronchiectasis without acute exacerbation	Diagnosis	ICD-9-CM
494.1	Bronchiectasis with acute exacerbation	Diagnosis	ICD-9-CM
495	Extrinsic allergic alveolitis	Diagnosis	ICD-9-CM
495.0	Farmers' lung	Diagnosis	ICD-9-CM
495.1	Bagassosis	Diagnosis	ICD-9-CM
495.2	Bird-fanciers' lung	Diagnosis	ICD-9-CM
495.3	Suberosis	Diagnosis	ICD-9-CM
495.4	Malt workers' lung	Diagnosis	ICD-9-CM
495.5	Mushroom workers' lung	Diagnosis	ICD-9-CM
495.6	Maple bark-strippers' lung	Diagnosis	ICD-9-CM
495.7	Ventilation pneumonitis	Diagnosis	ICD-9-CM
495.8	Other specified allergic alveolitis and pneumonitis	Diagnosis	ICD-9-CM
495.9	Unspecified allergic alveolitis and pneumonitis	Diagnosis	ICD-9-CM
496	Chronic airway obstruction, not elsewhere classified	Diagnosis	ICD-9-CM
J40	Bronchitis, not specified as acute or chronic	Diagnosis	ICD-10-CM
J41.0	Simple chronic bronchitis	Diagnosis	ICD-10-CM
J41.1	Mucopurulent chronic bronchitis	Diagnosis	ICD-10-CM
J41.8	Mixed simple and mucopurulent chronic bronchitis	Diagnosis	ICD-10-CM
J42	Unspecified chronic bronchitis	Diagnosis	ICD-10-CM
J43.0	Unilateral pulmonary emphysema [MacLeod's syndrome]	Diagnosis	ICD-10-CM
J43.1	Panlobular emphysema	Diagnosis	ICD-10-CM
J43.2	Centrilobular emphysema	Diagnosis	ICD-10-CM
J43.8	Other emphysema	Diagnosis	ICD-10-CM
J43.9	Emphysema, unspecified	Diagnosis	ICD-10-CM
J44.0	Chronic obstructive pulmonary disease with acute lower respiratory infection	Diagnosis	ICD-10-CM
J44.1	Chronic obstructive pulmonary disease with (acute) exacerbation	Diagnosis	ICD-10-CM
J44.9	Chronic obstructive pulmonary disease, unspecified	Diagnosis	ICD-10-CM
J47.0	Bronchiectasis with acute lower respiratory infection	Diagnosis	ICD-10-CM
J47.1	Bronchiectasis with (acute) exacerbation	Diagnosis	ICD-10-CM

Code	Description	Code Category	Code Type
J47.9	Bronchiectasis, uncomplicated	Diagnosis	ICD-10-CM
J67.0	Farmer's lung	Diagnosis	ICD-10-CM
J67.1	Bagassosis	Diagnosis	ICD-10-CM
J67.2	Bird fancier's lung	Diagnosis	ICD-10-CM
J67.3	Suberosis	Diagnosis	ICD-10-CM
J67.4	Maltworker's lung	Diagnosis	ICD-10-CM
J67.5	Mushroom-worker's lung	Diagnosis	ICD-10-CM
J67.6	Maple-bark-stripper's lung	Diagnosis	ICD-10-CM
J67.7	Air conditioner and humidifier lung	Diagnosis	ICD-10-CM
J67.8	Hypersensitivity pneumonitis due to other organic dusts	Diagnosis	ICD-10-CM
J67.9	Hypersensitivity pneumonitis due to unspecified organic dust	Diagnosis	ICD-10-CM
**Cystic Fibrosis**			
E84	Cystic fibrosis	Diagnosis	ICD-10-CM
E84.0	Cystic fibrosis with pulmonary manifestations	Diagnosis	ICD-10-CM
E84.1	Cystic fibrosis with intestinal manifestations	Diagnosis	ICD-10-CM
E84.11	Meconium ileus in cystic fibrosis	Diagnosis	ICD-10-CM
E84.19	Cystic fibrosis with other intestinal manifestations	Diagnosis	ICD-10-CM
E84.8	Cystic fibrosis with other manifestations	Diagnosis	ICD-10-CM
E84.9	Cystic fibrosis, unspecified	Diagnosis	ICD-10-CM
Z14.1	Cystic fibrosis carrier	Diagnosis	ICD-10-CM
**Tuberculosis**			
010.90	Primary tuberculous infection, unspecified, confirmation unspecified	Diagnosis	ICD-9-CM
011	Pulmonary tuberculosis	Diagnosis	ICD-9-CM
011.0	Tuberculosis of lung, infiltrative	Diagnosis	ICD-9-CM
011.00	Tuberculosis of lung, infiltrative, confirmation unspecified	Diagnosis	ICD-9-CM
011.01	Tuberculosis of lung, infiltrative, bacteriological or histological examination not done	Diagnosis	ICD-9-CM
011.02	Tuberculosis of lung, infiltrative, bacteriological or histological examination unknown (at present)	Diagnosis	ICD-9-CM
011.03	Tuberculosis of lung, infiltrative, tubercle bacilli found (in sputum) by microscopy	Diagnosis	ICD-9-CM
011.04	Tuberculosis of lung, infiltrative, tubercle bacilli not found (in sputum) by microscopy, but found by bacterial culture	Diagnosis	ICD-9-CM
011.05	Tuberculosis of lung, infiltrative, tubercle bacilli not found by bacteriological examination, but tuberculosis confirmed histologically	Diagnosis	ICD-9-CM
011.06	Tuberculosis of lung, infiltrative, tubercle bacilli not found bacteriological or	Diagnosis	ICD-9-CM
011.1	Tuberculosis of lung, nodular	Diagnosis	ICD-9-CM
011.10	Tuberculosis of lung, nodular, confirmation unspecified	Diagnosis	ICD-9-CM
011.11	Tuberculosis of lung, nodular, bacteriological or histological examination not done	Diagnosis	ICD-9-CM
011.12	Tuberculosis of lung, nodular, bacteriological or histological examination unknown (at present)	Diagnosis	ICD-9-CM
011.13	Tuberculosis of lung, nodular, tubercle bacilli found (in sputum) by microscopy	Diagnosis	ICD-9-CM
011.14	Tuberculosis of lung, nodular, tubercle bacilli not found (in sputum) by microscopy,	Diagnosis	ICD-9-CM
011.15	Tuberculosis of lung, nodular, tubercle bacilli not found by bacteriological examination, but tuberculosis confirmed histologically	Diagnosis	ICD-9-CM

Code	Description	Code Category	Code Type
011.16	Tuberculosis of lung, nodular, tubercle bacilli not found by bacteriological or histological examination, but tuberculosis confirmed by other methods [inoculation of animals]	Diagnosis	ICD-9-CM
011.2	Tuberculosis of lung with cavitation	Diagnosis	ICD-9-CM
011.20	Tuberculosis of lung with cavitation, confirmation unspecified	Diagnosis	ICD-9-CM
011.21	Tuberculosis of lung with cavitation, bacteriological or histological examination not done	Diagnosis	ICD-9-CM
011.22	Tuberculosis of lung with cavitation, bacteriological or histological examination unknown (at present)	Diagnosis	ICD-9-CM
011.23	Tuberculosis of lung with cavitation, tubercle bacilli found (in sputum) by microscopy	Diagnosis	ICD-9-CM
011.24	Tuberculosis of lung with cavitation, tubercle bacilli not found (in sputum) by microscopy, but found by bacterial culture	Diagnosis	ICD-9-CM
011.25	Tuberculosis of lung with cavitation, tubercle bacilli not found by bacteriological examination, but tuberculosis confirmed histologically	Diagnosis	ICD-9-CM
011.26	Tuberculosis of lung with cavitation, tubercle bacilli not found by bacteriological or histological examination, but tuberculosis confirmed by other methods [inoculation of animals]	Diagnosis	ICD-9-CM
011.3	Tuberculosis of bronchus	Diagnosis	ICD-9-CM
011.30	Tuberculosis of bronchus, confirmation unspecified	Diagnosis	ICD-9-CM
011.31	Tuberculosis of bronchus, bacteriological or histological examination not done	Diagnosis	ICD-9-CM
011.32	Tuberculosis of bronchus, bacteriological or histological examination unknown (at present)	Diagnosis	ICD-9-CM
011.33	Tuberculosis of bronchus, tubercle bacilli found (in sputum) by microscopy	Diagnosis	ICD-9-CM
011.34	Tuberculosis of bronchus, tubercle bacilli not found (in sputum) by microscopy, but found in bacterial culture	Diagnosis	ICD-9-CM
011.35	Tuberculosis of bronchus, tubercle bacilli not found by bacteriological examination, but tuberculosis confirmed histologically	Diagnosis	ICD-9-CM
011.36	Tuberculosis of bronchus, tubercle bacilli not found by bacteriological or histological examination, but tuberculosis confirmed by other methods [inoculation of animals]	Diagnosis	ICD-9-CM
011.4	Tuberculous fibrosis of lung	Diagnosis	ICD-9-CM
011.40	Tuberculous fibrosis of lung, confirmation unspecified	Diagnosis	ICD-9-CM
011.41	Tuberculous fibrosis of lung, bacteriological or histological examination not done	Diagnosis	ICD-9-CM
011.42	Tuberculous fibrosis of lung, bacteriological or histological examination unknown (at present)	Diagnosis	ICD-9-CM
011.43	Tuberculous fibrosis of lung, tubercle bacilli found (in sputum) by microscopy	Diagnosis	ICD-9-CM
011.44	Tuberculous fibrosis of lung, tubercle bacilli not found (in sputum) by microscopy,	Diagnosis	ICD-9-CM
011.45	Tuberculous fibrosis of lung, tubercle bacilli not found by bacteriological examination, but tuberculosis confirmed histologically	Diagnosis	ICD-9-CM
011.46	Tuberculous fibrosis of lung, tubercle bacilli not found by bacteriological or histological examination, but tuberculosis confirmed by other methods [inoculation of animals]	Diagnosis	ICD-9-CM
011.5	Tuberculous bronchiectasis	Diagnosis	ICD-9-CM
011.50	Tuberculous bronchiectasis, confirmation unspecified	Diagnosis	ICD-9-CM

Code	Description	Code Category	Code Type
011.51	Tuberculous bronchiectasis, bacteriological or histological examination not done	Diagnosis	ICD-9-CM
011.52	Tuberculous bronchiectasis, bacteriological or histological examination unknown (at present)	Diagnosis	ICD-9-CM
011.53	Tuberculous bronchiectasis, tubercle bacilli found (in sputum) by microscopy	Diagnosis	ICD-9-CM
011.54	Tuberculous bronchiectasis, tubercle bacilli not found (in sputum) by microscopy,	Diagnosis	ICD-9-CM
011.55	Tuberculous bronchiectasis, tubercle bacilli not found by bacteriological examination, but tuberculosis confirmed histologically	Diagnosis	ICD-9-CM
011.56	Tuberculous bronchiectasis, tubercle bacilli not found by bacteriological or histological examination, but tuberculosis confirmed by other methods [inoculation of animals]	Diagnosis	ICD-9-CM
011.6	Tuberculous pneumonia (any form)	Diagnosis	ICD-9-CM
011.60	Tuberculous pneumonia (any form), confirmation unspecified	Diagnosis	ICD-9-CM
011.61	Tuberculous pneumonia (any form), bacteriological or histological examination not done	Diagnosis	ICD-9-CM
011.62	Tuberculous pneumonia (any form), bacteriological or histological examination unknown (at present)	Diagnosis	ICD-9-CM
011.63	Tuberculous pneumonia (any form), tubercle bacilli found (in sputum) by microscopy	Diagnosis	ICD-9-CM
011.64	Tuberculous pneumonia (any form), tubercle bacilli not found (in sputum) by microscopy, but found by bacterial culture	Diagnosis	ICD-9-CM
011.65	Tuberculous pneumonia (any form), tubercle bacilli not found by bacteriological examination, but tuberculosis confirmed histologically	Diagnosis	ICD-9-CM
011.66	Tuberculous pneumonia (any form), tubercle bacilli not found by bacteriological or histological examination, but tuberculosis confirmed by other methods [inoculation of animals]	Diagnosis	ICD-9-CM
011.7	Tuberculous pneumothorax	Diagnosis	ICD-9-CM
011.70	Tuberculous pneumothorax, confirmation unspecified	Diagnosis	ICD-9-CM
011.71	Tuberculous pneumothorax, bacteriological or histological examination not done	Diagnosis	ICD-9-CM
011.72	Tuberculous pneumothorax, bacteriological or histological examination unknown (at present)	Diagnosis	ICD-9-CM
011.73	Tuberculous pneumothorax, tubercle bacilli not found (in sputum) by microscopy	Diagnosis	ICD-9-CM
011.74	Tuberculous pneumothorax, tubercle bacilli not found (in sputum) by microscopy,	Diagnosis	ICD-9-CM
011.75	Tuberculous pneumothorax, tubercle bacilli not found by bacteriological examination, but tuberculosis confirmed histologically	Diagnosis	ICD-9-CM
011.76	Tuberculous pneumothorax, tubercle bacilli not found by bacteriological or histological examination but tuberculosis confirmed by other methods [inoculation of animals]	Diagnosis	ICD-9-CM
011.8	Other specified pulmonary tuberculosis	Diagnosis	ICD-9-CM
011.80	Other specified pulmonary tuberculosis, confirmation unspecified	Diagnosis	ICD-9-CM
011.81	Other specified pulmonary tuberculosis, bacteriological or histological examination not done	Diagnosis	ICD-9-CM
011.82	Other specified pulmonary tuberculosis, bacteriological or histological examination unknown (at present)	Diagnosis	ICD-9-CM

Code	Description	Code Category	Code Type
011.83	Other specified pulmonary tuberculosis, tubercle bacilli found (in sputum) by microscopy	Diagnosis	ICD-9-CM
011.84	Other specified pulmonary tuberculosis, tubercle bacilli not found (in sputum) by microscopy, but found by bacterial culture	Diagnosis	ICD-9-CM
011.85	Other specified pulmonary tuberculosis, tubercle bacilli not found by bacteriological examination, but tuberculosis confirmed histologically	Diagnosis	ICD-9-CM
011.86	Other specified pulmonary tuberculosis, tubercle bacilli not found by bacteriological or histological examination, but tuberculosis confirmed by other methods [inoculation of animals]	Diagnosis	ICD-9-CM
011.9	Unspecified pulmonary tuberculosis	Diagnosis	ICD-9-CM
011.90	Unspecified pulmonary tuberculosis, confirmation unspecified	Diagnosis	ICD-9-CM
011.91	Unspecified pulmonary tuberculosis, bacteriological or histological examination not done	Diagnosis	ICD-9-CM
011.92	Unspecified pulmonary tuberculosis, bacteriological or histological examination unknown (at present)	Diagnosis	ICD-9-CM
011.93	Unspecified pulmonary tuberculosis, tubercle bacilli found (in sputum) by microscopy	Diagnosis	ICD-9-CM
011.94	Unspecified pulmonary tuberculosis, tubercle bacilli not found (in sputum) by microscopy, but found by bacterial culture	Diagnosis	ICD-9-CM
011.95	Unspecified pulmonary tuberculosis, tubercle bacilli not found by bacteriological examination, but tuberculosis confirmed histologically	Diagnosis	ICD-9-CM
011.96	Unspecified pulmonary tuberculosis, tubercle bacilli not found by bacteriological or histological examination, but tuberculosis confirmed by other methods [inoculation of animals]	Diagnosis	ICD-9-CM
012	Other respiratory tuberculosis	Diagnosis	ICD-9-CM
012.0	Tuberculous pleurisy	Diagnosis	ICD-9-CM
012.00	Tuberculous pleurisy, confirmation unspecified	Diagnosis	ICD-9-CM
012.01	Tuberculous pleurisy, bacteriological or histological examination not done	Diagnosis	ICD-9-CM
012.02	Tuberculous pleurisy, bacteriological or histological examination unknown (at present)	Diagnosis	ICD-9-CM
012.03	Tuberculous pleurisy, tubercle bacilli found (in sputum) by microscopy	Diagnosis	ICD-9-CM
012.04	Tuberculous pleurisy, tubercle bacilli not found (in sputum) by microscopy, but found by bacterial culture	Diagnosis	ICD-9-CM
012.05	Tuberculous pleurisy, tubercle bacilli not found by bacteriological examination, but tuberculosis confirmed histologically	Diagnosis	ICD-9-CM
012.06	Tuberculous pleurisy, tubercle bacilli not found by bacteriological or histological examination, but tuberculosis confirmed by other methods [inoculation of animals]	Diagnosis	ICD-9-CM
012.2	Isolated tracheal or bronchial tuberculosis	Diagnosis	ICD-9-CM
012.20	Isolated tracheal or bronchial tuberculosis, unspecified	Diagnosis	ICD-9-CM
012.21	Isolated tracheal or bronchial tuberculosis, bacteriological or histological examination not done	Diagnosis	ICD-9-CM
012.22	Isolated tracheal or bronchial tuberculosis, bacteriological or histological examination unknown (at present)	Diagnosis	ICD-9-CM

Code	Description	Code Category	Code Type
012.23	Isolated tracheal or bronchial tuberculosis, tubercle bacilli found (in sputum) by microscopy	Diagnosis	ICD-9-CM
012.24	Isolated tracheal or bronchial tuberculosis, tubercle bacilli not found (in sputum) by microscopy, but found by bacterial culture	Diagnosis	ICD-9-CM
012.25	Isolated tracheal or bronchial tuberculosis, tubercle bacilli not found by bacteriological examination, but tuberculosis confirmed histologically	Diagnosis	ICD-9-CM
012.26	Isolated tracheal or bronchial tuberculosis, tubercle bacilli not found by bacteriological or histological examination, but tuberculosis confirmed by other methods [inoculation of animals]	Diagnosis	ICD-9-CM
012.8	Other specified respiratory tuberculosis	Diagnosis	ICD-9-CM
012.80	Other specified respiratory tuberculosis, confirmation unspecified	Diagnosis	ICD-9-CM
012.81	Other specified respiratory tuberculosis, bacteriological or histological examination not done	Diagnosis	ICD-9-CM
012.82	Other specified respiratory tuberculosis, bacteriological or histological examination unknown (at present)	Diagnosis	ICD-9-CM
012.83	Other specified respiratory tuberculosis, tubercle bacilli found (in sputum) by microscopy	Diagnosis	ICD-9-CM
012.84	Other specified respiratory tuberculosis, tubercle bacilli not found (in sputum) by microscopy, but found by bacterial culture	Diagnosis	ICD-9-CM
012.85	Other specified respiratory tuberculosis, tubercle bacilli not found by bacteriological examination, but tuberculosis confirmed histologically	Diagnosis	ICD-9-CM
012.86	Other specified respiratory tuberculosis, tubercle bacilli not found by bacteriological or histological examination, but tuberculosis confirmed by other methods [inoculation of animals]	Diagnosis	ICD-9-CM
A15.0	Tuberculosis of lung	Diagnosis	ICD-10-CM
A15.5	Tuberculosis of larynx, trachea and bronchus	Diagnosis	ICD-10-CM
A15.6	Tuberculous pleurisy	Diagnosis	ICD-10-CM
A15.7	Primary respiratory tuberculosis	Diagnosis	ICD-10-CM
A15.8	Other respiratory tuberculosis	Diagnosis	ICD-10-CM
A15.9	Respiratory tuberculosis unspecified	Diagnosis	ICD-10-CM
Z86.11	personal history of tuberculosis	Diagnosis	ICD-10-CM
J65	Pneumoconiosis associated with tuberculosis	Diagnosis	ICD-10-CM
**Other Chronic Respiratory Disorders**			
B4481	Allergic bronchopulmonary aspergillosis	Diagnosis	ICD-10-CM
G4734	Idio sleep related nonobstructive alveolar hypoventilation	Diagnosis	ICD-10-CM
G4736	Sleep related hypoventilation in conditions classd elswhr	Diagnosis	ICD-10-CM
G4739	Other sleep apnea	Diagnosis	ICD-10-CM
J61	Pneumoconiosis due to asbestos and other mineral fibers	Diagnosis	ICD-10-CM
J62	Pneumoconiosis due to dust containing silica	Diagnosis	ICD-10-CM
J628	Pneumoconiosis due to other dust containing silica	Diagnosis	ICD-10-CM
J63	Pneumoconiosis due to other inorganic dusts	Diagnosis	ICD-10-CM
J636	Pneumoconiosis due to other specified inorganic dusts	Diagnosis	ICD-10-CM
J65	Pneumoconiosis associated with tuberculosis	Diagnosis	ICD-10-CM
J70	Respiratory conditions due to other external agents	Diagnosis	ICD-10-CM
J701	Chronic and other pulmonary manifestations due to radiatio	Diagnosis	ICD-10-CM

Code	Description	Code Category	Code Type
J703	Chronic drug-induced interstitial lung disorders	Diagnosis	ICD-10-CM
J704	Drug-induced interstitial lung disorders, unspecified	Diagnosis	ICD-10-CM
J705	Respiratory conditions due to smoke inhalation	Diagnosis	ICD-10-CM
J708	Respiratory conditions due to oth external agents	Diagnosis	ICD-10-CM
J709	Respiratory conditions due to unspecified external agent	Diagnosis	ICD-10-CM
J82	Pulmonary eosinophilia, not elsewhere classified	Diagnosis	ICD-10-CM
J828	Pulmonary eosinophilia, not elsewhere classified	Diagnosis	ICD-10-CM
J8289	Other pulmonary eosinophilia, not elsewhere classified	Diagnosis	ICD-10-CM
J84	Other interstitial pulmonary diseases	Diagnosis	ICD-10-CM
J840	Alveolar and parieto-alveolar conditions	Diagnosis	ICD-10-CM
J8409	Other alveolar and parieto-alveolar conditions	Diagnosis	ICD-10-CM
J841	Other interstitial pulmonary diseases with fibrosis	Diagnosis	ICD-10-CM
J8410	Pulmonary fibrosis, unspecified	Diagnosis	ICD-10-CM
J84111	Idiopathic interstitial pneumonia, not otherwise specified	Diagnosis	ICD-10-CM
J84113	Idiopathic non-specific interstitial pneumonitis	Diagnosis	ICD-10-CM
J84115	Respiratory bronchiolitis interstitial lung disease	Diagnosis	ICD-10-CM
J84117	Desquamative interstitial pneumonia	Diagnosis	ICD-10-CM
J8417	Oth interstit pulmon dis w fibrosis in dis classd elswhr	Diagnosis	ICD-10-CM
J84170	Interstit lung dis w progr fibrotic phenotype dis classd e	Diagnosis	ICD-10-CM
J84178	Oth interstit pulmon dis with fibrosis in dis classd elswh	Diagnosis	ICD-10-CM
J848	Other specified interstitial pulmonary diseases	Diagnosis	ICD-10-CM
J8482	Adult pulmonary Langerhans cell histiocytosis	Diagnosis	ICD-10-CM
J8484	Other interstitial lung diseases of childhood	Diagnosis	ICD-10-CM
J84841	Neuroendocrine cell hyperplasia of infancy	Diagnosis	ICD-10-CM
J84842	Pulmonary interstitial glycogenosis	Diagnosis	ICD-10-CM
J84843	Alveolar capillary dysplasia with vein misalignment	Diagnosis	ICD-10-CM
J84848	Other interstitial lung diseases of childhood	Diagnosis	ICD-10-CM
J8489	Other specified interstitial pulmonary diseases	Diagnosis	ICD-10-CM
J849	Interstitial pulmonary disease, unspecified	Diagnosis	ICD-10-CM
J91	Pleural effusion in conditions classified elsewhere	Diagnosis	ICD-10-CM
J920	Pleural plaque with presence of asbestos	Diagnosis	ICD-10-CM
P27	Chronic respiratory disease originating in the perinatal period	Diagnosis	ICD-10-CM
P270	Wilson-Mikity syndrome	Diagnosis	ICD-10-CM
P271	Bronchopulmonary dysplasia origin in the perinatal period	Diagnosis	ICD-10-CM
P278	Oth chronic resp diseases origin in the perinatal period	Diagnosis	ICD-10-CM
P279	Unsp chronic resp disease origin in the perinatal period	Diagnosis	ICD-10-CM
J410	Simple chronic bronchitis	Diagnosis	ICD-10-CM
J411	Mucopurulent chronic bronchitis	Diagnosis	ICD-10-CM
J418	Mixed simple and mucopurulent chronic bronchitis	Diagnosis	ICD-10-CM
J44	Other chronic obstructive pulmonary disease	Diagnosis	ICD-10-CM
J982	Interstitial emphysema	Diagnosis	ICD-10-CM
J983	compensatory emphysema	Diagnosis	ICD-10-CM
J984	other disorders of lung	Diagnosis	ICD-10-CM
J989	Respiratory disease (chronic) NOS	Diagnosis	ICD-10-CM
J961	Chronic respiratory failure	Diagnosis	ICD-10-CM

Code	Description	Code Category	Code Type
	**Diabetes**		
250.01	Diabetes mellitus without mention of complication, type I [juvenile type], not stated as uncontrolled	Diagnosis	ICD-9-CM
250.03	Diabetes mellitus without mention of complication, type I [juvenile type], uncontrolled	Diagnosis	ICD-9-CM
250.11	Diabetes with ketoacidosis, type I [juvenile type], not stated as uncontrolled	Diagnosis	ICD-9-CM
250.13	Diabetes with ketoacidosis, type I [juvenile type], uncontrolled	Diagnosis	ICD-9-CM
250.21	Diabetes with hyperosmolarity, type I [juvenile type], not stated as uncontrolled	Diagnosis	ICD-9-CM
250.23	Diabetes with hyperosmolarity, type I [juvenile type], uncontrolled	Diagnosis	ICD-9-CM
250.31	Diabetes with other coma, type I [juvenile type], not stated as uncontrolled	Diagnosis	ICD-9-CM
250.33	Diabetes with other coma, type I [juvenile type], uncontrolled	Diagnosis	ICD-9-CM
250.41	Diabetes with renal manifestations, type I [juvenile type], not stated as uncontrolled	Diagnosis	ICD-9-CM
250.43	Diabetes with renal manifestations, type I [juvenile type], uncontrolled	Diagnosis	ICD-9-CM
250.51	Diabetes with ophthalmic manifestations, type I [juvenile type], not stated as uncontrolled	Diagnosis	ICD-9-CM
250.53	Diabetes with ophthalmic manifestations, type I [juvenile type], uncontrolled	Diagnosis	ICD-9-CM
250.61	Diabetes with neurological manifestations, type I [juvenile type], not stated as uncontrolled	Diagnosis	ICD-9-CM
250.63	Diabetes with neurological manifestations, type I [juvenile type], uncontrolled	Diagnosis	ICD-9-CM
250.71	Diabetes with peripheral circulatory disorders, type I [juvenile type], not stated as uncontrolled	Diagnosis	ICD-9-CM
250.73	Diabetes with peripheral circulatory disorders, type I [juvenile type], uncontrolled	Diagnosis	ICD-9-CM
250.81	Diabetes with other specified manifestations, type I [juvenile type], not stated as uncontrolled	Diagnosis	ICD-9-CM
250.83	Diabetes with other specified manifestations, type I [juvenile type], uncontrolled	Diagnosis	ICD-9-CM
250.91	Diabetes with unspecified complication, type I [juvenile type], not stated as uncontrolled	Diagnosis	ICD-9-CM
250.93	Diabetes with unspecified complication, type I [juvenile type], uncontrolled	Diagnosis	ICD-9-CM
E10.10	Type 1 diabetes mellitus with ketoacidosis without coma	Diagnosis	ICD-10-CM
E10.11	Type 1 diabetes mellitus with ketoacidosis with coma	Diagnosis	ICD-10-CM
E10.21	Type 1 diabetes mellitus with diabetic nephropathy	Diagnosis	ICD-10-CM
E10.22	Type 1 diabetes mellitus with diabetic chronic kidney disease	Diagnosis	ICD-10-CM
E10.29	Type 1 diabetes mellitus with other diabetic kidney complication	Diagnosis	ICD-10-CM
E10.311	Type 1 diabetes mellitus with unspecified diabetic retinopathy with macular edema	Diagnosis	ICD-10-CM
E10.319	Type 1 diabetes mellitus with unspecified diabetic retinopathy without macular edema	Diagnosis	ICD-10-CM
E10.3211	Type 1 diabetes mellitus with mild nonproliferative diabetic retinopathy with macular edema, right eye	Diagnosis	ICD-10-CM
E10.3212	Type 1 diabetes mellitus with mild nonproliferative diabetic retinopathy with macular edema, left eye	Diagnosis	ICD-10-CM
E10.3213	Type 1 diabetes mellitus with mild nonproliferative diabetic retinopathy with macular edema, bilateral	Diagnosis	ICD-10-CM

Code	Description	Code Category	Code Type
E10.3219	Type 1 diabetes mellitus with mild nonproliferative diabetic retinopathy with macular edema, unspecified eye	Diagnosis	ICD-10-CM
E10.3291	Type 1 diabetes mellitus with mild nonproliferative diabetic retinopathy without macular edema, right eye	Diagnosis	ICD-10-CM
E10.3292	Type 1 diabetes mellitus with mild nonproliferative diabetic retinopathy without macular edema, left eye	Diagnosis	ICD-10-CM
E10.3293	Type 1 diabetes mellitus with mild nonproliferative diabetic retinopathy without macular edema, bilateral	Diagnosis	ICD-10-CM
E10.3299	Type 1 diabetes mellitus with mild nonproliferative diabetic retinopathy without macular edema, unspecified eye	Diagnosis	ICD-10-CM
E10.3311	Type 1 diabetes mellitus with moderate nonproliferative diabetic retinopathy with [illegible]	Diagnosis	ICD-10-CM
E10.3312	Type 1 diabetes mellitus with moderate nonproliferative diabetic retinopathy with macular edema, left eye	Diagnosis	ICD-10-CM
E10.3313	Type 1 diabetes mellitus with moderate nonproliferative diabetic retinopathy with macular edema, bilateral	Diagnosis	ICD-10-CM
E10.3319	Type 1 diabetes mellitus with moderate nonproliferative diabetic retinopathy with macular edema, unspecified eye	Diagnosis	ICD-10-CM
E10.3391	Type 1 diabetes mellitus with moderate nonproliferative diabetic retinopathy without macular edema, right eye	Diagnosis	ICD-10-CM
E10.3392	Type 1 diabetes mellitus with moderate nonproliferative diabetic retinopathy without macular edema, left eye	Diagnosis	ICD-10-CM
E10.3393	Type 1 diabetes mellitus with moderate nonproliferative diabetic retinopathy without macular edema, bilateral	Diagnosis	ICD-10-CM
E10.3399	Type 1 diabetes mellitus with moderate nonproliferative diabetic retinopathy without macular edema, unspecified eye	Diagnosis	ICD-10-CM
E10.3411	Type 1 diabetes mellitus with severe nonproliferative diabetic retinopathy with macular edema, right eye	Diagnosis	ICD-10-CM
E10.3412	Type 1 diabetes mellitus with severe nonproliferative diabetic retinopathy with macular edema, left eye	Diagnosis	ICD-10-CM
E10.3413	Type 1 diabetes mellitus with severe nonproliferative diabetic retinopathy with macular edema, bilateral	Diagnosis	ICD-10-CM
E10.3419	Type 1 diabetes mellitus with severe nonproliferative diabetic retinopathy with macular edema, unspecified eye	Diagnosis	ICD-10-CM
E10.3491	Type 1 diabetes mellitus with severe nonproliferative diabetic retinopathy without macular edema, right eye	Diagnosis	ICD-10-CM
E10.3492	Type 1 diabetes mellitus with severe nonproliferative diabetic retinopathy without macular edema, left eye	Diagnosis	ICD-10-CM
E10.3493	Type 1 diabetes mellitus with severe nonproliferative diabetic retinopathy without macular edema, bilateral	Diagnosis	ICD-10-CM
E10.3499	Type 1 diabetes mellitus with severe nonproliferative diabetic retinopathy without macular edema, unspecified eye	Diagnosis	ICD-10-CM
E10.3511	Type 1 diabetes mellitus with proliferative diabetic retinopathy with macular edema, right eye	Diagnosis	ICD-10-CM
E10.3512	Type 1 diabetes mellitus with proliferative diabetic retinopathy with macular edema, left eye	Diagnosis	ICD-10-CM

Code	Description	Code Category	Code Type
E10.3513	Type 1 diabetes mellitus with proliferative diabetic retinopathy with macular edema, bilateral	Diagnosis	ICD-10-CM
E10.3519	Type 1 diabetes mellitus with proliferative diabetic retinopathy with macular edema, unspecified eye	Diagnosis	ICD-10-CM
E10.3521	Type 1 diabetes mellitus with proliferative diabetic retinopathy with traction retinal detachment involving the macula, right eye	Diagnosis	ICD-10-CM
E10.3522	Type 1 diabetes mellitus with proliferative diabetic retinopathy with traction retinal detachment involving the macula, left eye	Diagnosis	ICD-10-CM
E10.3523	Type 1 diabetes mellitus with proliferative diabetic retinopathy with traction retinal detachment involving the macula, bilateral	Diagnosis	ICD-10-CM
E10.3529	Type 1 diabetes mellitus with proliferative diabetic retinopathy with traction retinal detachment involving the macula, unspecified eye	Diagnosis	ICD-10-CM
E10.3531	Type 1 diabetes mellitus with proliferative diabetic retinopathy with traction retinal detachment not involving the macula, right eye	Diagnosis	ICD-10-CM
E10.3532	Type 1 diabetes mellitus with proliferative diabetic retinopathy with traction retinal detachment not involving the macula, left eye	Diagnosis	ICD-10-CM
E10.3533	Type 1 diabetes mellitus with proliferative diabetic retinopathy with traction retinal detachment not involving the macula, bilateral	Diagnosis	ICD-10-CM
E10.3539	Type 1 diabetes mellitus with proliferative diabetic retinopathy with traction retinal detachment not involving the macula, unspecified eye	Diagnosis	ICD-10-CM
E10.3541	Type 1 diabetes mellitus with proliferative diabetic retinopathy with combined traction retinal detachment and rhegmatogenous retinal detachment, right eye	Diagnosis	ICD-10-CM
E10.3542	Type 1 diabetes mellitus with proliferative diabetic retinopathy with combined traction retinal detachment and rhegmatogenous retinal detachment, left eye	Diagnosis	ICD-10-CM
E10.3543	Type 1 diabetes mellitus with proliferative diabetic retinopathy with combined traction retinal detachment and rhegmatogenous retinal detachment, bilateral	Diagnosis	ICD-10-CM
E10.3549	Type 1 diabetes mellitus with proliferative diabetic retinopathy with combined traction retinal detachment and rhegmatogenous retinal detachment, unspecified eye	Diagnosis	ICD-10-CM
E10.3551	Type 1 diabetes mellitus with stable proliferative diabetic retinopathy, right eye	Diagnosis	ICD-10-CM
E10.3552	Type 1 diabetes mellitus with stable proliferative diabetic retinopathy, left eye	Diagnosis	ICD-10-CM
E10.3553	Type 1 diabetes mellitus with stable proliferative diabetic retinopathy, bilateral	Diagnosis	ICD-10-CM
E10.3559	Type 1 diabetes mellitus with stable proliferative diabetic retinopathy, unspecified eye	Diagnosis	ICD-10-CM
E10.3591	Type 1 diabetes mellitus with proliferative diabetic retinopathy without macular edema, right eye	Diagnosis	ICD-10-CM
E10.3592	Type 1 diabetes mellitus with proliferative diabetic retinopathy without macular edema, left eye	Diagnosis	ICD-10-CM
E10.3593	Type 1 diabetes mellitus with proliferative diabetic retinopathy without macular edema, bilateral	Diagnosis	ICD-10-CM
E10.3599	Type 1 diabetes mellitus with proliferative diabetic retinopathy without macular edema, unspecified eye	Diagnosis	ICD-10-CM
E10.36	Type 1 diabetes mellitus with diabetic cataract	Diagnosis	ICD-10-CM
E10.37X1	Type 1 diabetes mellitus with diabetic macular edema, resolved following treatment, right eye	Diagnosis	ICD-10-CM

Code	Description	Code Category	Code Type
E10.37X2	Type 1 diabetes mellitus with diabetic macular edema, resolved following treatment, left eye	Diagnosis	ICD-10-CM
E10.37X3	Type 1 diabetes mellitus with diabetic macular edema, resolved following treatment, bilateral	Diagnosis	ICD-10-CM
E10.37X9	Type 1 diabetes mellitus with diabetic macular edema, resolved following treatment, unspecified eye	Diagnosis	ICD-10-CM
E10.39	Type 1 diabetes mellitus with other diabetic ophthalmic complication	Diagnosis	ICD-10-CM
E10.40	Type 1 diabetes mellitus with diabetic neuropathy, unspecified	Diagnosis	ICD-10-CM
E10.41	Type 1 diabetes mellitus with diabetic mononeuropathy	Diagnosis	ICD-10-CM
E10.42	Type 1 diabetes mellitus with diabetic polyneuropathy	Diagnosis	ICD-10-CM
E10.43	Type 1 diabetes mellitus with diabetic autonomic (poly)neuropathy	Diagnosis	ICD-10-CM
E10.44	Type 1 diabetes mellitus with diabetic amyotrophy	Diagnosis	ICD-10-CM
E10.49	Type 1 diabetes mellitus with other diabetic neurological complication	Diagnosis	ICD-10-CM
E10.51	Type 1 diabetes mellitus with diabetic peripheral angiopathy without gangrene	Diagnosis	ICD-10-CM
E10.52	Type 1 diabetes mellitus with diabetic peripheral angiopathy with gangrene	Diagnosis	ICD-10-CM
E10.59	Type 1 diabetes mellitus with other circulatory complications	Diagnosis	ICD-10-CM
E10.610	Type 1 diabetes mellitus with diabetic neuropathic arthropathy	Diagnosis	ICD-10-CM
E10.618	Type 1 diabetes mellitus with other diabetic arthropathy	Diagnosis	ICD-10-CM
E10.620	Type 1 diabetes mellitus with diabetic dermatitis	Diagnosis	ICD-10-CM
E10.621	Type 1 diabetes mellitus with foot ulcer	Diagnosis	ICD-10-CM
E10.622	Type 1 diabetes mellitus with other skin ulcer	Diagnosis	ICD-10-CM
E10.628	Type 1 diabetes mellitus with other skin complications	Diagnosis	ICD-10-CM
E10.630	Type 1 diabetes mellitus with periodontal disease	Diagnosis	ICD-10-CM
E10.638	Type 1 diabetes mellitus with other oral complications	Diagnosis	ICD-10-CM
E10.641	Type 1 diabetes mellitus with hypoglycemia with coma	Diagnosis	ICD-10-CM
E10.649	Type 1 diabetes mellitus with hypoglycemia without coma	Diagnosis	ICD-10-CM
E10.65	Type 1 diabetes mellitus with hyperglycemia	Diagnosis	ICD-10-CM
E10.69	Type 1 diabetes mellitus with other specified complication	Diagnosis	ICD-10-CM
E10.8	Type 1 diabetes mellitus with unspecified complications	Diagnosis	ICD-10-CM
E10.9	Type 1 diabetes mellitus without complications	Diagnosis	ICD-10-CM
250.00	Diabetes mellitus without mention of complication, type II or unspecified type, not stated as uncontrolled	Diagnosis	ICD-9-CM
250.10	Diabetes with ketoacidosis, type II or unspecified type, not stated as uncontrolled	Diagnosis	ICD-9-CM
250.20	Diabetes with hyperosmolarity, type II or unspecified type, not stated as uncontrolled	Diagnosis	ICD-9-CM
250.30	Diabetes with other coma, type II or unspecified type, not stated as uncontrolled	Diagnosis	ICD-9-CM
250.40	Diabetes with renal manifestations, type II or unspecified type, not stated as uncontrolled	Diagnosis	ICD-9-CM
250.50	Diabetes with ophthalmic manifestations, type II or unspecified type, not stated as uncontrolled	Diagnosis	ICD-9-CM
250.60	Diabetes with neurological manifestations, type II or unspecified type, not stated as uncontrolled	Diagnosis	ICD-9-CM
250.70	Diabetes with peripheral circulatory disorders, type II or unspecified type, not stated as uncontrolled	Diagnosis	ICD-9-CM

Code	Description	Code Category	Code Type
250.80	Diabetes with other specified manifestations, type II or unspecified type, not stated as uncontrolled	Diagnosis	ICD-9-CM
250.90	Diabetes with unspecified complication, type II or unspecified type, not stated as uncontrolled	Diagnosis	ICD-9-CM
250.02	Diabetes mellitus without mention of complication, type II or unspecified type, uncontrolled	Diagnosis	ICD-9-CM
250.12	Diabetes with ketoacidosis, type II or unspecified type, uncontrolled	Diagnosis	ICD-9-CM
250.22	Diabetes with hyperosmolarity, type II or unspecified type, uncontrolled	Diagnosis	ICD-9-CM
250.32	Diabetes with other coma, type II or unspecified type, uncontrolled	Diagnosis	ICD-9-CM
250.42	Diabetes with renal manifestations, type II or unspecified type, uncontrolled	Diagnosis	ICD-9-CM
250.52	Diabetes with ophthalmic manifestations, type II or unspecified type, uncontrolled	Diagnosis	ICD-9-CM
250.62	Diabetes with neurological manifestations, type II or unspecified type, uncontrolled	Diagnosis	ICD-9-CM
250.72	Diabetes with peripheral circulatory disorders, type II or unspecified type, uncontrolled	Diagnosis	ICD-9-CM
250.82	Diabetes with other specified manifestations, type II or unspecified type, uncontrolled	Diagnosis	ICD-9-CM
250.92	Diabetes with unspecified complication, type II or unspecified type, uncontrolled	Diagnosis	ICD-9-CM
E11.00	Type 2 diabetes mellitus with hyperosmolarity without nonketotic hyperglycemic-hyperosmolar coma (NKHHC)	Diagnosis	ICD-10-CM
E11.01	Type 2 diabetes mellitus with hyperosmolarity with coma	Diagnosis	ICD-10-CM
E11.10	Type 2 diabetes mellitus with ketoacidosis without coma	Diagnosis	ICD-10-CM
E11.11	Type 2 diabetes mellitus with ketoacidosis with coma	Diagnosis	ICD-10-CM
E11.21	Type 2 diabetes mellitus with diabetic nephropathy	Diagnosis	ICD-10-CM
E11.22	Type 2 diabetes mellitus with diabetic chronic kidney disease	Diagnosis	ICD-10-CM
E11.29	Type 2 diabetes mellitus with other diabetic kidney complication	Diagnosis	ICD-10-CM
E11.311	Type 2 diabetes mellitus with unspecified diabetic retinopathy with macular edema	Diagnosis	ICD-10-CM
E11.319	Type 2 diabetes mellitus with unspecified diabetic retinopathy without macular edema	Diagnosis	ICD-10-CM
E11.3211	Type 2 diabetes mellitus with mild nonproliferative diabetic retinopathy with macular edema, right eye	Diagnosis	ICD-10-CM
E11.3212	Type 2 diabetes mellitus with mild nonproliferative diabetic retinopathy with macular edema, left eye	Diagnosis	ICD-10-CM
E11.3213	Type 2 diabetes mellitus with mild nonproliferative diabetic retinopathy with macular edema, bilateral	Diagnosis	ICD-10-CM
E11.3219	Type 2 diabetes mellitus with mild nonproliferative diabetic retinopathy with macular edema, unspecified eye	Diagnosis	ICD-10-CM
E11.3291	Type 2 diabetes mellitus with mild nonproliferative diabetic retinopathy without macular edema, right eye	Diagnosis	ICD-10-CM
E11.3292	Type 2 diabetes mellitus with mild nonproliferative diabetic retinopathy without macular edema, left eye	Diagnosis	ICD-10-CM
E11.3293	Type 2 diabetes mellitus with mild nonproliferative diabetic retinopathy without macular edema, bilateral	Diagnosis	ICD-10-CM

Code	Description	Code Category	Code Type
E11.3299	Type 2 diabetes mellitus with mild nonproliferative diabetic retinopathy without macular edema, unspecified eye	Diagnosis	ICD-10-CM
E11.3311	Type 2 diabetes mellitus with moderate nonproliferative diabetic retinopathy with macular edema, right eye	Diagnosis	ICD-10-CM
E11.3312	Type 2 diabetes mellitus with moderate nonproliferative diabetic retinopathy with macular edema, left eye	Diagnosis	ICD-10-CM
E11.3313	Type 2 diabetes mellitus with moderate nonproliferative diabetic retinopathy with macular edema, bilateral	Diagnosis	ICD-10-CM
E11.3319	Type 2 diabetes mellitus with moderate nonproliferative diabetic retinopathy with macular edema, unspecified eye	Diagnosis	ICD-10-CM
E11.3391	Type 2 diabetes mellitus with moderate nonproliferative diabetic retinopathy without macular edema, right eye	Diagnosis	ICD-10-CM
E11.3392	Type 2 diabetes mellitus with moderate nonproliferative diabetic retinopathy without macular edema, left eye	Diagnosis	ICD-10-CM
E11.3393	Type 2 diabetes mellitus with moderate nonproliferative diabetic retinopathy without macular edema, bilateral	Diagnosis	ICD-10-CM
E11.3399	Type 2 diabetes mellitus with moderate nonproliferative diabetic retinopathy without macular edema, unspecified eye	Diagnosis	ICD-10-CM
E11.3411	Type 2 diabetes mellitus with severe nonproliferative diabetic retinopathy with macular edema, right eye	Diagnosis	ICD-10-CM
E11.3412	Type 2 diabetes mellitus with severe nonproliferative diabetic retinopathy with macular edema, left eye	Diagnosis	ICD-10-CM
E11.3413	Type 2 diabetes mellitus with severe nonproliferative diabetic retinopathy with macular edema, bilateral	Diagnosis	ICD-10-CM
E11.3419	Type 2 diabetes mellitus with severe nonproliferative diabetic retinopathy with macular edema, unspecified eye	Diagnosis	ICD-10-CM
E11.3491	Type 2 diabetes mellitus with severe nonproliferative diabetic retinopathy without macular edema, right eye	Diagnosis	ICD-10-CM
E11.3492	Type 2 diabetes mellitus with severe nonproliferative diabetic retinopathy without macular edema, left eye	Diagnosis	ICD-10-CM
E11.3493	Type 2 diabetes mellitus with severe nonproliferative diabetic retinopathy without macular edema, bilateral	Diagnosis	ICD-10-CM
E11.3499	Type 2 diabetes mellitus with severe nonproliferative diabetic retinopathy without macular edema, unspecified eye	Diagnosis	ICD-10-CM
E11.3511	Type 2 diabetes mellitus with proliferative diabetic retinopathy with macular edema, right eye	Diagnosis	ICD-10-CM
E11.3512	Type 2 diabetes mellitus with proliferative diabetic retinopathy with macular edema, left eye	Diagnosis	ICD-10-CM
E11.3513	Type 2 diabetes mellitus with proliferative diabetic retinopathy with macular edema, bilateral	Diagnosis	ICD-10-CM
E11.3519	Type 2 diabetes mellitus with proliferative diabetic retinopathy with macular edema, unspecified eye	Diagnosis	ICD-10-CM
E11.3521	Type 2 diabetes mellitus with proliferative diabetic retinopathy with traction retinal detachment involving the macula, right eye	Diagnosis	ICD-10-CM

Code	Description	Code Category	Code Type
E11.3522	Type 2 diabetes mellitus with proliferative diabetic retinopathy with traction retinal detachment involving the macula, left eye	Diagnosis	ICD-10-CM
E11.3523	Type 2 diabetes mellitus with proliferative diabetic retinopathy with traction retinal detachment involving the macula, bilateral	Diagnosis	ICD-10-CM
E11.3529	Type 2 diabetes mellitus with proliferative diabetic retinopathy with traction retinal detachment involving the macula, unspecified eye	Diagnosis	ICD-10-CM
E11.3531	Type 2 diabetes mellitus with proliferative diabetic retinopathy with traction retinal detachment not involving the macula, right eye	Diagnosis	ICD-10-CM
E11.3532	Type 2 diabetes mellitus with proliferative diabetic retinopathy with traction retinal detachment not involving the macula, left eye	Diagnosis	ICD-10-CM
E11.3533	Type 2 diabetes mellitus with proliferative diabetic retinopathy with traction retinal detachment not involving the macula, bilateral	Diagnosis	ICD-10-CM
E11.3539	Type 2 diabetes mellitus with proliferative diabetic retinopathy with traction retinal detachment not involving the macula, unspecified eye	Diagnosis	ICD-10-CM
E11.3541	Type 2 diabetes mellitus with proliferative diabetic retinopathy with combined traction retinal detachment and rhegmatogenous retinal detachment, right eye	Diagnosis	ICD-10-CM
E11.3542	Type 2 diabetes mellitus with proliferative diabetic retinopathy with combined traction retinal detachment and rhegmatogenous retinal detachment, left eye	Diagnosis	ICD-10-CM
E11.3543	Type 2 diabetes mellitus with proliferative diabetic retinopathy with combined traction retinal detachment and rhegmatogenous retinal detachment, bilateral	Diagnosis	ICD-10-CM
E11.3549	Type 2 diabetes mellitus with proliferative diabetic retinopathy with combined traction retinal detachment and rhegmatogenous retinal detachment, unspecified eye	Diagnosis	ICD-10-CM
E11.3551	Type 2 diabetes mellitus with stable proliferative diabetic retinopathy, right eye	Diagnosis	ICD-10-CM
E11.3552	Type 2 diabetes mellitus with stable proliferative diabetic retinopathy, left eye	Diagnosis	ICD-10-CM
E11.3553	Type 2 diabetes mellitus with stable proliferative diabetic retinopathy, bilateral	Diagnosis	ICD-10-CM
E11.3559	Type 2 diabetes mellitus with stable proliferative diabetic retinopathy, unspecified eye	Diagnosis	ICD-10-CM
E11.3591	Type 2 diabetes mellitus with proliferative diabetic retinopathy without macular edema, right eye	Diagnosis	ICD-10-CM
E11.3592	Type 2 diabetes mellitus with proliferative diabetic retinopathy without macular edema, left eye	Diagnosis	ICD-10-CM
E11.3593	Type 2 diabetes mellitus with proliferative diabetic retinopathy without macular edema, bilateral	Diagnosis	ICD-10-CM
E11.3599	Type 2 diabetes mellitus with proliferative diabetic retinopathy without macular edema, unspecified eye	Diagnosis	ICD-10-CM
E11.36	Type 2 diabetes mellitus with diabetic cataract	Diagnosis	ICD-10-CM
E11.37X1	Type 2 diabetes mellitus with diabetic macular edema, resolved following treatment, right eye	Diagnosis	ICD-10-CM
E11.37X2	Type 2 diabetes mellitus with diabetic macular edema, resolved following treatment, left eye	Diagnosis	ICD-10-CM
E11.37X3	Type 2 diabetes mellitus with diabetic macular edema, resolved following treatment, bilateral	Diagnosis	ICD-10-CM
E11.37X9	Type 2 diabetes mellitus with diabetic macular edema, resolved following treatment, unspecified eye	Diagnosis	ICD-10-CM

Code	Description	Code Category	Code Type
E11.39	Type 2 diabetes mellitus with other diabetic ophthalmic complication	Diagnosis	ICD-10-CM
E11.40	Type 2 diabetes mellitus with diabetic neuropathy, unspecified	Diagnosis	ICD-10-CM
E11.41	Type 2 diabetes mellitus with diabetic mononeuropathy	Diagnosis	ICD-10-CM
E11.42	Type 2 diabetes mellitus with diabetic polyneuropathy	Diagnosis	ICD-10-CM
E11.43	Type 2 diabetes mellitus with diabetic autonomic (poly)neuropathy	Diagnosis	ICD-10-CM
E11.44	Type 2 diabetes mellitus with diabetic amyotrophy	Diagnosis	ICD-10-CM
E11.49	Type 2 diabetes mellitus with other diabetic neurological complication	Diagnosis	ICD-10-CM
E11.51	Type 2 diabetes mellitus with diabetic peripheral angiopathy without gangrene	Diagnosis	ICD-10-CM
E11.52	Type 2 diabetes mellitus with diabetic peripheral angiopathy with gangrene	Diagnosis	ICD-10-CM
E11.59	Type 2 diabetes mellitus with other circulatory complications	Diagnosis	ICD-10-CM
E11.610	Type 2 diabetes mellitus with diabetic neuropathic arthropathy	Diagnosis	ICD-10-CM
E11.618	Type 2 diabetes mellitus with other diabetic arthropathy	Diagnosis	ICD-10-CM
E11.620	Type 2 diabetes mellitus with diabetic dermatitis	Diagnosis	ICD-10-CM
E11.621	Type 2 diabetes mellitus with foot ulcer	Diagnosis	ICD-10-CM
E11.622	Type 2 diabetes mellitus with other skin ulcer	Diagnosis	ICD-10-CM
E11.628	Type 2 diabetes mellitus with other skin complications	Diagnosis	ICD-10-CM
E11.630	Type 2 diabetes mellitus with periodontal disease	Diagnosis	ICD-10-CM
E11.638	Type 2 diabetes mellitus with other oral complications	Diagnosis	ICD-10-CM
E11.641	Type 2 diabetes mellitus with hypoglycemia with coma	Diagnosis	ICD-10-CM
E11.649	Type 2 diabetes mellitus with hypoglycemia without coma	Diagnosis	ICD-10-CM
E11.65	Type 2 diabetes mellitus with hyperglycemia	Diagnosis	ICD-10-CM
E11.69	Type 2 diabetes mellitus with other specified complication	Diagnosis	ICD-10-CM
E11.8	Type 2 diabetes mellitus with unspecified complications	Diagnosis	ICD-10-CM
E11.9	Type 2 diabetes mellitus without complications	Diagnosis	ICD-10-CM
E13.00	Other specified diabetes mellitus with hyperosmolarity without nonketotic hyperglycemic-hyperosmolar coma (NKHHC)	Diagnosis	ICD-10-CM
E13.01	Other specified diabetes mellitus with hyperosmolarity with coma	Diagnosis	ICD-10-CM
E13.10	Other specified diabetes mellitus with ketoacidosis without coma	Diagnosis	ICD-10-CM
E13.11	Other specified diabetes mellitus with ketoacidosis with coma	Diagnosis	ICD-10-CM
E13.21	Other specified diabetes mellitus with diabetic nephropathy	Diagnosis	ICD-10-CM
E13.22	Other specified diabetes mellitus with diabetic chronic kidney disease	Diagnosis	ICD-10-CM
E13.29	Other specified diabetes mellitus with other diabetic kidney complication	Diagnosis	ICD-10-CM
E13.311	Other specified diabetes mellitus with unspecified diabetic retinopathy with macular edema	Diagnosis	ICD-10-CM
E13.319	Other specified diabetes mellitus with unspecified diabetic retinopathy without macular edema	Diagnosis	ICD-10-CM
E13.3211	Other specified diabetes mellitus with mild nonproliferative diabetic retinopathy with macular edema, right eye	Diagnosis	ICD-10-CM
E13.3212	Other specified diabetes mellitus with mild nonproliferative diabetic retinopathy with macular edema, left eye	Diagnosis	ICD-10-CM
E13.3213	Other specified diabetes mellitus with mild nonproliferative diabetic retinopathy with macular edema, bilateral	Diagnosis	ICD-10-CM
E13.3219	Other specified diabetes mellitus with mild nonproliferative diabetic retinopathy with macular edema, unspecified eye	Diagnosis	ICD-10-CM

Code	Description	Code Category	Code Type
E13.3291	Other specified diabetes mellitus with mild nonproliferative diabetic retinopathy without macular edema, right eye	Diagnosis	ICD-10-CM
E13.3292	Other specified diabetes mellitus with mild nonproliferative diabetic retinopathy without macular edema, left eye	Diagnosis	ICD-10-CM
E13.3293	Other specified diabetes mellitus with mild nonproliferative diabetic retinopathy without macular edema, bilateral	Diagnosis	ICD-10-CM
E13.3299	Other specified diabetes mellitus with mild nonproliferative diabetic retinopathy without macular edema, unspecified eye	Diagnosis	ICD-10-CM
E13.3311	Other specified diabetes mellitus with moderate nonproliferative diabetic retinopathy with macular edema, right eye	Diagnosis	ICD-10-CM
E13.3312	Other specified diabetes mellitus with moderate nonproliferative diabetic retinopathy with macular edema, left eye	Diagnosis	ICD-10-CM
E13.3313	Other specified diabetes mellitus with moderate nonproliferative diabetic retinopathy with macular edema, bilateral	Diagnosis	ICD-10-CM
E13.3319	Other specified diabetes mellitus with moderate nonproliferative diabetic retinopathy with macular edema, unspecified eye	Diagnosis	ICD-10-CM
E13.3391	Other specified diabetes mellitus with moderate nonproliferative diabetic retinopathy without macular edema, right eye	Diagnosis	ICD-10-CM
E13.3392	Other specified diabetes mellitus with moderate nonproliferative diabetic retinopathy without macular edema, left eye	Diagnosis	ICD-10-CM
E13.3393	Other specified diabetes mellitus with moderate nonproliferative diabetic retinopathy without macular edema, bilateral	Diagnosis	ICD-10-CM
E13.3399	Other specified diabetes mellitus with moderate nonproliferative diabetic retinopathy without macular edema, unspecified eye	Diagnosis	ICD-10-CM
E13.3411	Other specified diabetes mellitus with severe nonproliferative diabetic retinopathy with macular edema, right eye	Diagnosis	ICD-10-CM
E13.3412	Other specified diabetes mellitus with severe nonproliferative diabetic retinopathy with macular edema, left eye	Diagnosis	ICD-10-CM
E13.3413	Other specified diabetes mellitus with severe nonproliferative diabetic retinopathy with macular edema, bilateral	Diagnosis	ICD-10-CM
E13.3419	Other specified diabetes mellitus with severe nonproliferative diabetic retinopathy with macular edema, unspecified eye	Diagnosis	ICD-10-CM
E13.3491	Other specified diabetes mellitus with severe nonproliferative diabetic retinopathy without macular edema, right eye	Diagnosis	ICD-10-CM
E13.3492	Other specified diabetes mellitus with severe nonproliferative diabetic retinopathy without macular edema, left eye	Diagnosis	ICD-10-CM
E13.3493	Other specified diabetes mellitus with severe nonproliferative diabetic retinopathy without macular edema, bilateral	Diagnosis	ICD-10-CM
E13.3499	Other specified diabetes mellitus with severe nonproliferative diabetic retinopathy without macular edema, unspecified eye	Diagnosis	ICD-10-CM
E13.3511	Other specified diabetes mellitus with proliferative diabetic retinopathy with macular edema, right eye	Diagnosis	ICD-10-CM
E13.3512	Other specified diabetes mellitus with proliferative diabetic retinopathy with macular edema, left eye	Diagnosis	ICD-10-CM

Code	Description	Code Category	Code Type
E13.3513	Other specified diabetes mellitus with proliferative diabetic retinopathy with macular edema, bilateral	Diagnosis	ICD-10-CM
E13.3519	Other specified diabetes mellitus with proliferative diabetic retinopathy with macular edema, unspecified eye	Diagnosis	ICD-10-CM
E13.3521	Other specified diabetes mellitus with proliferative diabetic retinopathy with traction retinal detachment involving the macula, right eye	Diagnosis	ICD-10-CM
E13.3522	Other specified diabetes mellitus with proliferative diabetic retinopathy with traction retinal detachment involving the macula, left eye	Diagnosis	ICD-10-CM
E13.3523	Other specified diabetes mellitus with proliferative diabetic retinopathy with traction retinal detachment involving the macula, bilateral	Diagnosis	ICD-10-CM
E13.3529	Other specified diabetes mellitus with proliferative diabetic retinopathy with traction retinal detachment involving the macula, unspecified eye	Diagnosis	ICD-10-CM
E13.3531	Other specified diabetes mellitus with proliferative diabetic retinopathy with traction retinal detachment not involving the macula, right eye	Diagnosis	ICD-10-CM
E13.3532	Other specified diabetes mellitus with proliferative diabetic retinopathy with traction retinal detachment not involving the macula, left eye	Diagnosis	ICD-10-CM
E13.3533	Other specified diabetes mellitus with proliferative diabetic retinopathy with traction retinal detachment not involving the macula, bilateral	Diagnosis	ICD-10-CM
E13.3539	Other specified diabetes mellitus with proliferative diabetic retinopathy with traction retinal detachment not involving the macula, unspecified eye	Diagnosis	ICD-10-CM
E13.3541	Other specified diabetes mellitus with proliferative diabetic retinopathy with combined traction retinal detachment and rhegmatogenous retinal detachment, right eye	Diagnosis	ICD-10-CM
E13.3542	Other specified diabetes mellitus with proliferative diabetic retinopathy with combined traction retinal detachment and rhegmatogenous retinal detachment, left eye	Diagnosis	ICD-10-CM
E13.3543	Other specified diabetes mellitus with proliferative diabetic retinopathy with combined traction retinal detachment and rhegmatogenous retinal detachment, bilateral	Diagnosis	ICD-10-CM
E13.3549	Other specified diabetes mellitus with proliferative diabetic retinopathy with combined traction retinal detachment and rhegmatogenous retinal detachment, unspecified eye	Diagnosis	ICD-10-CM
E13.3551	Other specified diabetes mellitus with stable proliferative diabetic retinopathy, right eye	Diagnosis	ICD-10-CM
E13.3552	Other specified diabetes mellitus with stable proliferative diabetic retinopathy, left eye	Diagnosis	ICD-10-CM
E13.3553	Other specified diabetes mellitus with stable proliferative diabetic retinopathy, bilateral	Diagnosis	ICD-10-CM
E13.3559	Other specified diabetes mellitus with stable proliferative diabetic retinopathy, unspecified eye	Diagnosis	ICD-10-CM
E13.3591	Other specified diabetes mellitus with proliferative diabetic retinopathy without macular edema, right eye	Diagnosis	ICD-10-CM
E13.3592	Other specified diabetes mellitus with proliferative diabetic retinopathy without macular edema, left eye	Diagnosis	ICD-10-CM

Code	Description	Code Category	Code Type
E13.3593	Other specified diabetes mellitus with proliferative diabetic retinopathy without macular edema, bilateral	Diagnosis	ICD-10-CM
E13.3599	Other specified diabetes mellitus with proliferative diabetic retinopathy without macular edema, unspecified eye	Diagnosis	ICD-10-CM
E13.36	Other specified diabetes mellitus with diabetic cataract	Diagnosis	ICD-10-CM
E13.37X1	Other specified diabetes mellitus with diabetic macular edema, resolved following treatment, right eye	Diagnosis	ICD-10-CM
E13.37X2	Other specified diabetes mellitus with diabetic macular edema, resolved following treatment, left eye	Diagnosis	ICD-10-CM
E13.37X3	Other specified diabetes mellitus with diabetic macular edema, resolved following treatment, bilateral	Diagnosis	ICD-10-CM
E13.37X9	Other specified diabetes mellitus with diabetic macular edema, resolved following treatment, unspecified eye	Diagnosis	ICD-10-CM
E13.39	Other specified diabetes mellitus with other diabetic ophthalmic complication	Diagnosis	ICD-10-CM
E13.40	Other specified diabetes mellitus with diabetic neuropathy, unspecified	Diagnosis	ICD-10-CM
E13.41	Other specified diabetes mellitus with diabetic mononeuropathy	Diagnosis	ICD-10-CM
E13.42	Other specified diabetes mellitus with diabetic polyneuropathy	Diagnosis	ICD-10-CM
E13.43	Other specified diabetes mellitus with diabetic autonomic (poly)neuropathy	Diagnosis	ICD-10-CM
E13.44	Other specified diabetes mellitus with diabetic amyotrophy	Diagnosis	ICD-10-CM
E13.49	Other specified diabetes mellitus with other diabetic neurological complication	Diagnosis	ICD-10-CM
E13.51	Other specified diabetes mellitus with diabetic peripheral angiopathy without gangrene	Diagnosis	ICD-10-CM
E13.52	Other specified diabetes mellitus with diabetic peripheral angiopathy with gangrene	Diagnosis	ICD-10-CM
E13.59	Other specified diabetes mellitus with other circulatory complications	Diagnosis	ICD-10-CM
E13.610	Other specified diabetes mellitus with diabetic neuropathic arthropathy	Diagnosis	ICD-10-CM
E13.618	Other specified diabetes mellitus with other diabetic arthropathy	Diagnosis	ICD-10-CM
E13.620	Other specified diabetes mellitus with diabetic dermatitis	Diagnosis	ICD-10-CM
E13.621	Other specified diabetes mellitus with foot ulcer	Diagnosis	ICD-10-CM
E13.622	Other specified diabetes mellitus with other skin ulcer	Diagnosis	ICD-10-CM
E13.628	Other specified diabetes mellitus with other skin complications	Diagnosis	ICD-10-CM
E13.630	Other specified diabetes mellitus with periodontal disease	Diagnosis	ICD-10-CM
E13.638	Other specified diabetes mellitus with other oral complications	Diagnosis	ICD-10-CM
E13.641	Other specified diabetes mellitus with hypoglycemia with coma	Diagnosis	ICD-10-CM
E13.649	Other specified diabetes mellitus with hypoglycemia without coma	Diagnosis	ICD-10-CM
E13.65	Other specified diabetes mellitus with hyperglycemia	Diagnosis	ICD-10-CM
E13.69	Other specified diabetes mellitus with other specified complication	Diagnosis	ICD-10-CM
E13.8	Other specified diabetes mellitus with unspecified complications	Diagnosis	ICD-10-CM
E13.9	Other specified diabetes mellitus without complications	Diagnosis	ICD-10-CM
648.0	Maternal diabetes mellitus complicating pregnancy, childbirth, or the puerperium	Diagnosis	ICD-9-CM
648.00	Maternal diabetes mellitus, complicating pregnancy, childbirth, or the puerperium, unspecified as to episode of care	Diagnosis	ICD-9-CM
648.01	Maternal diabetes mellitus with delivery	Diagnosis	ICD-9-CM
648.02	Maternal diabetes mellitus with delivery, with current postpartum complication	Diagnosis	ICD-9-CM
648.03	Maternal diabetes mellitus, antepartum	Diagnosis	ICD-9-CM

Code	Description	Code Category	Code Type
648.04	Maternal diabetes mellitus, complicating pregnancy, childbirth, or the puerperium, postpartum condition or complication	Diagnosis	ICD-9-CM
648.81	Abnormal maternal glucose tolerance, with delivery	Diagnosis	ICD-9-CM
648.82	Abnormal maternal glucose tolerance, with delivery, with current postpartum complication	Diagnosis	ICD-9-CM
648.83	Abnormal maternal glucose tolerance, antepartum	Diagnosis	ICD-9-CM
648.84	Abnormal maternal glucose tolerance complicating pregnancy, childbirth, or the puerperium, postpartum condition or complication	Diagnosis	ICD-9-CM
O24319	Unspecified pre-existing diabetes mellitus in pregnancy, unspecified trimester	Diagnosis	ICD-10-CM
O2432	Unspecified pre-existing diabetes mellitus in childbirth	Diagnosis	ICD-10-CM
O2492	Unspecified diabetes mellitus in childbirth	Diagnosis	ICD-10-CM
O24913	Unspecified diabetes mellitus in pregnancy, third trimester	Diagnosis	ICD-10-CM
O24912	Unspecified diabetes mellitus in pregnancy, second trimester	Diagnosis	ICD-10-CM
O24911	Unspecified diabetes mellitus in pregnancy, first trimester	Diagnosis	ICD-10-CM
O2493	Unspecified diabetes mellitus in the puerperium	Diagnosis	ICD-10-CM
O24019	Pre-existing type 1 diabetes mellitus, in pregnancy, unspecified trimester	Diagnosis	ICD-10-CM
O24919	Unspecified diabetes mellitus in pregnancy, unspecified trimester	Diagnosis	ICD-10-CM
O24819	Other pre-existing diabetes mellitus in pregnancy, unspecified trimester	Diagnosis	ICD-10-CM
O24119	Pre-existing type 2 diabetes mellitus, in pregnancy, unspecified trimester	Diagnosis	ICD-10-CM
O24011	Pre-existing type 1 diabetes mellitus, in pregnancy, first trimester	Diagnosis	ICD-10-CM
O2482	Other pre-existing diabetes mellitus in childbirth	Diagnosis	ICD-10-CM
O24813	Other pre-existing diabetes mellitus in pregnancy, third trimester	Diagnosis	ICD-10-CM
O24812	Other pre-existing diabetes mellitus in pregnancy, second trimester	Diagnosis	ICD-10-CM
O24811	Other pre-existing diabetes mellitus in pregnancy, first trimester	Diagnosis	ICD-10-CM
O24313	Unspecified pre-existing diabetes mellitus in pregnancy, third trimester	Diagnosis	ICD-10-CM
O24312	Unspecified pre-existing diabetes mellitus in pregnancy, second trimester	Diagnosis	ICD-10-CM
O24311	Unspecified pre-existing diabetes mellitus in pregnancy, first trimester	Diagnosis	ICD-10-CM
O2412	Pre-existing type 2 diabetes mellitus, in childbirth	Diagnosis	ICD-10-CM
O24113	Pre-existing type 2 diabetes mellitus, in pregnancy, third trimester	Diagnosis	ICD-10-CM
O24112	Pre-existing type 2 diabetes mellitus, in pregnancy, second trimester	Diagnosis	ICD-10-CM
O24111	Pre-existing type 2 diabetes mellitus, in pregnancy, first trimester	Diagnosis	ICD-10-CM
O2402	Pre-existing type 1 diabetes mellitus, in childbirth	Diagnosis	ICD-10-CM
O24013	Pre-existing type 1 diabetes mellitus, in pregnancy, third trimester	Diagnosis	ICD-10-CM
O24012	Pre-existing type 1 diabetes mellitus, in pregnancy, second trimester	Diagnosis	ICD-10-CM
O2403	Pre-existing type 1 diabetes mellitus, in the puerperium	Diagnosis	ICD-10-CM
O2483	Other pre-existing diabetes mellitus in the puerperium	Diagnosis	ICD-10-CM
O2433	Unspecified pre-existing diabetes mellitus in the puerperium	Diagnosis	ICD-10-CM
O2413	Pre-existing type 2 diabetes mellitus, in the puerperium	Diagnosis	ICD-10-CM
O24.414	Gestational diabetes mellitus in pregnancy, insulin controlled	Diagnosis	ICD-10-CM
O24.415	Gestational diabetes mellitus in pregnancy, controlled by oral hypoglycemic drugs	Diagnosis	ICD-10-CM
O24.419	Gestational diabetes mellitus in pregnancy, unspecified control	Diagnosis	ICD-10-CM
O24.425	Gestational diabetes mellitus in childbirth, controlled by oral hypoglycemic drugs	Diagnosis	ICD-10-CM
O24.429	Gestational diabetes mellitus in childbirth, unspecified control	Diagnosis	ICD-10-CM
O24.434	Gestational diabetes mellitus in the puerperium, insulin controlled	Diagnosis	ICD-10-CM
O24.435	Gestational diabetes mellitus in puerperium, controlled by oral hypoglycemic drugs	Diagnosis	ICD-10-CM

Code	Description	Code Category	Code Type
O24.439	Gestational diabetes mellitus in the puerperium, unspecified control	Diagnosis	ICD-10-CM
E08.610	Diabetes mellitus due to underlying condition with diabetic neuropathic arthropathy	Diagnosis	ICD-10-CM
E08.618	Diabetes mellitus due to underlying condition with other diabetic arthropathy	Diagnosis	ICD-10-CM
E09.610	Drug or chemical induced diabetes mellitus with diabetic neuropathic arthropathy	Diagnosis	ICD-10-CM
E09.618	Drug or chemical induced diabetes mellitus with other diabetic arthropathy	Diagnosis	ICD-10-CM
E10.610	Type 1 diabetes mellitus with diabetic neuropathic arthropathy	Diagnosis	ICD-10-CM
E10.618	Type 1 diabetes mellitus with other diabetic arthropathy	Diagnosis	ICD-10-CM
E11.610	Type 2 diabetes mellitus with diabetic neuropathic arthropathy	Diagnosis	ICD-10-CM
E11.618	Type 2 diabetes mellitus with other diabetic arthropathy	Diagnosis	ICD-10-CM
E13.610	Other specified diabetes mellitus with diabetic neuropathic arthropathy	Diagnosis	ICD-10-CM
E13.618	Other specified diabetes mellitus with other diabetic arthropathy	Diagnosis	ICD-10-CM
249	Secondary diabetes mellitus	Diagnosis	ICD-9-CM
249.0	Secondary diabetes mellitus without mention of complication	Diagnosis	ICD-9-CM
249.00	Secondary diabetes mellitus without mention of complication, not stated as uncontrolled, or unspecified	Diagnosis	ICD-9-CM
249.01	Secondary diabetes mellitus without mention of complication, uncontrolled	Diagnosis	ICD-9-CM
249.1	Secondary diabetes mellitus with ketoacidosis	Diagnosis	ICD-9-CM
249.10	Secondary diabetes mellitus with ketoacidosis, not stated as uncontrolled, or unspecified	Diagnosis	ICD-9-CM
249.11	Secondary diabetes mellitus with ketoacidosis, uncontrolled	Diagnosis	ICD-9-CM
249.2	Secondary diabetes mellitus with hyperosmolarity	Diagnosis	ICD-9-CM
249.20	Secondary diabetes mellitus with hyperosmolarity, not stated as uncontrolled, or unspecified	Diagnosis	ICD-9-CM
249.21	Secondary diabetes mellitus with hyperosmolarity, uncontrolled	Diagnosis	ICD-9-CM
249.3	Secondary diabetes mellitus with other coma	Diagnosis	ICD-9-CM
249.30	Secondary diabetes mellitus with other coma, not stated as uncontrolled, or unspecified	Diagnosis	ICD-9-CM
249.31	Secondary diabetes mellitus with other coma, uncontrolled	Diagnosis	ICD-9-CM
249.4	Secondary diabetes mellitus with renal manifestations	Diagnosis	ICD-9-CM
249.40	Secondary diabetes mellitus with renal manifestations, not stated as uncontrolled, or unspecified	Diagnosis	ICD-9-CM
249.41	Secondary diabetes mellitus with renal manifestations, uncontrolled	Diagnosis	ICD-9-CM
249.5	Secondary diabetes mellitus with ophthalmic manifestations	Diagnosis	ICD-9-CM
249.50	Secondary diabetes mellitus with ophthalmic manifestations, not stated as uncontrolled, or unspecified	Diagnosis	ICD-9-CM
249.51	Secondary diabetes mellitus with ophthalmic manifestations, uncontrolled	Diagnosis	ICD-9-CM
249.6	Secondary diabetes mellitus with neurological manifestations	Diagnosis	ICD-9-CM
249.60	Secondary diabetes mellitus with neurological manifestations, not stated as uncontrolled, or unspecified	Diagnosis	ICD-9-CM
249.61	Secondary diabetes mellitus with neurological manifestations, uncontrolled	Diagnosis	ICD-9-CM
249.7	Secondary diabetes mellitus with peripheral circulatory disorders	Diagnosis	ICD-9-CM
249.70	Secondary diabetes mellitus with peripheral circulatory disorders, not stated as uncontrolled, or unspecified	Diagnosis	ICD-9-CM
249.71	Secondary diabetes mellitus with peripheral circulatory disorders, uncontrolled	Diagnosis	ICD-9-CM
249.8	Secondary diabetes mellitus with other specified manifestations	Diagnosis	ICD-9-CM

Code	Description	Code Category	Code Type
249.80	Secondary diabetes mellitus with other specified manifestations, not stated as uncontrolled, or unspecified	Diagnosis	ICD-9-CM
249.81	Secondary diabetes mellitus with other specified manifestations, uncontrolled	Diagnosis	ICD-9-CM
249.9	Secondary diabetes mellitus with unspecified complication	Diagnosis	ICD-9-CM
249.90	Secondary diabetes mellitus with unspecified complication, not stated as uncontrolled, or unspecified	Diagnosis	ICD-9-CM
249.91	Secondary diabetes mellitus with unspecified complication, uncontrolled	Diagnosis	ICD-9-CM
251.1	Other specified hypoglycemia	Diagnosis	ICD-9-CM
E08.00	Diabetes mellitus due to underlying condition with hyperosmolarity without nonketotic hyperglycemic-hyperosmolar coma (NKHHC)	Diagnosis	ICD-10-CM
E08.01	Diabetes mellitus due to underlying condition with hyperosmolarity with coma	Diagnosis	ICD-10-CM
E08.10	Diabetes mellitus due to underlying condition with ketoacidosis without coma	Diagnosis	ICD-10-CM
E08.11	Diabetes mellitus due to underlying condition with ketoacidosis with coma	Diagnosis	ICD-10-CM
E08.311	Diabetes mellitus due to underlying condition with unspecified diabetic retinopathy with macular edema	Diagnosis	ICD-10-CM
E08.319	Diabetes mellitus due to underlying condition with unspecified diabetic retinopathy without macular edema	Diagnosis	ICD-10-CM
E08.3211	Diabetes mellitus due to underlying condition with mild nonproliferative diabetic retinopathy with macular edema, right eye	Diagnosis	ICD-10-CM
E08.3212	Diabetes mellitus due to underlying condition with mild nonproliferative diabetic retinopathy with macular edema, left eye	Diagnosis	ICD-10-CM
E08.3213	Diabetes mellitus due to underlying condition with mild nonproliferative diabetic retinopathy with macular edema, bilateral	Diagnosis	ICD-10-CM
E08.3219	Diabetes mellitus due to underlying condition with mild nonproliferative diabetic retinopathy with macular edema, unspecified eye	Diagnosis	ICD-10-CM
E08.3291	Diabetes mellitus due to underlying condition with mild nonproliferative diabetic retinopathy without macular edema, right eye	Diagnosis	ICD-10-CM
E08.3292	Diabetes mellitus due to underlying condition with mild nonproliferative diabetic retinopathy without macular edema, left eye	Diagnosis	ICD-10-CM
E08.3293	Diabetes mellitus due to underlying condition with mild nonproliferative diabetic retinopathy without macular edema, bilateral	Diagnosis	ICD-10-CM
E08.3299	Diabetes mellitus due to underlying condition with mild nonproliferative diabetic retinopathy without macular edema, unspecified eye	Diagnosis	ICD-10-CM
E08.3311	Diabetes mellitus due to underlying condition with moderate nonproliferative diabetic retinopathy with macular edema, right eye	Diagnosis	ICD-10-CM
E08.3312	Diabetes mellitus due to underlying condition with moderate nonproliferative diabetic retinopathy with macular edema, left eye	Diagnosis	ICD-10-CM
E08.3313	Diabetes mellitus due to underlying condition with moderate nonproliferative diabetic retinopathy with macular edema, bilateral	Diagnosis	ICD-10-CM
E08.3319	Diabetes mellitus due to underlying condition with moderate nonproliferative diabetic retinopathy with macular edema, unspecified eye	Diagnosis	ICD-10-CM
E08.3391	Diabetes mellitus due to underlying condition with moderate nonproliferative diabetic retinopathy without macular edema, right eye	Diagnosis	ICD-10-CM
E08.3392	Diabetes mellitus due to underlying condition with moderate nonproliferative diabetic retinopathy without macular edema, left eye	Diagnosis	ICD-10-CM

Code	Description	Code Category	Code Type
E08.3393	Diabetes mellitus due to underlying condition with moderate nonproliferative diabetic retinopathy without macular edema, bilateral	Diagnosis	ICD-10-CM
E08.3399	Diabetes mellitus due to underlying condition with moderate nonproliferative diabetic retinopathy without macular edema, unspecified eye	Diagnosis	ICD-10-CM
E08.3411	Diabetes mellitus due to underlying condition with severe nonproliferative diabetic retinopathy with macular edema, right eye	Diagnosis	ICD-10-CM
E08.3412	Diabetes mellitus due to underlying condition with severe nonproliferative diabetic retinopathy with macular edema, left eye	Diagnosis	ICD-10-CM
E08.3413	Diabetes mellitus due to underlying condition with severe nonproliferative diabetic retinopathy with macular edema, bilateral	Diagnosis	ICD-10-CM
E08.3419	Diabetes mellitus due to underlying condition with severe nonproliferative diabetic retinopathy with macular edema, unspecified eye	Diagnosis	ICD-10-CM
E08.3491	Diabetes mellitus due to underlying condition with severe nonproliferative diabetic retinopathy without macular edema, right eye	Diagnosis	ICD-10-CM
E08.3492	Diabetes mellitus due to underlying condition with severe nonproliferative diabetic retinopathy without macular edema, left eye	Diagnosis	ICD-10-CM
E08.3493	Diabetes mellitus due to underlying condition with severe nonproliferative diabetic retinopathy without macular edema, bilateral	Diagnosis	ICD-10-CM
E08.3499	Diabetes mellitus due to underlying condition with severe nonproliferative diabetic retinopathy without macular edema, unspecified eye	Diagnosis	ICD-10-CM
E08.3511	Diabetes mellitus due to underlying condition with proliferative diabetic retinopathy with macular edema, right eye	Diagnosis	ICD-10-CM
E08.3512	Diabetes mellitus due to underlying condition with proliferative diabetic retinopathy with macular edema, left eye	Diagnosis	ICD-10-CM
E08.3513	Diabetes mellitus due to underlying condition with proliferative diabetic retinopathy with macular edema, bilateral	Diagnosis	ICD-10-CM
E08.3519	Diabetes mellitus due to underlying condition with proliferative diabetic retinopathy with macular edema, unspecified eye	Diagnosis	ICD-10-CM
E08.3521	Diabetes mellitus due to underlying condition with proliferative diabetic retinopathy with traction retinal detachment involving the macula, right eye	Diagnosis	ICD-10-CM
E08.3522	Diabetes mellitus due to underlying condition with proliferative diabetic retinopathy with traction retinal detachment involving the macula, left eye	Diagnosis	ICD-10-CM
E08.3523	Diabetes mellitus due to underlying condition with proliferative diabetic retinopathy with traction retinal detachment involving the macula, bilateral	Diagnosis	ICD-10-CM
E08.3529	Diabetes mellitus due to underlying condition with proliferative diabetic retinopathy with traction retinal detachment involving the macula, unspecified eye	Diagnosis	ICD-10-CM
E08.3531	Diabetes mellitus due to underlying condition with proliferative diabetic retinopathy with traction retinal detachment not involving the macula, right eye	Diagnosis	ICD-10-CM
E08.3532	Diabetes mellitus due to underlying condition with proliferative diabetic retinopathy with traction retinal detachment not involving the macula, left eye	Diagnosis	ICD-10-CM
E08.3533	Diabetes mellitus due to underlying condition with proliferative diabetic retinopathy with traction retinal detachment not involving the macula, bilateral	Diagnosis	ICD-10-CM

Code	Description	Code Category	Code Type
E08.3539	Diabetes mellitus due to underlying condition with proliferative diabetic retinopathy with traction retinal detachment not involving the macula, unspecified eye	Diagnosis	ICD-10-CM
E08.3541	Diabetes mellitus due to underlying condition with proliferative diabetic retinopathy with combined traction retinal detachment and rhegmatogenous retinal detachment, right eye	Diagnosis	ICD-10-CM
E08.3542	Diabetes mellitus due to underlying condition with proliferative diabetic retinopathy with combined traction retinal detachment and rhegmatogenous retinal detachment, left eye	Diagnosis	ICD-10-CM
E08.3543	Diabetes mellitus due to underlying condition with proliferative diabetic retinopathy with combined traction retinal detachment and rhegmatogenous retinal detachment, bilateral	Diagnosis	ICD-10-CM
E08.3549	Diabetes mellitus due to underlying condition with proliferative diabetic retinopathy with combined traction retinal detachment and rhegmatogenous retinal detachment, unspecified eye	Diagnosis	ICD-10-CM
E08.3551	Diabetes mellitus due to underlying condition with stable proliferative diabetic retinopathy, right eye	Diagnosis	ICD-10-CM
E08.3552	Diabetes mellitus due to underlying condition with stable proliferative diabetic retinopathy, left eye	Diagnosis	ICD-10-CM
E08.3553	Diabetes mellitus due to underlying condition with stable proliferative diabetic retinopathy, bilateral	Diagnosis	ICD-10-CM
E08.3559	Diabetes mellitus due to underlying condition with stable proliferative diabetic retinopathy, unspecified eye	Diagnosis	ICD-10-CM
E08.3591	Diabetes mellitus due to underlying condition with proliferative diabetic retinopathy without macular edema, right eye	Diagnosis	ICD-10-CM
E08.3592	Diabetes mellitus due to underlying condition with proliferative diabetic retinopathy without macular edema, left eye	Diagnosis	ICD-10-CM
E08.3593	Diabetes mellitus due to underlying condition with proliferative diabetic retinopathy without macular edema, bilateral	Diagnosis	ICD-10-CM
E08.3599	Diabetes mellitus due to underlying condition with proliferative diabetic retinopathy without macular edema, unspecified eye	Diagnosis	ICD-10-CM
E08.36	Diabetes mellitus due to underlying condition with diabetic cataract	Diagnosis	ICD-10-CM
E08.37X1	Diabetes mellitus due to underlying condition with diabetic macular edema, resolved following treatment, right eye	Diagnosis	ICD-10-CM
E08.37X2	Diabetes mellitus due to underlying condition with diabetic macular edema, resolved following treatment, left eye	Diagnosis	ICD-10-CM
E08.37X3	Diabetes mellitus due to underlying condition with diabetic macular edema, resolved following treatment, bilateral	Diagnosis	ICD-10-CM
E08.37X9	Diabetes mellitus due to underlying condition with diabetic macular edema, resolved following treatment, unspecified eye	Diagnosis	ICD-10-CM
E08.39	Diabetes mellitus due to underlying condition with other diabetic ophthalmic complication	Diagnosis	ICD-10-CM
E08.40	Diabetes mellitus due to underlying condition with diabetic neuropathy, unspecified	Diagnosis	ICD-10-CM
E08.41	Diabetes mellitus due to underlying condition with diabetic mononeuropathy	Diagnosis	ICD-10-CM

Code	Description	Code Category	Code Type
E08.42	Diabetes mellitus due to underlying condition with diabetic polyneuropathy	Diagnosis	ICD-10-CM
E08.43	Diabetes mellitus due to underlying condition with diabetic autonomic (poly)neuropathy	Diagnosis	ICD-10-CM
E08.44	Diabetes mellitus due to underlying condition with diabetic amyotrophy	Diagnosis	ICD-10-CM
E08.49	Diabetes mellitus due to underlying condition with other diabetic neurological complication	Diagnosis	ICD-10-CM
E08.51	Diabetes mellitus due to underlying condition with diabetic peripheral angiopathy without gangrene	Diagnosis	ICD-10-CM
E08.52	Diabetes mellitus due to underlying condition with diabetic peripheral angiopathy with gangrene	Diagnosis	ICD-10-CM
E08.59	Diabetes mellitus due to underlying condition with other circulatory complications	Diagnosis	ICD-10-CM
E08.610	Diabetes mellitus due to underlying condition with diabetic neuropathic arthropathy	Diagnosis	ICD-10-CM
E08.618	Diabetes mellitus due to underlying condition with other diabetic arthropathy	Diagnosis	ICD-10-CM
E08.620	Diabetes mellitus due to underlying condition with diabetic dermatitis	Diagnosis	ICD-10-CM
E08.621	Diabetes mellitus due to underlying condition with foot ulcer	Diagnosis	ICD-10-CM
E08.622	Diabetes mellitus due to underlying condition with other skin ulcer	Diagnosis	ICD-10-CM
E08.628	Diabetes mellitus due to underlying condition with other skin complications	Diagnosis	ICD-10-CM
E08.630	Diabetes mellitus due to underlying condition with periodontal disease	Diagnosis	ICD-10-CM
E08.638	Diabetes mellitus due to underlying condition with other oral complications	Diagnosis	ICD-10-CM
E08.641	Diabetes mellitus due to underlying condition with hypoglycemia with coma	Diagnosis	ICD-10-CM
E08.649	Diabetes mellitus due to underlying condition with hypoglycemia without coma	Diagnosis	ICD-10-CM
E08.65	Diabetes mellitus due to underlying condition with hyperglycemia	Diagnosis	ICD-10-CM
E08.69	Diabetes mellitus due to underlying condition with other specified complication	Diagnosis	ICD-10-CM
E08.8	Diabetes mellitus due to underlying condition with unspecified complications	Diagnosis	ICD-10-CM
E08.9	Diabetes mellitus due to underlying condition without complications	Diagnosis	ICD-10-CM
E09.00	Drug or chemical induced diabetes mellitus with hyperosmolarity without nonketotic hyperglycemic-hyperosmolar coma (NKHHC)	Diagnosis	ICD-10-CM
E09.01	Drug or chemical induced diabetes mellitus with hyperosmolarity with coma	Diagnosis	ICD-10-CM
E09.10	Drug or chemical induced diabetes mellitus with ketoacidosis without coma	Diagnosis	ICD-10-CM
E09.11	Drug or chemical induced diabetes mellitus with ketoacidosis with coma	Diagnosis	ICD-10-CM
E09.21	Drug or chemical induced diabetes mellitus with diabetic nephropathy	Diagnosis	ICD-10-CM
E09.22	Drug or chemical induced diabetes mellitus with diabetic chronic kidney disease	Diagnosis	ICD-10-CM
E09.29	Drug or chemical induced diabetes mellitus with other diabetic kidney complication	Diagnosis	ICD-10-CM
E09.311	Drug or chemical induced diabetes mellitus with unspecified diabetic retinopathy with macular edema	Diagnosis	ICD-10-CM
E09.319	Drug or chemical induced diabetes mellitus with unspecified diabetic retinopathy without macular edema	Diagnosis	ICD-10-CM
E09.3211	Drug or chemical induced diabetes mellitus with mild nonproliferative diabetic retinopathy with macular edema, right eye	Diagnosis	ICD-10-CM
E09.3212	Drug or chemical induced diabetes mellitus with mild nonproliferative diabetic retinopathy with macular edema, left eye	Diagnosis	ICD-10-CM
E09.3213	Drug or chemical induced diabetes mellitus with mild nonproliferative diabetic retinopathy with macular edema, bilateral	Diagnosis	ICD-10-CM

Code	Description	Code Category	Code Type
E09.3219	Drug or chemical induced diabetes mellitus with mild nonproliferative diabetic retinopathy with macular edema, unspecified eye	Diagnosis	ICD-10-CM
E09.3291	Drug or chemical induced diabetes mellitus with mild nonproliferative diabetic retinopathy without macular edema, right eye	Diagnosis	ICD-10-CM
E09.3292	Drug or chemical induced diabetes mellitus with mild nonproliferative diabetic retinopathy without macular edema, left eye	Diagnosis	ICD-10-CM
E09.3293	Drug or chemical induced diabetes mellitus with mild nonproliferative diabetic retinopathy without macular edema, bilateral	Diagnosis	ICD-10-CM
E09.3299	Drug or chemical induced diabetes mellitus with mild nonproliferative diabetic retinopathy without macular edema, unspecified eye	Diagnosis	ICD-10-CM
E09.3311	Drug or chemical induced diabetes mellitus with moderate nonproliferative diabetic retinopathy with macular edema, right eye	Diagnosis	ICD-10-CM
E09.3312	Drug or chemical induced diabetes mellitus with moderate nonproliferative diabetic retinopathy with macular edema, left eye	Diagnosis	ICD-10-CM
E09.3313	Drug or chemical induced diabetes mellitus with moderate nonproliferative diabetic retinopathy with macular edema, bilateral	Diagnosis	ICD-10-CM
E09.3319	Drug or chemical induced diabetes mellitus with moderate nonproliferative diabetic retinopathy with macular edema, unspecified eye	Diagnosis	ICD-10-CM
E09.3391	Drug or chemical induced diabetes mellitus with moderate nonproliferative diabetic retinopathy without macular edema, right eye	Diagnosis	ICD-10-CM
E09.3392	Drug or chemical induced diabetes mellitus with moderate nonproliferative diabetic retinopathy without macular edema, left eye	Diagnosis	ICD-10-CM
E09.3393	Drug or chemical induced diabetes mellitus with moderate nonproliferative diabetic retinopathy without macular edema, bilateral	Diagnosis	ICD-10-CM
E09.3399	Drug or chemical induced diabetes mellitus with moderate nonproliferative diabetic retinopathy without macular edema, unspecified eye	Diagnosis	ICD-10-CM
E09.3411	Drug or chemical induced diabetes mellitus with severe nonproliferative diabetic retinopathy with macular edema, right eye	Diagnosis	ICD-10-CM
E09.3412	Drug or chemical induced diabetes mellitus with severe nonproliferative diabetic retinopathy with macular edema, left eye	Diagnosis	ICD-10-CM
E09.3413	Drug or chemical induced diabetes mellitus with severe nonproliferative diabetic retinopathy with macular edema, bilateral	Diagnosis	ICD-10-CM
E09.3419	Drug or chemical induced diabetes mellitus with severe nonproliferative diabetic retinopathy with macular edema, unspecified eye	Diagnosis	ICD-10-CM
E09.3491	Drug or chemical induced diabetes mellitus with severe nonproliferative diabetic retinopathy without macular edema, right eye	Diagnosis	ICD-10-CM
E09.3492	Drug or chemical induced diabetes mellitus with severe nonproliferative diabetic retinopathy without macular edema, left eye	Diagnosis	ICD-10-CM
E09.3493	Drug or chemical induced diabetes mellitus with severe nonproliferative diabetic retinopathy without macular edema, bilateral	Diagnosis	ICD-10-CM
E09.3499	Drug or chemical induced diabetes mellitus with severe nonproliferative diabetic retinopathy without macular edema, unspecified eye	Diagnosis	ICD-10-CM
E09.3511	Drug or chemical induced diabetes mellitus with proliferative diabetic retinopathy with macular edema, right eye	Diagnosis	ICD-10-CM

Code	Description	Code Category	Code Type
E09.3512	Drug or chemical induced diabetes mellitus with proliferative diabetic retinopathy with macular edema, left eye	Diagnosis	ICD-10-CM
E09.3513	Drug or chemical induced diabetes mellitus with proliferative diabetic retinopathy with macular edema, bilateral	Diagnosis	ICD-10-CM
E09.3519	Drug or chemical induced diabetes mellitus with proliferative diabetic retinopathy with macular edema, unspecified eye	Diagnosis	ICD-10-CM
E09.3521	Drug or chemical induced diabetes mellitus with proliferative diabetic retinopathy with traction retinal detachment involving the macula, right eye	Diagnosis	ICD-10-CM
E09.3522	Drug or chemical induced diabetes mellitus with proliferative diabetic retinopathy with traction retinal detachment involving the macula, left eye	Diagnosis	ICD-10-CM
E09.3523	Drug or chemical induced diabetes mellitus with proliferative diabetic retinopathy with traction retinal detachment involving the macula, bilateral	Diagnosis	ICD-10-CM
E09.3529	Drug or chemical induced diabetes mellitus with proliferative diabetic retinopathy with traction retinal detachment involving the macula, unspecified eye	Diagnosis	ICD-10-CM
E09.3531	Drug or chemical induced diabetes mellitus with proliferative diabetic retinopathy with traction retinal detachment not involving the macula, right eye	Diagnosis	ICD-10-CM
E09.3532	Drug or chemical induced diabetes mellitus with proliferative diabetic retinopathy with traction retinal detachment not involving the macula, left eye	Diagnosis	ICD-10-CM
E09.3533	Drug or chemical induced diabetes mellitus with proliferative diabetic retinopathy with traction retinal detachment not involving the macula, bilateral	Diagnosis	ICD-10-CM
E09.3539	Drug or chemical induced diabetes mellitus with proliferative diabetic retinopathy with traction retinal detachment not involving the macula, unspecified eye	Diagnosis	ICD-10-CM
E09.3541	Drug or chemical induced diabetes mellitus with proliferative diabetic retinopathy with combined traction retinal detachment and rhegmatogenous retinal detachment, right eye	Diagnosis	ICD-10-CM
E09.3542	Drug or chemical induced diabetes mellitus with proliferative diabetic retinopathy with combined traction retinal detachment and rhegmatogenous retinal detachment, left eye	Diagnosis	ICD-10-CM
E09.3543	Drug or chemical induced diabetes mellitus with proliferative diabetic retinopathy with combined traction retinal detachment and rhegmatogenous retinal detachment, bilateral	Diagnosis	ICD-10-CM
E09.3549	Drug or chemical induced diabetes mellitus with proliferative diabetic retinopathy with combined traction retinal detachment and rhegmatogenous retinal detachment, unspecified eye	Diagnosis	ICD-10-CM
E09.3551	Drug or chemical induced diabetes mellitus with stable proliferative diabetic retinopathy, right eye	Diagnosis	ICD-10-CM
E09.3552	Drug or chemical induced diabetes mellitus with stable proliferative diabetic retinopathy, left eye	Diagnosis	ICD-10-CM
E09.3553	Drug or chemical induced diabetes mellitus with stable proliferative diabetic retinopathy, bilateral	Diagnosis	ICD-10-CM
E09.3559	Drug or chemical induced diabetes mellitus with stable proliferative diabetic retinopathy, unspecified eye	Diagnosis	ICD-10-CM
E09.3591	Drug or chemical induced diabetes mellitus with proliferative diabetic retinopathy without macular edema, right eye	Diagnosis	ICD-10-CM

Code	Description	Code Category	Code Type
E09.3592	Drug or chemical induced diabetes mellitus with proliferative diabetic retinopathy without macular edema, left eye	Diagnosis	ICD-10-CM
E09.3593	Drug or chemical induced diabetes mellitus with proliferative diabetic retinopathy without macular edema, bilateral	Diagnosis	ICD-10-CM
E09.3599	Drug or chemical induced diabetes mellitus with proliferative diabetic retinopathy without macular edema, unspecified eye	Diagnosis	ICD-10-CM
E09.36	Drug or chemical induced diabetes mellitus with diabetic cataract	Diagnosis	ICD-10-CM
E09.37X1	Drug or chemical induced diabetes mellitus with diabetic macular edema, resolved following treatment, right eye	Diagnosis	ICD-10-CM
E09.37X2	Drug or chemical induced diabetes mellitus with diabetic macular edema, resolved following treatment, left eye	Diagnosis	ICD-10-CM
E09.37X3	Drug or chemical induced diabetes mellitus with diabetic macular edema, resolved following treatment, bilateral	Diagnosis	ICD-10-CM
E09.37X9	Drug or chemical induced diabetes mellitus with diabetic macular edema, resolved following treatment, unspecified eye	Diagnosis	ICD-10-CM
E09.39	Drug or chemical induced diabetes mellitus with other diabetic ophthalmic complication	Diagnosis	ICD-10-CM
E09.40	Drug or chemical induced diabetes mellitus with neurological complications with diabetic neuropathy, unspecified	Diagnosis	ICD-10-CM
E09.41	Drug or chemical induced diabetes mellitus with neurological complications with diabetic mononeuropathy	Diagnosis	ICD-10-CM
E09.42	Drug or chemical induced diabetes mellitus with neurological complications with diabetic polyneuropathy	Diagnosis	ICD-10-CM
E09.43	Drug or chemical induced diabetes mellitus with neurological complications with diabetic autonomic (poly)neuropathy	Diagnosis	ICD-10-CM
E09.44	Drug or chemical induced diabetes mellitus with neurological complications with diabetic amyotrophy	Diagnosis	ICD-10-CM
E09.49	Drug or chemical induced diabetes mellitus with neurological complications with other diabetic neurological complication	Diagnosis	ICD-10-CM
E09.51	Drug or chemical induced diabetes mellitus with diabetic peripheral angiopathy without gangrene	Diagnosis	ICD-10-CM
E09.52	Drug or chemical induced diabetes mellitus with diabetic peripheral angiopathy with gangrene	Diagnosis	ICD-10-CM
E09.59	Drug or chemical induced diabetes mellitus with other circulatory complications	Diagnosis	ICD-10-CM
E09.610	Drug or chemical induced diabetes mellitus with diabetic neuropathic arthropathy	Diagnosis	ICD-10-CM
E09.618	Drug or chemical induced diabetes mellitus with other diabetic arthropathy	Diagnosis	ICD-10-CM
E09.620	Drug or chemical induced diabetes mellitus with diabetic dermatitis	Diagnosis	ICD-10-CM
E09.621	Drug or chemical induced diabetes mellitus with foot ulcer	Diagnosis	ICD-10-CM
E09.622	Drug or chemical induced diabetes mellitus with other skin ulcer	Diagnosis	ICD-10-CM
E09.628	Drug or chemical induced diabetes mellitus with other skin complications	Diagnosis	ICD-10-CM
E09.630	Drug or chemical induced diabetes mellitus with periodontal disease	Diagnosis	ICD-10-CM
E09.638	Drug or chemical induced diabetes mellitus with other oral complications	Diagnosis	ICD-10-CM
E09.641	Drug or chemical induced diabetes mellitus with hypoglycemia with coma	Diagnosis	ICD-10-CM
E09.649	Drug or chemical induced diabetes mellitus with hypoglycemia without coma	Diagnosis	ICD-10-CM
E09.65	Drug or chemical induced diabetes mellitus with hyperglycemia	Diagnosis	ICD-10-CM

Code	Description	Code Category	Code Type
E09.69	Drug or chemical induced diabetes mellitus with other specified complication	Diagnosis	ICD-10-CM
E09.8	Drug or chemical induced diabetes mellitus with unspecified complications	Diagnosis	ICD-10-CM
E09.9	Drug or chemical induced diabetes mellitus without complications	Diagnosis	ICD-10-CM
E13.00	Other specified diabetes mellitus with hyperosmolarity without nonketotic hyperglycemic-hyperosmolar coma (NKHHC)	Diagnosis	ICD-10-CM
E13.01	Other specified diabetes mellitus with hyperosmolarity with coma	Diagnosis	ICD-10-CM
E13.10	Other specified diabetes mellitus with ketoacidosis without coma	Diagnosis	ICD-10-CM
E13.11	Other specified diabetes mellitus with ketoacidosis with coma	Diagnosis	ICD-10-CM
E13.311	Other specified diabetes mellitus with unspecified diabetic retinopathy with macular edema	Diagnosis	ICD-10-CM
E13.319	Other specified diabetes mellitus with unspecified diabetic retinopathy without macular edema	Diagnosis	ICD-10-CM
E13.3211	Other specified diabetes mellitus with mild nonproliferative diabetic retinopathy with macular edema, right eye	Diagnosis	ICD-10-CM
E13.3212	Other specified diabetes mellitus with mild nonproliferative diabetic retinopathy with macular edema, left eye	Diagnosis	ICD-10-CM
E13.3213	Other specified diabetes mellitus with mild nonproliferative diabetic retinopathy with macular edema, bilateral	Diagnosis	ICD-10-CM
E13.3219	Other specified diabetes mellitus with mild nonproliferative diabetic retinopathy with macular edema, unspecified eye	Diagnosis	ICD-10-CM
E13.3291	Other specified diabetes mellitus with mild nonproliferative diabetic retinopathy without macular edema, right eye	Diagnosis	ICD-10-CM
E13.3292	Other specified diabetes mellitus with mild nonproliferative diabetic retinopathy without macular edema, left eye	Diagnosis	ICD-10-CM
E13.3293	Other specified diabetes mellitus with mild nonproliferative diabetic retinopathy without macular edema, bilateral	Diagnosis	ICD-10-CM
E13.3299	Other specified diabetes mellitus with mild nonproliferative diabetic retinopathy without macular edema, unspecified eye	Diagnosis	ICD-10-CM
E13.3311	Other specified diabetes mellitus with moderate nonproliferative diabetic retinopathy with macular edema, right eye	Diagnosis	ICD-10-CM
E13.3312	Other specified diabetes mellitus with moderate nonproliferative diabetic retinopathy with macular edema, left eye	Diagnosis	ICD-10-CM
E13.3313	Other specified diabetes mellitus with moderate nonproliferative diabetic retinopathy with macular edema, bilateral	Diagnosis	ICD-10-CM
E13.3319	Other specified diabetes mellitus with moderate nonproliferative diabetic retinopathy with macular edema, unspecified eye	Diagnosis	ICD-10-CM
E13.3391	Other specified diabetes mellitus with moderate nonproliferative diabetic retinopathy without macular edema, right eye	Diagnosis	ICD-10-CM
E13.3392	Other specified diabetes mellitus with moderate nonproliferative diabetic retinopathy without macular edema, left eye	Diagnosis	ICD-10-CM
E13.3393	Other specified diabetes mellitus with moderate nonproliferative diabetic retinopathy without macular edema, bilateral	Diagnosis	ICD-10-CM
E13.3399	Other specified diabetes mellitus with moderate nonproliferative diabetic retinopathy without macular edema, unspecified eye	Diagnosis	ICD-10-CM

Code	Description	Code Category	Code Type
E13.3411	Other specified diabetes mellitus with severe nonproliferative diabetic retinopathy with macular edema, right eye	Diagnosis	ICD-10-CM
E13.3412	Other specified diabetes mellitus with severe nonproliferative diabetic retinopathy with macular edema, left eye	Diagnosis	ICD-10-CM
E13.3413	Other specified diabetes mellitus with severe nonproliferative diabetic retinopathy with macular edema, bilateral	Diagnosis	ICD-10-CM
E13.3419	Other specified diabetes mellitus with severe nonproliferative diabetic retinopathy with macular edema, unspecified eye	Diagnosis	ICD-10-CM
E13.3491	Other specified diabetes mellitus with severe nonproliferative diabetic retinopathy without macular edema, right eye	Diagnosis	ICD-10-CM
E13.3492	Other specified diabetes mellitus with severe nonproliferative diabetic retinopathy without macular edema, left eye	Diagnosis	ICD-10-CM
E13.3493	Other specified diabetes mellitus with severe nonproliferative diabetic retinopathy without macular edema, bilateral	Diagnosis	ICD-10-CM
E13.3499	Other specified diabetes mellitus with severe nonproliferative diabetic retinopathy without macular edema, unspecified eye	Diagnosis	ICD-10-CM
E13.3511	Other specified diabetes mellitus with proliferative diabetic retinopathy with macular edema, right eye	Diagnosis	ICD-10-CM
E13.3512	Other specified diabetes mellitus with proliferative diabetic retinopathy with macular edema, left eye	Diagnosis	ICD-10-CM
E13.3513	Other specified diabetes mellitus with proliferative diabetic retinopathy with macular edema, bilateral	Diagnosis	ICD-10-CM
E13.3519	Other specified diabetes mellitus with proliferative diabetic retinopathy with macular edema, unspecified eye	Diagnosis	ICD-10-CM
E13.3521	Other specified diabetes mellitus with proliferative diabetic retinopathy with traction retinal detachment involving the macula, right eye	Diagnosis	ICD-10-CM
E13.3522	Other specified diabetes mellitus with proliferative diabetic retinopathy with traction retinal detachment involving the macula, left eye	Diagnosis	ICD-10-CM
E13.3523	Other specified diabetes mellitus with proliferative diabetic retinopathy with traction retinal detachment involving the macula, bilateral	Diagnosis	ICD-10-CM
E13.3529	Other specified diabetes mellitus with proliferative diabetic retinopathy with traction retinal detachment involving the macula, unspecified eye	Diagnosis	ICD-10-CM
E13.3531	Other specified diabetes mellitus with proliferative diabetic retinopathy with traction retinal detachment not involving the macula, right eye	Diagnosis	ICD-10-CM
E13.3532	Other specified diabetes mellitus with proliferative diabetic retinopathy with traction retinal detachment not involving the macula, left eye	Diagnosis	ICD-10-CM
E13.3533	Other specified diabetes mellitus with proliferative diabetic retinopathy with traction retinal detachment not involving the macula, bilateral	Diagnosis	ICD-10-CM
E13.3539	Other specified diabetes mellitus with proliferative diabetic retinopathy with traction retinal detachment not involving the macula, unspecified eye	Diagnosis	ICD-10-CM
E13.3541	Other specified diabetes mellitus with proliferative diabetic retinopathy with combined traction retinal detachment and rhegmatogenous retinal detachment, right eye	Diagnosis	ICD-10-CM

Code	Description	Code Category	Code Type
E13.3542	Other specified diabetes mellitus with proliferative diabetic retinopathy with combined traction retinal detachment and rhegmatogenous retinal detachment, left eye	Diagnosis	ICD-10-CM
E13.3543	Other specified diabetes mellitus with proliferative diabetic retinopathy with combined traction retinal detachment and rhegmatogenous retinal detachment, bilateral	Diagnosis	ICD-10-CM
E13.3549	Other specified diabetes mellitus with proliferative diabetic retinopathy with combined traction retinal detachment and rhegmatogenous retinal detachment, unspecified eye	Diagnosis	ICD-10-CM
E13.3551	Other specified diabetes mellitus with stable proliferative diabetic retinopathy, right eye	Diagnosis	ICD-10-CM
E13.3552	Other specified diabetes mellitus with stable proliferative diabetic retinopathy, left eye	Diagnosis	ICD-10-CM
E13.3553	Other specified diabetes mellitus with stable proliferative diabetic retinopathy, bilateral	Diagnosis	ICD-10-CM
E13.3559	Other specified diabetes mellitus with stable proliferative diabetic retinopathy, unspecified eye	Diagnosis	ICD-10-CM
E13.3591	Other specified diabetes mellitus with proliferative diabetic retinopathy without macular edema, right eye	Diagnosis	ICD-10-CM
E13.3592	Other specified diabetes mellitus with proliferative diabetic retinopathy without macular edema, left eye	Diagnosis	ICD-10-CM
E13.3593	Other specified diabetes mellitus with proliferative diabetic retinopathy without macular edema, bilateral	Diagnosis	ICD-10-CM
E13.3599	Other specified diabetes mellitus with proliferative diabetic retinopathy without macular edema, unspecified eye	Diagnosis	ICD-10-CM
E13.36	Other specified diabetes mellitus with diabetic cataract	Diagnosis	ICD-10-CM
E13.37X1	Other specified diabetes mellitus with diabetic macular edema, resolved following treatment, right eye	Diagnosis	ICD-10-CM
E13.37X2	Other specified diabetes mellitus with diabetic macular edema, resolved following treatment, left eye	Diagnosis	ICD-10-CM
E13.37X3	Other specified diabetes mellitus with diabetic macular edema, resolved following treatment, bilateral	Diagnosis	ICD-10-CM
E13.37X9	Other specified diabetes mellitus with diabetic macular edema, resolved following treatment, unspecified eye	Diagnosis	ICD-10-CM
E13.39	Other specified diabetes mellitus with other diabetic ophthalmic complication	Diagnosis	ICD-10-CM
E13.618	Other specified diabetes mellitus with other diabetic arthropathy	Diagnosis	ICD-10-CM
E13.620	Other specified diabetes mellitus with diabetic dermatitis	Diagnosis	ICD-10-CM
E13.621	Other specified diabetes mellitus with foot ulcer	Diagnosis	ICD-10-CM
E13.622	Other specified diabetes mellitus with other skin ulcer	Diagnosis	ICD-10-CM
E13.628	Other specified diabetes mellitus with other skin complications	Diagnosis	ICD-10-CM
E13.630	Other specified diabetes mellitus with periodontal disease	Diagnosis	ICD-10-CM
E13.638	Other specified diabetes mellitus with other oral complications	Diagnosis	ICD-10-CM
E13.641	Other specified diabetes mellitus with hypoglycemia with coma	Diagnosis	ICD-10-CM
E13.649	Other specified diabetes mellitus with hypoglycemia without coma	Diagnosis	ICD-10-CM
E13.65	Other specified diabetes mellitus with hyperglycemia	Diagnosis	ICD-10-CM

Code	Description	Code Category	Code Type
E13.69	Other specified diabetes mellitus with other specified complication	Diagnosis	ICD-10-CM
E13.9	Other specified diabetes mellitus without complications	Diagnosis	ICD-10-CM
E08.21	Diabetes mellitus due to underlying condition with diabetic nephropathy	Diagnosis	ICD-10-CM
E08.22	Diabetes mellitus due to underlying condition with diabetic chronic kidney disease	Diagnosis	ICD-10-CM
E08.29	Diabetes mellitus due to underlying condition with other diabetic kidney complicati	Diagnosis	ICD-10-CM
	**Heart Failure**		
428	Heart failure	Diagnosis	ICD-9-CM
428.0	Congestive heart failure, unspecified	Diagnosis	ICD-9-CM
428.1	Left heart failure	Diagnosis	ICD-9-CM
428.2	Systolic heart failure	Diagnosis	ICD-9-CM
428.20	Unspecified systolic heart failure	Diagnosis	ICD-9-CM
428.21	Acute systolic heart failure	Diagnosis	ICD-9-CM
428.22	Chronic systolic heart failure	Diagnosis	ICD-9-CM
428.23	Acute on chronic systolic heart failure	Diagnosis	ICD-9-CM
428.3	Diastolic heart failure	Diagnosis	ICD-9-CM
428.30	Unspecified diastolic heart failure	Diagnosis	ICD-9-CM
428.31	Acute diastolic heart failure	Diagnosis	ICD-9-CM
428.32	Chronic diastolic heart failure	Diagnosis	ICD-9-CM
428.33	Acute on chronic diastolic heart failure	Diagnosis	ICD-9-CM
428.4	Combined systolic and diastolic heart failure	Diagnosis	ICD-9-CM
428.40	Unspecified combined systolic and diastolic heart failure	Diagnosis	ICD-9-CM
428.41	Acute combined systolic and diastolic heart failure	Diagnosis	ICD-9-CM
428.42	Chronic combined systolic and diastolic heart failure	Diagnosis	ICD-9-CM
428.43	Acute on chronic combined systolic and diastolic heart failure	Diagnosis	ICD-9-CM
428.9	Unspecified heart failure	Diagnosis	ICD-9-CM
I50.1	Left ventricular failure, unspecified	Diagnosis	ICD-10-CM
I50.20	Unspecified systolic (congestive) heart failure	Diagnosis	ICD-10-CM
I50.21	Acute systolic (congestive) heart failure	Diagnosis	ICD-10-CM
I50.22	Chronic systolic (congestive) heart failure	Diagnosis	ICD-10-CM
I50.23	Acute on chronic systolic (congestive) heart failure	Diagnosis	ICD-10-CM
I50.30	Unspecified diastolic (congestive) heart failure	Diagnosis	ICD-10-CM
I50.31	Acute diastolic (congestive) heart failure	Diagnosis	ICD-10-CM
I50.32	Chronic diastolic (congestive) heart failure	Diagnosis	ICD-10-CM
I50.33	Acute on chronic diastolic (congestive) heart failure	Diagnosis	ICD-10-CM
I50.40	Unspecified combined systolic (congestive) and diastolic (congestive) heart failure	Diagnosis	ICD-10-CM
I50.41	Acute combined systolic (congestive) and diastolic (congestive) heart failure	Diagnosis	ICD-10-CM
I50.42	Chronic combined systolic (congestive) and diastolic (congestive) heart failure	Diagnosis	ICD-10-CM
I50.43	Acute on chronic combined systolic (congestive) and diastolic (congestive) heart fail	Diagnosis	ICD-10-CM
I50.810	Right heart failure, unspecified	Diagnosis	ICD-10-CM
I50.811	Acute right heart failure	Diagnosis	ICD-10-CM
I50.812	Chronic right heart failure	Diagnosis	ICD-10-CM
I50.813	Acute on chronic right heart failure	Diagnosis	ICD-10-CM
I50.814	Right heart failure due to left heart failure	Diagnosis	ICD-10-CM
I50.82	Biventricular heart failure	Diagnosis	ICD-10-CM
I50.83	High output heart failure	Diagnosis	ICD-10-CM

Code	Description	Code Category	Code Type
I50.84	End stage heart failure	Diagnosis	ICD-10-CM
I50.89	Other heart failure	Diagnosis	ICD-10-CM
I50.9	Heart failure, unspecified	Diagnosis	ICD-10-CM
	**Cardiovascular Disease**		
410	Acute myocardial infarction	Diagnosis	ICD-9-CM
410.0	Acute myocardial infarction of anterolateral wall	Diagnosis	ICD-9-CM
410.00	Acute myocardial infarction of anterolateral wall, episode of care unspecified	Diagnosis	ICD-9-CM
410.01	Acute myocardial infarction of anterolateral wall, initial episode of care	Diagnosis	ICD-9-CM
410.02	Acute myocardial infarction of anterolateral wall, subsequent episode of care	Diagnosis	ICD-9-CM
410.1	Acute myocardial infarction of other anterior wall	Diagnosis	ICD-9-CM
410.10	Acute myocardial infarction of other anterior wall, episode of care unspecified	Diagnosis	ICD-9-CM
410.11	Acute myocardial infarction of other anterior wall, initial episode of care	Diagnosis	ICD-9-CM
410.12	Acute myocardial infarction of other anterior wall, subsequent episode of care	Diagnosis	ICD-9-CM
410.2	Acute myocardial infarction of inferolateral wall	Diagnosis	ICD-9-CM
410.20	Acute myocardial infarction of inferolateral wall, episode of care unspecified	Diagnosis	ICD-9-CM
410.21	Acute myocardial infarction of inferolateral wall, initial episode of care	Diagnosis	ICD-9-CM
410.22	Acute myocardial infarction of inferolateral wall, subsequent episode of care	Diagnosis	ICD-9-CM
410.3	Acute myocardial infarction of inferoposterior wall	Diagnosis	ICD-9-CM
410.30	Acute myocardial infarction of inferoposterior wall, episode of care unspecified	Diagnosis	ICD-9-CM
410.31	Acute myocardial infarction of inferoposterior wall, initial episode of care	Diagnosis	ICD-9-CM
410.32	Acute myocardial infarction of inferoposterior wall, subsequent episode of care	Diagnosis	ICD-9-CM
410.4	Acute myocardial infarction of other inferior wall	Diagnosis	ICD-9-CM
410.40	Acute myocardial infarction of other inferior wall, episode of care unspecified	Diagnosis	ICD-9-CM
410.41	Acute myocardial infarction of other inferior wall, initial episode of care	Diagnosis	ICD-9-CM
410.42	Acute myocardial infarction of other inferior wall, subsequent episode of care	Diagnosis	ICD-9-CM
410.5	Acute myocardial infarction of other lateral wall	Diagnosis	ICD-9-CM
410.50	Acute myocardial infarction of other lateral wall, episode of care unspecified	Diagnosis	ICD-9-CM
410.51	Acute myocardial infarction of other lateral wall, initial episode of care	Diagnosis	ICD-9-CM
410.52	Acute myocardial infarction of other lateral wall, subsequent episode of care	Diagnosis	ICD-9-CM
410.6	Acute myocardial infarction, true posterior wall infarction	Diagnosis	ICD-9-CM
410.60	Acute myocardial infarction, true posterior wall infarction, episode of care unspecified	Diagnosis	ICD-9-CM
410.61	Acute myocardial infarction, true posterior wall infarction, initial episode of care	Diagnosis	ICD-9-CM
410.62	Acute myocardial infarction, true posterior wall infarction, subsequent episode of care	Diagnosis	ICD-9-CM
410.7	Acute myocardial infarction, subendocardial infarction	Diagnosis	ICD-9-CM
410.70	Acute myocardial infarction, subendocardial infarction, episode of care unspecified	Diagnosis	ICD-9-CM
410.71	Acute myocardial infarction, subendocardial infarction, initial episode of care	Diagnosis	ICD-9-CM
410.72	Acute myocardial infarction, subendocardial infarction, subsequent episode of care	Diagnosis	ICD-9-CM
410.8	Acute myocardial infarction of other specified sites	Diagnosis	ICD-9-CM
410.80	Acute myocardial infarction of other specified sites, episode of care unspecified	Diagnosis	ICD-9-CM
410.81	Acute myocardial infarction of other specified sites, initial episode of care	Diagnosis	ICD-9-CM
410.82	Acute myocardial infarction of other specified sites, subsequent episode of care	Diagnosis	ICD-9-CM

Code	Description	Code Category	Code Type
410.9	Acute myocardial infarction, unspecified site	Diagnosis	ICD-9-CM
410.90	Acute myocardial infarction, unspecified site, episode of care unspecified	Diagnosis	ICD-9-CM
410.91	Acute myocardial infarction, unspecified site, initial episode of care	Diagnosis	ICD-9-CM
410.92	Acute myocardial infarction, unspecified site, subsequent episode of care	Diagnosis	ICD-9-CM
411	Other acute and subacute forms of ischemic heart disease	Diagnosis	ICD-9-CM
411.0	Postmyocardial infarction syndrome	Diagnosis	ICD-9-CM
411.1	Intermediate coronary syndrome	Diagnosis	ICD-9-CM
411.8	Other acute and subacute forms of ischemic heart disease	Diagnosis	ICD-9-CM
411.81	Acute coronary occlusion without myocardial infarction	Diagnosis	ICD-9-CM
411.89	Other acute and subacute form of ischemic heart disease	Diagnosis	ICD-9-CM
412	Old myocardial infarction	Diagnosis	ICD-9-CM
413	Angina pectoris	Diagnosis	ICD-9-CM
413.0	Angina decubitus	Diagnosis	ICD-9-CM
413.1	Prinzmetal angina	Diagnosis	ICD-9-CM
413.9	Other and unspecified angina pectoris	Diagnosis	ICD-9-CM
414	Other forms of chronic ischemic heart disease	Diagnosis	ICD-9-CM
414.0	Coronary atherosclerosis	Diagnosis	ICD-9-CM
414.00	Coronary atherosclerosis of unspecified type of vessel, native or graft	Diagnosis	ICD-9-CM
414.01	Coronary atherosclerosis of native coronary artery	Diagnosis	ICD-9-CM
414.02	Coronary atherosclerosis of autologous vein bypass graft	Diagnosis	ICD-9-CM
414.03	Coronary atherosclerosis of nonautologous biological bypass graft	Diagnosis	ICD-9-CM
414.04	Coronary atherosclerosis of artery bypass graft	Diagnosis	ICD-9-CM
414.05	Coronary atherosclerosis of unspecified type of bypass graft	Diagnosis	ICD-9-CM
414.06	Coronary atherosclerosis, of native coronary artery of transplanted heart	Diagnosis	ICD-9-CM
414.07	Coronary atherosclerosis, of bypass graft (artery) (vein) of transplanted heart	Diagnosis	ICD-9-CM
414.1	Aneurysm and dissection of heart	Diagnosis	ICD-9-CM
414.10	Aneurysm of heart	Diagnosis	ICD-9-CM
414.11	Aneurysm of coronary vessels	Diagnosis	ICD-9-CM
414.12	Dissection of coronary artery	Diagnosis	ICD-9-CM
414.19	Other aneurysm of heart	Diagnosis	ICD-9-CM
414.2	Chronic total occlusion of coronary artery	Diagnosis	ICD-9-CM
414.3	Coronary atherosclerosis due to lipid rich plaque	Diagnosis	ICD-9-CM
414.4	Coronary atherosclerosis due to calcified coronary lesion	Diagnosis	ICD-9-CM
414.8	Other specified forms of chronic ischemic heart disease	Diagnosis	ICD-9-CM
414.9	Unspecified chronic ischemic heart disease	Diagnosis	ICD-9-CM
I20.0	Unstable angina	Diagnosis	ICD-10-CM
I20.1	Angina pectoris with documented spasm	Diagnosis	ICD-10-CM
I20.8	Other forms of angina pectoris	Diagnosis	ICD-10-CM
I20.9	Angina pectoris, unspecified	Diagnosis	ICD-10-CM
I21.01	ST elevation (STEMI) myocardial infarction involving left main coronary artery	Diagnosis	ICD-10-CM
I21.02	ST elevation (STEMI) myocardial infarction involving left anterior descending coronary artery	Diagnosis	ICD-10-CM
I21.09	ST elevation (STEMI) myocardial infarction involving other coronary artery of anterior wall	Diagnosis	ICD-10-CM
I21.11	ST elevation (STEMI) myocardial infarction involving right coronary artery	Diagnosis	ICD-10-CM

Code	Description	Code Category	Code Type
I21.19	ST elevation (STEMI) myocardial infarction involving other coronary artery of inferior wall	Diagnosis	ICD-10-CM
I21.21	ST elevation (STEMI) myocardial infarction involving left circumflex coronary artery	Diagnosis	ICD-10-CM
I21.29	ST elevation (STEMI) myocardial infarction involving other sites	Diagnosis	ICD-10-CM
I21.3	ST elevation (STEMI) myocardial infarction of unspecified site	Diagnosis	ICD-10-CM
I21.4	Non-ST elevation (NSTEMI) myocardial infarction	Diagnosis	ICD-10-CM
I21.9	Acute myocardial infarction, unspecified	Diagnosis	ICD-10-CM
I21.A1	Myocardial infarction type 2	Diagnosis	ICD-10-CM
I21.A9	Other myocardial infarction type	Diagnosis	ICD-10-CM
I22.0	Subsequent ST elevation (STEMI) myocardial infarction of anterior wall	Diagnosis	ICD-10-CM
I22.1	Subsequent ST elevation (STEMI) myocardial infarction of inferior wall	Diagnosis	ICD-10-CM
I22.2	Subsequent non-ST elevation (NSTEMI) myocardial infarction	Diagnosis	ICD-10-CM
I22.8	Subsequent ST elevation (STEMI) myocardial infarction of other sites	Diagnosis	ICD-10-CM
I22.9	Subsequent ST elevation (STEMI) myocardial infarction of unspecified site	Diagnosis	ICD-10-CM
I24.0	Acute coronary thrombosis not resulting in myocardial infarction	Diagnosis	ICD-10-CM
I24.1	Dressler's syndrome	Diagnosis	ICD-10-CM
I24.8	Other forms of acute ischemic heart disease	Diagnosis	ICD-10-CM
I24.9	Acute ischemic heart disease, unspecified	Diagnosis	ICD-10-CM
I25.10	Atherosclerotic heart disease of native coronary artery without angina pectoris	Diagnosis	ICD-10-CM
I25.110	Atherosclerotic heart disease of native coronary artery with unstable angina pectoris	Diagnosis	ICD-10-CM
I25.111	Atherosclerotic heart disease of native coronary artery with angina pectoris with documented spasm	Diagnosis	ICD-10-CM
I25.118	Atherosclerotic heart disease of native coronary artery with other forms of angina pectoris	Diagnosis	ICD-10-CM
I25.119	Atherosclerotic heart disease of native coronary artery with unspecified angina pectoris	Diagnosis	ICD-10-CM
I25.2	Old myocardial infarction	Diagnosis	ICD-10-CM
I25.3	Aneurysm of heart	Diagnosis	ICD-10-CM
I25.41	Coronary artery aneurysm	Diagnosis	ICD-10-CM
I25.42	Coronary artery dissection	Diagnosis	ICD-10-CM
I25.5	Ischemic cardiomyopathy	Diagnosis	ICD-10-CM
I25.6	Silent myocardial ischemia	Diagnosis	ICD-10-CM
I25.700	Atherosclerosis of coronary artery bypass graft(s), unspecified, with unstable angina pectoris	Diagnosis	ICD-10-CM
I25.701	Atherosclerosis of coronary artery bypass graft(s), unspecified, with angina pectoris with documented spasm	Diagnosis	ICD-10-CM
I25.708	Atherosclerosis of coronary artery bypass graft(s), unspecified, with other forms of angina pectoris	Diagnosis	ICD-10-CM
I25.709	Atherosclerosis of coronary artery bypass graft(s), unspecified, with unspecified angina pectoris	Diagnosis	ICD-10-CM
I25.710	Atherosclerosis of autologous vein coronary artery bypass graft(s) with unstable angina pectoris	Diagnosis	ICD-10-CM

Code	Description	Code Category	Code Type
I25.711	Atherosclerosis of autologous vein coronary artery bypass graft(s) with angina pectoris with documented spasm	Diagnosis	ICD-10-CM
I25.718	Atherosclerosis of autologous vein coronary artery bypass graft(s) with other forms of angina pectoris	Diagnosis	ICD-10-CM
I25.719	Atherosclerosis of autologous vein coronary artery bypass graft(s) with unspecified angina pectoris	Diagnosis	ICD-10-CM
I25.720	Atherosclerosis of autologous artery coronary artery bypass graft(s) with unstable angina pectoris	Diagnosis	ICD-10-CM
I25.721	Atherosclerosis of autologous artery coronary artery bypass graft(s) with angina pectoris with documented spasm	Diagnosis	ICD-10-CM
I25.728	Atherosclerosis of autologous artery coronary artery bypass graft(s) with other forms of angina pectoris	Diagnosis	ICD-10-CM
I25.729	Atherosclerosis of autologous artery coronary artery bypass graft(s) with unspecified angina pectoris	Diagnosis	ICD-10-CM
I25.730	Atherosclerosis of nonautologous biological coronary artery bypass graft(s) with unstable angina pectoris	Diagnosis	ICD-10-CM
I25.731	Atherosclerosis of nonautologous biological coronary artery bypass graft(s) with angina pectoris with documented spasm	Diagnosis	ICD-10-CM
I25.738	Atherosclerosis of nonautologous biological coronary artery bypass graft(s) with other forms of angina pectoris	Diagnosis	ICD-10-CM
I25.739	Atherosclerosis of nonautologous biological coronary artery bypass graft(s) with unspecified angina pectoris	Diagnosis	ICD-10-CM
I25.750	Atherosclerosis of native coronary artery of transplanted heart with unstable angina	Diagnosis	ICD-10-CM
I25.751	Atherosclerosis of native coronary artery of transplanted heart with angina pectoris with documented spasm	Diagnosis	ICD-10-CM
I25.758	Atherosclerosis of native coronary artery of transplanted heart with other forms of angina pectoris	Diagnosis	ICD-10-CM
I25.759	Atherosclerosis of native coronary artery of transplanted heart with unspecified angina pectoris	Diagnosis	ICD-10-CM
I25.760	Atherosclerosis of bypass graft of coronary artery of transplanted heart with unstable angina	Diagnosis	ICD-10-CM
I25.761	Atherosclerosis of bypass graft of coronary artery of transplanted heart with angina pectoris with documented spasm	Diagnosis	ICD-10-CM
I25.768	Atherosclerosis of bypass graft of coronary artery of transplanted heart with other forms of angina pectoris	Diagnosis	ICD-10-CM
I25.769	Atherosclerosis of bypass graft of coronary artery of transplanted heart with unspecified angina pectoris	Diagnosis	ICD-10-CM
I25.790	Atherosclerosis of other coronary artery bypass graft(s) with unstable angina pectoris	Diagnosis	ICD-10-CM
I25.791	Atherosclerosis of other coronary artery bypass graft(s) with angina pectoris with documented spasm	Diagnosis	ICD-10-CM
I25.798	Atherosclerosis of other coronary artery bypass graft(s) with other forms of angina pectoris	Diagnosis	ICD-10-CM

Code	Description	Code Category	Code Type
I25.799	Atherosclerosis of other coronary artery bypass graft(s) with unspecified angina pectoris	Diagnosis	ICD-10-CM
I25.810	Atherosclerosis of coronary artery bypass graft(s) without angina pectoris	Diagnosis	ICD-10-CM
I25.811	Atherosclerosis of native coronary artery of transplanted heart without angina pectoris	Diagnosis	ICD-10-CM
I25.812	Atherosclerosis of bypass graft of coronary artery of transplanted heart without angina pectoris	Diagnosis	ICD-10-CM
I25.82	Chronic total occlusion of coronary artery	Diagnosis	ICD-10-CM
I25.83	Coronary atherosclerosis due to lipid rich plaque	Diagnosis	ICD-10-CM
I25.84	Coronary atherosclerosis due to calcified coronary lesion	Diagnosis	ICD-10-CM
I25.89	Other forms of chronic ischemic heart disease	Diagnosis	ICD-10-CM
I25.9	Chronic ischemic heart disease, unspecified	Diagnosis	ICD-10-CM
**Congenital Heart Disease**			
P293	Persistent fetal circulation	Diagnosis	ICD-10-CM
P298	Oth cardiovasc disorders originating in the perinatal period	Diagnosis	ICD-10-CM
P2938	Other persistent fetal circulation	Diagnosis	ICD-10-CM
Q20	Congenital malformations of cardiac chambers and connections	Diagnosis	ICD-10-CM
Q21	Congenital malformations of cardiac septa	Diagnosis	ICD-10-CM
Q22	Congenital malformations of pulmonary and tricuspid valves	Diagnosis	ICD-10-CM
Q23	Congenital malformations of aortic and mitral valves	Diagnosis	ICD-10-CM
Q24	Other congenital malformations of heart	Diagnosis	ICD-10-CM
Q200	Common arterial trunk	Diagnosis	ICD-10-CM
Q201	Double outlet right ventricle	Diagnosis	ICD-10-CM
Q202	Double outlet left ventricle	Diagnosis	ICD-10-CM
Q203	Discordant ventriculoarterial connection	Diagnosis	ICD-10-CM
Q204	Double inlet ventricle	Diagnosis	ICD-10-CM
Q205	Discordant atrioventricular connection	Diagnosis	ICD-10-CM
Q206	Isomerism of atrial appendages	Diagnosis	ICD-10-CM
Q208	Oth congenital malform of cardiac chambers and connections	Diagnosis	ICD-10-CM
Q209	Congenital malform of cardiac chambers and connections, unsp	Diagnosis	ICD-10-CM
Q210	Ventricular septal defect	Diagnosis	ICD-10-CM
Q211	Atrial septal defect	Diagnosis	ICD-10-CM
Q212	Atrioventricular septal defect	Diagnosis	ICD-10-CM
Q213	Tetralogy of Fallot	Diagnosis	ICD-10-CM
Q214	Aortopulmonary septal defect	Diagnosis	ICD-10-CM
Q218	Other congenital malformations of cardiac septa	Diagnosis	ICD-10-CM
Q219	Congenital malformation of cardiac septum, unspecified	Diagnosis	ICD-10-CM
Q220	Pulmonary valve atresia	Diagnosis	ICD-10-CM
Q221	Congenital pulmonary valve stenosis	Diagnosis	ICD-10-CM
Q222	Congenital pulmonary valve insufficiency	Diagnosis	ICD-10-CM
Q223	Other congenital malformations of pulmonary valve	Diagnosis	ICD-10-CM
Q224	Congenital tricuspid stenosis	Diagnosis	ICD-10-CM
Q225	Ebstein's anomaly	Diagnosis	ICD-10-CM
Q226	Hypoplastic right heart syndrome	Diagnosis	ICD-10-CM
Q228	Other congenital malformations of tricuspid valve	Diagnosis	ICD-10-CM

Code	Description	Code Category	Code Type
Q229	Congenital malformation of tricuspid valve, unspecified	Diagnosis	ICD-10-CM
Q230	Congenital stenosis of aortic valve	Diagnosis	ICD-10-CM
Q231	Congenital insufficiency of aortic valve	Diagnosis	ICD-10-CM
Q232	Congenital mitral stenosis	Diagnosis	ICD-10-CM
Q233	Congenital mitral insufficiency	Diagnosis	ICD-10-CM
Q234	Hypoplastic left heart syndrome	Diagnosis	ICD-10-CM
Q238	Other congenital malformations of aortic and mitral valves	Diagnosis	ICD-10-CM
Q239	Congenital malformation of aortic and mitral valves, unsp	Diagnosis	ICD-10-CM
Q240	Dextrocardia	Diagnosis	ICD-10-CM
Q241	Levocardia	Diagnosis	ICD-10-CM
Q242	Cor triatriatum	Diagnosis	ICD-10-CM
Q243	Pulmonary infundibular stenosis	Diagnosis	ICD-10-CM
Q244	Congenital subaortic stenosis	Diagnosis	ICD-10-CM
Q245	Malformation of coronary vessels	Diagnosis	ICD-10-CM
Q246	Congenital heart block	Diagnosis	ICD-10-CM
Q248	Other specified congenital malformations of heart	Diagnosis	ICD-10-CM
Q249	Congenital malformation of heart, unspecified	Diagnosis	ICD-10-CM
Q250	Patent ductus arteriosus	Diagnosis	ICD-10-CM
Q251	Coarctation of aorta	Diagnosis	ICD-10-CM
Q2521	Interruption of aortic arch	Diagnosis	ICD-10-CM
Q2529	Other atresia of aorta	Diagnosis	ICD-10-CM
Q253	Supravalvular aortic stenosis	Diagnosis	ICD-10-CM
Q2540	Congenital malformation of aorta unspecified	Diagnosis	ICD-10-CM
Q2541	Absence and aplasia of aorta	Diagnosis	ICD-10-CM
Q2542	Hypoplasia of aorta	Diagnosis	ICD-10-CM
Q2543	Congenital aneurysm of aorta	Diagnosis	ICD-10-CM
Q2544	Congenital dilation of aorta	Diagnosis	ICD-10-CM
Q2545	Double aortic arch	Diagnosis	ICD-10-CM
Q2546	Tortuous aortic arch	Diagnosis	ICD-10-CM
Q2547	Right aortic arch	Diagnosis	ICD-10-CM
Q2548	Anomalous origin of subclavian artery	Diagnosis	ICD-10-CM
Q2549	Other congenital malformations of aorta	Diagnosis	ICD-10-CM
Q255	Atresia of pulmonary artery	Diagnosis	ICD-10-CM
Q256	Stenosis of pulmonary artery	Diagnosis	ICD-10-CM
Q2571	Coarctation of pulmonary artery	Diagnosis	ICD-10-CM
Q2572	Congenital pulmonary arteriovenous malformation	Diagnosis	ICD-10-CM
Q2579	Other congenital malformations of pulmonary artery	Diagnosis	ICD-10-CM
Q258	Other congenital malformations of other great arteries	Diagnosis	ICD-10-CM
Q259	Congenital malformation of great arteries, unspecified	Diagnosis	ICD-10-CM
Q260	Congenital stenosis of vena cava	Diagnosis	ICD-10-CM
Q261	Persistent left superior vena cava	Diagnosis	ICD-10-CM
Q262	Total anomalous pulmonary venous connection	Diagnosis	ICD-10-CM
Q263	Partial anomalous pulmonary venous connection	Diagnosis	ICD-10-CM
Q264	Anomalous pulmonary venous connection, unspecified	Diagnosis	ICD-10-CM
Q8740	Marfan's syndrome, unspecified	Diagnosis	ICD-10-CM

Code	Description	Code Category	Code Type
Q8741	Marfan's syndrome with cardiovascular manifestations	Diagnosis	ICD-10-CM
Q87410	Marfan's syndrome with aortic dilation	Diagnosis	ICD-10-CM
Q87418	Marfan's syndrome with other cardiovascular manifestations	Diagnosis	ICD-10-CM
Q8782	Arterial tortuosity syndrome	Diagnosis	ICD-10-CM
Q900	Trisomy 21, nonmosaicism (meiotic nondisjunction)	Diagnosis	ICD-10-CM
Q901	Trisomy 21, mosaicism (mitotic nondisjunction)	Diagnosis	ICD-10-CM
Q902	Trisomy 21, translocation	Diagnosis	ICD-10-CM
Q909	Down syndrome, unspecified	Diagnosis	ICD-10-CM
Q910	Trisomy 18, nonmosaicism (meiotic nondisjunction)	Diagnosis	ICD-10-CM
Q911	Trisomy 18, mosaicism (mitotic nondisjunction)	Diagnosis	ICD-10-CM
Q912	Trisomy 18, translocation	Diagnosis	ICD-10-CM
Q913	Trisomy 18, unspecified	Diagnosis	ICD-10-CM
Q914	Trisomy 13, nonmosaicism (meiotic nondisjunction)	Diagnosis	ICD-10-CM
Q915	Trisomy 13, mosaicism (mitotic nondisjunction)	Diagnosis	ICD-10-CM
Q916	Trisomy 13, translocation	Diagnosis	ICD-10-CM
Q917	Trisomy 13, unspecified	Diagnosis	ICD-10-CM
Q9381	Velo-cardio-facial syndrome	Diagnosis	ICD-10-CM
Q9382	Williams syndrome	Diagnosis	ICD-10-CM
02Q50ZZ	Repair Atrial Septum, Open Approach	Procedure	ICD-10-PCS
02U507Z	Supplement Atrial Septum with Autologous Tissue Substitute, Open Approach	Procedure	ICD-10-PCS
02U50JZ	Supplement Atrial Septum with Synthetic Substitute, Open Approach	Procedure	ICD-10-PCS
02U53JZ	Supplement Atrial Septum with Synthetic Substitute, Percutaneous Approach	Procedure	ICD-10-PCS
02QM0ZZ	Repair Ventricular Septum, Open Approach	Procedure	ICD-10-PCS
02UM07Z	Supplement Ventricular Septum with Autologous Tissue Substitute, Open Approach	Procedure	ICD-10-PCS
02UM0JZ	Supplement Ventricular Septum with Synthetic Substitute, Open Approach	Procedure	ICD-10-PCS
02UM3JZ	Supplement Ventricular Septum with Synthetic Substitute, Percutaneous Approach	Procedure	ICD-10-PCS
93530	Right heart catheterization, for congenital cardiac anomalies	Procedure	CPT-4
93531	Combined right heart catheterization and retrograde left heart catheterization, for congenital cardiac anomalies	Procedure	CPT-4
93532	Combined right heart catheterization and transseptal left heart catheterization through intact septum with or without retrograde left heart catheterization, for congenital cardiac anomalies	Procedure	CPT-4
93533	Combined right heart catheterization and transseptal left heart catheterization through existing septal opening, with or without retrograde left heart catheterization, for congenital cardiac anomalies	Procedure	CPT-4
75573	Computed tomography, heart, with contrast material, for evaluation of cardiac structure and morphology in the setting of congenital heart disease (including 3D image postprocessing, assessment of LV cardiac function, RV structure and function and evaluati	Procedure	CPT-4
75572	Computed tomography, heart, with contrast material, for evaluation of cardiac structure and morphology (including 3D image postprocessing, assessment of cardiac function, and evaluation of venous structures, if performed)	Procedure	CPT-4
33924	Ligation and takedown of a systemic-to-pulmonary artery shunt, performed in conjunction with a congenital heart procedure (List separately in addition to code for primary procedure)	Procedure	CPT-4

Code	Description	Code Category	Code Type
93563	Injection procedure during cardiac catheterization including imaging supervision, interpretation, and report, for selective coronary angiography during congenital heart catheterization (List separately in addition to code for primary procedure)	Procedure	CPT-4
93564	Injection procedure during cardiac catheterization including imaging supervision, interpretation, and report, for selective opacification of aortocoronary venous or arterial bypass graft(s) (eg, aortocoronary saphenous vein, free radial artery, or free ma	Procedure	CPT-4
33647	Repair of atrial septal defect and ventricular septal defect, with direct or patch closure	Procedure	CPT-4
33720	Repair sinus of Valsalva aneurysm, with cardiopulmonary bypass	Procedure	CPT-4
33745	Transcatheter intracardiac shunt (TIS) creation by stent placement for congenital cardiac anomalies to establish effective intracardiac flow, including all imaging guidance by the proceduralist, when performed, left and right heart diagnostic cardiac cath	Procedure	CPT-4
33746	Transcatheter intracardiac shunt (TIS) creation by stent placement for congenital cardiac anomalies to establish effective intracardiac flow, including all imaging guidance by the proceduralist, when performed, left and right heart diagnostic cardiac cath	Procedure	CPT-4
33741	Transcatheter atrial septostomy (TAS) for congenital cardiac anomalies to create effective atrial flow, including all imaging guidance by the proceduralist, when performed, any method (eg, Rashkind, Sang-Park, balloon, cutting balloon, blade)	Procedure	CPT-4
93303	Transthoracic echocardiography for congenital cardiac anomalies, complete	Procedure	CPT-4
93304	Transthoracic echocardiography for congenital cardiac anomalies, follow-up or limited	Procedure	CPT-4
93316	Transesophageal echocardiography for congenital cardiac anomalies, placement of transesophageal probe only, image acquisition, interpretation and report only	Procedure	CPT-4
93317	Transesophageal echocardiography for congenital cardiac anomalies	Procedure	CPT-4
93315	Transesophageal echocardiography for congenital cardiac anomalies, including probe placement, image acquisition, interpretation and report	Procedure	CPT-4
93581	Percutaneous transcatheter closure of a congenital ventricular septal defect with implant	Procedure	CPT-4
93580	Percutaneous transcatheter closure of congenital interatrial communication (ie, Fontan fenestration, atrial septal defect) with implant	Procedure	CPT-4
	**Hematological Disorders**		
280	Iron deficiency anemias	Diagnosis	ICD-9-CM
281.0	Pernicious anemia	Diagnosis	ICD-9-CM
281.1	Other vitamin B12 deficiency anemia	Diagnosis	ICD-9-CM
281.2	Folate-deficiency anemia	Diagnosis	ICD-9-CM
281.3	Other specified megaloblastic anemias not elsewhere classified	Diagnosis	ICD-9-CM
282.4	Thalassemias	Diagnosis	ICD-9-CM
282.40	Thalassemia, unspecified	Diagnosis	ICD-9-CM
282.41	Sickle-cell thalassemia without crisis	Diagnosis	ICD-9-CM
282.42	Sickle-cell thalassemia with crisis	Diagnosis	ICD-9-CM
282.43	Alpha thalassemia	Diagnosis	ICD-9-CM
282.44	Beta thalassemia	Diagnosis	ICD-9-CM

Code	Description	Code Category	Code Type
282.45	Delta-beta thalassemia	Diagnosis	ICD-9-CM
282.46	Thalassemia minor	Diagnosis	ICD-9-CM
282.47	Hemoglobin E-beta thalassemia	Diagnosis	ICD-9-CM
282.49	Other thalassemia	Diagnosis	ICD-9-CM
282.5	Sickle-cell trait	Diagnosis	ICD-9-CM
282.6	Sickle-cell disease	Diagnosis	ICD-9-CM
282.60	Sickle-cell disease, unspecified	Diagnosis	ICD-9-CM
282.63	Sickle-cell/Hb-C disease without crisis	Diagnosis	ICD-9-CM
282.64	Sickle-cell/Hb-C disease with crisis	Diagnosis	ICD-9-CM
282.68	Other sickle-cell disease without crisis	Diagnosis	ICD-9-CM
282.69	Other sickle-cell disease with crisis	Diagnosis	ICD-9-CM
282.7	Other hemoglobinopathies	Diagnosis	ICD-9-CM
282.8	Other specified hereditary hemolytic anemias	Diagnosis	ICD-9-CM
282.9	Unspecified hereditary hemolytic anemia	Diagnosis	ICD-9-CM
283	Acquired hemolytic anemias	Diagnosis	ICD-9-CM
283.0	Autoimmune hemolytic anemias	Diagnosis	ICD-9-CM
283.1	Non-autoimmune hemolytic anemias	Diagnosis	ICD-9-CM
283.10	Unspecified non-autoimmune hemolytic anemia	Diagnosis	ICD-9-CM
283.11	Hemolytic-uremic syndrome	Diagnosis	ICD-9-CM
283.19	Other non-autoimmune hemolytic anemias	Diagnosis	ICD-9-CM
283.9	Acquired hemolytic anemia, unspecified	Diagnosis	ICD-9-CM
284	Aplastic anemia and other bone marrow failure syndromes	Diagnosis	ICD-9-CM
284.0	Constitutional aplastic anemia	Diagnosis	ICD-9-CM
284.01	Constitutional red blood cell aplasia	Diagnosis	ICD-9-CM
284.09	Other constitutional aplastic anemia	Diagnosis	ICD-9-CM
284.1	Pancytopenia	Diagnosis	ICD-9-CM
284.11	Antineoplastic chemotherapy induced pancytopenia	Diagnosis	ICD-9-CM
284.12	Other drug induced pancytopenia	Diagnosis	ICD-9-CM
284.19	Other pancytopenia	Diagnosis	ICD-9-CM
284.2	Myelophthisis	Diagnosis	ICD-9-CM
284.8	Other specified aplastic anemias	Diagnosis	ICD-9-CM
284.81	Red cell aplasia (acquired) (adult) (with thymoma)	Diagnosis	ICD-9-CM
284.89	Other specified aplastic anemias	Diagnosis	ICD-9-CM
284.9	Unspecified aplastic anemia	Diagnosis	ICD-9-CM
285	Other and unspecified anemias	Diagnosis	ICD-9-CM
285.0	Sideroblastic anemia	Diagnosis	ICD-9-CM
285.1	Acute posthemorrhagic anemia	Diagnosis	ICD-9-CM
285.2	Anemia of chronic disease	Diagnosis	ICD-9-CM
285.21	Anemia in chronic kidney disease	Diagnosis	ICD-9-CM
285.22	Anemia in neoplastic disease	Diagnosis	ICD-9-CM
285.29	Anemia of other chronic disease	Diagnosis	ICD-9-CM
285.3	Antineoplastic chemotherapy induced anemia	Diagnosis	ICD-9-CM
285.8	Other specified anemias	Diagnosis	ICD-9-CM
285.9	Unspecified anemia	Diagnosis	ICD-9-CM
286	Coagulation defects	Diagnosis	ICD-9-CM

Code	Description	Code Category	Code Type
286.0	Congenital factor VIII disorder	Diagnosis	ICD-9-CM
286.1	Congenital factor IX disorder	Diagnosis	ICD-9-CM
286.2	Congenital factor XI deficiency	Diagnosis	ICD-9-CM
286.3	Congenital deficiency of other clotting factors	Diagnosis	ICD-9-CM
286.4	Von Willebrand's disease	Diagnosis	ICD-9-CM
286.5	Hemorrhagic disorder due to intrinsic circulating anticoagulants, antibodies or inhibi	Diagnosis	ICD-9-CM
286.52	Acquired hemophilia	Diagnosis	ICD-9-CM
286.53	Antiphospholipid antibody with hemorrhagic disorder	Diagnosis	ICD-9-CM
286.59	Other hemorrhagic disorder due to intrinsic circulating anticoagulants, antibodies, o	Diagnosis	ICD-9-CM
286.6	Defibrination syndrome	Diagnosis	ICD-9-CM
286.7	Acquired coagulation factor deficiency	Diagnosis	ICD-9-CM
286.9	Other and unspecified coagulation defects	Diagnosis	ICD-9-CM
287	Purpura and other hemorrhagic conditions	Diagnosis	ICD-9-CM
287.0	Allergic purpura	Diagnosis	ICD-9-CM
287.1	Qualitative platelet defects	Diagnosis	ICD-9-CM
287.2	Other nonthrombocytopenic purpuras	Diagnosis	ICD-9-CM
287.3	Primary thrombocytopenia	Diagnosis	ICD-9-CM
287.30	Primary thrombocytopenia, unspecified	Diagnosis	ICD-9-CM
287.31	Immune thrombocytopenic purpura	Diagnosis	ICD-9-CM
287.32	Evans' syndrome	Diagnosis	ICD-9-CM
287.33	Congenital and hereditary thrombocytopenic purpura	Diagnosis	ICD-9-CM
287.39	Other primary thrombocytopenia	Diagnosis	ICD-9-CM
287.4	Secondary thrombocytopenia	Diagnosis	ICD-9-CM
287.41	Posttransfusion purpura	Diagnosis	ICD-9-CM
287.49	Other secondary thrombocytopenia	Diagnosis	ICD-9-CM
287.5	Unspecified thrombocytopenia	Diagnosis	ICD-9-CM
287.8	Other specified hemorrhagic conditions	Diagnosis	ICD-9-CM
287.9	Unspecified hemorrhagic conditions	Diagnosis	ICD-9-CM
288	Diseases of white blood cells	Diagnosis	ICD-9-CM
288.0	Neutropenia	Diagnosis	ICD-9-CM
288.00	Neutropenia, unspecified	Diagnosis	ICD-9-CM
288.01	Congenital neutropenia	Diagnosis	ICD-9-CM
288.02	Cyclic neutropenia	Diagnosis	ICD-9-CM
288.03	Drug induced neutropenia	Diagnosis	ICD-9-CM
288.04	Neutropenia due to infection	Diagnosis	ICD-9-CM
288.09	Other neutropenia	Diagnosis	ICD-9-CM
288.1	Functional disorders of polymorphonuclear neutrophils	Diagnosis	ICD-9-CM
288.2	Genetic anomalies of leukocytes	Diagnosis	ICD-9-CM
288.3	Eosinophilia	Diagnosis	ICD-9-CM
288.4	Hemophagocytic syndromes	Diagnosis	ICD-9-CM
288.5	Decreased white blood cell count	Diagnosis	ICD-9-CM
288.50	Leukocytopenia, unspecified	Diagnosis	ICD-9-CM
288.51	Lymphocytopenia	Diagnosis	ICD-9-CM
288.59	Other decreased white blood cell count	Diagnosis	ICD-9-CM
288.66	Bandemia	Diagnosis	ICD-9-CM

Code	Description	Code Category	Code Type
288.69	Other elevated white blood cell count	Diagnosis	ICD-9-CM
288.8	Other specified disease of white blood cells	Diagnosis	ICD-9-CM
288.9	Unspecified disease of white blood cells	Diagnosis	ICD-9-CM
289	Other diseases of blood and blood-forming organs	Diagnosis	ICD-9-CM
289.0	Polycythemia, secondary	Diagnosis	ICD-9-CM
289.4	Hypersplenism	Diagnosis	ICD-9-CM
289.5	Other diseases of spleen	Diagnosis	ICD-9-CM
289.50	Unspecified disease of spleen	Diagnosis	ICD-9-CM
289.51	Chronic congestive splenomegaly	Diagnosis	ICD-9-CM
289.52	Splenic sequestration	Diagnosis	ICD-9-CM
289.53	Neutropenic splenomegaly	Diagnosis	ICD-9-CM
289.59	Other diseases of spleen	Diagnosis	ICD-9-CM
289.6	Familial polycythemia	Diagnosis	ICD-9-CM
289.83	Myelofibrosis	Diagnosis	ICD-9-CM
289.84	Heparin-induced thrombocytopenia [HIT]	Diagnosis	ICD-9-CM
289.89	Other specified diseases of blood and blood-forming organs	Diagnosis	ICD-9-CM
289.9	Unspecified diseases of blood and blood-forming organs	Diagnosis	ICD-9-CM
D47.4	Osteomyelofibrosis	Diagnosis	ICD-10-CM
D50.9	Iron deficiency anemia, unspecified	Diagnosis	ICD-10-CM
D51.0	Vitamin B12 deficiency anemia due to intrinsic factor deficiency	Diagnosis	ICD-10-CM
D51.1	Vitamin B12 deficiency anemia due to selective vitamin B12 malabsorption with proteinuria	Diagnosis	ICD-10-CM
D51.2	Transcobalamin II deficiency	Diagnosis	ICD-10-CM
D51.3	Other dietary vitamin B12 deficiency anemia	Diagnosis	ICD-10-CM
D51.8	Other vitamin B12 deficiency anemias	Diagnosis	ICD-10-CM
D51.9	Vitamin B12 deficiency anemia, unspecified	Diagnosis	ICD-10-CM
D52.0	Dietary folate deficiency anemia	Diagnosis	ICD-10-CM
D52.1	Drug-induced folate deficiency anemia	Diagnosis	ICD-10-CM
D52.8	Other folate deficiency anemias	Diagnosis	ICD-10-CM
D52.9	Folate deficiency anemia, unspecified	Diagnosis	ICD-10-CM
D53.0	Protein deficiency anemia	Diagnosis	ICD-10-CM
D53.1	Other megaloblastic anemias, not elsewhere classified	Diagnosis	ICD-10-CM
D56.0	Alpha thalassemia	Diagnosis	ICD-10-CM
D56.1	Beta thalassemia	Diagnosis	ICD-10-CM
D56.2	Delta-beta thalassemia	Diagnosis	ICD-10-CM
D56.3	Thalassemia minor	Diagnosis	ICD-10-CM
D56.4	Hereditary persistence of fetal hemoglobin [HPFH]	Diagnosis	ICD-10-CM
D56.5	Hemoglobin E-beta thalassemia	Diagnosis	ICD-10-CM
D56.8	Other thalassemias	Diagnosis	ICD-10-CM
D56.9	Thalassemia, unspecified	Diagnosis	ICD-10-CM
D57.00	Hb-SS disease with crisis, unspecified	Diagnosis	ICD-10-CM
D57.01	Hb-SS disease with acute chest syndrome	Diagnosis	ICD-10-CM
D57.02	Hb-SS disease with splenic sequestration	Diagnosis	ICD-10-CM
D57.1	Sickle-cell disease without crisis	Diagnosis	ICD-10-CM
D57.20	Sickle-cell/Hb-C disease without crisis	Diagnosis	ICD-10-CM

Code	Description	Code Category	Code Type
D57.211	Sickle-cell/Hb-C disease with acute chest syndrome	Diagnosis	ICD-10-CM
D57.212	Sickle-cell/Hb-C disease with splenic sequestration	Diagnosis	ICD-10-CM
D57.219	Sickle-cell/Hb-C disease with crisis, unspecified	Diagnosis	ICD-10-CM
D57.3	Sickle-cell trait	Diagnosis	ICD-10-CM
D57.40	Sickle-cell thalassemia without crisis	Diagnosis	ICD-10-CM
D57.411	Sickle-cell thalassemia with acute chest syndrome	Diagnosis	ICD-10-CM
D57.412	Sickle-cell thalassemia with splenic sequestration	Diagnosis	ICD-10-CM
D57.419	Sickle-cell thalassemia with crisis, unspecified	Diagnosis	ICD-10-CM
D57.80	Other sickle-cell disorders without crisis	Diagnosis	ICD-10-CM
D57.811	Other sickle-cell disorders with acute chest syndrome	Diagnosis	ICD-10-CM
D57.812	Other sickle-cell disorders with splenic sequestration	Diagnosis	ICD-10-CM
D57.819	Other sickle-cell disorders with crisis, unspecified	Diagnosis	ICD-10-CM
D58.0	Hereditary spherocytosis	Diagnosis	ICD-10-CM
D58.1	Hereditary elliptocytosis	Diagnosis	ICD-10-CM
D58.2	Other hemoglobinopathies	Diagnosis	ICD-10-CM
D58.8	Other specified hereditary hemolytic anemias	Diagnosis	ICD-10-CM
D58.9	Hereditary hemolytic anemia, unspecified	Diagnosis	ICD-10-CM
D59.0	Drug-induced autoimmune hemolytic anemia	Diagnosis	ICD-10-CM
D59.1	Other autoimmune hemolytic anemias	Diagnosis	ICD-10-CM
D59.2	Drug-induced nonautoimmune hemolytic anemia	Diagnosis	ICD-10-CM
D59.3	Hemolytic-uremic syndrome	Diagnosis	ICD-10-CM
D59.4	Other nonautoimmune hemolytic anemias	Diagnosis	ICD-10-CM
D59.5	Paroxysmal nocturnal hemoglobinuria [Marchiafava-Micheli]	Diagnosis	ICD-10-CM
D59.6	Hemoglobinuria due to hemolysis from other external causes	Diagnosis	ICD-10-CM
D59.8	Other acquired hemolytic anemias	Diagnosis	ICD-10-CM
D59.9	Acquired hemolytic anemia, unspecified	Diagnosis	ICD-10-CM
D60.0	Chronic acquired pure red cell aplasia	Diagnosis	ICD-10-CM
D60.1	Transient acquired pure red cell aplasia	Diagnosis	ICD-10-CM
D60.8	Other acquired pure red cell aplasias	Diagnosis	ICD-10-CM
D60.9	Acquired pure red cell aplasia, unspecified	Diagnosis	ICD-10-CM
D61.01	Constitutional (pure) red blood cell aplasia	Diagnosis	ICD-10-CM
D61.09	Other constitutional aplastic anemia	Diagnosis	ICD-10-CM
D61.1	Drug-induced aplastic anemia	Diagnosis	ICD-10-CM
D61.2	Aplastic anemia due to other external agents	Diagnosis	ICD-10-CM
D61.3	Idiopathic aplastic anemia	Diagnosis	ICD-10-CM
D61.810	Antineoplastic chemotherapy induced pancytopenia	Diagnosis	ICD-10-CM
D61.811	Other drug-induced pancytopenia	Diagnosis	ICD-10-CM
D61.818	Other pancytopenia	Diagnosis	ICD-10-CM
D61.82	Myelophthisis	Diagnosis	ICD-10-CM
D61.89	Other specified aplastic anemias and other bone marrow failure syndromes	Diagnosis	ICD-10-CM
D61.9	Aplastic anemia, unspecified	Diagnosis	ICD-10-CM
D62	Acute posthemorrhagic anemia	Diagnosis	ICD-10-CM
D63.0	Anemia in neoplastic disease	Diagnosis	ICD-10-CM
D63.1	Anemia in chronic kidney disease	Diagnosis	ICD-10-CM
D63.8	Anemia in other chronic diseases classified elsewhere	Diagnosis	ICD-10-CM

Code	Description	Code Category	Code Type
D64.0	Hereditary sideroblastic anemia	Diagnosis	ICD-10-CM
D64.1	Secondary sideroblastic anemia due to disease	Diagnosis	ICD-10-CM
D64.2	Secondary sideroblastic anemia due to drugs and toxins	Diagnosis	ICD-10-CM
D64.3	Other sideroblastic anemias	Diagnosis	ICD-10-CM
D64.4	Congenital dyserythropoietic anemia	Diagnosis	ICD-10-CM
D64.81	Anemia due to antineoplastic chemotherapy	Diagnosis	ICD-10-CM
D64.89	Other specified anemias	Diagnosis	ICD-10-CM
D64.9	Anemia, unspecified	Diagnosis	ICD-10-CM
D65	Disseminated intravascular coagulation [defibrination syndrome]	Diagnosis	ICD-10-CM
D66	Hereditary factor VIII deficiency	Diagnosis	ICD-10-CM
D67	Hereditary factor IX deficiency	Diagnosis	ICD-10-CM
D68.0	Von Willebrand's disease	Diagnosis	ICD-10-CM
D68.1	Hereditary factor XI deficiency	Diagnosis	ICD-10-CM
D68.2	Hereditary deficiency of other clotting factors	Diagnosis	ICD-10-CM
D68.311	Acquired hemophilia	Diagnosis	ICD-10-CM
D68.312	Antiphospholipid antibody with hemorrhagic disorder	Diagnosis	ICD-10-CM
D68.318	Other hemorrhagic disorder due to intrinsic circulating anticoagulants, antibodies, or inhibitors	Diagnosis	ICD-10-CM
D68.32	Hemorrhagic disorder due to extrinsic circulating anticoagulants	Diagnosis	ICD-10-CM
D68.4	Acquired coagulation factor deficiency	Diagnosis	ICD-10-CM
D68.51	Activated protein C resistance	Diagnosis	ICD-10-CM
D68.52	Prothrombin gene mutation	Diagnosis	ICD-10-CM
D68.59	Other primary thrombophilia	Diagnosis	ICD-10-CM
D68.61	Antiphospholipid syndrome	Diagnosis	ICD-10-CM
D68.62	Lupus anticoagulant syndrome	Diagnosis	ICD-10-CM
D68.69	Other thrombophilia	Diagnosis	ICD-10-CM
D68.8	Other specified coagulation defects	Diagnosis	ICD-10-CM
D68.9	Coagulation defect, unspecified	Diagnosis	ICD-10-CM
D69.2	Other nonthrombocytopenic purpura	Diagnosis	ICD-10-CM
D69.3	Immune thrombocytopenic purpura	Diagnosis	ICD-10-CM
D69.41	Evans syndrome	Diagnosis	ICD-10-CM
D69.42	Congenital and hereditary thrombocytopenia purpura	Diagnosis	ICD-10-CM
D69.49	Other primary thrombocytopenia	Diagnosis	ICD-10-CM
D69.51	Posttransfusion purpura	Diagnosis	ICD-10-CM
D69.59	Other secondary thrombocytopenia	Diagnosis	ICD-10-CM
D69.6	Thrombocytopenia, unspecified	Diagnosis	ICD-10-CM
D69.8	Other specified hemorrhagic conditions	Diagnosis	ICD-10-CM
D69.9	Hemorrhagic condition, unspecified	Diagnosis	ICD-10-CM
D70.0	Congenital agranulocytosis	Diagnosis	ICD-10-CM
D70.1	Agranulocytosis secondary to cancer chemotherapy	Diagnosis	ICD-10-CM
D70.2	Other drug-induced agranulocytosis	Diagnosis	ICD-10-CM
D70.3	Neutropenia due to infection	Diagnosis	ICD-10-CM
D70.4	Cyclic neutropenia	Diagnosis	ICD-10-CM
D70.8	Other neutropenia	Diagnosis	ICD-10-CM
D70.9	Neutropenia, unspecified	Diagnosis	ICD-10-CM

Code	Description	Code Category	Code Type
D71	Functional disorders of polymorphonuclear neutrophils	Diagnosis	ICD-10-CM
D72.0	Genetic anomalies of leukocytes	Diagnosis	ICD-10-CM
D72.810	Lymphocytopenia	Diagnosis	ICD-10-CM
D72.818	Other decreased white blood cell count	Diagnosis	ICD-10-CM
D72.819	Decreased white blood cell count, unspecified	Diagnosis	ICD-10-CM
D72.825	Bandemia	Diagnosis	ICD-10-CM
D72.828	Other elevated white blood cell count	Diagnosis	ICD-10-CM
D72.829	Elevated white blood cell count, unspecified	Diagnosis	ICD-10-CM
D72.89	Other specified disorders of white blood cells	Diagnosis	ICD-10-CM
D72.9	Disorder of white blood cells, unspecified	Diagnosis	ICD-10-CM
D73.0	Hyposplenism	Diagnosis	ICD-10-CM
D73.1	Hypersplenism	Diagnosis	ICD-10-CM
D73.2	Chronic congestive splenomegaly	Diagnosis	ICD-10-CM
D73.81	Neutropenic splenomegaly	Diagnosis	ICD-10-CM
D73.89	Other diseases of spleen	Diagnosis	ICD-10-CM
D73.9	Disease of spleen, unspecified	Diagnosis	ICD-10-CM
D75.81	Myelofibrosis	Diagnosis	ICD-10-CM
D75.82	Heparin induced thrombocytopenia (HIT)	Diagnosis	ICD-10-CM
D75.89	Other specified diseases of blood and blood-forming organs	Diagnosis	ICD-10-CM
D75.9	Disease of blood and blood-forming organs, unspecified	Diagnosis	ICD-10-CM
D76.1	Hemophagocytic lymphohistiocytosis	Diagnosis	ICD-10-CM
D76.2	Hemophagocytic syndrome, infection-associated	Diagnosis	ICD-10-CM
D76.3	Other histiocytosis syndromes	Diagnosis	ICD-10-CM
D77	Other disorders of blood and blood-forming organs in diseases classified elsewhere	Diagnosis	ICD-10-CM
D89.2	Hypergammaglobulinemia, unspecified	Diagnosis	ICD-10-CM
**Sickle Cell Disease or Thalassemia**			
D57	Sickle-cell disorders	Diagnosis	ICD-10-CM
D57.0	Hb-SS disease with crisis	Diagnosis	ICD-10-CM
D57.00	Hb-SS disease with crisis, unspecified	Diagnosis	ICD-10-CM
D57.01	Hb-SS disease with acute chest syndrome	Diagnosis	ICD-10-CM
D57.02	Hb-SS disease with splenic sequestration	Diagnosis	ICD-10-CM
D57.1	Sickle-cell disease without crisis	Diagnosis	ICD-10-CM
D57.2	Sickle-cell/Hb-C disease	Diagnosis	ICD-10-CM
D57.20	Sickle-cell/Hb-C disease without crisis	Diagnosis	ICD-10-CM
D57.21	Sickle-cell/Hb-C disease with crisis	Diagnosis	ICD-10-CM
D57.211	Sickle-cell/Hb-C disease with acute chest syndrome	Diagnosis	ICD-10-CM
D57.212	Sickle-cell/Hb-C disease with splenic sequestration	Diagnosis	ICD-10-CM
D57.219	Sickle-cell/Hb-C disease with crisis, unspecified	Diagnosis	ICD-10-CM
D57.3	Sickle-cell trait	Diagnosis	ICD-10-CM
D57.4	Sickle-cell thalassemia	Diagnosis	ICD-10-CM
D57.40	Sickle-cell thalassemia without crisis	Diagnosis	ICD-10-CM
D57.41	Sickle-cell thalassemia with crisis	Diagnosis	ICD-10-CM
D57.411	Sickle-cell thalassemia with acute chest syndrome	Diagnosis	ICD-10-CM
D57.412	Sickle-cell thalassemia with splenic sequestration	Diagnosis	ICD-10-CM
D57.419	Sickle-cell thalassemia with crisis, unspecified	Diagnosis	ICD-10-CM

Code	Description	Code Category	Code Type
D57.8	Other sickle-cell disorders	Diagnosis	ICD-10-CM
D57.80	Other sickle-cell disorders without crisis	Diagnosis	ICD-10-CM
D57.81	Other sickle-cell disorders with crisis	Diagnosis	ICD-10-CM
D57.811	Other sickle-cell disorders with acute chest syndrome	Diagnosis	ICD-10-CM
D57.812	Other sickle-cell disorders with splenic sequestration	Diagnosis	ICD-10-CM
D57.819	Other sickle-cell disorders with crisis, unspecified	Diagnosis	ICD-10-CM
D56.0	Alpha thalassemia	Diagnosis	ICD-10-CM
D56.1	Beta thalassemia	Diagnosis	ICD-10-CM
D56.2	Delta-beta thalassemia	Diagnosis	ICD-10-CM
D56.3	Thalassemia minor	Diagnosis	ICD-10-CM
D56.4	Hereditary persistence of fetal hemoglobin [HPFH]	Diagnosis	ICD-10-CM
D56.5	Hemoglobin E-beta thalassemia	Diagnosis	ICD-10-CM
D56.8	Other thalassemias	Diagnosis	ICD-10-CM
D56.9	Thalassemia, unspecified	Diagnosis	ICD-10-CM
**Mental Health Conditions**			
F33.8	Other recurrent depressive disorders	Diagnosis	ICD-10-CM
F43.21	Adjustment disorder with depressed mood	Diagnosis	ICD-10-CM
F34.1	Dysthymic disorder	Diagnosis	ICD-10-CM
F43.23	Adjustment disorder with mixed anxiety and depressed mood	Diagnosis	ICD-10-CM
F33.2	Major depressive disorder, recurrent severe without psychotic features	Diagnosis	ICD-10-CM
F33.42	Major depressive disorder, recurrent, in full remission	Diagnosis	ICD-10-CM
F33.41	Major depressive disorder, recurrent, in partial remission	Diagnosis	ICD-10-CM
F33.40	Major depressive disorder, recurrent, in remission, unspecified	Diagnosis	ICD-10-CM
F33.0	Major depressive disorder, recurrent, mild	Diagnosis	ICD-10-CM
F33.1	Major depressive disorder, recurrent, moderate	Diagnosis	ICD-10-CM
F33.3	Major depressive disorder, recurrent, severe with psychotic symptoms	Diagnosis	ICD-10-CM
F33.9	Major depressive disorder, recurrent, unspecified	Diagnosis	ICD-10-CM
F32.5	Major depressive disorder, single episode, in full remission	Diagnosis	ICD-10-CM
F32.4	Major depressive disorder, single episode, in partial remission	Diagnosis	ICD-10-CM
F32.0	Major depressive disorder, single episode, mild	Diagnosis	ICD-10-CM
F32.1	Major depressive disorder, single episode, moderate	Diagnosis	ICD-10-CM
F32.3	Major depressive disorder, single episode, severe with psychotic features	Diagnosis	ICD-10-CM
F32.2	Major depressive disorder, single episode, severe without psychotic features	Diagnosis	ICD-10-CM
F32.9	Major depressive disorder, single episode, unspecified	Diagnosis	ICD-10-CM
F31.31	Bipolar disorder, current episode depressed, mild	Diagnosis	ICD-10-CM
F31.30	Bipolar disorder, current episode depressed, mild or moderate severity,	Diagnosis	ICD-10-CM
F31.32	Bipolar disorder, current episode depressed, moderate	Diagnosis	ICD-10-CM
F31.5	Bipolar disorder, current episode depressed, severe, with psychotic features	Diagnosis	ICD-10-CM
F31.4	Bipolar disorder, current episode depressed, severe, without psychotic features	Diagnosis	ICD-10-CM
F31.61	Bipolar disorder, current episode mixed, mild	Diagnosis	ICD-10-CM
F31.62	Bipolar disorder, current episode mixed, moderate	Diagnosis	ICD-10-CM
F31.64	Bipolar disorder, current episode mixed, severe, with psychotic features	Diagnosis	ICD-10-CM
F31.63	Bipolar disorder, current episode mixed, severe, without psychotic features	Diagnosis	ICD-10-CM
F31.60	Bipolar disorder, current episode mixed, unspecified	Diagnosis	ICD-10-CM
F31.76	Bipolar disorder, in full remission, most recent episode depressed	Diagnosis	ICD-10-CM

Code	Description	Code Category	Code Type
F31.78	Bipolar disorder, in full remission, most recent episode mixed	Diagnosis	ICD-10-CM
F31.75	Bipolar disorder, in partial remission, most recent episode depressed	Diagnosis	ICD-10-CM
F31.77	Bipolar disorder, in partial remission, most recent episode mixed	Diagnosis	ICD-10-CM
F31.81	Bipolar II disorder	Diagnosis	ICD-10-CM
**Down Syndrome**			
Q90	Down syndrome	Diagnosis	ICD-10-CM
Q90.0	Trisomy 21, nonmosaicism (meiotic nondisjunction)	Diagnosis	ICD-10-CM
Q90.1	Trisomy 21, mosaicism (mitotic nondisjunction)	Diagnosis	ICD-10-CM
Q90.2	Trisomy 21, translocation	Diagnosis	ICD-10-CM
Q90.9	Down syndrome, unspecified	Diagnosis	ICD-10-CM
**Liver and Renal Disorders**			
B18	Chronic viral hepatitis	Diagnosis	ICD-10-CM
B18.0	Chronic viral hepatitis B with delta-agent	Diagnosis	ICD-10-CM
B18.1	Chronic viral hepatitis B without delta-agent	Diagnosis	ICD-10-CM
B18.2	Chronic viral hepatitis C	Diagnosis	ICD-10-CM
B18.8	Other chronic viral hepatitis	Diagnosis	ICD-10-CM
B18.9	Chronic viral hepatitis, unspecified	Diagnosis	ICD-10-CM
I85.0	Esophageal varices	Diagnosis	ICD-10-CM
I85.00	Esophageal varices without bleeding	Diagnosis	ICD-10-CM
I85.01	Esophageal varices with bleeding	Diagnosis	ICD-10-CM
I86.4	Gastric varices	Diagnosis	ICD-10-CM
K70.0	Alcoholic fatty liver	Diagnosis	ICD-10-CM
K70.1	Alcoholic hepatitis	Diagnosis	ICD-10-CM
K70.10	Alcoholic hepatitis without ascites	Diagnosis	ICD-10-CM
K70.11	Alcoholic hepatitis with ascites	Diagnosis	ICD-10-CM
K70.2	Alcoholic fibrosis and sclerosis of liver	Diagnosis	ICD-10-CM
K70.3	Alcoholic cirrhosis of liver	Diagnosis	ICD-10-CM
K70.30	Alcoholic cirrhosis of liver without ascites	Diagnosis	ICD-10-CM
K70.31	Alcoholic cirrhosis of liver with ascites	Diagnosis	ICD-10-CM
K70.4	Alcoholic hepatic failure	Diagnosis	ICD-10-CM
K70.40	Alcoholic hepatic failure without coma	Diagnosis	ICD-10-CM
K70.41	Alcoholic hepatic failure with coma	Diagnosis	ICD-10-CM
K70.9	Alcoholic liver disease, unspecified	Diagnosis	ICD-10-CM
K71.1	Toxic liver disease with hepatic necrosis	Diagnosis	ICD-10-CM
K71.10	Toxic liver disease with hepatic necrosis, without coma	Diagnosis	ICD-10-CM
K71.11	Toxic liver disease with hepatic necrosis, with coma	Diagnosis	ICD-10-CM
K71.3	Toxic liver disease with chronic persistent hepatitis	Diagnosis	ICD-10-CM
K71.4	Toxic liver disease with chronic lobular hepatitis	Diagnosis	ICD-10-CM
K71.5	Toxic liver disease with chronic active hepatitis	Diagnosis	ICD-10-CM
K71.50	Toxic liver disease with chronic active hepatitis without ascites	Diagnosis	ICD-10-CM
K71.51	Toxic liver disease with chronic active hepatitis with ascites	Diagnosis	ICD-10-CM
K71.7	Toxic liver disease with fibrosis and cirrhosis of liver	Diagnosis	ICD-10-CM
K72.1	Chronic hepatic failure	Diagnosis	ICD-10-CM
K72.10	Chronic hepatic failure without coma	Diagnosis	ICD-10-CM
K72.11	Chronic hepatic failure with coma	Diagnosis	ICD-10-CM

Code	Description	Code Category	Code Type
K72.9	Hepatic failure, unspecified	Diagnosis	ICD-10-CM
K72.90	Hepatic failure, unspecified without coma	Diagnosis	ICD-10-CM
K72.91	Hepatic failure, unspecified with coma	Diagnosis	ICD-10-CM
K73	Chronic hepatitis, not elsewhere classified	Diagnosis	ICD-10-CM
K73.0	Chronic persistent hepatitis, not elsewhere classified	Diagnosis	ICD-10-CM
K73.1	Chronic lobular hepatitis, not elsewhere classified	Diagnosis	ICD-10-CM
K73.2	Chronic active hepatitis, not elsewhere classified	Diagnosis	ICD-10-CM
K73.8	Other chronic hepatitis, not elsewhere classified	Diagnosis	ICD-10-CM
K73.9	Chronic hepatitis, unspecified	Diagnosis	ICD-10-CM
K74	Fibrosis and cirrhosis of liver	Diagnosis	ICD-10-CM
K74.0	Hepatic fibrosis	Diagnosis	ICD-10-CM
K74.1	Hepatic sclerosis	Diagnosis	ICD-10-CM
K74.2	Hepatic fibrosis with hepatic sclerosis	Diagnosis	ICD-10-CM
K74.3	Primary biliary cirrhosis	Diagnosis	ICD-10-CM
K74.4	Secondary biliary cirrhosis	Diagnosis	ICD-10-CM
K74.5	Biliary cirrhosis, unspecified	Diagnosis	ICD-10-CM
K74.6	Other and unspecified cirrhosis of liver	Diagnosis	ICD-10-CM
K74.60	Unspecified cirrhosis of liver	Diagnosis	ICD-10-CM
K74.69	Other cirrhosis of liver	Diagnosis	ICD-10-CM
K76.0	Fatty (change of) liver, not elsewhere classified	Diagnosis	ICD-10-CM
K76.2	Central hemorrhagic necrosis of liver	Diagnosis	ICD-10-CM
K76.3	Infarction of liver	Diagnosis	ICD-10-CM
K76.4	Peliosis hepatis	Diagnosis	ICD-10-CM
K76.5	Hepatic veno-occlusive disease	Diagnosis	ICD-10-CM
K76.6	Portal hypertension	Diagnosis	ICD-10-CM
K76.7	Hepatorenal syndrome	Diagnosis	ICD-10-CM
K76.8	Other specified diseases of liver	Diagnosis	ICD-10-CM
K76.81	Hepatopulmonary syndrome	Diagnosis	ICD-10-CM
K76.89	Other specified diseases of liver	Diagnosis	ICD-10-CM
K76.9	Liver disease, unspecified	Diagnosis	ICD-10-CM
Z94.4	Liver transplant status	Diagnosis	ICD-10-CM
K71.0	Toxic liver disease with cholestasis	Diagnosis	ICD-10-CM
K71.2	Toxic liver disease with acute hepatitis	Diagnosis	ICD-10-CM
K71.6	Toxic liver disease with hepatitis, not elsewhere classified	Diagnosis	ICD-10-CM
K71.8	Toxic liver disease with other disorders of liver	Diagnosis	ICD-10-CM
K71.9	Toxic liver disease, unspecified	Diagnosis	ICD-10-CM
K75.0	Abscess of liver	Diagnosis	ICD-10-CM
K75.1	Phlebitis of portal vein	Diagnosis	ICD-10-CM
K75.2	Nonspecific reactive hepatitis	Diagnosis	ICD-10-CM
K75.3	Granulomatous hepatitis, not elsewhere classified	Diagnosis	ICD-10-CM
K75.4	Autoimmune hepatitis	Diagnosis	ICD-10-CM
K75.8	Other specified inflammatory liver diseases	Diagnosis	ICD-10-CM
K75.81	Nonalcoholic steatohepatitis (NASH)	Diagnosis	ICD-10-CM
K75.89	Other specified inflammatory liver diseases	Diagnosis	ICD-10-CM
K75.9	Inflammatory liver disease, unspecified	Diagnosis	ICD-10-CM

Code	Description	Code Category	Code Type
K76.1	Chronic passive congestion of liver	Diagnosis	ICD-10-CM
E83.01	Wilson's disease	Diagnosis	ICD-10-CM
E88.01	Alpha-1-antitrypsin deficiency	Diagnosis	ICD-10-CM
K83.01	Primary sclerosing cholangitis	Diagnosis	ICD-10-CM
E83.110	Hereditary hemochromatosis	Diagnosis	ICD-10-CM
R18	Ascites	Diagnosis	ICD-10-CM
R18.8	Other ascites	Diagnosis	ICD-10-CM
K65.2	Spontaneous bacterial peritonitis	Diagnosis	ICD-10-CM
K72.01	Acute and subacute hepatic failure with coma	Diagnosis	ICD-10-CM
K91.83	Postprocedural hepatorenal syndrome	Diagnosis	ICD-10-CM
I85	Esophageal varices	Diagnosis	ICD-10-CM
I85.1	Secondary esophageal varices	Diagnosis	ICD-10-CM
I85.10	Secondary esophageal varices without bleeding	Diagnosis	ICD-10-CM
I85.11	Secondary esophageal varices with bleeding	Diagnosis	ICD-10-CM
580	Acute glomerulonephritis	Diagnosis	ICD-9-CM
580.0	Acute glomerulonephritis with lesion of proliferative glomerulonephritis	Diagnosis	ICD-9-CM
580.4	Acute glomerulonephritis with lesion of rapidly progressive glomerulonephritis	Diagnosis	ICD-9-CM
580.8	Acute glomerulonephritis with other specified pathological lesion in kidney	Diagnosis	ICD-9-CM
580.81	Acute glomerulonephritis with other specified pathological lesion in kidney in disease classified elsewhere	Diagnosis	ICD-9-CM
580.89	Other acute glomerulonephritis with other specified pathological lesion in kidney	Diagnosis	ICD-9-CM
580.9	Acute glomerulonephritis with unspecified pathological lesion in kidney	Diagnosis	ICD-9-CM
581	Nephrotic syndrome	Diagnosis	ICD-9-CM
581.0	Nephrotic syndrome with lesion of proliferative glomerulonephritis	Diagnosis	ICD-9-CM
581.1	Nephrotic syndrome with lesion of membranous glomerulonephritis	Diagnosis	ICD-9-CM
581.2	Nephrotic syndrome with lesion of membranoproliferative glomerulonephritis	Diagnosis	ICD-9-CM
581.3	Nephrotic syndrome with lesion of minimal change glomerulonephritis	Diagnosis	ICD-9-CM
581.8	Nephrotic syndrome with other specified pathological lesion in kidney	Diagnosis	ICD-9-CM
581.81	Nephrotic syndrome with other specified pathological lesion in kidney in diseases classified elsewhere	Diagnosis	ICD-9-CM
581.89	Other nephrotic syndrome with specified pathological lesion in kidney	Diagnosis	ICD-9-CM
581.9	Nephrotic syndrome with unspecified pathological lesion in kidney	Diagnosis	ICD-9-CM
582	Chronic glomerulonephritis	Diagnosis	ICD-9-CM
582.0	Chronic glomerulonephritis with lesion of proliferative glomerulonephritis	Diagnosis	ICD-9-CM
582.1	Chronic glomerulonephritis with lesion of membranous glomerulonephritis	Diagnosis	ICD-9-CM
582.2	Chronic glomerulonephritis with lesion of membranoproliferative glomerulonephritis	Diagnosis	ICD-9-CM
582.4	Chronic glomerulonephritis with lesion of rapidly progressive glomerulonephritis	Diagnosis	ICD-9-CM
582.8	Chronic glomerulonephritis with other specified pathological lesion in kidney	Diagnosis	ICD-9-CM
582.81	Chronic glomerulonephritis with other specified pathological lesion in kidney in diseases classified elsewhere	Diagnosis	ICD-9-CM
582.89	Other chronic glomerulonephritis with specified pathological lesion in kidney	Diagnosis	ICD-9-CM
582.9	Chronic glomerulonephritis with unspecified pathological lesion in kidney	Diagnosis	ICD-9-CM
583	Nephritis and nephropathy, not specified as acute or chronic	Diagnosis	ICD-9-CM

Code	Description	Code Category	Code Type
583.0	Nephritis and nephropathy, not specified as acute or chronic, with lesion of proliferative glomerulonephritis	Diagnosis	ICD-9-CM
583.1	Nephritis and nephropathy, not specified as acute or chronic, with lesion of membranous glomerulonephritis	Diagnosis	ICD-9-CM
583.2	Nephritis and nephropathy, not specified as acute or chronic, with lesion of membranoproliferative glomerulonephritis	Diagnosis	ICD-9-CM
583.4	Nephritis and nephropathy, not specified as acute or chronic, with lesion of rapidly progressive glomerulonephritis	Diagnosis	ICD-9-CM
583.6	Nephritis and nephropathy, not specified as acute or chronic, with lesion of renal cortical necrosis	Diagnosis	ICD-9-CM
583.7	Nephritis and nephropathy, not specified as acute or chronic, with lesion of renal medullary necrosis	Diagnosis	ICD-9-CM
583.8	Nephritis and nephropathy, not specified as acute or chronic, with other specified pathological lesion in kidney	Diagnosis	ICD-9-CM
583.81	Nephritis and nephropathy, not specified as acute or chronic, with other specified pathological lesion in kidney, in diseases classified elsewhere	Diagnosis	ICD-9-CM
583.89	Other nephritis and nephropathy, not specified as acute or chronic, with specified pathological lesion in kidney	Diagnosis	ICD-9-CM
583.9	Nephritis and nephropathy, not specified as acute or chronic, with unspecified pathological lesion in kidney	Diagnosis	ICD-9-CM
584	Acute kidney failure	Diagnosis	ICD-9-CM
584.5	Acute kidney failure with lesion of tubular necrosis	Diagnosis	ICD-9-CM
584.6	Acute kidney failure with lesion of renal cortical necrosis	Diagnosis	ICD-9-CM
584.7	Acute kidney failure with lesion of medullary [papillary] necrosis	Diagnosis	ICD-9-CM
584.8	Acute kidney failure with other specified pathological lesion in kidney	Diagnosis	ICD-9-CM
584.9	Acute kidney failure, unspecified	Diagnosis	ICD-9-CM
585	Chronic kidney disease (CKD)	Diagnosis	ICD-9-CM
585.1	Chronic kidney disease, Stage I	Diagnosis	ICD-9-CM
585.2	Chronic kidney disease, Stage II (mild)	Diagnosis	ICD-9-CM
585.3	Chronic kidney disease, Stage III (moderate)	Diagnosis	ICD-9-CM
585.4	Chronic kidney disease, Stage IV (severe)	Diagnosis	ICD-9-CM
585.5	Chronic kidney disease, Stage V	Diagnosis	ICD-9-CM
585.6	End stage renal disease	Diagnosis	ICD-9-CM
585.9	Chronic kidney disease, unspecified	Diagnosis	ICD-9-CM
586	Unspecified renal failure	Diagnosis	ICD-9-CM
587	Unspecified renal sclerosis	Diagnosis	ICD-9-CM
588	Disorders resulting from impaired renal function	Diagnosis	ICD-9-CM
588.0	Renal osteodystrophy	Diagnosis	ICD-9-CM
588.1	Nephrogenic diabetes insipidus	Diagnosis	ICD-9-CM
588.8	Other specified disorder resulting from impaired renal function	Diagnosis	ICD-9-CM
588.89	Other specified disorders resulting from impaired renal function	Diagnosis	ICD-9-CM
588.9	Unspecified disorder resulting from impaired renal function	Diagnosis	ICD-9-CM
590	Infections of kidney	Diagnosis	ICD-9-CM
590.0	Chronic pyelonephritis	Diagnosis	ICD-9-CM
590.00	Chronic pyelonephritis without lesion of renal medullary necrosis	Diagnosis	ICD-9-CM

Code	Description	Code Category	Code Type
590.01	Chronic pyelonephritis with lesion of renal medullary necrosis	Diagnosis	ICD-9-CM
590.1	Acute pyelonephritis	Diagnosis	ICD-9-CM
590.10	Acute pyelonephritis without lesion of renal medullary necrosis	Diagnosis	ICD-9-CM
590.11	Acute pyelonephritis with lesion of renal medullary necrosis	Diagnosis	ICD-9-CM
590.2	Renal and perinephric abscess	Diagnosis	ICD-9-CM
590.3	Pyeloureteritis cystica	Diagnosis	ICD-9-CM
590.8	Other pyelonephritis or pyonephrosis, not specified as acute or chronic	Diagnosis	ICD-9-CM
590.80	Unspecified pyelonephritis	Diagnosis	ICD-9-CM
590.81	Pyelitis or pyelonephritis in diseases classified elsewhere	Diagnosis	ICD-9-CM
590.9	Unspecified infection of kidney	Diagnosis	ICD-9-CM
591	Hydronephrosis	Diagnosis	ICD-9-CM
593	Other disorders of kidney and ureter	Diagnosis	ICD-9-CM
593.1	Hypertrophy of kidney	Diagnosis	ICD-9-CM
593.4	Other ureteric obstruction	Diagnosis	ICD-9-CM
593.5	Hydroureter	Diagnosis	ICD-9-CM
593.7	Vesicoureteral reflux	Diagnosis	ICD-9-CM
593.70	Vesicoureteral reflux, unspecified or without reflex nephropathy	Diagnosis	ICD-9-CM
593.71	Vesicoureteral reflux with reflux nephropathy, unilateral	Diagnosis	ICD-9-CM
593.72	Vesicoureteral reflux with reflux nephropathy, bilateral	Diagnosis	ICD-9-CM
593.73	Vesicoureteral reflux with reflux nephropathy, NOS	Diagnosis	ICD-9-CM
593.8	Other specified disorders of kidney and ureter	Diagnosis	ICD-9-CM
593.81	Vascular disorders of kidney	Diagnosis	ICD-9-CM
593.89	Other specified disorder of kidney and ureter	Diagnosis	ICD-9-CM
593.9	Unspecified disorder of kidney and ureter	Diagnosis	ICD-9-CM
599.6	Urinary obstruction	Diagnosis	ICD-9-CM
599.60	Urinary obstruction, unspecified	Diagnosis	ICD-9-CM
599.69	Urinary obstruction, not elsewhere classified	Diagnosis	ICD-9-CM
599.7	Hematuria	Diagnosis	ICD-9-CM
599.70	Hematuria, unspecified	Diagnosis	ICD-9-CM
599.71	Gross hematuria	Diagnosis	ICD-9-CM
599.72	Microscopic hematuria	Diagnosis	ICD-9-CM
E09.21	Drug or chemical induced diabetes mellitus with diabetic nephropathy	Diagnosis	ICD-10-CM
E09.22	Drug or chemical induced diabetes mellitus with diabetic chronic kidney disease	Diagnosis	ICD-10-CM
E09.29	Drug or chemical induced diabetes mellitus with other diabetic kidney complication	Diagnosis	ICD-10-CM
M32.14	Glomerular disease in systemic lupus erythematosus	Diagnosis	ICD-10-CM
M32.15	Tubulo-interstitial nephropathy in systemic lupus erythematosus	Diagnosis	ICD-10-CM
M35.04	Sicca syndrome with tubulo-interstitial nephropathy	Diagnosis	ICD-10-CM
N00.0	Acute nephritic syndrome with minor glomerular abnormality	Diagnosis	ICD-10-CM
N00.1	Acute nephritic syndrome with focal and segmental glomerular lesions	Diagnosis	ICD-10-CM
N00.2	Acute nephritic syndrome with diffuse membranous glomerulonephritis	Diagnosis	ICD-10-CM
N00.3	Acute nephritic syndrome with diffuse mesangial proliferative glomerulonephritis	Diagnosis	ICD-10-CM
N00.4	Acute nephritic syndrome with diffuse endocapillary proliferative glomerulonephritis	Diagnosis	ICD-10-CM
N00.5	Acute nephritic syndrome with diffuse mesangiocapillary glomerulonephritis	Diagnosis	ICD-10-CM
N00.6	Acute nephritic syndrome with dense deposit disease	Diagnosis	ICD-10-CM

Code	Description	Code Category	Code Type
N00.7	Acute nephritic syndrome with diffuse crescentic glomerulonephritis	Diagnosis	ICD-10-CM
N00.8	Acute nephritic syndrome with other morphologic changes	Diagnosis	ICD-10-CM
N00.9	Acute nephritic syndrome with unspecified morphologic changes	Diagnosis	ICD-10-CM
N01.0	Rapidly progressive nephritic syndrome with minor glomerular abnormality	Diagnosis	ICD-10-CM
N01.1	Rapidly progressive nephritic syndrome with focal and segmental glomerular lesions	Diagnosis	ICD-10-CM
N01.2	Rapidly progressive nephritic syndrome with diffuse membranous glomerulonephritis	Diagnosis	ICD-10-CM
N01.3	Rapidly progressive nephritic syndrome with diffuse mesangial proliferative glomerulonephritis	Diagnosis	ICD-10-CM
N01.4	Rapidly progressive nephritic syndrome with diffuse endocapillary proliferative glomerulonephritis	Diagnosis	ICD-10-CM
N01.5	Rapidly progressive nephritic syndrome with diffuse mesangiocapillary glomerulonephritis	Diagnosis	ICD-10-CM
N01.6	Rapidly progressive nephritic syndrome with dense deposit disease	Diagnosis	ICD-10-CM
N01.7	Rapidly progressive nephritic syndrome with diffuse crescentic glomerulonephritis	Diagnosis	ICD-10-CM
N01.8	Rapidly progressive nephritic syndrome with other morphologic changes	Diagnosis	ICD-10-CM
N01.9	Rapidly progressive nephritic syndrome with unspecified morphologic changes	Diagnosis	ICD-10-CM
N02.0	Recurrent and persistent hematuria with minor glomerular abnormality	Diagnosis	ICD-10-CM
N02.1	Recurrent and persistent hematuria with focal and segmental glomerular lesions	Diagnosis	ICD-10-CM
N02.2	Recurrent and persistent hematuria with diffuse membranous glomerulonephritis	Diagnosis	ICD-10-CM
N02.3	Recurrent and persistent hematuria with diffuse mesangial proliferative glomerulonephritis	Diagnosis	ICD-10-CM
N02.4	Recurrent and persistent hematuria with diffuse endocapillary proliferative glomerulonephritis	Diagnosis	ICD-10-CM
N02.5	Recurrent and persistent hematuria with diffuse mesangiocapillary glomerulonephritis	Diagnosis	ICD-10-CM
N02.6	Recurrent and persistent hematuria with dense deposit disease	Diagnosis	ICD-10-CM
N02.7	Recurrent and persistent hematuria with diffuse crescentic glomerulonephritis	Diagnosis	ICD-10-CM
N02.8	Recurrent and persistent hematuria with other morphologic changes	Diagnosis	ICD-10-CM
N02.9	Recurrent and persistent hematuria with unspecified morphologic changes	Diagnosis	ICD-10-CM
N03.0	Chronic nephritic syndrome with minor glomerular abnormality	Diagnosis	ICD-10-CM
N03.1	Chronic nephritic syndrome with focal and segmental glomerular lesions	Diagnosis	ICD-10-CM
N03.2	Chronic nephritic syndrome with diffuse membranous glomerulonephritis	Diagnosis	ICD-10-CM
N03.3	Chronic nephritic syndrome with diffuse mesangial proliferative glomerulonephritis	Diagnosis	ICD-10-CM
N03.4	Chronic nephritic syndrome with diffuse endocapillary proliferative glomerulonephritis	Diagnosis	ICD-10-CM
N03.5	Chronic nephritic syndrome with diffuse mesangiocapillary glomerulonephritis	Diagnosis	ICD-10-CM
N03.6	Chronic nephritic syndrome with dense deposit disease	Diagnosis	ICD-10-CM
N03.7	Chronic nephritic syndrome with diffuse crescentic glomerulonephritis	Diagnosis	ICD-10-CM
N03.8	Chronic nephritic syndrome with other morphologic changes	Diagnosis	ICD-10-CM
N03.9	Chronic nephritic syndrome with unspecified morphologic changes	Diagnosis	ICD-10-CM
N04.0	Nephrotic syndrome with minor glomerular abnormality	Diagnosis	ICD-10-CM
N04.1	Nephrotic syndrome with focal and segmental glomerular lesions	Diagnosis	ICD-10-CM
N04.2	Nephrotic syndrome with diffuse membranous glomerulonephritis	Diagnosis	ICD-10-CM

Code	Description	Code Category	Code Type
N04.3	Nephrotic syndrome with diffuse mesangial proliferative glomerulonephritis	Diagnosis	ICD-10-CM
N04.4	Nephrotic syndrome with diffuse endocapillary proliferative glomerulonephritis	Diagnosis	ICD-10-CM
N04.5	Nephrotic syndrome with diffuse mesangiocapillary glomerulonephritis	Diagnosis	ICD-10-CM
N04.6	Nephrotic syndrome with dense deposit disease	Diagnosis	ICD-10-CM
N04.7	Nephrotic syndrome with diffuse crescentic glomerulonephritis	Diagnosis	ICD-10-CM
N04.8	Nephrotic syndrome with other morphologic changes	Diagnosis	ICD-10-CM
N04.9	Nephrotic syndrome with unspecified morphologic changes	Diagnosis	ICD-10-CM
N05.0	Unspecified nephritic syndrome with minor glomerular abnormality	Diagnosis	ICD-10-CM
N05.1	Unspecified nephritic syndrome with focal and segmental glomerular lesions	Diagnosis	ICD-10-CM
N05.2	Unspecified nephritic syndrome with diffuse membranous glomerulonephritis	Diagnosis	ICD-10-CM
N05.3	Unspecified nephritic syndrome with diffuse mesangial proliferative glomerulonephritis	Diagnosis	ICD-10-CM
N05.4	Unspecified nephritic syndrome with diffuse endocapillary proliferative glomerulonephritis	Diagnosis	ICD-10-CM
N05.5	Unspecified nephritic syndrome with diffuse mesangiocapillary glomerulonephritis	Diagnosis	ICD-10-CM
N05.6	Unspecified nephritic syndrome with dense deposit disease	Diagnosis	ICD-10-CM
N05.7	Unspecified nephritic syndrome with diffuse crescentic glomerulonephritis	Diagnosis	ICD-10-CM
N05.8	Unspecified nephritic syndrome with other morphologic changes	Diagnosis	ICD-10-CM
N05.9	Unspecified nephritic syndrome with unspecified morphologic changes	Diagnosis	ICD-10-CM
N07.0	Hereditary nephropathy, not elsewhere classified with minor glomerular abnormality	Diagnosis	ICD-10-CM
N07.1	Hereditary nephropathy, not elsewhere classified with focal and segmental glomerular lesions	Diagnosis	ICD-10-CM
N07.2	Hereditary nephropathy, not elsewhere classified with diffuse membranous glomerulonephritis	Diagnosis	ICD-10-CM
N07.3	Hereditary nephropathy, not elsewhere classified with diffuse mesangial proliferative glomerulonephritis	Diagnosis	ICD-10-CM
N07.4	Hereditary nephropathy, not elsewhere classified with diffuse endocapillary proliferative glomerulonephritis	Diagnosis	ICD-10-CM
N07.5	Hereditary nephropathy, not elsewhere classified with diffuse mesangiocapillary glomerulonephritis	Diagnosis	ICD-10-CM
N07.6	Hereditary nephropathy, not elsewhere classified with dense deposit disease	Diagnosis	ICD-10-CM
N07.7	Hereditary nephropathy, not elsewhere classified with diffuse crescentic glomerulonephritis	Diagnosis	ICD-10-CM
N07.8	Hereditary nephropathy, not elsewhere classified with other morphologic lesions	Diagnosis	ICD-10-CM
N07.9	Hereditary nephropathy, not elsewhere classified with unspecified morphologic lesions	Diagnosis	ICD-10-CM
N08	Glomerular disorders in diseases classified elsewhere	Diagnosis	ICD-10-CM
N10	Acute pyelonephritis	Diagnosis	ICD-10-CM
N11.0	Nonobstructive reflux-associated chronic pyelonephritis	Diagnosis	ICD-10-CM
N11.1	Chronic obstructive pyelonephritis	Diagnosis	ICD-10-CM
N11.8	Other chronic tubulo-interstitial nephritis	Diagnosis	ICD-10-CM
N11.9	Chronic tubulo-interstitial nephritis, unspecified	Diagnosis	ICD-10-CM
N12	Tubulo-interstitial nephritis, not specified as acute or chronic	Diagnosis	ICD-10-CM
N13.0	Hydronephrosis with ureteropelvic junction obstruction	Diagnosis	ICD-10-CM

Code	Description	Code Category	Code Type
N13.1	Hydronephrosis with ureteral stricture, not elsewhere classified	Diagnosis	ICD-10-CM
N13.2	Hydronephrosis with renal and ureteral calculous obstruction	Diagnosis	ICD-10-CM
N13.30	Unspecified hydronephrosis	Diagnosis	ICD-10-CM
N13.39	Other hydronephrosis	Diagnosis	ICD-10-CM
N13.4	Hydroureter	Diagnosis	ICD-10-CM
N13.5	Crossing vessel and stricture of ureter without hydronephrosis	Diagnosis	ICD-10-CM
N13.6	Pyonephrosis	Diagnosis	ICD-10-CM
N13.70	Vesicoureteral-reflux, unspecified	Diagnosis	ICD-10-CM
N13.71	Vesicoureteral-reflux without reflux nephropathy	Diagnosis	ICD-10-CM
N13.721	Vesicoureteral-reflux with reflux nephropathy without hydroureter, unilateral	Diagnosis	ICD-10-CM
N13.722	Vesicoureteral-reflux with reflux nephropathy without hydroureter, bilateral	Diagnosis	ICD-10-CM
N13.729	Vesicoureteral-reflux with reflux nephropathy without hydroureter, unspecified	Diagnosis	ICD-10-CM
N13.731	Vesicoureteral-reflux with reflux nephropathy with hydroureter, unilateral	Diagnosis	ICD-10-CM
N13.732	Vesicoureteral-reflux with reflux nephropathy with hydroureter, bilateral	Diagnosis	ICD-10-CM
N13.739	Vesicoureteral-reflux with reflux nephropathy with hydroureter, unspecified	Diagnosis	ICD-10-CM
N13.8	Other obstructive and reflux uropathy	Diagnosis	ICD-10-CM
N13.9	Obstructive and reflux uropathy, unspecified	Diagnosis	ICD-10-CM
N14.0	Analgesic nephropathy	Diagnosis	ICD-10-CM
N14.1	Nephropathy induced by other drugs, medicaments and biological substances	Diagnosis	ICD-10-CM
N14.2	Nephropathy induced by unspecified drug, medicament or biological substance	Diagnosis	ICD-10-CM
N14.3	Nephropathy induced by heavy metals	Diagnosis	ICD-10-CM
N14.4	Toxic nephropathy, not elsewhere classified	Diagnosis	ICD-10-CM
N15.0	Balkan nephropathy	Diagnosis	ICD-10-CM
N15.1	Renal and perinephric abscess	Diagnosis	ICD-10-CM
N15.8	Other specified renal tubulo-interstitial diseases	Diagnosis	ICD-10-CM
N15.9	Renal tubulo-interstitial disease, unspecified	Diagnosis	ICD-10-CM
N16	Renal tubulo-interstitial disorders in diseases classified elsewhere	Diagnosis	ICD-10-CM
N17.0	Acute kidney failure with tubular necrosis	Diagnosis	ICD-10-CM
N17.1	Acute kidney failure with acute cortical necrosis	Diagnosis	ICD-10-CM
N17.2	Acute kidney failure with medullary necrosis	Diagnosis	ICD-10-CM
N17.8	Other acute kidney failure	Diagnosis	ICD-10-CM
N17.9	Acute kidney failure, unspecified	Diagnosis	ICD-10-CM
N18.1	Chronic kidney disease, stage 1	Diagnosis	ICD-10-CM
N18.2	Chronic kidney disease, stage 2 (mild)	Diagnosis	ICD-10-CM
N18.3	Chronic kidney disease, stage 3 (moderate)	Diagnosis	ICD-10-CM
N18.4	Chronic kidney disease, stage 4 (severe)	Diagnosis	ICD-10-CM
N18.5	Chronic kidney disease, stage 5	Diagnosis	ICD-10-CM
N18.6	End stage renal disease	Diagnosis	ICD-10-CM
N18.9	Chronic kidney disease, unspecified	Diagnosis	ICD-10-CM
N19	Unspecified kidney failure	Diagnosis	ICD-10-CM
N25.89	Other disorders resulting from impaired renal tubular function	Diagnosis	ICD-10-CM
N25.9	Disorder resulting from impaired renal tubular function, unspecified	Diagnosis	ICD-10-CM
N28.89	Other specified disorders of kidney and ureter	Diagnosis	ICD-10-CM
N28.9	Disorder of kidney and ureter, unspecified	Diagnosis	ICD-10-CM
N29	Other disorders of kidney and ureter in diseases classified elsewhere	Diagnosis	ICD-10-CM

Code	Description	Code Category	Code Type
	**Obesity**		
278.00	Obesity, unspecified	Diagnosis	ICD-9-CM
278.01	Morbid obesity	Diagnosis	ICD-9-CM
539.0	Complications of gastric band procedure	Diagnosis	ICD-9-CM
539.01	Infection due to gastric band procedure	Diagnosis	ICD-9-CM
539.09	Other complications of gastric band procedure	Diagnosis	ICD-9-CM
539.8	Complications of other bariatric procedure	Diagnosis	ICD-9-CM
539.81	Infection due to other bariatric procedure	Diagnosis	ICD-9-CM
539.89	Other complications of other bariatric procedure	Diagnosis	ICD-9-CM
649.1	Obesity complicating pregnancy, childbirth, or the puerperium	Diagnosis	ICD-9-CM
649.10	Obesity complicating pregnancy, childbirth, or the puerperium, unspecified as to episode of care or not applicable	Diagnosis	ICD-9-CM
649.11	Obesity complicating pregnancy, childbirth, or the puerperium, delivered, with or without mention of antepartum condition	Diagnosis	ICD-9-CM
649.12	Obesity complicating pregnancy, childbirth, or the puerperium, delivered, with mention of postpartum complication	Diagnosis	ICD-9-CM
649.13	Obesity complicating pregnancy, childbirth, or the puerperium, antepartum condition or complication	Diagnosis	ICD-9-CM
649.14	Obesity complicating pregnancy, childbirth, or the puerperium, postpartum condition or complication	Diagnosis	ICD-9-CM
649.2	Bariatric surgery status complicating pregnancy, childbirth, or the puerperium	Diagnosis	ICD-9-CM
649.20	Bariatric surgery status complicating pregnancy, childbirth, or the puerperium, unspecified as to episode of care or not applicable	Diagnosis	ICD-9-CM
649.21	Bariatric surgery status complicating pregnancy, childbirth, or the puerperium, delivered, with or without mention of antepartum condition	Diagnosis	ICD-9-CM
649.22	Bariatric surgery status complicating pregnancy, childbirth, or the puerperium, delivered, with mention of postpartum complication	Diagnosis	ICD-9-CM
649.23	Bariatric surgery status complicating pregnancy, childbirth, or the puerperium, antepartum condition or complication	Diagnosis	ICD-9-CM
649.24	Bariatric surgery status complicating pregnancy, childbirth, or the puerperium, postpartum condition or complication	Diagnosis	ICD-9-CM
V85.3	Body Mass Index between 30-39, adult	Diagnosis	ICD-9-CM
V85.30	Body Mass Index 30.0-30.9, adult	Diagnosis	ICD-9-CM
V85.31	Body Mass Index 31.0-31.9, adult	Diagnosis	ICD-9-CM
V85.32	Body Mass Index 32.0-32.9, adult	Diagnosis	ICD-9-CM
V85.33	Body Mass Index 33.0-33.9, adult	Diagnosis	ICD-9-CM
V85.34	Body Mass Index 34.0-34.9, adult	Diagnosis	ICD-9-CM
V85.35	Body Mass Index 35.0-35.9, adult	Diagnosis	ICD-9-CM
V85.36	Body Mass Index 36.0-36.9, adult	Diagnosis	ICD-9-CM
V85.37	Body Mass Index 37.0-37.9, adult	Diagnosis	ICD-9-CM
V85.38	Body Mass Index 38.0-38.9, adult	Diagnosis	ICD-9-CM
V85.39	Body Mass Index 39.0-39.9, adult	Diagnosis	ICD-9-CM
V85.4	Body Mass Index 40 and over, adult	Diagnosis	ICD-9-CM
V85.41	Body Mass Index 40.0-44.9, adult	Diagnosis	ICD-9-CM
V85.42	Body Mass Index 45.0-49.9, adult	Diagnosis	ICD-9-CM

Code	Description	Code Category	Code Type
V85.43	Body Mass Index 50.0-59.9, adult	Diagnosis	ICD-9-CM
V85.44	Body Mass Index 60.0-69.9, adult	Diagnosis	ICD-9-CM
V85.45	Body Mass Index 70 and over, adult	Diagnosis	ICD-9-CM
E66.01	Morbid (severe) obesity due to excess calories	Diagnosis	ICD-10-CM
E66.09	Other obesity due to excess calories	Diagnosis	ICD-10-CM
E66.1	Drug-induced obesity	Diagnosis	ICD-10-CM
E66.8	Other obesity	Diagnosis	ICD-10-CM
E66.9	Obesity, unspecified	Diagnosis	ICD-10-CM
K95.01	Infection due to gastric band procedure	Diagnosis	ICD-10-CM
K95.09	Other complications of gastric band procedure	Diagnosis	ICD-10-CM
K95.81	Infection due to other bariatric procedure	Diagnosis	ICD-10-CM
K95.89	Other complications of other bariatric procedure	Diagnosis	ICD-10-CM
O99.210	Obesity complicating pregnancy, unspecified trimester	Diagnosis	ICD-10-CM
O99.211	Obesity complicating pregnancy, first trimester	Diagnosis	ICD-10-CM
O99.212	Obesity complicating pregnancy, second trimester	Diagnosis	ICD-10-CM
O99.213	Obesity complicating pregnancy, third trimester	Diagnosis	ICD-10-CM
O99.214	Obesity complicating childbirth	Diagnosis	ICD-10-CM
O99.215	Obesity complicating the puerperium	Diagnosis	ICD-10-CM
O99.840	Bariatric surgery status complicating pregnancy, unspecified trimester	Diagnosis	ICD-10-CM
O99.841	Bariatric surgery status complicating pregnancy, first trimester	Diagnosis	ICD-10-CM
O99.842	Bariatric surgery status complicating pregnancy, second trimester	Diagnosis	ICD-10-CM
O99.843	Bariatric surgery status complicating pregnancy, third trimester	Diagnosis	ICD-10-CM
O99.844	Bariatric surgery status complicating childbirth	Diagnosis	ICD-10-CM
O99.845	Bariatric surgery status complicating the puerperium	Diagnosis	ICD-10-CM
Z68.30	Body mass index (BMI) 30.0-30.9, adult	Diagnosis	ICD-10-CM
Z68.31	Body mass index (BMI) 31.0-31.9, adult	Diagnosis	ICD-10-CM
Z68.32	Body mass index (BMI) 32.0-32.9, adult	Diagnosis	ICD-10-CM
Z68.33	Body mass index (BMI) 33.0-33.9, adult	Diagnosis	ICD-10-CM
Z68.34	Body mass index (BMI) 34.0-34.9, adult	Diagnosis	ICD-10-CM
Z68.35	Body mass index (BMI) 35.0-35.9, adult	Diagnosis	ICD-10-CM
Z68.36	Body mass index (BMI) 36.0-36.9, adult	Diagnosis	ICD-10-CM
Z68.37	Body mass index (BMI) 37.0-37.9, adult	Diagnosis	ICD-10-CM
Z68.38	Body mass index (BMI) 38.0-38.9, adult	Diagnosis	ICD-10-CM
Z68.39	Body mass index (BMI) 39.0-39.9, adult	Diagnosis	ICD-10-CM
Z68.41	Body mass index (BMI) 40.0-44.9, adult	Diagnosis	ICD-10-CM
Z68.42	Body mass index (BMI) 45.0-49.9, adult	Diagnosis	ICD-10-CM
Z68.43	Body mass index (BMI) 50-59.9, adult	Diagnosis	ICD-10-CM
Z68.44	Body mass index (BMI) 60.0-69.9, adult	Diagnosis	ICD-10-CM
Z68.45	Body mass index (BMI) 70 or greater, adult	Diagnosis	ICD-10-CM
278.03	Obesity hypoventilation syndrome	Diagnosis	ICD-10-CM
E66.2	Morbid (severe) obesity with alveolar hypoventilation	Diagnosis	ICD-10-CM
43.89	Open and other partial gastrectomy	Procedure	ICD-9-PCS
44.38	Laparoscopic gastroenterostomy	Procedure	ICD-9-PCS
44.39	Other gastroenterostomy without gastrectomy	Procedure	ICD-9-PCS
44.68	Laparoscopic gastroplasty	Procedure	ICD-9-PCS

Code	Description	Code Category	Code Type
44.95	Laparoscopic gastric restrictive procedure	Procedure	ICD-9-PCS
0D16079	Bypass Stomach to Duodenum with Autologous Tissue Substitute, Open Approach	Procedure	ICD-10-PCS
0D1607A	Bypass Stomach to Jejunum with Autologous Tissue Substitute, Open Approach	Procedure	ICD-10-PCS
0D1607B	Bypass Stomach to Ileum with Autologous Tissue Substitute, Open Approach	Procedure	ICD-10-PCS
0D1607L	Bypass Stomach to Transverse Colon with Autologous Tissue Substitute, Open Approach	Procedure	ICD-10-PCS
0D160J9	Bypass Stomach to Duodenum with Synthetic Substitute, Open Approach	Procedure	ICD-10-PCS
0D160JA	Bypass Stomach to Jejunum with Synthetic Substitute, Open Approach	Procedure	ICD-10-PCS
0D160JB	Bypass Stomach to Ileum with Synthetic Substitute, Open Approach	Procedure	ICD-10-PCS
0D160JL	Bypass Stomach to Transverse Colon with Synthetic Substitute, Open Approach	Procedure	ICD-10-PCS
0D160K9	Bypass Stomach to Duodenum with Nonautologous Tissue Substitute, Open Approach	Procedure	ICD-10-PCS
0D160KA	Bypass Stomach to Jejunum with Nonautologous Tissue Substitute, Open Approach	Procedure	ICD-10-PCS
0D160KB	Bypass Stomach to Ileum with Nonautologous Tissue Substitute, Open Approach	Procedure	ICD-10-PCS
0D160KL	Bypass Stomach to Transverse Colon with Nonautologous Tissue Substitute, Open Approach	Procedure	ICD-10-PCS
0D160Z9	Bypass Stomach to Duodenum, Open Approach	Procedure	ICD-10-PCS
0D160ZA	Bypass Stomach to Jejunum, Open Approach	Procedure	ICD-10-PCS
0D160ZB	Bypass Stomach to Ileum, Open Approach	Procedure	ICD-10-PCS
0D160ZL	Bypass Stomach to Transverse Colon, Open Approach	Procedure	ICD-10-PCS
0D16479	Bypass Stomach to Duodenum with Autologous Tissue Substitute, Percutaneous Endoscopic Approach	Procedure	ICD-10-PCS
0D1647A	Bypass Stomach to Jejunum with Autologous Tissue Substitute, Percutaneous Endoscopic Approach	Procedure	ICD-10-PCS
0D1647B	Bypass Stomach to Ileum with Autologous Tissue Substitute, Percutaneous Endoscopic Approach	Procedure	ICD-10-PCS
0D1647L	Bypass Stomach to Transverse Colon with Autologous Tissue Substitute, Percutaneous Endoscopic Approach	Procedure	ICD-10-PCS
0D164J9	Bypass Stomach to Duodenum with Synthetic Substitute, Percutaneous Endoscopic Approach	Procedure	ICD-10-PCS
0D164JA	Bypass Stomach to Jejunum with Synthetic Substitute, Percutaneous Endoscopic Approach	Procedure	ICD-10-PCS
0D164JB	Bypass Stomach to Ileum with Synthetic Substitute, Percutaneous Endoscopic Approach	Procedure	ICD-10-PCS
0D164JL	Bypass Stomach to Transverse Colon with Synthetic Substitute, Percutaneous Endoscopic Approach	Procedure	ICD-10-PCS
0D164K9	Bypass Stomach to Duodenum with Nonautologous Tissue Substitute, Percutaneous Endoscopic Approach	Procedure	ICD-10-PCS
0D164KA	Bypass Stomach to Jejunum with Nonautologous Tissue Substitute, Percutaneous Endoscopic Approach	Procedure	ICD-10-PCS
0D164KB	Bypass Stomach to Ileum with Nonautologous Tissue Substitute, Percutaneous Endoscopic Approach	Procedure	ICD-10-PCS
0D164KL	Bypass Stomach to Transverse Colon with Nonautologous Tissue Substitute, Percutaneous Endoscopic Approach	Procedure	ICD-10-PCS

Code	Description	Code Category	Code Type
0D164Z9	Bypass Stomach to Duodenum, Percutaneous Endoscopic Approach	Procedure	ICD-10-PCS
0D164ZA	Bypass Stomach to Jejunum, Percutaneous Endoscopic Approach	Procedure	ICD-10-PCS
0D164ZB	Bypass Stomach to Ileum, Percutaneous Endoscopic Approach	Procedure	ICD-10-PCS
0D164ZL	Bypass Stomach to Transverse Colon, Percutaneous Endoscopic Approach	Procedure	ICD-10-PCS
0D16879	Bypass Stomach to Duodenum with Autologous Tissue Substitute, Via Natural or Artificial Opening Endoscopic	Procedure	ICD-10-PCS
0D1687A	Bypass Stomach to Jejunum with Autologous Tissue Substitute, Via Natural or Artificial Opening Endoscopic	Procedure	ICD-10-PCS
0D1687B	Bypass Stomach to Ileum with Autologous Tissue Substitute, Via Natural or Artificial Opening Endoscopic	Procedure	ICD-10-PCS
0D1687L	Bypass Stomach to Transverse Colon with Autologous Tissue Substitute, Via Natural or Artificial Opening Endoscopic	Procedure	ICD-10-PCS
0D168J9	Bypass Stomach to Duodenum with Synthetic Substitute, Via Natural or Artificial Opening Endoscopic	Procedure	ICD-10-PCS
0D168JA	Bypass Stomach to Jejunum with Synthetic Substitute, Via Natural or Artificial Opening Endoscopic	Procedure	ICD-10-PCS
0D168JB	Bypass Stomach to Ileum with Synthetic Substitute, Via Natural or Artificial Opening Endoscopic	Procedure	ICD-10-PCS
0D168JL	Bypass Stomach to Transverse Colon with Synthetic Substitute, Via Natural or Artificial Opening Endoscopic	Procedure	ICD-10-PCS
0D168K9	Bypass Stomach to Duodenum with Nonautologous Tissue Substitute, Via Natural or Artificial Opening Endoscopic	Procedure	ICD-10-PCS
0D168KA	Bypass Stomach to Jejunum with Nonautologous Tissue Substitute, Via Natural or Artificial Opening Endoscopic	Procedure	ICD-10-PCS
0D168KB	Bypass Stomach to Ileum with Nonautologous Tissue Substitute, Via Natural or Artificial Opening Endoscopic	Procedure	ICD-10-PCS
0D168KL	Bypass Stomach to Transverse Colon with Nonautologous Tissue Substitute, Via Natural or Artificial Opening Endoscopic	Procedure	ICD-10-PCS
0D168Z9	Bypass Stomach to Duodenum, Via Natural or Artificial Opening Endoscopic	Procedure	ICD-10-PCS
0D168ZA	Bypass Stomach to Jejunum, Via Natural or Artificial Opening Endoscopic	Procedure	ICD-10-PCS
0D168ZB	Bypass Stomach to Ileum, Via Natural or Artificial Opening Endoscopic	Procedure	ICD-10-PCS
0D168ZL	Bypass Stomach to Transverse Colon, Via Natural or Artificial Opening Endoscopic	Procedure	ICD-10-PCS
0D19079	Bypass Duodenum to Duodenum with Autologous Tissue Substitute, Open Approach	Procedure	ICD-10-PCS
0D1907A	Bypass Duodenum to Jejunum with Autologous Tissue Substitute, Open Approach	Procedure	ICD-10-PCS
0D1907B	Bypass Duodenum to Ileum with Autologous Tissue Substitute, Open Approach	Procedure	ICD-10-PCS
0D190J9	Bypass Duodenum to Duodenum with Synthetic Substitute, Open Approach	Procedure	ICD-10-PCS
0D190JA	Bypass Duodenum to Jejunum with Synthetic Substitute, Open Approach	Procedure	ICD-10-PCS
0D190JB	Bypass Duodenum to Ileum with Synthetic Substitute, Open Approach	Procedure	ICD-10-PCS
0D190K9	Bypass Duodenum to Duodenum with Nonautologous Tissue Substitute, Open Approach	Procedure	ICD-10-PCS
0D190KA	Bypass Duodenum to Jejunum with Nonautologous Tissue Substitute, Open Approach	Procedure	ICD-10-PCS

Code	Description	Code Category	Code Type
0D190KB	Bypass Duodenum to Ileum with Nonautologous Tissue Substitute, Open Approach	Procedure	ICD-10-PCS
0D190Z9	Bypass Duodenum to Duodenum, Open Approach	Procedure	ICD-10-PCS
0D190ZA	Bypass Duodenum to Jejunum, Open Approach	Procedure	ICD-10-PCS
0D190ZB	Bypass Duodenum to Ileum, Open Approach	Procedure	ICD-10-PCS
0D19479	Bypass Duodenum to Duodenum with Autologous Tissue Substitute, Percutaneous Endoscopic Approach	Procedure	ICD-10-PCS
0D1947A	Bypass Duodenum to Jejunum with Autologous Tissue Substitute, Percutaneous Endoscopic Approach	Procedure	ICD-10-PCS
0D1947B	Bypass Duodenum to Ileum with Autologous Tissue Substitute, Percutaneous Endoscopic Approach	Procedure	ICD-10-PCS
0D194J9	Bypass Duodenum to Duodenum with Synthetic Substitute, Percutaneous Endoscopic Approach	Procedure	ICD-10-PCS
0D194JA	Bypass Duodenum to Jejunum with Synthetic Substitute, Percutaneous Endoscopic Approach	Procedure	ICD-10-PCS
0D194JB	Bypass Duodenum to Ileum with Synthetic Substitute, Percutaneous Endoscopic Approach	Procedure	ICD-10-PCS
0D194K9	Bypass Duodenum to Duodenum with Nonautologous Tissue Substitute, Percutaneous Endoscopic Approach	Procedure	ICD-10-PCS
0D194KA	Bypass Duodenum to Jejunum with Nonautologous Tissue Substitute, Percutaneous Endoscopic Approach	Procedure	ICD-10-PCS
0D194KB	Bypass Duodenum to Ileum with Nonautologous Tissue Substitute, Percutaneous Endoscopic Approach	Procedure	ICD-10-PCS
0D194Z9	Bypass Duodenum to Duodenum, Percutaneous Endoscopic Approach	Procedure	ICD-10-PCS
0D194ZA	Bypass Duodenum to Jejunum, Percutaneous Endoscopic Approach	Procedure	ICD-10-PCS
0D194ZB	Bypass Duodenum to Ileum, Percutaneous Endoscopic Approach	Procedure	ICD-10-PCS
0D19879	Bypass Duodenum to Duodenum with Autologous Tissue Substitute, Via Natural or Artificial Opening Endoscopic	Procedure	ICD-10-PCS
0D1987A	Bypass Duodenum to Jejunum with Autologous Tissue Substitute, Via Natural or Artificial Opening Endoscopic	Procedure	ICD-10-PCS
0D1987B	Bypass Duodenum to Ileum with Autologous Tissue Substitute, Via Natural or Artificial Opening Endoscopic	Procedure	ICD-10-PCS
0D198J9	Bypass Duodenum to Duodenum with Synthetic Substitute, Via Natural or Artificial Opening Endoscopic	Procedure	ICD-10-PCS
0D198JA	Bypass Duodenum to Jejunum with Synthetic Substitute, Via Natural or Artificial Opening Endoscopic	Procedure	ICD-10-PCS
0D198JB	Bypass Duodenum to Ileum with Synthetic Substitute, Via Natural or Artificial Opening Endoscopic	Procedure	ICD-10-PCS
0D198K9	Bypass Duodenum to Duodenum with Nonautologous Tissue Substitute, Via Natural or Artificial Opening Endoscopic	Procedure	ICD-10-PCS
0D198KA	Bypass Duodenum to Jejunum with Nonautologous Tissue Substitute, Via Natural or Artificial Opening Endoscopic	Procedure	ICD-10-PCS
0D198KB	Bypass Duodenum to Ileum with Nonautologous Tissue Substitute, Via Natural or Artificial Opening Endoscopic	Procedure	ICD-10-PCS
0D198Z9	Bypass Duodenum to Duodenum, Via Natural or Artificial Opening Endoscopic	Procedure	ICD-10-PCS

Code	Description	Code Category	Code Type
0D198ZA	Bypass Duodenum to Jejunum, Via Natural or Artificial Opening Endoscopic	Procedure	ICD-10-PCS
0D198ZB	Bypass Duodenum to Ileum, Via Natural or Artificial Opening Endoscopic	Procedure	ICD-10-PCS
0D1A07A	Bypass Jejunum to Jejunum with Autologous Tissue Substitute, Open Approach	Procedure	ICD-10-PCS
0D1A07B	Bypass Jejunum to Ileum with Autologous Tissue Substitute, Open Approach	Procedure	ICD-10-PCS
0D1A0JA	Bypass Jejunum to Jejunum with Synthetic Substitute, Open Approach	Procedure	ICD-10-PCS
0D1A0JB	Bypass Jejunum to Ileum with Synthetic Substitute, Open Approach	Procedure	ICD-10-PCS
0D1A0KA	Bypass Jejunum to Jejunum with Nonautologous Tissue Substitute, Open Approach	Procedure	ICD-10-PCS
0D1A0KB	Bypass Jejunum to Ileum with Nonautologous Tissue Substitute, Open Approach	Procedure	ICD-10-PCS
0D1A0ZA	Bypass Jejunum to Jejunum, Open Approach	Procedure	ICD-10-PCS
0D1A0ZB	Bypass Jejunum to Ileum, Open Approach	Procedure	ICD-10-PCS
0D1A47A	Bypass Jejunum to Jejunum with Autologous Tissue Substitute, Percutaneous Endoscopic Approach	Procedure	ICD-10-PCS
0D1A47B	Bypass Jejunum to Ileum with Autologous Tissue Substitute, Percutaneous Endoscopic Approach	Procedure	ICD-10-PCS
0D1A4JA	Bypass Jejunum to Jejunum with Synthetic Substitute, Percutaneous Endoscopic Approach	Procedure	ICD-10-PCS
0D1A4JB	Bypass Jejunum to Ileum with Synthetic Substitute, Percutaneous Endoscopic Approach	Procedure	ICD-10-PCS
0D1A4KA	Bypass Jejunum to Jejunum with Nonautologous Tissue Substitute, Percutaneous Endoscopic Approach	Procedure	ICD-10-PCS
0D1A4KB	Bypass Jejunum to Ileum with Nonautologous Tissue Substitute, Percutaneous Endoscopic Approach	Procedure	ICD-10-PCS
0D1A4ZA	Bypass Jejunum to Jejunum, Percutaneous Endoscopic Approach	Procedure	ICD-10-PCS
0D1A4ZB	Bypass Jejunum to Ileum, Percutaneous Endoscopic Approach	Procedure	ICD-10-PCS
0D1A87A	Bypass Jejunum to Jejunum with Autologous Tissue Substitute, Via Natural or Artificial Opening Endoscopic	Procedure	ICD-10-PCS
0D1A87B	Bypass Jejunum to Ileum with Autologous Tissue Substitute, Via Natural or Artificial Opening Endoscopic	Procedure	ICD-10-PCS
0D1A8JA	Bypass Jejunum to Jejunum with Synthetic Substitute, Via Natural or Artificial Opening Endoscopic	Procedure	ICD-10-PCS
0D1A8JB	Bypass Jejunum to Ileum with Synthetic Substitute, Via Natural or Artificial Opening Endoscopic	Procedure	ICD-10-PCS
0D1A8KA	Bypass Jejunum to Jejunum with Nonautologous Tissue Substitute, Via Natural or Artificial Opening Endoscopic	Procedure	ICD-10-PCS
0D1A8KB	Bypass Jejunum to Ileum with Nonautologous Tissue Substitute, Via Natural or Artificial Opening Endoscopic	Procedure	ICD-10-PCS
0D1A8ZA	Bypass Jejunum to Jejunum, Via Natural or Artificial Opening Endoscopic	Procedure	ICD-10-PCS
0D1A8ZB	Bypass Jejunum to Ileum, Via Natural or Artificial Opening Endoscopic	Procedure	ICD-10-PCS
0D1A8ZH	Bypass Jejunum to Cecum, Via Natural or Artificial Opening Endoscopic	Procedure	ICD-10-PCS
0D1B07B	Bypass Ileum to Ileum with Autologous Tissue Substitute, Open Approach	Procedure	ICD-10-PCS
0D1B0JB	Bypass Ileum to Ileum with Synthetic Substitute, Open Approach	Procedure	ICD-10-PCS
0D1B0KB	Bypass Ileum to Ileum with Nonautologous Tissue Substitute, Open Approach	Procedure	ICD-10-PCS
0D1B0ZB	Bypass Ileum to Ileum, Open Approach	Procedure	ICD-10-PCS
0D1B47B	Bypass Ileum to Ileum with Autologous Tissue Substitute, Percutaneous Endoscopic Approach	Procedure	ICD-10-PCS

Code	Description	Code Category	Code Type
0D1B4JB	Bypass Ileum to Ileum with Synthetic Substitute, Percutaneous Endoscopic Approach	Procedure	ICD-10-PCS
0D1B4KB	Bypass Ileum to Ileum with Nonautologous Tissue Substitute, Percutaneous Endoscopic Approach	Procedure	ICD-10-PCS
0D1B4ZB	Bypass Ileum to Ileum, Percutaneous Endoscopic Approach	Procedure	ICD-10-PCS
0D1B87B	Bypass Ileum to Ileum with Autologous Tissue Substitute, Via Natural or Artificial Opening Endoscopic	Procedure	ICD-10-PCS
0D1B8JB	Bypass Ileum to Ileum with Synthetic Substitute, Via Natural or Artificial Opening Endoscopic	Procedure	ICD-10-PCS
0D1B8KB	Bypass Ileum to Ileum with Nonautologous Tissue Substitute, Via Natural or Artificial Opening Endoscopic	Procedure	ICD-10-PCS
0D1B8ZB	Bypass Ileum to Ileum, Via Natural or Artificial Opening Endoscopic	Procedure	ICD-10-PCS
0D1B8ZH	Bypass Ileum to Cecum, Via Natural or Artificial Opening Endoscopic	Procedure	ICD-10-PCS
0DB60Z3	Excision of Stomach, Open Approach, Vertical	Procedure	ICD-10-PCS
0DB60ZZ	Excision of Stomach, Open Approach	Procedure	ICD-10-PCS
0DB63Z3	Excision of Stomach, Percutaneous Approach, Vertical	Procedure	ICD-10-PCS
0DB63ZZ	Excision of Stomach, Percutaneous Approach	Procedure	ICD-10-PCS
0DB67Z3	Excision of Stomach, Via Natural or Artificial Opening, Vertical	Procedure	ICD-10-PCS
0DB67ZZ	Excision of Stomach, Via Natural or Artificial Opening	Procedure	ICD-10-PCS
0DB68Z3	Excision of Stomach, Via Natural or Artificial Opening Endoscopic, Vertical	Procedure	ICD-10-PCS
0DB80ZZ	Excision of Small Intestine, Open Approach	Procedure	ICD-10-PCS
0DB90ZZ	Excision of Duodenum, Open Approach	Procedure	ICD-10-PCS
0DBB0ZZ	Excision of Ileum, Open Approach	Procedure	ICD-10-PCS
0DQ64ZZ	Repair Stomach, Percutaneous Endoscopic Approach	Procedure	ICD-10-PCS
0DV64CZ	Restriction of Stomach with Extraluminal Device, Percutaneous Endoscopic Approach	Procedure	ICD-10-PCS
0F190Z3	Bypass Common Bile Duct to Duodenum, Open Approach	Procedure	ICD-10-PCS
43644	Laparoscopy, surgical, gastric restrictive procedure; with gastric bypass and Roux-en-Y gastroenterostomy (roux limb 150 cm or less)	Procedure	CPT-4
43645	Laparoscopy, surgical, gastric restrictive procedure; with gastric bypass and small intestine reconstruction to limit absorption	Procedure	CPT-4
43659	Unlisted laparoscopy procedure, stomach	Procedure	CPT-4
43770	Laparoscopy, surgical, gastric restrictive procedure; placement of adjustable gastric restrictive device (eg, gastric band and subcutaneous port components)	Procedure	CPT-4
43842	Gastric restrictive procedure, without gastric bypass, for morbid obesity; vertical-banded gastroplasty	Procedure	CPT-4
43843	Gastric restrictive procedure, without gastric bypass, for morbid obesity; other than vertical-banded gastroplasty	Procedure	CPT-4
43844	GASTRIC BYPASS NOT ROUX-EN-Y GASTROENTEROSTOMY	Procedure	CPT-4
43845	Gastric restrictive procedure with partial gastrectomy, pylorus-preserving duodenoileostomy and ileoileostomy (50 to 100 cm common channel) to limit absorption (biliopancreatic diversion with duodenal switch)	Procedure	CPT-4
43846	Gastric restrictive procedure, with gastric bypass for morbid obesity; with short limb (150 cm or less) Roux-en-Y gastroenterostomy	Procedure	CPT-4

Code	Description	Code Category	Code Type
43847	Gastric restrictive procedure, with gastric bypass for morbid obesity; with small intestine reconstruction to limit absorption	Procedure	CPT-4
S2082	Laparoscopy, surgical; gastric restrictive procedure, adjustable gastric band includes placement of subcutaneous port	Procedure	HCPCS
S2085	Laparoscopy, gastric restrictive procedure, with gastric bypass for morbid obesity, with short limb (less than 100 cm) roux-en-y gastroenterostomy	Procedure	HCPCS
	**Immunocompromised State**		
C88.0	Waldenstrom macroglobulinemia	Diagnosis	ICD-10-CM
C96.5	Multifocal and unisystemic Langerhans-cell histiocytosis	Diagnosis	ICD-10-CM
C96.6	Unifocal Langerhans-cell histiocytosis	Diagnosis	ICD-10-CM
D47.2	Monoclonal gammopathy	Diagnosis	ICD-10-CM
D80.0	Hereditary hypogammaglobulinemia	Diagnosis	ICD-10-CM
D80.1	Nonfamilial hypogammaglobulinemia	Diagnosis	ICD-10-CM
D80.2	Selective deficiency of immunoglobulin A [IgA]	Diagnosis	ICD-10-CM
D80.3	Selective deficiency of immunoglobulin G [IgG] subclasses	Diagnosis	ICD-10-CM
D80.4	Selective deficiency of immunoglobulin M [IgM]	Diagnosis	ICD-10-CM
D80.5	Immunodeficiency with increased immunoglobulin M [IgM]	Diagnosis	ICD-10-CM
D80.6	Antibody deficiency with near-normal immunoglobulins or with hyperimmunoglobulinemia	Diagnosis	ICD-10-CM
D80.7	Transient hypogammaglobulinemia of infancy	Diagnosis	ICD-10-CM
D80.8	Other immunodeficiencies with predominantly antibody defects	Diagnosis	ICD-10-CM
D80.9	Immunodeficiency with predominantly antibody defects, unspecified	Diagnosis	ICD-10-CM
D81.0	Severe combined immunodeficiency [SCID] with reticular dysgenesis	Diagnosis	ICD-10-CM
D81.1	Severe combined immunodeficiency [SCID] with low T- and B-cell numbers	Diagnosis	ICD-10-CM
D81.2	Severe combined immunodeficiency [SCID] with low or normal B-cell numbers	Diagnosis	ICD-10-CM
D81.3	Adenosine deaminase [ADA] deficiency	Diagnosis	ICD-10-CM
D81.4	Nezelof's syndrome	Diagnosis	ICD-10-CM
D81.5	Purine nucleoside phosphorylase [PNP] deficiency	Diagnosis	ICD-10-CM
D81.6	Major histocompatibility complex class I deficiency	Diagnosis	ICD-10-CM
D81.7	Major histocompatibility complex class II deficiency	Diagnosis	ICD-10-CM
D81.810	Biotinidase deficiency	Diagnosis	ICD-10-CM
D81.818	Other biotin-dependent carboxylase deficiency	Diagnosis	ICD-10-CM
D81.819	Biotin-dependent carboxylase deficiency, unspecified	Diagnosis	ICD-10-CM
D81.89	Other combined immunodeficiencies	Diagnosis	ICD-10-CM
D81.9	Combined immunodeficiency, unspecified	Diagnosis	ICD-10-CM
D82.0	Wiskott-Aldrich syndrome	Diagnosis	ICD-10-CM
D82.1	Di George's syndrome	Diagnosis	ICD-10-CM
D82.2	Immunodeficiency with short-limbed stature	Diagnosis	ICD-10-CM
D82.3	Immunodeficiency following hereditary defective response to Epstein-Barr virus	Diagnosis	ICD-10-CM
D82.4	Hyperimmunoglobulin E [IgE] syndrome	Diagnosis	ICD-10-CM
D82.8	Immunodeficiency associated with other specified major defects	Diagnosis	ICD-10-CM
D82.9	Immunodeficiency associated with major defect, unspecified	Diagnosis	ICD-10-CM
D83.0	Common variable immunodeficiency with predominant abnormalities of B-cell numbers and function	Diagnosis	ICD-10-CM

Code	Description	Code Category	Code Type
D83.1	Common variable immunodeficiency with predominant immunoregulatory T-cell disorders	Diagnosis	ICD-10-CM
D83.2	Common variable immunodeficiency with autoantibodies to B- or T-cells	Diagnosis	ICD-10-CM
D83.8	Other common variable immunodeficiencies	Diagnosis	ICD-10-CM
D83.9	Common variable immunodeficiency, unspecified	Diagnosis	ICD-10-CM
D84.0	Lymphocyte function antigen-1 [LFA-1] defect	Diagnosis	ICD-10-CM
D84.1	Defects in the complement system	Diagnosis	ICD-10-CM
D84.8	Other specified immunodeficiencies	Diagnosis	ICD-10-CM
D84.9	Immunodeficiency, unspecified	Diagnosis	ICD-10-CM
D89.0	Polyclonal hypergammaglobulinemia	Diagnosis	ICD-10-CM
D89.1	Cryoglobulinemia	Diagnosis	ICD-10-CM
D89.2	Hypergammaglobulinemia, unspecified	Diagnosis	ICD-10-CM
D89.3	Immune reconstitution syndrome	Diagnosis	ICD-10-CM
D89.40	Mast cell activation, unspecified	Diagnosis	ICD-10-CM
D89.41	Monoclonal mast cell activation syndrome	Diagnosis	ICD-10-CM
D89.42	Idiopathic mast cell activation syndrome	Diagnosis	ICD-10-CM
D89.43	Secondary mast cell activation	Diagnosis	ICD-10-CM
D89.49	Other mast cell activation disorder	Diagnosis	ICD-10-CM
D89.810	Acute graft-versus-host disease	Diagnosis	ICD-10-CM
D89.811	Chronic graft-versus-host disease	Diagnosis	ICD-10-CM
D89.812	Acute on chronic graft-versus-host disease	Diagnosis	ICD-10-CM
D89.813	Graft-versus-host disease, unspecified	Diagnosis	ICD-10-CM
D89.82	Autoimmune lymphoproliferative syndrome [ALPS]	Diagnosis	ICD-10-CM
D89.89	Other specified disorders involving the immune mechanism, not elsewhere classified	Diagnosis	ICD-10-CM
D89.9	Disorder involving the immune mechanism, unspecified	Diagnosis	ICD-10-CM
D70.1	Agranulocytosis secondary to cancer chemotherapy	Diagnosis	ICD-10-CM
D70.2	Other drug-induced agranulocytosis	Diagnosis	ICD-10-CM
D70.3	Neutropenia due to infection	Diagnosis	ICD-10-CM
D70.4	Cyclic neutropenia	Diagnosis	ICD-10-CM
D70.8	Other neutropenia	Diagnosis	ICD-10-CM
D70.9	Neutropenia, unspecified	Diagnosis	ICD-10-CM
D72.819	Decreased white blood cell count, unspecified	Diagnosis	ICD-10-CM
T86.91	Unspecified transplanted organ and tissue rejection	Diagnosis	ICD-10-CM
Z94	Transplanted organ and tissue status	Diagnosis	ICD-10-CM
Z94.0	Kidney transplant status	Diagnosis	ICD-10-CM
Z94.1	Heart transplant status	Diagnosis	ICD-10-CM
Z94.2	Lung transplant status	Diagnosis	ICD-10-CM
Z94.3	Heart and lungs transplant status	Diagnosis	ICD-10-CM
Z94.4	Liver transplant status	Diagnosis	ICD-10-CM
Z94.5	Skin transplant status	Diagnosis	ICD-10-CM
Z94.6	Bone transplant status	Diagnosis	ICD-10-CM
Z94.7	Corneal transplant status	Diagnosis	ICD-10-CM
Z94.8	Other transplanted organ and tissue status	Diagnosis	ICD-10-CM
Z94.81	Bone marrow transplant status	Diagnosis	ICD-10-CM

Code	Description	Code Category	Code Type
Z94.82	Intestine transplant status	Diagnosis	ICD-10-CM
Z94.83	Pancreas transplant status	Diagnosis	ICD-10-CM
Z94.84	Stem cells transplant status	Diagnosis	ICD-10-CM
Z94.89	Other transplanted organ and tissue status	Diagnosis	ICD-10-CM
Z94.9	Transplanted organ and tissue status, unspecified	Diagnosis	ICD-10-CM
B20	Human immunodeficiency virus [HIV] disease	Diagnosis	ICD-10-CM
B97.35	Human immunodeficiency virus, type 2 [HIV 2] as the cause of diseases classified elsewhere	Diagnosis	ICD-10-CM
Z21	Asymptomatic human immunodeficiency virus [HIV] infection status	Diagnosis	ICD-10-CM
	**Malignant Cancer**		
C00.0	Malignant neoplasm of external upper lip	Diagnosis	ICD-10-CM
C00.1	Malignant neoplasm of external lower lip	Diagnosis	ICD-10-CM
C00.2	Malignant neoplasm of external lip, unspecified	Diagnosis	ICD-10-CM
C00.3	Malignant neoplasm of upper lip, inner aspect	Diagnosis	ICD-10-CM
C00.4	Malignant neoplasm of lower lip, inner aspect	Diagnosis	ICD-10-CM
C00.5	Malignant neoplasm of lip, unspecified, inner aspect	Diagnosis	ICD-10-CM
C00.6	Malignant neoplasm of commissure of lip, unspecified	Diagnosis	ICD-10-CM
C00.8	Malignant neoplasm of overlapping sites of lip	Diagnosis	ICD-10-CM
C00.9	Malignant neoplasm of lip, unspecified	Diagnosis	ICD-10-CM
C01	Malignant neoplasm of base of tongue	Diagnosis	ICD-10-CM
C02.0	Malignant neoplasm of dorsal surface of tongue	Diagnosis	ICD-10-CM
C02.1	Malignant neoplasm of border of tongue	Diagnosis	ICD-10-CM
C02.2	Malignant neoplasm of ventral surface of tongue	Diagnosis	ICD-10-CM
C02.3	Malignant neoplasm of anterior two-thirds of tongue, part unspecified	Diagnosis	ICD-10-CM
C02.4	Malignant neoplasm of lingual tonsil	Diagnosis	ICD-10-CM
C02.8	Malignant neoplasm of overlapping sites of tongue	Diagnosis	ICD-10-CM
C02.9	Malignant neoplasm of tongue, unspecified	Diagnosis	ICD-10-CM
C03.0	Malignant neoplasm of upper gum	Diagnosis	ICD-10-CM
C03.1	Malignant neoplasm of lower gum	Diagnosis	ICD-10-CM
C03.9	Malignant neoplasm of gum, unspecified	Diagnosis	ICD-10-CM
C04.0	Malignant neoplasm of anterior floor of mouth	Diagnosis	ICD-10-CM
C04.1	Malignant neoplasm of lateral floor of mouth	Diagnosis	ICD-10-CM
C04.8	Malignant neoplasm of overlapping sites of floor of mouth	Diagnosis	ICD-10-CM
C04.9	Malignant neoplasm of floor of mouth, unspecified	Diagnosis	ICD-10-CM
C05.0	Malignant neoplasm of hard palate	Diagnosis	ICD-10-CM
C05.1	Malignant neoplasm of soft palate	Diagnosis	ICD-10-CM
C05.2	Malignant neoplasm of uvula	Diagnosis	ICD-10-CM
C05.8	Malignant neoplasm of overlapping sites of palate	Diagnosis	ICD-10-CM
C05.9	Malignant neoplasm of palate, unspecified	Diagnosis	ICD-10-CM
C06.0	Malignant neoplasm of cheek mucosa	Diagnosis	ICD-10-CM
C06.1	Malignant neoplasm of vestibule of mouth	Diagnosis	ICD-10-CM
C06.2	Malignant neoplasm of retromolar area	Diagnosis	ICD-10-CM
C06.80	Malignant neoplasm of overlapping sites of unspecified parts of mouth	Diagnosis	ICD-10-CM
C06.89	Malignant neoplasm of overlapping sites of other parts of mouth	Diagnosis	ICD-10-CM
C06.9	Malignant neoplasm of mouth, unspecified	Diagnosis	ICD-10-CM

Code	Description	Code Category	Code Type
C07	Malignant neoplasm of parotid gland	Diagnosis	ICD-10-CM
C08.0	Malignant neoplasm of submandibular gland	Diagnosis	ICD-10-CM
C08.1	Malignant neoplasm of sublingual gland	Diagnosis	ICD-10-CM
C08.9	Malignant neoplasm of major salivary gland, unspecified	Diagnosis	ICD-10-CM
C09.0	Malignant neoplasm of tonsillar fossa	Diagnosis	ICD-10-CM
C09.1	Malignant neoplasm of tonsillar pillar (anterior) (posterior)	Diagnosis	ICD-10-CM
C09.8	Malignant neoplasm of overlapping sites of tonsil	Diagnosis	ICD-10-CM
C09.9	Malignant neoplasm of tonsil, unspecified	Diagnosis	ICD-10-CM
C10.0	Malignant neoplasm of vallecula	Diagnosis	ICD-10-CM
C10.1	Malignant neoplasm of anterior surface of epiglottis	Diagnosis	ICD-10-CM
C10.2	Malignant neoplasm of lateral wall of oropharynx	Diagnosis	ICD-10-CM
C10.3	Malignant neoplasm of posterior wall of oropharynx	Diagnosis	ICD-10-CM
C10.4	Malignant neoplasm of branchial cleft	Diagnosis	ICD-10-CM
C10.8	Malignant neoplasm of overlapping sites of oropharynx	Diagnosis	ICD-10-CM
C10.9	Malignant neoplasm of oropharynx, unspecified	Diagnosis	ICD-10-CM
C11.0	Malignant neoplasm of superior wall of nasopharynx	Diagnosis	ICD-10-CM
C11.1	Malignant neoplasm of posterior wall of nasopharynx	Diagnosis	ICD-10-CM
C11.2	Malignant neoplasm of lateral wall of nasopharynx	Diagnosis	ICD-10-CM
C11.3	Malignant neoplasm of anterior wall of nasopharynx	Diagnosis	ICD-10-CM
C11.8	Malignant neoplasm of overlapping sites of nasopharynx	Diagnosis	ICD-10-CM
C11.9	Malignant neoplasm of nasopharynx, unspecified	Diagnosis	ICD-10-CM
C12	Malignant neoplasm of pyriform sinus	Diagnosis	ICD-10-CM
C13.0	Malignant neoplasm of postcricoid region	Diagnosis	ICD-10-CM
C13.1	Malignant neoplasm of aryepiglottic fold, hypopharyngeal aspect	Diagnosis	ICD-10-CM
C13.2	Malignant neoplasm of posterior wall of hypopharynx	Diagnosis	ICD-10-CM
C13.8	Malignant neoplasm of overlapping sites of hypopharynx	Diagnosis	ICD-10-CM
C13.9	Malignant neoplasm of hypopharynx, unspecified	Diagnosis	ICD-10-CM
C14.0	Malignant neoplasm of pharynx, unspecified	Diagnosis	ICD-10-CM
C14.2	Malignant neoplasm of Waldeyer's ring	Diagnosis	ICD-10-CM
C14.8	Malignant neoplasm of overlapping sites of lip, oral cavity and pharynx	Diagnosis	ICD-10-CM
C15.3	Malignant neoplasm of upper third of esophagus	Diagnosis	ICD-10-CM
C15.4	Malignant neoplasm of middle third of esophagus	Diagnosis	ICD-10-CM
C15.5	Malignant neoplasm of lower third of esophagus	Diagnosis	ICD-10-CM
C15.8	Malignant neoplasm of overlapping sites of esophagus	Diagnosis	ICD-10-CM
C15.9	Malignant neoplasm of esophagus, unspecified	Diagnosis	ICD-10-CM
C16.0	Malignant neoplasm of cardia	Diagnosis	ICD-10-CM
C16.1	Malignant neoplasm of fundus of stomach	Diagnosis	ICD-10-CM
C16.2	Malignant neoplasm of body of stomach	Diagnosis	ICD-10-CM
C16.3	Malignant neoplasm of pyloric antrum	Diagnosis	ICD-10-CM
C16.4	Malignant neoplasm of pylorus	Diagnosis	ICD-10-CM
C16.5	Malignant neoplasm of lesser curvature of stomach, unspecified	Diagnosis	ICD-10-CM
C16.6	Malignant neoplasm of greater curvature of stomach, unspecified	Diagnosis	ICD-10-CM
C16.8	Malignant neoplasm of overlapping sites of stomach	Diagnosis	ICD-10-CM
C16.9	Malignant neoplasm of stomach, unspecified	Diagnosis	ICD-10-CM
C17.0	Malignant neoplasm of duodenum	Diagnosis	ICD-10-CM

Code	Description	Code Category	Code Type
C17.1	Malignant neoplasm of jejunum	Diagnosis	ICD-10-CM
C17.2	Malignant neoplasm of ileum	Diagnosis	ICD-10-CM
C17.3	Meckel's diverticulum, malignant	Diagnosis	ICD-10-CM
C17.8	Malignant neoplasm of overlapping sites of small intestine	Diagnosis	ICD-10-CM
C17.9	Malignant neoplasm of small intestine, unspecified	Diagnosis	ICD-10-CM
C18.0	Malignant neoplasm of cecum	Diagnosis	ICD-10-CM
C18.1	Malignant neoplasm of appendix	Diagnosis	ICD-10-CM
C18.2	Malignant neoplasm of ascending colon	Diagnosis	ICD-10-CM
C18.3	Malignant neoplasm of hepatic flexure	Diagnosis	ICD-10-CM
C18.4	Malignant neoplasm of transverse colon	Diagnosis	ICD-10-CM
C18.5	Malignant neoplasm of splenic flexure	Diagnosis	ICD-10-CM
C18.6	Malignant neoplasm of descending colon	Diagnosis	ICD-10-CM
C18.7	Malignant neoplasm of sigmoid colon	Diagnosis	ICD-10-CM
C18.8	Malignant neoplasm of overlapping sites of colon	Diagnosis	ICD-10-CM
C18.9	Malignant neoplasm of colon, unspecified	Diagnosis	ICD-10-CM
C19	Malignant neoplasm of rectosigmoid junction	Diagnosis	ICD-10-CM
C20	Malignant neoplasm of rectum	Diagnosis	ICD-10-CM
C21.0	Malignant neoplasm of anus, unspecified	Diagnosis	ICD-10-CM
C21.1	Malignant neoplasm of anal canal	Diagnosis	ICD-10-CM
C21.2	Malignant neoplasm of cloacogenic zone	Diagnosis	ICD-10-CM
C21.8	Malignant neoplasm of overlapping sites of rectum, anus and anal canal	Diagnosis	ICD-10-CM
C22.0	Liver cell carcinoma	Diagnosis	ICD-10-CM
C22.1	Intrahepatic bile duct carcinoma	Diagnosis	ICD-10-CM
C22.2	Hepatoblastoma	Diagnosis	ICD-10-CM
C22.3	Angiosarcoma of liver	Diagnosis	ICD-10-CM
C22.4	Other sarcomas of liver	Diagnosis	ICD-10-CM
C22.7	Other specified carcinomas of liver	Diagnosis	ICD-10-CM
C22.8	Malignant neoplasm of liver, primary, unspecified as to type	Diagnosis	ICD-10-CM
C22.9	Malignant neoplasm of liver, not specified as primary or secondary	Diagnosis	ICD-10-CM
C23	Malignant neoplasm of gallbladder	Diagnosis	ICD-10-CM
C24.0	Malignant neoplasm of extrahepatic bile duct	Diagnosis	ICD-10-CM
C24.1	Malignant neoplasm of ampulla of Vater	Diagnosis	ICD-10-CM
C24.8	Malignant neoplasm of overlapping sites of biliary tract	Diagnosis	ICD-10-CM
C24.9	Malignant neoplasm of biliary tract, unspecified	Diagnosis	ICD-10-CM
C25.0	Malignant neoplasm of head of pancreas	Diagnosis	ICD-10-CM
C25.1	Malignant neoplasm of body of pancreas	Diagnosis	ICD-10-CM
C25.2	Malignant neoplasm of tail of pancreas	Diagnosis	ICD-10-CM
C25.3	Malignant neoplasm of pancreatic duct	Diagnosis	ICD-10-CM
C25.4	Malignant neoplasm of endocrine pancreas	Diagnosis	ICD-10-CM
C25.7	Malignant neoplasm of other parts of pancreas	Diagnosis	ICD-10-CM
C25.8	Malignant neoplasm of overlapping sites of pancreas	Diagnosis	ICD-10-CM
C25.9	Malignant neoplasm of pancreas, unspecified	Diagnosis	ICD-10-CM
C26.0	Malignant neoplasm of intestinal tract, part unspecified	Diagnosis	ICD-10-CM
C26.1	Malignant neoplasm of spleen	Diagnosis	ICD-10-CM
C26.9	Malignant neoplasm of ill-defined sites within the digestive system	Diagnosis	ICD-10-CM

Code	Description	Code Category	Code Type
C30.0	Malignant neoplasm of nasal cavity	Diagnosis	ICD-10-CM
C30.1	Malignant neoplasm of middle ear	Diagnosis	ICD-10-CM
C31.0	Malignant neoplasm of maxillary sinus	Diagnosis	ICD-10-CM
C31.1	Malignant neoplasm of ethmoidal sinus	Diagnosis	ICD-10-CM
C31.2	Malignant neoplasm of frontal sinus	Diagnosis	ICD-10-CM
C31.3	Malignant neoplasm of sphenoid sinus	Diagnosis	ICD-10-CM
C31.8	Malignant neoplasm of overlapping sites of accessory sinuses	Diagnosis	ICD-10-CM
C31.9	Malignant neoplasm of accessory sinus, unspecified	Diagnosis	ICD-10-CM
C32.0	Malignant neoplasm of glottis	Diagnosis	ICD-10-CM
C32.1	Malignant neoplasm of supraglottis	Diagnosis	ICD-10-CM
C32.2	Malignant neoplasm of subglottis	Diagnosis	ICD-10-CM
C32.3	Malignant neoplasm of laryngeal cartilage	Diagnosis	ICD-10-CM
C32.8	Malignant neoplasm of overlapping sites of larynx	Diagnosis	ICD-10-CM
C32.9	Malignant neoplasm of larynx, unspecified	Diagnosis	ICD-10-CM
C33	Malignant neoplasm of trachea	Diagnosis	ICD-10-CM
C34.00	Malignant neoplasm of unspecified main bronchus	Diagnosis	ICD-10-CM
C34.01	Malignant neoplasm of right main bronchus	Diagnosis	ICD-10-CM
C34.02	Malignant neoplasm of left main bronchus	Diagnosis	ICD-10-CM
C34.10	Malignant neoplasm of upper lobe, unspecified bronchus or lung	Diagnosis	ICD-10-CM
C34.11	Malignant neoplasm of upper lobe, right bronchus or lung	Diagnosis	ICD-10-CM
C34.12	Malignant neoplasm of upper lobe, left bronchus or lung	Diagnosis	ICD-10-CM
C34.2	Malignant neoplasm of middle lobe, bronchus or lung	Diagnosis	ICD-10-CM
C34.30	Malignant neoplasm of lower lobe, unspecified bronchus or lung	Diagnosis	ICD-10-CM
C34.31	Malignant neoplasm of lower lobe, right bronchus or lung	Diagnosis	ICD-10-CM
C34.32	Malignant neoplasm of lower lobe, left bronchus or lung	Diagnosis	ICD-10-CM
C34.80	Malignant neoplasm of overlapping sites of unspecified bronchus and lung	Diagnosis	ICD-10-CM
C34.81	Malignant neoplasm of overlapping sites of right bronchus and lung	Diagnosis	ICD-10-CM
C34.82	Malignant neoplasm of overlapping sites of left bronchus and lung	Diagnosis	ICD-10-CM
C34.90	Malignant neoplasm of unspecified part of unspecified bronchus or lung	Diagnosis	ICD-10-CM
C34.91	Malignant neoplasm of unspecified part of right bronchus or lung	Diagnosis	ICD-10-CM
C34.92	Malignant neoplasm of unspecified part of left bronchus or lung	Diagnosis	ICD-10-CM
C37	Malignant neoplasm of thymus	Diagnosis	ICD-10-CM
C38.0	Malignant neoplasm of heart	Diagnosis	ICD-10-CM
C38.1	Malignant neoplasm of anterior mediastinum	Diagnosis	ICD-10-CM
C38.2	Malignant neoplasm of posterior mediastinum	Diagnosis	ICD-10-CM
C38.3	Malignant neoplasm of mediastinum, part unspecified	Diagnosis	ICD-10-CM
C38.4	Malignant neoplasm of pleura	Diagnosis	ICD-10-CM
C38.8	Malignant neoplasm of overlapping sites of heart, mediastinum and pleura	Diagnosis	ICD-10-CM
C39.0	Malignant neoplasm of upper respiratory tract, part unspecified	Diagnosis	ICD-10-CM
C39.9	Malignant neoplasm of lower respiratory tract, part unspecified	Diagnosis	ICD-10-CM
C40.00	Malignant neoplasm of scapula and long bones of unspecified upper limb	Diagnosis	ICD-10-CM
C40.01	Malignant neoplasm of scapula and long bones of right upper limb	Diagnosis	ICD-10-CM
C40.02	Malignant neoplasm of scapula and long bones of left upper limb	Diagnosis	ICD-10-CM
C40.10	Malignant neoplasm of short bones of unspecified upper limb	Diagnosis	ICD-10-CM
C40.11	Malignant neoplasm of short bones of right upper limb	Diagnosis	ICD-10-CM

Code	Description	Code Category	Code Type
C40.12	Malignant neoplasm of short bones of left upper limb	Diagnosis	ICD-10-CM
C40.20	Malignant neoplasm of long bones of unspecified lower limb	Diagnosis	ICD-10-CM
C40.21	Malignant neoplasm of long bones of right lower limb	Diagnosis	ICD-10-CM
C40.22	Malignant neoplasm of long bones of left lower limb	Diagnosis	ICD-10-CM
C40.30	Malignant neoplasm of short bones of unspecified lower limb	Diagnosis	ICD-10-CM
C40.31	Malignant neoplasm of short bones of right lower limb	Diagnosis	ICD-10-CM
C40.32	Malignant neoplasm of short bones of left lower limb	Diagnosis	ICD-10-CM
C40.80	Malignant neoplasm of overlapping sites of bone and articular cartilage of unspecified limb	Diagnosis	ICD-10-CM
C40.81	Malignant neoplasm of overlapping sites of bone and articular cartilage of right limb	Diagnosis	ICD-10-CM
C40.82	Malignant neoplasm of overlapping sites of bone and articular cartilage of left limb	Diagnosis	ICD-10-CM
C40.90	Malignant neoplasm of unspecified bones and articular cartilage of unspecified limb	Diagnosis	ICD-10-CM
C40.91	Malignant neoplasm of unspecified bones and articular cartilage of right limb	Diagnosis	ICD-10-CM
C40.92	Malignant neoplasm of unspecified bones and articular cartilage of left limb	Diagnosis	ICD-10-CM
C41.0	Malignant neoplasm of bones of skull and face	Diagnosis	ICD-10-CM
C41.1	Malignant neoplasm of mandible	Diagnosis	ICD-10-CM
C41.2	Malignant neoplasm of vertebral column	Diagnosis	ICD-10-CM
C41.3	Malignant neoplasm of ribs, sternum and clavicle	Diagnosis	ICD-10-CM
C41.4	Malignant neoplasm of pelvic bones, sacrum and coccyx	Diagnosis	ICD-10-CM
C41.9	Malignant neoplasm of bone and articular cartilage, unspecified	Diagnosis	ICD-10-CM
C43.0	Malignant melanoma of lip	Diagnosis	ICD-10-CM
C43.10	Malignant melanoma of unspecified eyelid, including canthus	Diagnosis	ICD-10-CM
C43.11	Malignant melanoma of right eyelid, including canthus	Diagnosis	ICD-10-CM
C43.12	Malignant melanoma of left eyelid, including canthus	Diagnosis	ICD-10-CM
C43.20	Malignant melanoma of unspecified ear and external auricular canal	Diagnosis	ICD-10-CM
C43.21	Malignant melanoma of right ear and external auricular canal	Diagnosis	ICD-10-CM
C43.22	Malignant melanoma of left ear and external auricular canal	Diagnosis	ICD-10-CM
C43.30	Malignant melanoma of unspecified part of face	Diagnosis	ICD-10-CM
C43.31	Malignant melanoma of nose	Diagnosis	ICD-10-CM
C43.39	Malignant melanoma of other parts of face	Diagnosis	ICD-10-CM
C43.4	Malignant melanoma of scalp and neck	Diagnosis	ICD-10-CM
C43.51	Malignant melanoma of anal skin	Diagnosis	ICD-10-CM
C43.52	Malignant melanoma of skin of breast	Diagnosis	ICD-10-CM
C43.59	Malignant melanoma of other part of trunk	Diagnosis	ICD-10-CM
C43.60	Malignant melanoma of unspecified upper limb, including shoulder	Diagnosis	ICD-10-CM
C43.61	Malignant melanoma of right upper limb, including shoulder	Diagnosis	ICD-10-CM
C43.62	Malignant melanoma of left upper limb, including shoulder	Diagnosis	ICD-10-CM
C43.70	Malignant melanoma of unspecified lower limb, including hip	Diagnosis	ICD-10-CM
C43.71	Malignant melanoma of right lower limb, including hip	Diagnosis	ICD-10-CM
C43.72	Malignant melanoma of left lower limb, including hip	Diagnosis	ICD-10-CM
C43.8	Malignant melanoma of overlapping sites of skin	Diagnosis	ICD-10-CM
C43.9	Malignant melanoma of skin, unspecified	Diagnosis	ICD-10-CM
C45.0	Mesothelioma of pleura	Diagnosis	ICD-10-CM
C45.1	Mesothelioma of peritoneum	Diagnosis	ICD-10-CM
C45.2	Mesothelioma of pericardium	Diagnosis	ICD-10-CM

Code	Description	Code Category	Code Type
C45.7	Mesothelioma of other sites	Diagnosis	ICD-10-CM
C45.9	Mesothelioma, unspecified	Diagnosis	ICD-10-CM
C46.0	Kaposi's sarcoma of skin	Diagnosis	ICD-10-CM
C46.1	Kaposi's sarcoma of soft tissue	Diagnosis	ICD-10-CM
C46.2	Kaposi's sarcoma of palate	Diagnosis	ICD-10-CM
C46.3	Kaposi's sarcoma of lymph nodes	Diagnosis	ICD-10-CM
C46.4	Kaposi's sarcoma of gastrointestinal sites	Diagnosis	ICD-10-CM
C46.50	Kaposi's sarcoma of unspecified lung	Diagnosis	ICD-10-CM
C46.51	Kaposi's sarcoma of right lung	Diagnosis	ICD-10-CM
C46.52	Kaposi's sarcoma of left lung	Diagnosis	ICD-10-CM
C46.7	Kaposi's sarcoma of other sites	Diagnosis	ICD-10-CM
C46.9	Kaposi's sarcoma, unspecified	Diagnosis	ICD-10-CM
C47.0	Malignant neoplasm of peripheral nerves of head, face and neck	Diagnosis	ICD-10-CM
C47.10	Malignant neoplasm of peripheral nerves of unspecified upper limb, including should	Diagnosis	ICD-10-CM
C47.11	Malignant neoplasm of peripheral nerves of right upper limb, including shoulder	Diagnosis	ICD-10-CM
C47.12	Malignant neoplasm of peripheral nerves of left upper limb, including shoulder	Diagnosis	ICD-10-CM
C47.20	Malignant neoplasm of peripheral nerves of unspecified lower limb, including hip	Diagnosis	ICD-10-CM
C47.21	Malignant neoplasm of peripheral nerves of right lower limb, including hip	Diagnosis	ICD-10-CM
C47.22	Malignant neoplasm of peripheral nerves of left lower limb, including hip	Diagnosis	ICD-10-CM
C47.3	Malignant neoplasm of peripheral nerves of thorax	Diagnosis	ICD-10-CM
C47.4	Malignant neoplasm of peripheral nerves of abdomen	Diagnosis	ICD-10-CM
C47.5	Malignant neoplasm of peripheral nerves of pelvis	Diagnosis	ICD-10-CM
C47.6	Malignant neoplasm of peripheral nerves of trunk, unspecified	Diagnosis	ICD-10-CM
C47.8	Malignant neoplasm of overlapping sites of peripheral nerves and autonomic nervous system	Diagnosis	ICD-10-CM
C47.9	Malignant neoplasm of peripheral nerves and autonomic nervous system, unspecified	Diagnosis	ICD-10-CM
C48.0	Malignant neoplasm of retroperitoneum	Diagnosis	ICD-10-CM
C48.1	Malignant neoplasm of specified parts of peritoneum	Diagnosis	ICD-10-CM
C48.2	Malignant neoplasm of peritoneum, unspecified	Diagnosis	ICD-10-CM
C48.8	Malignant neoplasm of overlapping sites of retroperitoneum and peritoneum	Diagnosis	ICD-10-CM
C49.0	Malignant neoplasm of connective and soft tissue of head, face and neck	Diagnosis	ICD-10-CM
C49.10	Malignant neoplasm of connective and soft tissue of unspecified upper limb, including shoulder	Diagnosis	ICD-10-CM
C49.11	Malignant neoplasm of connective and soft tissue of right upper limb, including shoulder	Diagnosis	ICD-10-CM
C49.12	Malignant neoplasm of connective and soft tissue of left upper limb, including shoulder	Diagnosis	ICD-10-CM
C49.20	Malignant neoplasm of connective and soft tissue of unspecified lower limb, including hip	Diagnosis	ICD-10-CM
C49.21	Malignant neoplasm of connective and soft tissue of right lower limb, including hip	Diagnosis	ICD-10-CM
C49.22	Malignant neoplasm of connective and soft tissue of left lower limb, including hip	Diagnosis	ICD-10-CM
C49.3	Malignant neoplasm of connective and soft tissue of thorax	Diagnosis	ICD-10-CM
C49.4	Malignant neoplasm of connective and soft tissue of abdomen	Diagnosis	ICD-10-CM
C49.5	Malignant neoplasm of connective and soft tissue of pelvis	Diagnosis	ICD-10-CM

Code	Description	Code Category	Code Type
C49.6	Malignant neoplasm of connective and soft tissue of trunk, unspecified	Diagnosis	ICD-10-CM
C49.8	Malignant neoplasm of overlapping sites of connective and soft tissue	Diagnosis	ICD-10-CM
C49.9	Malignant neoplasm of connective and soft tissue, unspecified	Diagnosis	ICD-10-CM
C49.A0	Gastrointestinal stromal tumor, unspecified site	Diagnosis	ICD-10-CM
C49.A1	Gastrointestinal stromal tumor of esophagus	Diagnosis	ICD-10-CM
C49.A2	Gastrointestinal stromal tumor of stomach	Diagnosis	ICD-10-CM
C49.A3	Gastrointestinal stromal tumor of small intestine	Diagnosis	ICD-10-CM
C49.A4	Gastrointestinal stromal tumor of large intestine	Diagnosis	ICD-10-CM
C49.A5	Gastrointestinal stromal tumor of rectum	Diagnosis	ICD-10-CM
C49.A9	Gastrointestinal stromal tumor of other sites	Diagnosis	ICD-10-CM
C50.011	Malignant neoplasm of nipple and areola, right female breast	Diagnosis	ICD-10-CM
C50.012	Malignant neoplasm of nipple and areola, left female breast	Diagnosis	ICD-10-CM
C50.019	Malignant neoplasm of nipple and areola, unspecified female breast	Diagnosis	ICD-10-CM
C50.021	Malignant neoplasm of nipple and areola, right male breast	Diagnosis	ICD-10-CM
C50.022	Malignant neoplasm of nipple and areola, left male breast	Diagnosis	ICD-10-CM
C50.029	Malignant neoplasm of nipple and areola, unspecified male breast	Diagnosis	ICD-10-CM
C50.111	Malignant neoplasm of central portion of right female breast	Diagnosis	ICD-10-CM
C50.112	Malignant neoplasm of central portion of left female breast	Diagnosis	ICD-10-CM
C50.119	Malignant neoplasm of central portion of unspecified female breast	Diagnosis	ICD-10-CM
C50.121	Malignant neoplasm of central portion of right male breast	Diagnosis	ICD-10-CM
C50.122	Malignant neoplasm of central portion of left male breast	Diagnosis	ICD-10-CM
C50.129	Malignant neoplasm of central portion of unspecified male breast	Diagnosis	ICD-10-CM
C50.211	Malignant neoplasm of upper-inner quadrant of right female breast	Diagnosis	ICD-10-CM
C50.212	Malignant neoplasm of upper-inner quadrant of left female breast	Diagnosis	ICD-10-CM
C50.219	Malignant neoplasm of upper-inner quadrant of unspecified female breast	Diagnosis	ICD-10-CM
C50.221	Malignant neoplasm of upper-inner quadrant of right male breast	Diagnosis	ICD-10-CM
C50.222	Malignant neoplasm of upper-inner quadrant of left male breast	Diagnosis	ICD-10-CM
C50.229	Malignant neoplasm of upper-inner quadrant of unspecified male breast	Diagnosis	ICD-10-CM
C50.311	Malignant neoplasm of lower-inner quadrant of right female breast	Diagnosis	ICD-10-CM
C50.312	Malignant neoplasm of lower-inner quadrant of left female breast	Diagnosis	ICD-10-CM
C50.319	Malignant neoplasm of lower-inner quadrant of unspecified female breast	Diagnosis	ICD-10-CM
C50.321	Malignant neoplasm of lower-inner quadrant of right male breast	Diagnosis	ICD-10-CM
C50.322	Malignant neoplasm of lower-inner quadrant of left male breast	Diagnosis	ICD-10-CM
C50.329	Malignant neoplasm of lower-inner quadrant of unspecified male breast	Diagnosis	ICD-10-CM
C50.411	Malignant neoplasm of upper-outer quadrant of right female breast	Diagnosis	ICD-10-CM
C50.412	Malignant neoplasm of upper-outer quadrant of left female breast	Diagnosis	ICD-10-CM
C50.419	Malignant neoplasm of upper-outer quadrant of unspecified female breast	Diagnosis	ICD-10-CM
C50.421	Malignant neoplasm of upper-outer quadrant of right male breast	Diagnosis	ICD-10-CM
C50.422	Malignant neoplasm of upper-outer quadrant of left male breast	Diagnosis	ICD-10-CM
C50.429	Malignant neoplasm of upper-outer quadrant of unspecified male breast	Diagnosis	ICD-10-CM
C50.511	Malignant neoplasm of lower-outer quadrant of right female breast	Diagnosis	ICD-10-CM
C50.512	Malignant neoplasm of lower-outer quadrant of left female breast	Diagnosis	ICD-10-CM
C50.519	Malignant neoplasm of lower-outer quadrant of unspecified female breast	Diagnosis	ICD-10-CM
C50.521	Malignant neoplasm of lower-outer quadrant of right male breast	Diagnosis	ICD-10-CM
C50.522	Malignant neoplasm of lower-outer quadrant of left male breast	Diagnosis	ICD-10-CM

Code	Description	Code Category	Code Type
C50.529	Malignant neoplasm of lower-outer quadrant of unspecified male breast	Diagnosis	ICD-10-CM
C50.611	Malignant neoplasm of axillary tail of right female breast	Diagnosis	ICD-10-CM
C50.612	Malignant neoplasm of axillary tail of left female breast	Diagnosis	ICD-10-CM
C50.619	Malignant neoplasm of axillary tail of unspecified female breast	Diagnosis	ICD-10-CM
C50.621	Malignant neoplasm of axillary tail of right male breast	Diagnosis	ICD-10-CM
C50.622	Malignant neoplasm of axillary tail of left male breast	Diagnosis	ICD-10-CM
C50.629	Malignant neoplasm of axillary tail of unspecified male breast	Diagnosis	ICD-10-CM
C50.811	Malignant neoplasm of overlapping sites of right female breast	Diagnosis	ICD-10-CM
C50.812	Malignant neoplasm of overlapping sites of left female breast	Diagnosis	ICD-10-CM
C50.819	Malignant neoplasm of overlapping sites of unspecified female breast	Diagnosis	ICD-10-CM
C50.821	Malignant neoplasm of overlapping sites of right male breast	Diagnosis	ICD-10-CM
C50.822	Malignant neoplasm of overlapping sites of left male breast	Diagnosis	ICD-10-CM
C50.829	Malignant neoplasm of overlapping sites of unspecified male breast	Diagnosis	ICD-10-CM
C50.911	Malignant neoplasm of unspecified site of right female breast	Diagnosis	ICD-10-CM
C50.912	Malignant neoplasm of unspecified site of left female breast	Diagnosis	ICD-10-CM
C50.919	Malignant neoplasm of unspecified site of unspecified female breast	Diagnosis	ICD-10-CM
C50.921	Malignant neoplasm of unspecified site of right male breast	Diagnosis	ICD-10-CM
C50.922	Malignant neoplasm of unspecified site of left male breast	Diagnosis	ICD-10-CM
C50.929	Malignant neoplasm of unspecified site of unspecified male breast	Diagnosis	ICD-10-CM
C51.0	Malignant neoplasm of labium majus	Diagnosis	ICD-10-CM
C51.1	Malignant neoplasm of labium minus	Diagnosis	ICD-10-CM
C51.2	Malignant neoplasm of clitoris	Diagnosis	ICD-10-CM
C51.8	Malignant neoplasm of overlapping sites of vulva	Diagnosis	ICD-10-CM
C51.9	Malignant neoplasm of vulva, unspecified	Diagnosis	ICD-10-CM
C52	Malignant neoplasm of vagina	Diagnosis	ICD-10-CM
C53.0	Malignant neoplasm of endocervix	Diagnosis	ICD-10-CM
C53.1	Malignant neoplasm of exocervix	Diagnosis	ICD-10-CM
C53.8	Malignant neoplasm of overlapping sites of cervix uteri	Diagnosis	ICD-10-CM
C53.9	Malignant neoplasm of cervix uteri, unspecified	Diagnosis	ICD-10-CM
C54.0	Malignant neoplasm of isthmus uteri	Diagnosis	ICD-10-CM
C54.1	Malignant neoplasm of endometrium	Diagnosis	ICD-10-CM
C54.2	Malignant neoplasm of myometrium	Diagnosis	ICD-10-CM
C54.3	Malignant neoplasm of fundus uteri	Diagnosis	ICD-10-CM
C54.8	Malignant neoplasm of overlapping sites of corpus uteri	Diagnosis	ICD-10-CM
C54.9	Malignant neoplasm of corpus uteri, unspecified	Diagnosis	ICD-10-CM
C55	Malignant neoplasm of uterus, part unspecified	Diagnosis	ICD-10-CM
C56.1	Malignant neoplasm of right ovary	Diagnosis	ICD-10-CM
C56.2	Malignant neoplasm of left ovary	Diagnosis	ICD-10-CM
C56.9	Malignant neoplasm of unspecified ovary	Diagnosis	ICD-10-CM
C57.00	Malignant neoplasm of unspecified fallopian tube	Diagnosis	ICD-10-CM
C57.01	Malignant neoplasm of right fallopian tube	Diagnosis	ICD-10-CM
C57.02	Malignant neoplasm of left fallopian tube	Diagnosis	ICD-10-CM
C57.10	Malignant neoplasm of unspecified broad ligament	Diagnosis	ICD-10-CM
C57.11	Malignant neoplasm of right broad ligament	Diagnosis	ICD-10-CM
C57.12	Malignant neoplasm of left broad ligament	Diagnosis	ICD-10-CM

Code	Description	Code Category	Code Type
C57.20	Malignant neoplasm of unspecified round ligament	Diagnosis	ICD-10-CM
C57.21	Malignant neoplasm of right round ligament	Diagnosis	ICD-10-CM
C57.22	Malignant neoplasm of left round ligament	Diagnosis	ICD-10-CM
C57.3	Malignant neoplasm of parametrium	Diagnosis	ICD-10-CM
C57.4	Malignant neoplasm of uterine adnexa, unspecified	Diagnosis	ICD-10-CM
C57.7	Malignant neoplasm of other specified female genital organs	Diagnosis	ICD-10-CM
C57.8	Malignant neoplasm of overlapping sites of female genital organs	Diagnosis	ICD-10-CM
C57.9	Malignant neoplasm of female genital organ, unspecified	Diagnosis	ICD-10-CM
C58	Malignant neoplasm of placenta	Diagnosis	ICD-10-CM
C60.0	Malignant neoplasm of prepuce	Diagnosis	ICD-10-CM
C60.1	Malignant neoplasm of glans penis	Diagnosis	ICD-10-CM
C60.2	Malignant neoplasm of body of penis	Diagnosis	ICD-10-CM
C60.8	Malignant neoplasm of overlapping sites of penis	Diagnosis	ICD-10-CM
C60.9	Malignant neoplasm of penis, unspecified	Diagnosis	ICD-10-CM
C61	Malignant neoplasm of prostate	Diagnosis	ICD-10-CM
C62.00	Malignant neoplasm of unspecified undescended testis	Diagnosis	ICD-10-CM
C62.01	Malignant neoplasm of undescended right testis	Diagnosis	ICD-10-CM
C62.02	Malignant neoplasm of undescended left testis	Diagnosis	ICD-10-CM
C62.10	Malignant neoplasm of unspecified descended testis	Diagnosis	ICD-10-CM
C62.11	Malignant neoplasm of descended right testis	Diagnosis	ICD-10-CM
C62.12	Malignant neoplasm of descended left testis	Diagnosis	ICD-10-CM
C62.90	Malignant neoplasm of unspecified testis, unspecified whether descended or undescended	Diagnosis	ICD-10-CM
C62.91	Malignant neoplasm of right testis, unspecified whether descended or undescended	Diagnosis	ICD-10-CM
C62.92	Malignant neoplasm of left testis, unspecified whether descended or undescended	Diagnosis	ICD-10-CM
C63.00	Malignant neoplasm of unspecified epididymis	Diagnosis	ICD-10-CM
C63.01	Malignant neoplasm of right epididymis	Diagnosis	ICD-10-CM
C63.02	Malignant neoplasm of left epididymis	Diagnosis	ICD-10-CM
C63.10	Malignant neoplasm of unspecified spermatic cord	Diagnosis	ICD-10-CM
C63.11	Malignant neoplasm of right spermatic cord	Diagnosis	ICD-10-CM
C63.12	Malignant neoplasm of left spermatic cord	Diagnosis	ICD-10-CM
C63.2	Malignant neoplasm of scrotum	Diagnosis	ICD-10-CM
C63.7	Malignant neoplasm of other specified male genital organs	Diagnosis	ICD-10-CM
C63.8	Malignant neoplasm of overlapping sites of male genital organs	Diagnosis	ICD-10-CM
C63.9	Malignant neoplasm of male genital organ, unspecified	Diagnosis	ICD-10-CM
C64.1	Malignant neoplasm of right kidney, except renal pelvis	Diagnosis	ICD-10-CM
C64.2	Malignant neoplasm of left kidney, except renal pelvis	Diagnosis	ICD-10-CM
C64.9	Malignant neoplasm of unspecified kidney, except renal pelvis	Diagnosis	ICD-10-CM
C65.1	Malignant neoplasm of right renal pelvis	Diagnosis	ICD-10-CM
C65.2	Malignant neoplasm of left renal pelvis	Diagnosis	ICD-10-CM
C65.9	Malignant neoplasm of unspecified renal pelvis	Diagnosis	ICD-10-CM
C66.1	Malignant neoplasm of right ureter	Diagnosis	ICD-10-CM
C66.2	Malignant neoplasm of left ureter	Diagnosis	ICD-10-CM
C66.9	Malignant neoplasm of unspecified ureter	Diagnosis	ICD-10-CM
C67.0	Malignant neoplasm of trigone of bladder	Diagnosis	ICD-10-CM

Code	Description	Code Category	Code Type
C67.1	Malignant neoplasm of dome of bladder	Diagnosis	ICD-10-CM
C67.2	Malignant neoplasm of lateral wall of bladder	Diagnosis	ICD-10-CM
C67.3	Malignant neoplasm of anterior wall of bladder	Diagnosis	ICD-10-CM
C67.4	Malignant neoplasm of posterior wall of bladder	Diagnosis	ICD-10-CM
C67.5	Malignant neoplasm of bladder neck	Diagnosis	ICD-10-CM
C67.6	Malignant neoplasm of ureteric orifice	Diagnosis	ICD-10-CM
C67.7	Malignant neoplasm of urachus	Diagnosis	ICD-10-CM
C67.8	Malignant neoplasm of overlapping sites of bladder	Diagnosis	ICD-10-CM
C67.9	Malignant neoplasm of bladder, unspecified	Diagnosis	ICD-10-CM
C68.0	Malignant neoplasm of urethra	Diagnosis	ICD-10-CM
C68.1	Malignant neoplasm of paraurethral glands	Diagnosis	ICD-10-CM
C68.8	Malignant neoplasm of overlapping sites of urinary organs	Diagnosis	ICD-10-CM
C68.9	Malignant neoplasm of urinary organ, unspecified	Diagnosis	ICD-10-CM
C69.00	Malignant neoplasm of unspecified conjunctiva	Diagnosis	ICD-10-CM
C69.01	Malignant neoplasm of right conjunctiva	Diagnosis	ICD-10-CM
C69.02	Malignant neoplasm of left conjunctiva	Diagnosis	ICD-10-CM
C69.10	Malignant neoplasm of unspecified cornea	Diagnosis	ICD-10-CM
C69.11	Malignant neoplasm of right cornea	Diagnosis	ICD-10-CM
C69.12	Malignant neoplasm of left cornea	Diagnosis	ICD-10-CM
C69.20	Malignant neoplasm of unspecified retina	Diagnosis	ICD-10-CM
C69.21	Malignant neoplasm of right retina	Diagnosis	ICD-10-CM
C69.22	Malignant neoplasm of left retina	Diagnosis	ICD-10-CM
C69.30	Malignant neoplasm of unspecified choroid	Diagnosis	ICD-10-CM
C69.31	Malignant neoplasm of right choroid	Diagnosis	ICD-10-CM
C69.32	Malignant neoplasm of left choroid	Diagnosis	ICD-10-CM
C69.40	Malignant neoplasm of unspecified ciliary body	Diagnosis	ICD-10-CM
C69.41	Malignant neoplasm of right ciliary body	Diagnosis	ICD-10-CM
C69.42	Malignant neoplasm of left ciliary body	Diagnosis	ICD-10-CM
C69.50	Malignant neoplasm of unspecified lacrimal gland and duct	Diagnosis	ICD-10-CM
C69.51	Malignant neoplasm of right lacrimal gland and duct	Diagnosis	ICD-10-CM
C69.52	Malignant neoplasm of left lacrimal gland and duct	Diagnosis	ICD-10-CM
C69.60	Malignant neoplasm of unspecified orbit	Diagnosis	ICD-10-CM
C69.61	Malignant neoplasm of right orbit	Diagnosis	ICD-10-CM
C69.62	Malignant neoplasm of left orbit	Diagnosis	ICD-10-CM
C69.80	Malignant neoplasm of overlapping sites of unspecified eye and adnexa	Diagnosis	ICD-10-CM
C69.81	Malignant neoplasm of overlapping sites of right eye and adnexa	Diagnosis	ICD-10-CM
C69.82	Malignant neoplasm of overlapping sites of left eye and adnexa	Diagnosis	ICD-10-CM
C69.90	Malignant neoplasm of unspecified site of unspecified eye	Diagnosis	ICD-10-CM
C69.91	Malignant neoplasm of unspecified site of right eye	Diagnosis	ICD-10-CM
C69.92	Malignant neoplasm of unspecified site of left eye	Diagnosis	ICD-10-CM
C70.0	Malignant neoplasm of cerebral meninges	Diagnosis	ICD-10-CM
C70.1	Malignant neoplasm of spinal meninges	Diagnosis	ICD-10-CM
C70.9	Malignant neoplasm of meninges, unspecified	Diagnosis	ICD-10-CM
C71.0	Malignant neoplasm of cerebrum, except lobes and ventricles	Diagnosis	ICD-10-CM
C71.1	Malignant neoplasm of frontal lobe	Diagnosis	ICD-10-CM

Code	Description	Code Category	Code Type
C71.2	Malignant neoplasm of temporal lobe	Diagnosis	ICD-10-CM
C71.3	Malignant neoplasm of parietal lobe	Diagnosis	ICD-10-CM
C71.4	Malignant neoplasm of occipital lobe	Diagnosis	ICD-10-CM
C71.5	Malignant neoplasm of cerebral ventricle	Diagnosis	ICD-10-CM
C71.6	Malignant neoplasm of cerebellum	Diagnosis	ICD-10-CM
C71.7	Malignant neoplasm of brain stem	Diagnosis	ICD-10-CM
C71.8	Malignant neoplasm of overlapping sites of brain	Diagnosis	ICD-10-CM
C71.9	Malignant neoplasm of brain, unspecified	Diagnosis	ICD-10-CM
C72.0	Malignant neoplasm of spinal cord	Diagnosis	ICD-10-CM
C72.1	Malignant neoplasm of cauda equina	Diagnosis	ICD-10-CM
C72.20	Malignant neoplasm of unspecified olfactory nerve	Diagnosis	ICD-10-CM
C72.21	Malignant neoplasm of right olfactory nerve	Diagnosis	ICD-10-CM
C72.22	Malignant neoplasm of left olfactory nerve	Diagnosis	ICD-10-CM
C72.30	Malignant neoplasm of unspecified optic nerve	Diagnosis	ICD-10-CM
C72.31	Malignant neoplasm of right optic nerve	Diagnosis	ICD-10-CM
C72.32	Malignant neoplasm of left optic nerve	Diagnosis	ICD-10-CM
C72.40	Malignant neoplasm of unspecified acoustic nerve	Diagnosis	ICD-10-CM
C72.41	Malignant neoplasm of right acoustic nerve	Diagnosis	ICD-10-CM
C72.42	Malignant neoplasm of left acoustic nerve	Diagnosis	ICD-10-CM
C72.50	Malignant neoplasm of unspecified cranial nerve	Diagnosis	ICD-10-CM
C72.59	Malignant neoplasm of other cranial nerves	Diagnosis	ICD-10-CM
C72.9	Malignant neoplasm of central nervous system, unspecified	Diagnosis	ICD-10-CM
C73	Malignant neoplasm of thyroid gland	Diagnosis	ICD-10-CM
C74.00	Malignant neoplasm of cortex of unspecified adrenal gland	Diagnosis	ICD-10-CM
C74.01	Malignant neoplasm of cortex of right adrenal gland	Diagnosis	ICD-10-CM
C74.02	Malignant neoplasm of cortex of left adrenal gland	Diagnosis	ICD-10-CM
C74.10	Malignant neoplasm of medulla of unspecified adrenal gland	Diagnosis	ICD-10-CM
C74.11	Malignant neoplasm of medulla of right adrenal gland	Diagnosis	ICD-10-CM
C74.12	Malignant neoplasm of medulla of left adrenal gland	Diagnosis	ICD-10-CM
C74.90	Malignant neoplasm of unspecified part of unspecified adrenal gland	Diagnosis	ICD-10-CM
C74.91	Malignant neoplasm of unspecified part of right adrenal gland	Diagnosis	ICD-10-CM
C74.92	Malignant neoplasm of unspecified part of left adrenal gland	Diagnosis	ICD-10-CM
C75.0	Malignant neoplasm of parathyroid gland	Diagnosis	ICD-10-CM
C75.1	Malignant neoplasm of pituitary gland	Diagnosis	ICD-10-CM
C75.2	Malignant neoplasm of craniopharyngeal duct	Diagnosis	ICD-10-CM
C75.3	Malignant neoplasm of pineal gland	Diagnosis	ICD-10-CM
C75.4	Malignant neoplasm of carotid body	Diagnosis	ICD-10-CM
C75.5	Malignant neoplasm of aortic body and other paraganglia	Diagnosis	ICD-10-CM
C75.8	Malignant neoplasm with pluriglandular involvement, unspecified	Diagnosis	ICD-10-CM
C75.9	Malignant neoplasm of endocrine gland, unspecified	Diagnosis	ICD-10-CM
C76.0	Malignant neoplasm of head, face and neck	Diagnosis	ICD-10-CM
C76.1	Malignant neoplasm of thorax	Diagnosis	ICD-10-CM
C76.2	Malignant neoplasm of abdomen	Diagnosis	ICD-10-CM
C76.3	Malignant neoplasm of pelvis	Diagnosis	ICD-10-CM
C76.40	Malignant neoplasm of unspecified upper limb	Diagnosis	ICD-10-CM

Code	Description	Code Category	Code Type
C76.41	Malignant neoplasm of right upper limb	Diagnosis	ICD-10-CM
C76.42	Malignant neoplasm of left upper limb	Diagnosis	ICD-10-CM
C76.50	Malignant neoplasm of unspecified lower limb	Diagnosis	ICD-10-CM
C76.51	Malignant neoplasm of right lower limb	Diagnosis	ICD-10-CM
C76.52	Malignant neoplasm of left lower limb	Diagnosis	ICD-10-CM
C76.8	Malignant neoplasm of other specified ill-defined sites	Diagnosis	ICD-10-CM
C77.0	Secondary and unspecified malignant neoplasm of lymph nodes of head, face and neck	Diagnosis	ICD-10-CM
C77.1	Secondary and unspecified malignant neoplasm of intrathoracic lymph nodes	Diagnosis	ICD-10-CM
C77.2	Secondary and unspecified malignant neoplasm of intra-abdominal lymph nodes	Diagnosis	ICD-10-CM
C77.3	Secondary and unspecified malignant neoplasm of axilla and upper limb lymph node	Diagnosis	ICD-10-CM
C77.4	Secondary and unspecified malignant neoplasm of inguinal and lower limb lymph nodes	Diagnosis	ICD-10-CM
C77.5	Secondary and unspecified malignant neoplasm of intrapelvic lymph nodes	Diagnosis	ICD-10-CM
C77.8	Secondary and unspecified malignant neoplasm of lymph nodes of multiple regions	Diagnosis	ICD-10-CM
C77.9	Secondary and unspecified malignant neoplasm of lymph node, unspecified	Diagnosis	ICD-10-CM
C78.00	Secondary malignant neoplasm of unspecified lung	Diagnosis	ICD-10-CM
C78.01	Secondary malignant neoplasm of right lung	Diagnosis	ICD-10-CM
C78.02	Secondary malignant neoplasm of left lung	Diagnosis	ICD-10-CM
C78.1	Secondary malignant neoplasm of mediastinum	Diagnosis	ICD-10-CM
C78.2	Secondary malignant neoplasm of pleura	Diagnosis	ICD-10-CM
C78.30	Secondary malignant neoplasm of unspecified respiratory organ	Diagnosis	ICD-10-CM
C78.39	Secondary malignant neoplasm of other respiratory organs	Diagnosis	ICD-10-CM
C78.4	Secondary malignant neoplasm of small intestine	Diagnosis	ICD-10-CM
C78.5	Secondary malignant neoplasm of large intestine and rectum	Diagnosis	ICD-10-CM
C78.6	Secondary malignant neoplasm of retroperitoneum and peritoneum	Diagnosis	ICD-10-CM
C78.7	Secondary malignant neoplasm of liver and intrahepatic bile duct	Diagnosis	ICD-10-CM
C78.80	Secondary malignant neoplasm of unspecified digestive organ	Diagnosis	ICD-10-CM
C78.89	Secondary malignant neoplasm of other digestive organs	Diagnosis	ICD-10-CM
C79.00	Secondary malignant neoplasm of unspecified kidney and renal pelvis	Diagnosis	ICD-10-CM
C79.01	Secondary malignant neoplasm of right kidney and renal pelvis	Diagnosis	ICD-10-CM
C79.02	Secondary malignant neoplasm of left kidney and renal pelvis	Diagnosis	ICD-10-CM
C79.10	Secondary malignant neoplasm of unspecified urinary organs	Diagnosis	ICD-10-CM
C79.11	Secondary malignant neoplasm of bladder	Diagnosis	ICD-10-CM
C79.19	Secondary malignant neoplasm of other urinary organs	Diagnosis	ICD-10-CM
C79.2	Secondary malignant neoplasm of skin	Diagnosis	ICD-10-CM
C79.31	Secondary malignant neoplasm of brain	Diagnosis	ICD-10-CM
C79.32	Secondary malignant neoplasm of cerebral meninges	Diagnosis	ICD-10-CM
C79.40	Secondary malignant neoplasm of unspecified part of nervous system	Diagnosis	ICD-10-CM
C79.49	Secondary malignant neoplasm of other parts of nervous system	Diagnosis	ICD-10-CM
C79.51	Secondary malignant neoplasm of bone	Diagnosis	ICD-10-CM
C79.52	Secondary malignant neoplasm of bone marrow	Diagnosis	ICD-10-CM
C79.60	Secondary malignant neoplasm of unspecified ovary	Diagnosis	ICD-10-CM
C79.61	Secondary malignant neoplasm of right ovary	Diagnosis	ICD-10-CM
C79.62	Secondary malignant neoplasm of left ovary	Diagnosis	ICD-10-CM

Code	Description	Code Category	Code Type
C79.70	Secondary malignant neoplasm of unspecified adrenal gland	Diagnosis	ICD-10-CM
C79.71	Secondary malignant neoplasm of right adrenal gland	Diagnosis	ICD-10-CM
C79.72	Secondary malignant neoplasm of left adrenal gland	Diagnosis	ICD-10-CM
C79.81	Secondary malignant neoplasm of breast	Diagnosis	ICD-10-CM
C79.82	Secondary malignant neoplasm of genital organs	Diagnosis	ICD-10-CM
C79.89	Secondary malignant neoplasm of other specified sites	Diagnosis	ICD-10-CM
C79.9	Secondary malignant neoplasm of unspecified site	Diagnosis	ICD-10-CM
C80.0	Disseminated malignant neoplasm, unspecified	Diagnosis	ICD-10-CM
C80.1	Malignant (primary) neoplasm, unspecified	Diagnosis	ICD-10-CM
C80.2	Malignant neoplasm associated with transplanted organ	Diagnosis	ICD-10-CM
C81.00	Nodular lymphocyte predominant Hodgkin lymphoma, unspecified site	Diagnosis	ICD-10-CM
C81.01	Nodular lymphocyte predominant Hodgkin lymphoma, lymph nodes of head, face, and neck	Diagnosis	ICD-10-CM
C81.02	Nodular lymphocyte predominant Hodgkin lymphoma, intrathoracic lymph nodes	Diagnosis	ICD-10-CM
C81.03	Nodular lymphocyte predominant Hodgkin lymphoma, intra-abdominal lymph nodes	Diagnosis	ICD-10-CM
C81.04	Nodular lymphocyte predominant Hodgkin lymphoma, lymph nodes of axilla and upper limb	Diagnosis	ICD-10-CM
C81.05	Nodular lymphocyte predominant Hodgkin lymphoma, lymph nodes of inguinal region and lower limb	Diagnosis	ICD-10-CM
C81.06	Nodular lymphocyte predominant Hodgkin lymphoma, intrapelvic lymph nodes	Diagnosis	ICD-10-CM
C81.07	Nodular lymphocyte predominant Hodgkin lymphoma, spleen	Diagnosis	ICD-10-CM
C81.08	Nodular lymphocyte predominant Hodgkin lymphoma, lymph nodes of multiple sites	Diagnosis	ICD-10-CM
C81.09	Nodular lymphocyte predominant Hodgkin lymphoma, extranodal and solid organ sites	Diagnosis	ICD-10-CM
C81.10	Nodular sclerosis Hodgkin lymphoma, unspecified site	Diagnosis	ICD-10-CM
C81.11	Nodular sclerosis Hodgkin lymphoma, lymph nodes of head, face, and neck	Diagnosis	ICD-10-CM
C81.12	Nodular sclerosis Hodgkin lymphoma, intrathoracic lymph nodes	Diagnosis	ICD-10-CM
C81.13	Nodular sclerosis Hodgkin lymphoma, intra-abdominal lymph nodes	Diagnosis	ICD-10-CM
C81.14	Nodular sclerosis Hodgkin lymphoma, lymph nodes of axilla and upper limb	Diagnosis	ICD-10-CM
C81.15	Nodular sclerosis Hodgkin lymphoma, lymph nodes of inguinal region and lower liml	Diagnosis	ICD-10-CM
C81.16	Nodular sclerosis Hodgkin lymphoma, intrapelvic lymph nodes	Diagnosis	ICD-10-CM
C81.17	Nodular sclerosis Hodgkin lymphoma, spleen	Diagnosis	ICD-10-CM
C81.18	Nodular sclerosis Hodgkin lymphoma, lymph nodes of multiple sites	Diagnosis	ICD-10-CM
C81.19	Nodular sclerosis Hodgkin lymphoma, extranodal and solid organ sites	Diagnosis	ICD-10-CM
C81.20	Mixed cellularity Hodgkin lymphoma, unspecified site	Diagnosis	ICD-10-CM
C81.21	Mixed cellularity Hodgkin lymphoma, lymph nodes of head, face, and neck	Diagnosis	ICD-10-CM
C81.22	Mixed cellularity Hodgkin lymphoma, intrathoracic lymph nodes	Diagnosis	ICD-10-CM
C81.23	Mixed cellularity Hodgkin lymphoma, intra-abdominal lymph nodes	Diagnosis	ICD-10-CM
C81.24	Mixed cellularity Hodgkin lymphoma, lymph nodes of axilla and upper limb	Diagnosis	ICD-10-CM
C81.25	Mixed cellularity Hodgkin lymphoma, lymph nodes of inguinal region and lower limb	Diagnosis	ICD-10-CM
C81.26	Mixed cellularity Hodgkin lymphoma, intrapelvic lymph nodes	Diagnosis	ICD-10-CM
C81.27	Mixed cellularity Hodgkin lymphoma, spleen	Diagnosis	ICD-10-CM
C81.28	Mixed cellularity Hodgkin lymphoma, lymph nodes of multiple sites	Diagnosis	ICD-10-CM

Code	Description	Code Category	Code Type
C81.29	Mixed cellularity Hodgkin lymphoma, extranodal and solid organ sites	Diagnosis	ICD-10-CM
C81.30	Lymphocyte depleted Hodgkin lymphoma, unspecified site	Diagnosis	ICD-10-CM
C81.31	Lymphocyte depleted Hodgkin lymphoma, lymph nodes of head, face, and neck	Diagnosis	ICD-10-CM
C81.32	Lymphocyte depleted Hodgkin lymphoma, intrathoracic lymph nodes	Diagnosis	ICD-10-CM
C81.33	Lymphocyte depleted Hodgkin lymphoma, intra-abdominal lymph nodes	Diagnosis	ICD-10-CM
C81.34	Lymphocyte depleted Hodgkin lymphoma, lymph nodes of axilla and upper limb	Diagnosis	ICD-10-CM
C81.35	Lymphocyte depleted Hodgkin lymphoma, lymph nodes of inguinal region and lower limb	Diagnosis	ICD-10-CM
C81.36	Lymphocyte depleted Hodgkin lymphoma, intrapelvic lymph nodes	Diagnosis	ICD-10-CM
C81.37	Lymphocyte depleted Hodgkin lymphoma, spleen	Diagnosis	ICD-10-CM
C81.38	Lymphocyte depleted Hodgkin lymphoma, lymph nodes of multiple sites	Diagnosis	ICD-10-CM
C81.39	Lymphocyte depleted Hodgkin lymphoma, extranodal and solid organ sites	Diagnosis	ICD-10-CM
C81.40	Lymphocyte-rich Hodgkin lymphoma, unspecified site	Diagnosis	ICD-10-CM
C81.41	Lymphocyte-rich Hodgkin lymphoma, lymph nodes of head, face, and neck	Diagnosis	ICD-10-CM
C81.42	Lymphocyte-rich Hodgkin lymphoma, intrathoracic lymph nodes	Diagnosis	ICD-10-CM
C81.43	Lymphocyte-rich Hodgkin lymphoma, intra-abdominal lymph nodes	Diagnosis	ICD-10-CM
C81.44	Lymphocyte-rich Hodgkin lymphoma, lymph nodes of axilla and upper limb	Diagnosis	ICD-10-CM
C81.45	Lymphocyte-rich Hodgkin lymphoma, lymph nodes of inguinal region and lower limb	Diagnosis	ICD-10-CM
C81.46	Lymphocyte-rich Hodgkin lymphoma, intrapelvic lymph nodes	Diagnosis	ICD-10-CM
C81.47	Lymphocyte-rich Hodgkin lymphoma, spleen	Diagnosis	ICD-10-CM
C81.48	Lymphocyte-rich Hodgkin lymphoma, lymph nodes of multiple sites	Diagnosis	ICD-10-CM
C81.49	Lymphocyte-rich Hodgkin lymphoma, extranodal and solid organ sites	Diagnosis	ICD-10-CM
C81.70	Other Hodgkin lymphoma, unspecified site	Diagnosis	ICD-10-CM
C81.71	Other Hodgkin lymphoma, lymph nodes of head, face, and neck	Diagnosis	ICD-10-CM
C81.72	Other Hodgkin lymphoma, intrathoracic lymph nodes	Diagnosis	ICD-10-CM
C81.73	Other Hodgkin lymphoma, intra-abdominal lymph nodes	Diagnosis	ICD-10-CM
C81.74	Other Hodgkin lymphoma, lymph nodes of axilla and upper limb	Diagnosis	ICD-10-CM
C81.75	Other Hodgkin lymphoma, lymph nodes of inguinal region and lower limb	Diagnosis	ICD-10-CM
C81.76	Other Hodgkin lymphoma, intrapelvic lymph nodes	Diagnosis	ICD-10-CM
C81.77	Other Hodgkin lymphoma, spleen	Diagnosis	ICD-10-CM
C81.78	Other Hodgkin lymphoma, lymph nodes of multiple sites	Diagnosis	ICD-10-CM
C81.79	Other Hodgkin lymphoma, extranodal and solid organ sites	Diagnosis	ICD-10-CM
C81.90	Hodgkin lymphoma, unspecified, unspecified site	Diagnosis	ICD-10-CM
C81.91	Hodgkin lymphoma, unspecified, lymph nodes of head, face, and neck	Diagnosis	ICD-10-CM
C81.92	Hodgkin lymphoma, unspecified, intrathoracic lymph nodes	Diagnosis	ICD-10-CM
C81.93	Hodgkin lymphoma, unspecified, intra-abdominal lymph nodes	Diagnosis	ICD-10-CM
C81.94	Hodgkin lymphoma, unspecified, lymph nodes of axilla and upper limb	Diagnosis	ICD-10-CM
C81.95	Hodgkin lymphoma, unspecified, lymph nodes of inguinal region and lower limb	Diagnosis	ICD-10-CM
C81.96	Hodgkin lymphoma, unspecified, intrapelvic lymph nodes	Diagnosis	ICD-10-CM
C81.97	Hodgkin lymphoma, unspecified, spleen	Diagnosis	ICD-10-CM
C81.98	Hodgkin lymphoma, unspecified, lymph nodes of multiple sites	Diagnosis	ICD-10-CM
C81.99	Hodgkin lymphoma, unspecified, extranodal and solid organ sites	Diagnosis	ICD-10-CM
C82.00	Follicular lymphoma grade I, unspecified site	Diagnosis	ICD-10-CM
C82.01	Follicular lymphoma grade I, lymph nodes of head, face, and neck	Diagnosis	ICD-10-CM
C82.02	Follicular lymphoma grade I, intrathoracic lymph nodes	Diagnosis	ICD-10-CM

Code	Description	Code Category	Code Type
C82.03	Follicular lymphoma grade I, intra-abdominal lymph nodes	Diagnosis	ICD-10-CM
C82.04	Follicular lymphoma grade I, lymph nodes of axilla and upper limb	Diagnosis	ICD-10-CM
C82.05	Follicular lymphoma grade I, lymph nodes of inguinal region and lower limb	Diagnosis	ICD-10-CM
C82.06	Follicular lymphoma grade I, intrapelvic lymph nodes	Diagnosis	ICD-10-CM
C82.07	Follicular lymphoma grade I, spleen	Diagnosis	ICD-10-CM
C82.08	Follicular lymphoma grade I, lymph nodes of multiple sites	Diagnosis	ICD-10-CM
C82.09	Follicular lymphoma grade I, extranodal and solid organ sites	Diagnosis	ICD-10-CM
C82.10	Follicular lymphoma grade II, unspecified site	Diagnosis	ICD-10-CM
C82.11	Follicular lymphoma grade II, lymph nodes of head, face, and neck	Diagnosis	ICD-10-CM
C82.12	Follicular lymphoma grade II, intrathoracic lymph nodes	Diagnosis	ICD-10-CM
C82.13	Follicular lymphoma grade II, intra-abdominal lymph nodes	Diagnosis	ICD-10-CM
C82.14	Follicular lymphoma grade II, lymph nodes of axilla and upper limb	Diagnosis	ICD-10-CM
C82.15	Follicular lymphoma grade II, lymph nodes of inguinal region and lower limb	Diagnosis	ICD-10-CM
C82.16	Follicular lymphoma grade II, intrapelvic lymph nodes	Diagnosis	ICD-10-CM
C82.17	Follicular lymphoma grade II, spleen	Diagnosis	ICD-10-CM
C82.18	Follicular lymphoma grade II, lymph nodes of multiple sites	Diagnosis	ICD-10-CM
C82.19	Follicular lymphoma grade II, extranodal and solid organ sites	Diagnosis	ICD-10-CM
C82.20	Follicular lymphoma grade III, unspecified, unspecified site	Diagnosis	ICD-10-CM
C82.21	Follicular lymphoma grade III, unspecified, lymph nodes of head, face, and neck	Diagnosis	ICD-10-CM
C82.22	Follicular lymphoma grade III, unspecified, intrathoracic lymph nodes	Diagnosis	ICD-10-CM
C82.23	Follicular lymphoma grade III, unspecified, intra-abdominal lymph nodes	Diagnosis	ICD-10-CM
C82.24	Follicular lymphoma grade III, unspecified, lymph nodes of axilla and upper limb	Diagnosis	ICD-10-CM
C82.25	Follicular lymphoma grade III, unspecified, lymph nodes of inguinal region and lower limb	Diagnosis	ICD-10-CM
C82.26	Follicular lymphoma grade III, unspecified, intrapelvic lymph nodes	Diagnosis	ICD-10-CM
C82.27	Follicular lymphoma grade III, unspecified, spleen	Diagnosis	ICD-10-CM
C82.28	Follicular lymphoma grade III, unspecified, lymph nodes of multiple sites	Diagnosis	ICD-10-CM
C82.29	Follicular lymphoma grade III, unspecified, extranodal and solid organ sites	Diagnosis	ICD-10-CM
C82.30	Follicular lymphoma grade IIIa, unspecified site	Diagnosis	ICD-10-CM
C82.31	Follicular lymphoma grade IIIa, lymph nodes of head, face, and neck	Diagnosis	ICD-10-CM
C82.32	Follicular lymphoma grade IIIa, intrathoracic lymph nodes	Diagnosis	ICD-10-CM
C82.33	Follicular lymphoma grade IIIa, intra-abdominal lymph nodes	Diagnosis	ICD-10-CM
C82.34	Follicular lymphoma grade IIIa, lymph nodes of axilla and upper limb	Diagnosis	ICD-10-CM
C82.35	Follicular lymphoma grade IIIa, lymph nodes of inguinal region and lower limb	Diagnosis	ICD-10-CM
C82.36	Follicular lymphoma grade IIIa, intrapelvic lymph nodes	Diagnosis	ICD-10-CM
C82.37	Follicular lymphoma grade IIIa, spleen	Diagnosis	ICD-10-CM
C82.38	Follicular lymphoma grade IIIa, lymph nodes of multiple sites	Diagnosis	ICD-10-CM
C82.39	Follicular lymphoma grade IIIa, extranodal and solid organ sites	Diagnosis	ICD-10-CM
C82.40	Follicular lymphoma grade IIIb, unspecified site	Diagnosis	ICD-10-CM
C82.41	Follicular lymphoma grade IIIb, lymph nodes of head, face, and neck	Diagnosis	ICD-10-CM
C82.42	Follicular lymphoma grade IIIb, intrathoracic lymph nodes	Diagnosis	ICD-10-CM
C82.43	Follicular lymphoma grade IIIb, intra-abdominal lymph nodes	Diagnosis	ICD-10-CM
C82.44	Follicular lymphoma grade IIIb, lymph nodes of axilla and upper limb	Diagnosis	ICD-10-CM
C82.45	Follicular lymphoma grade IIIb, lymph nodes of inguinal region and lower limb	Diagnosis	ICD-10-CM
C82.46	Follicular lymphoma grade IIIb, intrapelvic lymph nodes	Diagnosis	ICD-10-CM

Code	Description	Code Category	Code Type
C82.47	Follicular lymphoma grade IIIb, spleen	Diagnosis	ICD-10-CM
C82.48	Follicular lymphoma grade IIIb, lymph nodes of multiple sites	Diagnosis	ICD-10-CM
C82.49	Follicular lymphoma grade IIIb, extranodal and solid organ sites	Diagnosis	ICD-10-CM
C82.50	Diffuse follicle center lymphoma, unspecified site	Diagnosis	ICD-10-CM
C82.51	Diffuse follicle center lymphoma, lymph nodes of head, face, and neck	Diagnosis	ICD-10-CM
C82.52	Diffuse follicle center lymphoma, intrathoracic lymph nodes	Diagnosis	ICD-10-CM
C82.53	Diffuse follicle center lymphoma, intra-abdominal lymph nodes	Diagnosis	ICD-10-CM
C82.54	Diffuse follicle center lymphoma, lymph nodes of axilla and upper limb	Diagnosis	ICD-10-CM
C82.55	Diffuse follicle center lymphoma, lymph nodes of inguinal region and lower limb	Diagnosis	ICD-10-CM
C82.56	Diffuse follicle center lymphoma, intrapelvic lymph nodes	Diagnosis	ICD-10-CM
C82.57	Diffuse follicle center lymphoma, spleen	Diagnosis	ICD-10-CM
C82.58	Diffuse follicle center lymphoma, lymph nodes of multiple sites	Diagnosis	ICD-10-CM
C82.59	Diffuse follicle center lymphoma, extranodal and solid organ sites	Diagnosis	ICD-10-CM
C82.60	Cutaneous follicle center lymphoma, unspecified site	Diagnosis	ICD-10-CM
C82.61	Cutaneous follicle center lymphoma, lymph nodes of head, face, and neck	Diagnosis	ICD-10-CM
C82.62	Cutaneous follicle center lymphoma, intrathoracic lymph nodes	Diagnosis	ICD-10-CM
C82.63	Cutaneous follicle center lymphoma, intra-abdominal lymph nodes	Diagnosis	ICD-10-CM
C82.64	Cutaneous follicle center lymphoma, lymph nodes of axilla and upper limb	Diagnosis	ICD-10-CM
C82.65	Cutaneous follicle center lymphoma, lymph nodes of inguinal region and lower limb	Diagnosis	ICD-10-CM
C82.66	Cutaneous follicle center lymphoma, intrapelvic lymph nodes	Diagnosis	ICD-10-CM
C82.67	Cutaneous follicle center lymphoma, spleen	Diagnosis	ICD-10-CM
C82.68	Cutaneous follicle center lymphoma, lymph nodes of multiple sites	Diagnosis	ICD-10-CM
C82.69	Cutaneous follicle center lymphoma, extranodal and solid organ sites	Diagnosis	ICD-10-CM
C82.80	Other types of follicular lymphoma, unspecified site	Diagnosis	ICD-10-CM
C82.81	Other types of follicular lymphoma, lymph nodes of head, face, and neck	Diagnosis	ICD-10-CM
C82.82	Other types of follicular lymphoma, intrathoracic lymph nodes	Diagnosis	ICD-10-CM
C82.83	Other types of follicular lymphoma, intra-abdominal lymph nodes	Diagnosis	ICD-10-CM
C82.84	Other types of follicular lymphoma, lymph nodes of axilla and upper limb	Diagnosis	ICD-10-CM
C82.85	Other types of follicular lymphoma, lymph nodes of inguinal region and lower limb	Diagnosis	ICD-10-CM
C82.86	Other types of follicular lymphoma, intrapelvic lymph nodes	Diagnosis	ICD-10-CM
C82.87	Other types of follicular lymphoma, spleen	Diagnosis	ICD-10-CM
C82.88	Other types of follicular lymphoma, lymph nodes of multiple sites	Diagnosis	ICD-10-CM
C82.89	Other types of follicular lymphoma, extranodal and solid organ sites	Diagnosis	ICD-10-CM
C82.90	Follicular lymphoma, unspecified, unspecified site	Diagnosis	ICD-10-CM
C82.91	Follicular lymphoma, unspecified, lymph nodes of head, face, and neck	Diagnosis	ICD-10-CM
C82.92	Follicular lymphoma, unspecified, intrathoracic lymph nodes	Diagnosis	ICD-10-CM
C82.93	Follicular lymphoma, unspecified, intra-abdominal lymph nodes	Diagnosis	ICD-10-CM
C82.94	Follicular lymphoma, unspecified, lymph nodes of axilla and upper limb	Diagnosis	ICD-10-CM
C82.95	Follicular lymphoma, unspecified, lymph nodes of inguinal region and lower limb	Diagnosis	ICD-10-CM
C82.96	Follicular lymphoma, unspecified, intrapelvic lymph nodes	Diagnosis	ICD-10-CM
C82.97	Follicular lymphoma, unspecified, spleen	Diagnosis	ICD-10-CM
C82.98	Follicular lymphoma, unspecified, lymph nodes of multiple sites	Diagnosis	ICD-10-CM
C82.99	Follicular lymphoma, unspecified, extranodal and solid organ sites	Diagnosis	ICD-10-CM
C83.00	Small cell B-cell lymphoma, unspecified site	Diagnosis	ICD-10-CM
C83.01	Small cell B-cell lymphoma, lymph nodes of head, face, and neck	Diagnosis	ICD-10-CM

Code	Description	Code Category	Code Type
C83.02	Small cell B-cell lymphoma, intrathoracic lymph nodes	Diagnosis	ICD-10-CM
C83.03	Small cell B-cell lymphoma, intra-abdominal lymph nodes	Diagnosis	ICD-10-CM
C83.04	Small cell B-cell lymphoma, lymph nodes of axilla and upper limb	Diagnosis	ICD-10-CM
C83.05	Small cell B-cell lymphoma, lymph nodes of inguinal region and lower limb	Diagnosis	ICD-10-CM
C83.06	Small cell B-cell lymphoma, intrapelvic lymph nodes	Diagnosis	ICD-10-CM
C83.07	Small cell B-cell lymphoma, spleen	Diagnosis	ICD-10-CM
C83.08	Small cell B-cell lymphoma, lymph nodes of multiple sites	Diagnosis	ICD-10-CM
C83.09	Small cell B-cell lymphoma, extranodal and solid organ sites	Diagnosis	ICD-10-CM
C83.10	Mantle cell lymphoma, unspecified site	Diagnosis	ICD-10-CM
C83.11	Mantle cell lymphoma, lymph nodes of head, face, and neck	Diagnosis	ICD-10-CM
C83.12	Mantle cell lymphoma, intrathoracic lymph nodes	Diagnosis	ICD-10-CM
C83.13	Mantle cell lymphoma, intra-abdominal lymph nodes	Diagnosis	ICD-10-CM
C83.14	Mantle cell lymphoma, lymph nodes of axilla and upper limb	Diagnosis	ICD-10-CM
C83.15	Mantle cell lymphoma, lymph nodes of inguinal region and lower limb	Diagnosis	ICD-10-CM
C83.16	Mantle cell lymphoma, intrapelvic lymph nodes	Diagnosis	ICD-10-CM
C83.17	Mantle cell lymphoma, spleen	Diagnosis	ICD-10-CM
C83.18	Mantle cell lymphoma, lymph nodes of multiple sites	Diagnosis	ICD-10-CM
C83.19	Mantle cell lymphoma, extranodal and solid organ sites	Diagnosis	ICD-10-CM
C83.30	Diffuse large B-cell lymphoma, unspecified site	Diagnosis	ICD-10-CM
C83.31	Diffuse large B-cell lymphoma, lymph nodes of head, face, and neck	Diagnosis	ICD-10-CM
C83.32	Diffuse large B-cell lymphoma, intrathoracic lymph nodes	Diagnosis	ICD-10-CM
C83.33	Diffuse large B-cell lymphoma, intra-abdominal lymph nodes	Diagnosis	ICD-10-CM
C83.34	Diffuse large B-cell lymphoma, lymph nodes of axilla and upper limb	Diagnosis	ICD-10-CM
C83.35	Diffuse large B-cell lymphoma, lymph nodes of inguinal region and lower limb	Diagnosis	ICD-10-CM
C83.36	Diffuse large B-cell lymphoma, intrapelvic lymph nodes	Diagnosis	ICD-10-CM
C83.37	Diffuse large B-cell lymphoma, spleen	Diagnosis	ICD-10-CM
C83.38	Diffuse large B-cell lymphoma, lymph nodes of multiple sites	Diagnosis	ICD-10-CM
C83.39	Diffuse large B-cell lymphoma, extranodal and solid organ sites	Diagnosis	ICD-10-CM
C83.50	Lymphoblastic (diffuse) lymphoma, unspecified site	Diagnosis	ICD-10-CM
C83.51	Lymphoblastic (diffuse) lymphoma, lymph nodes of head, face, and neck	Diagnosis	ICD-10-CM
C83.52	Lymphoblastic (diffuse) lymphoma, intrathoracic lymph nodes	Diagnosis	ICD-10-CM
C83.53	Lymphoblastic (diffuse) lymphoma, intra-abdominal lymph nodes	Diagnosis	ICD-10-CM
C83.54	Lymphoblastic (diffuse) lymphoma, lymph nodes of axilla and upper limb	Diagnosis	ICD-10-CM
C83.55	Lymphoblastic (diffuse) lymphoma, lymph nodes of inguinal region and lower limb	Diagnosis	ICD-10-CM
C83.56	Lymphoblastic (diffuse) lymphoma, intrapelvic lymph nodes	Diagnosis	ICD-10-CM
C83.57	Lymphoblastic (diffuse) lymphoma, spleen	Diagnosis	ICD-10-CM
C83.58	Lymphoblastic (diffuse) lymphoma, lymph nodes of multiple sites	Diagnosis	ICD-10-CM
C83.59	Lymphoblastic (diffuse) lymphoma, extranodal and solid organ sites	Diagnosis	ICD-10-CM
C83.70	Burkitt lymphoma, unspecified site	Diagnosis	ICD-10-CM
C83.71	Burkitt lymphoma, lymph nodes of head, face, and neck	Diagnosis	ICD-10-CM
C83.72	Burkitt lymphoma, intrathoracic lymph nodes	Diagnosis	ICD-10-CM
C83.73	Burkitt lymphoma, intra-abdominal lymph nodes	Diagnosis	ICD-10-CM
C83.74	Burkitt lymphoma, lymph nodes of axilla and upper limb	Diagnosis	ICD-10-CM
C83.75	Burkitt lymphoma, lymph nodes of inguinal region and lower limb	Diagnosis	ICD-10-CM
C83.76	Burkitt lymphoma, intrapelvic lymph nodes	Diagnosis	ICD-10-CM

Code	Description	Code Category	Code Type
C83.77	Burkitt lymphoma, spleen	Diagnosis	ICD-10-CM
C83.78	Burkitt lymphoma, lymph nodes of multiple sites	Diagnosis	ICD-10-CM
C83.79	Burkitt lymphoma, extranodal and solid organ sites	Diagnosis	ICD-10-CM
C83.80	Other non-follicular lymphoma, unspecified site	Diagnosis	ICD-10-CM
C83.81	Other non-follicular lymphoma, lymph nodes of head, face, and neck	Diagnosis	ICD-10-CM
C83.82	Other non-follicular lymphoma, intrathoracic lymph nodes	Diagnosis	ICD-10-CM
C83.83	Other non-follicular lymphoma, intra-abdominal lymph nodes	Diagnosis	ICD-10-CM
C83.84	Other non-follicular lymphoma, lymph nodes of axilla and upper limb	Diagnosis	ICD-10-CM
C83.85	Other non-follicular lymphoma, lymph nodes of inguinal region and lower limb	Diagnosis	ICD-10-CM
C83.86	Other non-follicular lymphoma, intrapelvic lymph nodes	Diagnosis	ICD-10-CM
C83.87	Other non-follicular lymphoma, spleen	Diagnosis	ICD-10-CM
C83.88	Other non-follicular lymphoma, lymph nodes of multiple sites	Diagnosis	ICD-10-CM
C83.89	Other non-follicular lymphoma, extranodal and solid organ sites	Diagnosis	ICD-10-CM
C83.90	Non-follicular (diffuse) lymphoma, unspecified, unspecified site	Diagnosis	ICD-10-CM
C83.91	Non-follicular (diffuse) lymphoma, unspecified, lymph nodes of head, face, and neck	Diagnosis	ICD-10-CM
C83.92	Non-follicular (diffuse) lymphoma, unspecified, intrathoracic lymph nodes	Diagnosis	ICD-10-CM
C83.93	Non-follicular (diffuse) lymphoma, unspecified, intra-abdominal lymph nodes	Diagnosis	ICD-10-CM
C83.94	Non-follicular (diffuse) lymphoma, unspecified, lymph nodes of axilla and upper limb	Diagnosis	ICD-10-CM
C83.95	Non-follicular (diffuse) lymphoma, unspecified, lymph nodes of inguinal region and lower limb	Diagnosis	ICD-10-CM
C83.96	Non-follicular (diffuse) lymphoma, unspecified, intrapelvic lymph nodes	Diagnosis	ICD-10-CM
C83.97	Non-follicular (diffuse) lymphoma, unspecified, spleen	Diagnosis	ICD-10-CM
C83.98	Non-follicular (diffuse) lymphoma, unspecified, lymph nodes of multiple sites	Diagnosis	ICD-10-CM
C83.99	Non-follicular (diffuse) lymphoma, unspecified, extranodal and solid organ sites	Diagnosis	ICD-10-CM
C84.00	Mycosis fungoides, unspecified site	Diagnosis	ICD-10-CM
C84.01	Mycosis fungoides, lymph nodes of head, face, and neck	Diagnosis	ICD-10-CM
C84.02	Mycosis fungoides, intrathoracic lymph nodes	Diagnosis	ICD-10-CM
C84.03	Mycosis fungoides, intra-abdominal lymph nodes	Diagnosis	ICD-10-CM
C84.04	Mycosis fungoides, lymph nodes of axilla and upper limb	Diagnosis	ICD-10-CM
C84.05	Mycosis fungoides, lymph nodes of inguinal region and lower limb	Diagnosis	ICD-10-CM
C84.06	Mycosis fungoides, intrapelvic lymph nodes	Diagnosis	ICD-10-CM
C84.07	Mycosis fungoides, spleen	Diagnosis	ICD-10-CM
C84.08	Mycosis fungoides, lymph nodes of multiple sites	Diagnosis	ICD-10-CM
C84.09	Mycosis fungoides, extranodal and solid organ sites	Diagnosis	ICD-10-CM
C84.10	Sezary disease, unspecified site	Diagnosis	ICD-10-CM
C84.11	Sezary disease, lymph nodes of head, face, and neck	Diagnosis	ICD-10-CM
C84.12	Sezary disease, intrathoracic lymph nodes	Diagnosis	ICD-10-CM
C84.13	Sezary disease, intra-abdominal lymph nodes	Diagnosis	ICD-10-CM
C84.14	Sezary disease, lymph nodes of axilla and upper limb	Diagnosis	ICD-10-CM
C84.15	Sezary disease, lymph nodes of inguinal region and lower limb	Diagnosis	ICD-10-CM
C84.16	Sezary disease, intrapelvic lymph nodes	Diagnosis	ICD-10-CM
C84.17	Sezary disease, spleen	Diagnosis	ICD-10-CM
C84.18	Sezary disease, lymph nodes of multiple sites	Diagnosis	ICD-10-CM
C84.19	Sezary disease, extranodal and solid organ sites	Diagnosis	ICD-10-CM
C84.40	Peripheral T-cell lymphoma, not classified, unspecified site	Diagnosis	ICD-10-CM

Code	Description	Code Category	Code Type
C84.41	Peripheral T-cell lymphoma, not classified, lymph nodes of head, face, and neck	Diagnosis	ICD-10-CM
C84.42	Peripheral T-cell lymphoma, not classified, intrathoracic lymph nodes	Diagnosis	ICD-10-CM
C84.43	Peripheral T-cell lymphoma, not classified, intra-abdominal lymph nodes	Diagnosis	ICD-10-CM
C84.44	Peripheral T-cell lymphoma, not classified, lymph nodes of axilla and upper limb	Diagnosis	ICD-10-CM
C84.45	Peripheral T-cell lymphoma, not classified, lymph nodes of inguinal region and lower limb	Diagnosis	ICD-10-CM
C84.46	Peripheral T-cell lymphoma, not classified, intrapelvic lymph nodes	Diagnosis	ICD-10-CM
C84.47	Peripheral T-cell lymphoma, not classified, spleen	Diagnosis	ICD-10-CM
C84.48	Peripheral T-cell lymphoma, not classified, lymph nodes of multiple sites	Diagnosis	ICD-10-CM
C84.49	Peripheral T-cell lymphoma, not classified, extranodal and solid organ sites	Diagnosis	ICD-10-CM
C84.60	Anaplastic large cell lymphoma, ALK-positive, unspecified site	Diagnosis	ICD-10-CM
C84.61	Anaplastic large cell lymphoma, ALK-positive, lymph nodes of head, face, and neck	Diagnosis	ICD-10-CM
C84.62	Anaplastic large cell lymphoma, ALK-positive, intrathoracic lymph nodes	Diagnosis	ICD-10-CM
C84.63	Anaplastic large cell lymphoma, ALK-positive, intra-abdominal lymph nodes	Diagnosis	ICD-10-CM
C84.64	Anaplastic large cell lymphoma, ALK-positive, lymph nodes of axilla and upper limb	Diagnosis	ICD-10-CM
C84.65	Anaplastic large cell lymphoma, ALK-positive, lymph nodes of inguinal region and lower limb	Diagnosis	ICD-10-CM
C84.66	Anaplastic large cell lymphoma, ALK-positive, intrapelvic lymph nodes	Diagnosis	ICD-10-CM
C84.67	Anaplastic large cell lymphoma, ALK-positive, spleen	Diagnosis	ICD-10-CM
C84.68	Anaplastic large cell lymphoma, ALK-positive, lymph nodes of multiple sites	Diagnosis	ICD-10-CM
C84.69	Anaplastic large cell lymphoma, ALK-positive, extranodal and solid organ sites	Diagnosis	ICD-10-CM
C84.70	Anaplastic large cell lymphoma, ALK-negative, unspecified site	Diagnosis	ICD-10-CM
C84.71	Anaplastic large cell lymphoma, ALK-negative, lymph nodes of head, face, and neck	Diagnosis	ICD-10-CM
C84.72	Anaplastic large cell lymphoma, ALK-negative, intrathoracic lymph nodes	Diagnosis	ICD-10-CM
C84.73	Anaplastic large cell lymphoma, ALK-negative, intra-abdominal lymph nodes	Diagnosis	ICD-10-CM
C84.74	Anaplastic large cell lymphoma, ALK-negative, lymph nodes of axilla and upper limb	Diagnosis	ICD-10-CM
C84.75	Anaplastic large cell lymphoma, ALK-negative, lymph nodes of inguinal region and lower limb	Diagnosis	ICD-10-CM
C84.76	Anaplastic large cell lymphoma, ALK-negative, intrapelvic lymph nodes	Diagnosis	ICD-10-CM
C84.77	Anaplastic large cell lymphoma, ALK-negative, spleen	Diagnosis	ICD-10-CM
C84.78	Anaplastic large cell lymphoma, ALK-negative, lymph nodes of multiple sites	Diagnosis	ICD-10-CM
C84.79	Anaplastic large cell lymphoma, ALK-negative, extranodal and solid organ sites	Diagnosis	ICD-10-CM
C84.90	Mature T/NK-cell lymphomas, unspecified, unspecified site	Diagnosis	ICD-10-CM
C84.91	Mature T/NK-cell lymphomas, unspecified, lymph nodes of head, face, and neck	Diagnosis	ICD-10-CM
C84.92	Mature T/NK-cell lymphomas, unspecified, intrathoracic lymph nodes	Diagnosis	ICD-10-CM
C84.93	Mature T/NK-cell lymphomas, unspecified, intra-abdominal lymph nodes	Diagnosis	ICD-10-CM
C84.94	Mature T/NK-cell lymphomas, unspecified, lymph nodes of axilla and upper limb	Diagnosis	ICD-10-CM
C84.95	Mature T/NK-cell lymphomas, unspecified, lymph nodes of inguinal region and lower limb	Diagnosis	ICD-10-CM
C84.96	Mature T/NK-cell lymphomas, unspecified, intrapelvic lymph nodes	Diagnosis	ICD-10-CM
C84.97	Mature T/NK-cell lymphomas, unspecified, spleen	Diagnosis	ICD-10-CM
C84.98	Mature T/NK-cell lymphomas, unspecified, lymph nodes of multiple sites	Diagnosis	ICD-10-CM
C84.99	Mature T/NK-cell lymphomas, unspecified, extranodal and solid organ sites	Diagnosis	ICD-10-CM
C84.A0	Cutaneous T-cell lymphoma, unspecified, unspecified site	Diagnosis	ICD-10-CM
C84.A1	Cutaneous T-cell lymphoma, unspecified lymph nodes of head, face, and neck	Diagnosis	ICD-10-CM

Code	Description	Code Category	Code Type
C84.A2	Cutaneous T-cell lymphoma, unspecified, intrathoracic lymph nodes	Diagnosis	ICD-10-CM
C84.A3	Cutaneous T-cell lymphoma, unspecified, intra-abdominal lymph nodes	Diagnosis	ICD-10-CM
C84.A4	Cutaneous T-cell lymphoma, unspecified, lymph nodes of axilla and upper limb	Diagnosis	ICD-10-CM
C84.A5	Cutaneous T-cell lymphoma, unspecified, lymph nodes of inguinal region and lower limb	Diagnosis	ICD-10-CM
C84.A6	Cutaneous T-cell lymphoma, unspecified, intrapelvic lymph nodes	Diagnosis	ICD-10-CM
C84.A7	Cutaneous T-cell lymphoma, unspecified, spleen	Diagnosis	ICD-10-CM
C84.A8	Cutaneous T-cell lymphoma, unspecified, lymph nodes of multiple sites	Diagnosis	ICD-10-CM
C84.A9	Cutaneous T-cell lymphoma, unspecified, extranodal and solid organ sites	Diagnosis	ICD-10-CM
C84.Z0	Other mature T/NK-cell lymphomas, unspecified site	Diagnosis	ICD-10-CM
C84.Z1	Other mature T/NK-cell lymphomas, lymph nodes of head, face, and neck	Diagnosis	ICD-10-CM
C84.Z2	Other mature T/NK-cell lymphomas, intrathoracic lymph nodes	Diagnosis	ICD-10-CM
C84.Z3	Other mature T/NK-cell lymphomas, intra-abdominal lymph nodes	Diagnosis	ICD-10-CM
C84.Z4	Other mature T/NK-cell lymphomas, lymph nodes of axilla and upper limb	Diagnosis	ICD-10-CM
C84.Z5	Other mature T/NK-cell lymphomas, lymph nodes of inguinal region and lower limb	Diagnosis	ICD-10-CM
C84.Z6	Other mature T/NK-cell lymphomas, intrapelvic lymph nodes	Diagnosis	ICD-10-CM
C84.Z7	Other mature T/NK-cell lymphomas, spleen	Diagnosis	ICD-10-CM
C84.Z8	Other mature T/NK-cell lymphomas, lymph nodes of multiple sites	Diagnosis	ICD-10-CM
C84.Z9	Other mature T/NK-cell lymphomas, extranodal and solid organ sites	Diagnosis	ICD-10-CM
C85.10	Unspecified B-cell lymphoma, unspecified site	Diagnosis	ICD-10-CM
C85.11	Unspecified B-cell lymphoma, lymph nodes of head, face, and neck	Diagnosis	ICD-10-CM
C85.12	Unspecified B-cell lymphoma, intrathoracic lymph nodes	Diagnosis	ICD-10-CM
C85.13	Unspecified B-cell lymphoma, intra-abdominal lymph nodes	Diagnosis	ICD-10-CM
C85.14	Unspecified B-cell lymphoma, lymph nodes of axilla and upper limb	Diagnosis	ICD-10-CM
C85.15	Unspecified B-cell lymphoma, lymph nodes of inguinal region and lower limb	Diagnosis	ICD-10-CM
C85.16	Unspecified B-cell lymphoma, intrapelvic lymph nodes	Diagnosis	ICD-10-CM
C85.17	Unspecified B-cell lymphoma, spleen	Diagnosis	ICD-10-CM
C85.18	Unspecified B-cell lymphoma, lymph nodes of multiple sites	Diagnosis	ICD-10-CM
C85.19	Unspecified B-cell lymphoma, extranodal and solid organ sites	Diagnosis	ICD-10-CM
C85.20	Mediastinal (thymic) large B-cell lymphoma, unspecified site	Diagnosis	ICD-10-CM
C85.21	Mediastinal (thymic) large B-cell lymphoma, lymph nodes of head, face, and neck	Diagnosis	ICD-10-CM
C85.22	Mediastinal (thymic) large B-cell lymphoma, intrathoracic lymph nodes	Diagnosis	ICD-10-CM
C85.23	Mediastinal (thymic) large B-cell lymphoma, intra-abdominal lymph nodes	Diagnosis	ICD-10-CM
C85.24	Mediastinal (thymic) large B-cell lymphoma, lymph nodes of axilla and upper limb	Diagnosis	ICD-10-CM
C85.25	Mediastinal (thymic) large B-cell lymphoma, lymph nodes of inguinal region and lower limb	Diagnosis	ICD-10-CM
C85.26	Mediastinal (thymic) large B-cell lymphoma, intrapelvic lymph nodes	Diagnosis	ICD-10-CM
C85.27	Mediastinal (thymic) large B-cell lymphoma, spleen	Diagnosis	ICD-10-CM
C85.28	Mediastinal (thymic) large B-cell lymphoma, lymph nodes of multiple sites	Diagnosis	ICD-10-CM
C85.29	Mediastinal (thymic) large B-cell lymphoma, extranodal and solid organ sites	Diagnosis	ICD-10-CM
C85.80	Other specified types of non-Hodgkin lymphoma, unspecified site	Diagnosis	ICD-10-CM
C85.81	Other specified types of non-Hodgkin lymphoma, lymph nodes of head, face, and ne	Diagnosis	ICD-10-CM
C85.82	Other specified types of non-Hodgkin lymphoma, intrathoracic lymph nodes	Diagnosis	ICD-10-CM
C85.83	Other specified types of non-Hodgkin lymphoma, intra-abdominal lymph nodes	Diagnosis	ICD-10-CM
C85.84	Other specified types of non-Hodgkin lymphoma, lymph nodes of axilla and upper li	Diagnosis	ICD-10-CM

Code	Description	Code Category	Code Type
C85.85	Other specified types of non-Hodgkin lymphoma, lymph nodes of inguinal region and lower limb	Diagnosis	ICD-10-CM
C85.86	Other specified types of non-Hodgkin lymphoma, intrapelvic lymph nodes	Diagnosis	ICD-10-CM
C85.87	Other specified types of non-Hodgkin lymphoma, spleen	Diagnosis	ICD-10-CM
C85.88	Other specified types of non-Hodgkin lymphoma, lymph nodes of multiple sites	Diagnosis	ICD-10-CM
C85.89	Other specified types of non-Hodgkin lymphoma, extranodal and solid organ sites	Diagnosis	ICD-10-CM
C85.90	Non-Hodgkin lymphoma, unspecified, unspecified site	Diagnosis	ICD-10-CM
C85.91	Non-Hodgkin lymphoma, unspecified, lymph nodes of head, face, and neck	Diagnosis	ICD-10-CM
C85.92	Non-Hodgkin lymphoma, unspecified, intrathoracic lymph nodes	Diagnosis	ICD-10-CM
C85.93	Non-Hodgkin lymphoma, unspecified, intra-abdominal lymph nodes	Diagnosis	ICD-10-CM
C85.94	Non-Hodgkin lymphoma, unspecified, lymph nodes of axilla and upper limb	Diagnosis	ICD-10-CM
C85.95	Non-Hodgkin lymphoma, unspecified, lymph nodes of inguinal region and lower liml	Diagnosis	ICD-10-CM
C85.96	Non-Hodgkin lymphoma, unspecified, intrapelvic lymph nodes	Diagnosis	ICD-10-CM
C85.97	Non-Hodgkin lymphoma, unspecified, spleen	Diagnosis	ICD-10-CM
C85.98	Non-Hodgkin lymphoma, unspecified, lymph nodes of multiple sites	Diagnosis	ICD-10-CM
C85.99	Non-Hodgkin lymphoma, unspecified, extranodal and solid organ sites	Diagnosis	ICD-10-CM
C86.0	Extranodal NK/T-cell lymphoma, nasal type	Diagnosis	ICD-10-CM
C86.1	Hepatosplenic T-cell lymphoma	Diagnosis	ICD-10-CM
C86.2	Enteropathy-type (intestinal) T-cell lymphoma	Diagnosis	ICD-10-CM
C86.3	Subcutaneous panniculitis-like T-cell lymphoma	Diagnosis	ICD-10-CM
C86.4	Blastic NK-cell lymphoma	Diagnosis	ICD-10-CM
C86.5	Angioimmunoblastic T-cell lymphoma	Diagnosis	ICD-10-CM
C86.6	Primary cutaneous CD30-positive T-cell proliferations	Diagnosis	ICD-10-CM
C88.2	Heavy chain disease	Diagnosis	ICD-10-CM
C88.3	Immunoproliferative small intestinal disease	Diagnosis	ICD-10-CM
C88.4	Extranodal marginal zone B-cell lymphoma of mucosa-associated lymphoid tissue [MALT-lymphoma]	Diagnosis	ICD-10-CM
C88.8	Other malignant immunoproliferative diseases	Diagnosis	ICD-10-CM
C88.9	Malignant immunoproliferative disease, unspecified	Diagnosis	ICD-10-CM
C90.00	Multiple myeloma not having achieved remission	Diagnosis	ICD-10-CM
C90.01	Multiple myeloma in remission	Diagnosis	ICD-10-CM
C90.02	Multiple myeloma in relapse	Diagnosis	ICD-10-CM
C90.10	Plasma cell leukemia not having achieved remission	Diagnosis	ICD-10-CM
C90.11	Plasma cell leukemia in remission	Diagnosis	ICD-10-CM
C90.12	Plasma cell leukemia in relapse	Diagnosis	ICD-10-CM
C90.20	Extramedullary plasmacytoma not having achieved remission	Diagnosis	ICD-10-CM
C90.21	Extramedullary plasmacytoma in remission	Diagnosis	ICD-10-CM
C90.22	Extramedullary plasmacytoma in relapse	Diagnosis	ICD-10-CM
C90.30	Solitary plasmacytoma not having achieved remission	Diagnosis	ICD-10-CM
C90.31	Solitary plasmacytoma in remission	Diagnosis	ICD-10-CM
C90.32	Solitary plasmacytoma in relapse	Diagnosis	ICD-10-CM
C91.00	Acute lymphoblastic leukemia not having achieved remission	Diagnosis	ICD-10-CM
C91.01	Acute lymphoblastic leukemia, in remission	Diagnosis	ICD-10-CM
C91.02	Acute lymphoblastic leukemia, in relapse	Diagnosis	ICD-10-CM
C91.10	Chronic lymphocytic leukemia of B-cell type not having achieved remission	Diagnosis	ICD-10-CM

Code	Description	Code Category	Code Type
C91.11	Chronic lymphocytic leukemia of B-cell type in remission	Diagnosis	ICD-10-CM
C91.12	Chronic lymphocytic leukemia of B-cell type in relapse	Diagnosis	ICD-10-CM
C91.30	Prolymphocytic leukemia of B-cell type not having achieved remission	Diagnosis	ICD-10-CM
C91.31	Prolymphocytic leukemia of B-cell type, in remission	Diagnosis	ICD-10-CM
C91.32	Prolymphocytic leukemia of B-cell type, in relapse	Diagnosis	ICD-10-CM
C91.40	Hairy cell leukemia not having achieved remission	Diagnosis	ICD-10-CM
C91.41	Hairy cell leukemia, in remission	Diagnosis	ICD-10-CM
C91.42	Hairy cell leukemia, in relapse	Diagnosis	ICD-10-CM
C91.50	Adult T-cell lymphoma/leukemia (HTLV-1-associated) not having achieved remission	Diagnosis	ICD-10-CM
C91.51	Adult T-cell lymphoma/leukemia (HTLV-1-associated), in remission	Diagnosis	ICD-10-CM
C91.52	Adult T-cell lymphoma/leukemia (HTLV-1-associated), in relapse	Diagnosis	ICD-10-CM
C91.60	Prolymphocytic leukemia of T-cell type not having achieved remission	Diagnosis	ICD-10-CM
C91.61	Prolymphocytic leukemia of T-cell type, in remission	Diagnosis	ICD-10-CM
C91.62	Prolymphocytic leukemia of T-cell type, in relapse	Diagnosis	ICD-10-CM
C91.90	Lymphoid leukemia, unspecified not having achieved remission	Diagnosis	ICD-10-CM
C91.91	Lymphoid leukemia, unspecified, in remission	Diagnosis	ICD-10-CM
C91.92	Lymphoid leukemia, unspecified, in relapse	Diagnosis	ICD-10-CM
C91.A0	Mature B-cell leukemia Burkitt-type not having achieved remission	Diagnosis	ICD-10-CM
C91.A1	Mature B-cell leukemia Burkitt-type, in remission	Diagnosis	ICD-10-CM
C91.A2	Mature B-cell leukemia Burkitt-type, in relapse	Diagnosis	ICD-10-CM
C91.Z0	Other lymphoid leukemia not having achieved remission	Diagnosis	ICD-10-CM
C91.Z1	Other lymphoid leukemia, in remission	Diagnosis	ICD-10-CM
C91.Z2	Other lymphoid leukemia, in relapse	Diagnosis	ICD-10-CM
C92.00	Acute myeloblastic leukemia, not having achieved remission	Diagnosis	ICD-10-CM
C92.01	Acute myeloblastic leukemia, in remission	Diagnosis	ICD-10-CM
C92.02	Acute myeloblastic leukemia, in relapse	Diagnosis	ICD-10-CM
C92.10	Chronic myeloid leukemia, BCR/ABL-positive, not having achieved remission	Diagnosis	ICD-10-CM
C92.11	Chronic myeloid leukemia, BCR/ABL-positive, in remission	Diagnosis	ICD-10-CM
C92.12	Chronic myeloid leukemia, BCR/ABL-positive, in relapse	Diagnosis	ICD-10-CM
C92.20	Atypical chronic myeloid leukemia, BCR/ABL-negative, not having achieved remissio	Diagnosis	ICD-10-CM
C92.21	Atypical chronic myeloid leukemia, BCR/ABL-negative, in remission	Diagnosis	ICD-10-CM
C92.22	Atypical chronic myeloid leukemia, BCR/ABL-negative, in relapse	Diagnosis	ICD-10-CM
C92.30	Myeloid sarcoma, not having achieved remission	Diagnosis	ICD-10-CM
C92.31	Myeloid sarcoma, in remission	Diagnosis	ICD-10-CM
C92.32	Myeloid sarcoma, in relapse	Diagnosis	ICD-10-CM
C92.40	Acute promyelocytic leukemia, not having achieved remission	Diagnosis	ICD-10-CM
C92.41	Acute promyelocytic leukemia, in remission	Diagnosis	ICD-10-CM
C92.42	Acute promyelocytic leukemia, in relapse	Diagnosis	ICD-10-CM
C92.50	Acute myelomonocytic leukemia, not having achieved remission	Diagnosis	ICD-10-CM
C92.51	Acute myelomonocytic leukemia, in remission	Diagnosis	ICD-10-CM
C92.52	Acute myelomonocytic leukemia, in relapse	Diagnosis	ICD-10-CM
C92.60	Acute myeloid leukemia with 11q23-abnormality not having achieved remission	Diagnosis	ICD-10-CM
C92.61	Acute myeloid leukemia with 11q23-abnormality in remission	Diagnosis	ICD-10-CM
C92.62	Acute myeloid leukemia with 11q23-abnormality in relapse	Diagnosis	ICD-10-CM
C92.90	Myeloid leukemia, unspecified, not having achieved remission	Diagnosis	ICD-10-CM

Code	Description	Code Category	Code Type
C92.91	Myeloid leukemia, unspecified in remission	Diagnosis	ICD-10-CM
C92.92	Myeloid leukemia, unspecified in relapse	Diagnosis	ICD-10-CM
C92.A0	Acute myeloid leukemia with multilineage dysplasia, not having achieved remission	Diagnosis	ICD-10-CM
C92.A1	Acute myeloid leukemia with multilineage dysplasia, in remission	Diagnosis	ICD-10-CM
C92.A2	Acute myeloid leukemia with multilineage dysplasia, in relapse	Diagnosis	ICD-10-CM
C92.Z0	Other myeloid leukemia not having achieved remission	Diagnosis	ICD-10-CM
C92.Z1	Other myeloid leukemia, in remission	Diagnosis	ICD-10-CM
C92.Z2	Other myeloid leukemia, in relapse	Diagnosis	ICD-10-CM
C93.00	Acute monoblastic/monocytic leukemia, not having achieved remission	Diagnosis	ICD-10-CM
C93.01	Acute monoblastic/monocytic leukemia, in remission	Diagnosis	ICD-10-CM
C93.02	Acute monoblastic/monocytic leukemia, in relapse	Diagnosis	ICD-10-CM
C93.10	Chronic myelomonocytic leukemia not having achieved remission	Diagnosis	ICD-10-CM
C93.11	Chronic myelomonocytic leukemia, in remission	Diagnosis	ICD-10-CM
C93.12	Chronic myelomonocytic leukemia, in relapse	Diagnosis	ICD-10-CM
C93.30	Juvenile myelomonocytic leukemia, not having achieved remission	Diagnosis	ICD-10-CM
C93.31	Juvenile myelomonocytic leukemia, in remission	Diagnosis	ICD-10-CM
C93.32	Juvenile myelomonocytic leukemia, in relapse	Diagnosis	ICD-10-CM
C93.90	Monocytic leukemia, unspecified, not having achieved remission	Diagnosis	ICD-10-CM
C93.91	Monocytic leukemia, unspecified in remission	Diagnosis	ICD-10-CM
C93.92	Monocytic leukemia, unspecified in relapse	Diagnosis	ICD-10-CM
C93.Z0	Other monocytic leukemia, not having achieved remission	Diagnosis	ICD-10-CM
C93.Z1	Other monocytic leukemia, in remission	Diagnosis	ICD-10-CM
C93.Z2	Other monocytic leukemia, in relapse	Diagnosis	ICD-10-CM
C94.00	Acute erythroid leukemia, not having achieved remission	Diagnosis	ICD-10-CM
C94.01	Acute erythroid leukemia, in remission	Diagnosis	ICD-10-CM
C94.02	Acute erythroid leukemia, in relapse	Diagnosis	ICD-10-CM
C94.20	Acute megakaryoblastic leukemia not having achieved remission	Diagnosis	ICD-10-CM
C94.21	Acute megakaryoblastic leukemia, in remission	Diagnosis	ICD-10-CM
C94.22	Acute megakaryoblastic leukemia, in relapse	Diagnosis	ICD-10-CM
C94.30	Mast cell leukemia not having achieved remission	Diagnosis	ICD-10-CM
C94.31	Mast cell leukemia, in remission	Diagnosis	ICD-10-CM
C94.32	Mast cell leukemia, in relapse	Diagnosis	ICD-10-CM
C94.80	Other specified leukemias not having achieved remission	Diagnosis	ICD-10-CM
C94.81	Other specified leukemias, in remission	Diagnosis	ICD-10-CM
C94.82	Other specified leukemias, in relapse	Diagnosis	ICD-10-CM
C95.00	Acute leukemia of unspecified cell type not having achieved remission	Diagnosis	ICD-10-CM
C95.01	Acute leukemia of unspecified cell type, in remission	Diagnosis	ICD-10-CM
C95.02	Acute leukemia of unspecified cell type, in relapse	Diagnosis	ICD-10-CM
C95.10	Chronic leukemia of unspecified cell type not having achieved remission	Diagnosis	ICD-10-CM
C95.11	Chronic leukemia of unspecified cell type, in remission	Diagnosis	ICD-10-CM
C95.12	Chronic leukemia of unspecified cell type, in relapse	Diagnosis	ICD-10-CM
C95.90	Leukemia, unspecified not having achieved remission	Diagnosis	ICD-10-CM
C95.91	Leukemia, unspecified, in remission	Diagnosis	ICD-10-CM
C95.92	Leukemia, unspecified, in relapse	Diagnosis	ICD-10-CM
C96.0	Multifocal and multisystemic (disseminated) Langerhans-cell histiocytosis	Diagnosis	ICD-10-CM

Code	Description	Code Category	Code Type
C96.20	Malignant mast cell neoplasm, unspecified	Diagnosis	ICD-10-CM
C96.21	Aggressive systemic mastocytosis	Diagnosis	ICD-10-CM
C96.22	Mast cell sarcoma	Diagnosis	ICD-10-CM
C96.29	Other malignant mast cell neoplasm	Diagnosis	ICD-10-CM
C96.4	Sarcoma of dendritic cells (accessory cells)	Diagnosis	ICD-10-CM
C96.9	Malignant neoplasm of lymphoid, hematopoietic and related tissue, unspecified	Diagnosis	ICD-10-CM
C96.A	Histiocytic sarcoma	Diagnosis	ICD-10-CM
C96.Z	Other specified malignant neoplasms of lymphoid, hematopoietic and related tissue	Diagnosis	ICD-10-CM
D03.0	Melanoma in situ of lip	Diagnosis	ICD-10-CM
D03.10	Melanoma in situ of unspecified eyelid, including canthus	Diagnosis	ICD-10-CM
D03.11	Melanoma in situ of right eyelid, including canthus	Diagnosis	ICD-10-CM
D03.12	Melanoma in situ of left eyelid, including canthus	Diagnosis	ICD-10-CM
D03.20	Melanoma in situ of unspecified ear and external auricular canal	Diagnosis	ICD-10-CM
D03.21	Melanoma in situ of right ear and external auricular canal	Diagnosis	ICD-10-CM
D03.22	Melanoma in situ of left ear and external auricular canal	Diagnosis	ICD-10-CM
D03.30	Melanoma in situ of unspecified part of face	Diagnosis	ICD-10-CM
D03.39	Melanoma in situ of other parts of face	Diagnosis	ICD-10-CM
D03.4	Melanoma in situ of scalp and neck	Diagnosis	ICD-10-CM
D03.51	Melanoma in situ of anal skin	Diagnosis	ICD-10-CM
D03.52	Melanoma in situ of breast (skin) (soft tissue)	Diagnosis	ICD-10-CM
D03.59	Melanoma in situ of other part of trunk	Diagnosis	ICD-10-CM
D03.60	Melanoma in situ of unspecified upper limb, including shoulder	Diagnosis	ICD-10-CM
D03.61	Melanoma in situ of right upper limb, including shoulder	Diagnosis	ICD-10-CM
D03.62	Melanoma in situ of left upper limb, including shoulder	Diagnosis	ICD-10-CM
D03.70	Melanoma in situ of unspecified lower limb, including hip	Diagnosis	ICD-10-CM
D03.71	Melanoma in situ of right lower limb, including hip	Diagnosis	ICD-10-CM
D03.72	Melanoma in situ of left lower limb, including hip	Diagnosis	ICD-10-CM
D03.8	Melanoma in situ of other sites	Diagnosis	ICD-10-CM
D03.9	Melanoma in situ, unspecified	Diagnosis	ICD-10-CM
D45	Polycythemia vera	Diagnosis	ICD-10-CM
D37.01	Neoplasm of uncertain behavior of lip	Diagnosis	ICD-10-CM
D37.02	Neoplasm of uncertain behavior of tongue	Diagnosis	ICD-10-CM
D37.030	Neoplasm of uncertain behavior of the parotid salivary glands	Diagnosis	ICD-10-CM
D37.031	Neoplasm of uncertain behavior of the sublingual salivary glands	Diagnosis	ICD-10-CM
D37.032	Neoplasm of uncertain behavior of the submandibular salivary glands	Diagnosis	ICD-10-CM
D37.039	Neoplasm of uncertain behavior of the major salivary glands, unspecified	Diagnosis	ICD-10-CM
D37.04	Neoplasm of uncertain behavior of the minor salivary glands	Diagnosis	ICD-10-CM
D37.05	Neoplasm of uncertain behavior of pharynx	Diagnosis	ICD-10-CM
D37.09	Neoplasm of uncertain behavior of other specified sites of the oral cavity	Diagnosis	ICD-10-CM
D37.1	Neoplasm of uncertain behavior of stomach	Diagnosis	ICD-10-CM
D37.2	Neoplasm of uncertain behavior of small intestine	Diagnosis	ICD-10-CM
D37.3	Neoplasm of uncertain behavior of appendix	Diagnosis	ICD-10-CM
D37.4	Neoplasm of uncertain behavior of colon	Diagnosis	ICD-10-CM
D37.5	Neoplasm of uncertain behavior of rectum	Diagnosis	ICD-10-CM
D37.6	Neoplasm of uncertain behavior of liver, gallbladder and bile ducts	Diagnosis	ICD-10-CM

Code	Description	Code Category	Code Type
D37.8	Neoplasm of uncertain behavior of other specified digestive organs	Diagnosis	ICD-10-CM
D37.9	Neoplasm of uncertain behavior of digestive organ, unspecified	Diagnosis	ICD-10-CM
D38.0	Neoplasm of uncertain behavior of larynx	Diagnosis	ICD-10-CM
D38.1	Neoplasm of uncertain behavior of trachea, bronchus and lung	Diagnosis	ICD-10-CM
D38.2	Neoplasm of uncertain behavior of pleura	Diagnosis	ICD-10-CM
D38.3	Neoplasm of uncertain behavior of mediastinum	Diagnosis	ICD-10-CM
D38.4	Neoplasm of uncertain behavior of thymus	Diagnosis	ICD-10-CM
D38.5	Neoplasm of uncertain behavior of other respiratory organs	Diagnosis	ICD-10-CM
D38.6	Neoplasm of uncertain behavior of respiratory organ, unspecified	Diagnosis	ICD-10-CM
D39.0	Neoplasm of uncertain behavior of uterus	Diagnosis	ICD-10-CM
D39.10	Neoplasm of uncertain behavior of unspecified ovary	Diagnosis	ICD-10-CM
D39.11	Neoplasm of uncertain behavior of right ovary	Diagnosis	ICD-10-CM
D39.12	Neoplasm of uncertain behavior of left ovary	Diagnosis	ICD-10-CM
D39.2	Neoplasm of uncertain behavior of placenta	Diagnosis	ICD-10-CM
D39.8	Neoplasm of uncertain behavior of other specified female genital organs	Diagnosis	ICD-10-CM
D39.9	Neoplasm of uncertain behavior of female genital organ, unspecified	Diagnosis	ICD-10-CM
D40.0	Neoplasm of uncertain behavior of prostate	Diagnosis	ICD-10-CM
D40.10	Neoplasm of uncertain behavior of unspecified testis	Diagnosis	ICD-10-CM
D40.11	Neoplasm of uncertain behavior of right testis	Diagnosis	ICD-10-CM
D40.12	Neoplasm of uncertain behavior of left testis	Diagnosis	ICD-10-CM
D40.8	Neoplasm of uncertain behavior of other specified male genital organs	Diagnosis	ICD-10-CM
D40.9	Neoplasm of uncertain behavior of male genital organ, unspecified	Diagnosis	ICD-10-CM
D41.00	Neoplasm of uncertain behavior of unspecified kidney	Diagnosis	ICD-10-CM
D41.01	Neoplasm of uncertain behavior of right kidney	Diagnosis	ICD-10-CM
D41.02	Neoplasm of uncertain behavior of left kidney	Diagnosis	ICD-10-CM
D41.10	Neoplasm of uncertain behavior of unspecified renal pelvis	Diagnosis	ICD-10-CM
D41.11	Neoplasm of uncertain behavior of right renal pelvis	Diagnosis	ICD-10-CM
D41.12	Neoplasm of uncertain behavior of left renal pelvis	Diagnosis	ICD-10-CM
D41.20	Neoplasm of uncertain behavior of unspecified ureter	Diagnosis	ICD-10-CM
D41.21	Neoplasm of uncertain behavior of right ureter	Diagnosis	ICD-10-CM
D41.22	Neoplasm of uncertain behavior of left ureter	Diagnosis	ICD-10-CM
D41.3	Neoplasm of uncertain behavior of urethra	Diagnosis	ICD-10-CM
D41.4	Neoplasm of uncertain behavior of bladder	Diagnosis	ICD-10-CM
D41.8	Neoplasm of uncertain behavior of other specified urinary organs	Diagnosis	ICD-10-CM
D41.9	Neoplasm of uncertain behavior of unspecified urinary organ	Diagnosis	ICD-10-CM
D42.0	Neoplasm of uncertain behavior of cerebral meninges	Diagnosis	ICD-10-CM
D42.1	Neoplasm of uncertain behavior of spinal meninges	Diagnosis	ICD-10-CM
D42.9	Neoplasm of uncertain behavior of meninges, unspecified	Diagnosis	ICD-10-CM
D43.0	Neoplasm of uncertain behavior of brain, supratentorial	Diagnosis	ICD-10-CM
D43.1	Neoplasm of uncertain behavior of brain, infratentorial	Diagnosis	ICD-10-CM
D43.2	Neoplasm of uncertain behavior of brain, unspecified	Diagnosis	ICD-10-CM
D43.3	Neoplasm of uncertain behavior of cranial nerves	Diagnosis	ICD-10-CM
D43.4	Neoplasm of uncertain behavior of spinal cord	Diagnosis	ICD-10-CM
D43.8	Neoplasm of uncertain behavior of other specified parts of central nervous system	Diagnosis	ICD-10-CM
D43.9	Neoplasm of uncertain behavior of central nervous system, unspecified	Diagnosis	ICD-10-CM

Code	Description	Code Category	Code Type
D44.0	Neoplasm of uncertain behavior of thyroid gland	Diagnosis	ICD-10-CM
D44.10	Neoplasm of uncertain behavior of unspecified adrenal gland	Diagnosis	ICD-10-CM
D44.11	Neoplasm of uncertain behavior of right adrenal gland	Diagnosis	ICD-10-CM
D44.12	Neoplasm of uncertain behavior of left adrenal gland	Diagnosis	ICD-10-CM
D44.2	Neoplasm of uncertain behavior of parathyroid gland	Diagnosis	ICD-10-CM
D44.3	Neoplasm of uncertain behavior of pituitary gland	Diagnosis	ICD-10-CM
D44.4	Neoplasm of uncertain behavior of craniopharyngeal duct	Diagnosis	ICD-10-CM
D44.5	Neoplasm of uncertain behavior of pineal gland	Diagnosis	ICD-10-CM
D44.6	Neoplasm of uncertain behavior of carotid body	Diagnosis	ICD-10-CM
D44.7	Neoplasm of uncertain behavior of aortic body and other paraganglia	Diagnosis	ICD-10-CM
D44.9	Neoplasm of uncertain behavior of unspecified endocrine gland	Diagnosis	ICD-10-CM
D47.01	Cutaneous mastocytosis	Diagnosis	ICD-10-CM
D47.02	Systemic mastocytosis	Diagnosis	ICD-10-CM
D47.09	Other mast cell neoplasms of uncertain behavior	Diagnosis	ICD-10-CM
D47.Z9	Other specified neoplasms of uncertain behavior of lymphoid, hematopoietic and related tissue	Diagnosis	ICD-10-CM
D48.0	Neoplasm of uncertain behavior of bone and articular cartilage	Diagnosis	ICD-10-CM
D48.1	Neoplasm of uncertain behavior of connective and other soft tissue	Diagnosis	ICD-10-CM
D48.2	Neoplasm of uncertain behavior of peripheral nerves and autonomic nervous systen	Diagnosis	ICD-10-CM
D48.3	Neoplasm of uncertain behavior of retroperitoneum	Diagnosis	ICD-10-CM
D48.4	Neoplasm of uncertain behavior of peritoneum	Diagnosis	ICD-10-CM
D48.60	Neoplasm of uncertain behavior of unspecified breast	Diagnosis	ICD-10-CM
D48.61	Neoplasm of uncertain behavior of right breast	Diagnosis	ICD-10-CM
D48.62	Neoplasm of uncertain behavior of left breast	Diagnosis	ICD-10-CM
D48.7	Neoplasm of uncertain behavior of other specified sites	Diagnosis	ICD-10-CM
Q85.02	Neurofibromatosis, type 2	Diagnosis	ICD-10-CM
Q85.03	Schwannomatosis	Diagnosis	ICD-10-CM
Q85.09	Other neurofibromatosis	Diagnosis	ICD-10-CM
C00	Malignant neoplasm of lip	Diagnosis	ICD-10-CM
C02	Malignant neoplasm of other and unspecified parts of tongue	Diagnosis	ICD-10-CM
C03	Malignant neoplasm of gum	Diagnosis	ICD-10-CM
C04	Malignant neoplasm of floor of mouth	Diagnosis	ICD-10-CM
C05	Malignant neoplasm of palate	Diagnosis	ICD-10-CM
C06	Malignant neoplasm of other and unspecified parts of mouth	Diagnosis	ICD-10-CM
C06.8	Malignant neoplasm of overlapping sites of other and unspecified parts of mouth	Diagnosis	ICD-10-CM
C08	Malignant neoplasm of other and unspecified major salivary glands	Diagnosis	ICD-10-CM
C09	Malignant neoplasm of tonsil	Diagnosis	ICD-10-CM
C10	Malignant neoplasm of oropharynx	Diagnosis	ICD-10-CM
C11	Malignant neoplasm of nasopharynx	Diagnosis	ICD-10-CM
C13	Malignant neoplasm of hypopharynx	Diagnosis	ICD-10-CM
C14	Malignant neoplasm of other and ill-defined sites in the lip, oral cavity and pharynx	Diagnosis	ICD-10-CM
C15	Malignant neoplasm of esophagus	Diagnosis	ICD-10-CM
C16	Malignant neoplasm of stomach	Diagnosis	ICD-10-CM
C17	Malignant neoplasm of small intestine	Diagnosis	ICD-10-CM
C18	Malignant neoplasm of colon	Diagnosis	ICD-10-CM

Code	Description	Code Category	Code Type
C21	Malignant neoplasm of anus and anal canal	Diagnosis	ICD-10-CM
C22	Malignant neoplasm of liver and intrahepatic bile ducts	Diagnosis	ICD-10-CM
C24	Malignant neoplasm of other and unspecified parts of biliary tract	Diagnosis	ICD-10-CM
C25	Malignant neoplasm of pancreas	Diagnosis	ICD-10-CM
C26	Malignant neoplasm of other and ill-defined digestive organs	Diagnosis	ICD-10-CM
C30	Malignant neoplasm of nasal cavity and middle ear	Diagnosis	ICD-10-CM
C31	Malignant neoplasm of accessory sinuses	Diagnosis	ICD-10-CM
C32	Malignant neoplasm of larynx	Diagnosis	ICD-10-CM
C34	Malignant neoplasm of bronchus and lung	Diagnosis	ICD-10-CM
C34.0	Malignant neoplasm of main bronchus	Diagnosis	ICD-10-CM
C34.1	Malignant neoplasm of upper lobe, bronchus or lung	Diagnosis	ICD-10-CM
C34.3	Malignant neoplasm of lower lobe, bronchus or lung	Diagnosis	ICD-10-CM
C34.8	Malignant neoplasm of overlapping sites of bronchus and lung	Diagnosis	ICD-10-CM
C34.9	Malignant neoplasm of unspecified part of bronchus or lung	Diagnosis	ICD-10-CM
C38	Malignant neoplasm of heart, mediastinum and pleura	Diagnosis	ICD-10-CM
C39	Malignant neoplasm of other and ill-defined sites in the respiratory system and intrathoracic organs	Diagnosis	ICD-10-CM
C40	Malignant neoplasm of bone and articular cartilage of limbs	Diagnosis	ICD-10-CM
C40.0	Malignant neoplasm of scapula and long bones of upper limb	Diagnosis	ICD-10-CM
C40.1	Malignant neoplasm of short bones of upper limb	Diagnosis	ICD-10-CM
C40.2	Malignant neoplasm of long bones of lower limb	Diagnosis	ICD-10-CM
C40.3	Malignant neoplasm of short bones of lower limb	Diagnosis	ICD-10-CM
C40.8	Malignant neoplasm of overlapping sites of bone and articular cartilage of limb	Diagnosis	ICD-10-CM
C40.9	Malignant neoplasm of unspecified bones and articular cartilage of limb	Diagnosis	ICD-10-CM
C41	Malignant neoplasm of bone and articular cartilage of other and unspecified sites	Diagnosis	ICD-10-CM
C43	Malignant melanoma of skin	Diagnosis	ICD-10-CM
C43.1	Malignant melanoma of eyelid, including canthus	Diagnosis	ICD-10-CM
C43.111	Malignant melanoma of right upper eyelid, including canthus	Diagnosis	ICD-10-CM
C43.112	Malignant melanoma of right lower eyelid, including canthus	Diagnosis	ICD-10-CM
C43.121	Malignant melanoma of left upper eyelid, including canthus	Diagnosis	ICD-10-CM
C43.122	Malignant melanoma of left lower eyelid, including canthus	Diagnosis	ICD-10-CM
C43.2	Malignant melanoma of ear and external auricular canal	Diagnosis	ICD-10-CM
C43.3	Malignant melanoma of other and unspecified parts of face	Diagnosis	ICD-10-CM
C43.5	Malignant melanoma of trunk	Diagnosis	ICD-10-CM
C43.6	Malignant melanoma of upper limb, including shoulder	Diagnosis	ICD-10-CM
C43.7	Malignant melanoma of lower limb, including hip	Diagnosis	ICD-10-CM
C44	Other and unspecified malignant neoplasm of skin	Diagnosis	ICD-10-CM
C44.0	Other and unspecified malignant neoplasm of skin of lip	Diagnosis	ICD-10-CM
C44.00	Unspecified malignant neoplasm of skin of lip	Diagnosis	ICD-10-CM
C44.09	Other specified malignant neoplasm of skin of lip	Diagnosis	ICD-10-CM
C44.1	Other and unspecified malignant neoplasm of skin of eyelid, including canthus	Diagnosis	ICD-10-CM
C44.10	Unspecified malignant neoplasm of skin of eyelid, including canthus	Diagnosis	ICD-10-CM
C44.101	Unspecified malignant neoplasm of skin of unspecified eyelid, including canthus	Diagnosis	ICD-10-CM
C44.102	Unspecified malignant neoplasm of skin of right eyelid, including canthus	Diagnosis	ICD-10-CM
C44.1021	Unspecified malignant neoplasm of skin of right upper eyelid, including canthus	Diagnosis	ICD-10-CM

Code	Description	Code Category	Code Type
C44.1022	Unspecified malignant neoplasm of skin of right lower eyelid, including canthus	Diagnosis	ICD-10-CM
C44.109	Unspecified malignant neoplasm of skin of left eyelid, including canthus	Diagnosis	ICD-10-CM
C44.1091	Unspecified malignant neoplasm of skin of left upper eyelid, including canthus	Diagnosis	ICD-10-CM
C44.1092	Unspecified malignant neoplasm of skin of left lower eyelid, including canthus	Diagnosis	ICD-10-CM
C44.13	Sebaceous cell carcinoma of skin of eyelid, including canthus	Diagnosis	ICD-10-CM
C44.131	Sebaceous cell carcinoma of skin of unspecified eyelid, including canthus	Diagnosis	ICD-10-CM
C44.132	Sebaceous cell carcinoma of skin of right eyelid, including canthus	Diagnosis	ICD-10-CM
C44.1321	Sebaceous cell carcinoma of skin of right upper eyelid, including canthus	Diagnosis	ICD-10-CM
C44.1322	Sebaceous cell carcinoma of skin of right lower eyelid, including canthus	Diagnosis	ICD-10-CM
C44.139	Sebaceous cell carcinoma of skin of left eyelid, including canthus	Diagnosis	ICD-10-CM
C44.1391	Sebaceous cell carcinoma of skin of left upper eyelid, including canthus	Diagnosis	ICD-10-CM
C44.1392	Sebaceous cell carcinoma of skin of left lower eyelid, including canthus	Diagnosis	ICD-10-CM
C44.19	Other specified malignant neoplasm of skin of eyelid, including canthus	Diagnosis	ICD-10-CM
C44.191	Other specified malignant neoplasm of skin of unspecified eyelid, including canthus	Diagnosis	ICD-10-CM
C44.192	Other specified malignant neoplasm of skin of right eyelid, including canthus	Diagnosis	ICD-10-CM
C44.1921	Other specified malignant neoplasm of skin of right upper eyelid, including canthus	Diagnosis	ICD-10-CM
C44.1922	Other specified malignant neoplasm of skin of right lower eyelid, including canthus	Diagnosis	ICD-10-CM
C44.199	Other specified malignant neoplasm of skin of left eyelid, including canthus	Diagnosis	ICD-10-CM
C44.1991	Other specified malignant neoplasm of skin of left upper eyelid, including canthus	Diagnosis	ICD-10-CM
C44.1992	Other specified malignant neoplasm of skin of left lower eyelid, including canthus	Diagnosis	ICD-10-CM
C44.2	Other and unspecified malignant neoplasm of skin of ear and external auricular cana	Diagnosis	ICD-10-CM
C44.20	Unspecified malignant neoplasm of skin of ear and external auricular canal	Diagnosis	ICD-10-CM
C44.201	Unspecified malignant neoplasm of skin of unspecified ear and external auricular canal	Diagnosis	ICD-10-CM
C44.202	Unspecified malignant neoplasm of skin of right ear and external auricular canal	Diagnosis	ICD-10-CM
C44.209	Unspecified malignant neoplasm of skin of left ear and external auricular canal	Diagnosis	ICD-10-CM
C44.29	Other specified malignant neoplasm of skin of ear and external auricular canal	Diagnosis	ICD-10-CM
C44.291	Other specified malignant neoplasm of skin of unspecified ear and external auricular canal	Diagnosis	ICD-10-CM
C44.292	Other specified malignant neoplasm of skin of right ear and external auricular canal	Diagnosis	ICD-10-CM
C44.299	Other specified malignant neoplasm of skin of left ear and external auricular canal	Diagnosis	ICD-10-CM
C44.3	Other and unspecified malignant neoplasm of skin of other and unspecified parts of face	Diagnosis	ICD-10-CM
C44.30	Unspecified malignant neoplasm of skin of other and unspecified parts of face	Diagnosis	ICD-10-CM
C44.300	Unspecified malignant neoplasm of skin of unspecified part of face	Diagnosis	ICD-10-CM
C44.301	Unspecified malignant neoplasm of skin of nose	Diagnosis	ICD-10-CM
C44.309	Unspecified malignant neoplasm of skin of other parts of face	Diagnosis	ICD-10-CM
C44.39	Other specified malignant neoplasm of skin of other and unspecified parts of face	Diagnosis	ICD-10-CM
C44.390	Other specified malignant neoplasm of skin of unspecified parts of face	Diagnosis	ICD-10-CM
C44.391	Other specified malignant neoplasm of skin of nose	Diagnosis	ICD-10-CM
C44.399	Other specified malignant neoplasm of skin of other parts of face	Diagnosis	ICD-10-CM
C44.4	Other and unspecified malignant neoplasm of skin of scalp and neck	Diagnosis	ICD-10-CM
C44.40	Unspecified malignant neoplasm of skin of scalp and neck	Diagnosis	ICD-10-CM
C44.49	Other specified malignant neoplasm of skin of scalp and neck	Diagnosis	ICD-10-CM
C44.5	Other and unspecified malignant neoplasm of skin of trunk	Diagnosis	ICD-10-CM

Code	Description	Code Category	Code Type
C44.50	Unspecified malignant neoplasm of skin of trunk	Diagnosis	ICD-10-CM
C44.500	Unspecified malignant neoplasm of anal skin	Diagnosis	ICD-10-CM
C44.501	Unspecified malignant neoplasm of skin of breast	Diagnosis	ICD-10-CM
C44.509	Unspecified malignant neoplasm of skin of other part of trunk	Diagnosis	ICD-10-CM
C44.59	Other specified malignant neoplasm of skin of trunk	Diagnosis	ICD-10-CM
C44.590	Other specified malignant neoplasm of anal skin	Diagnosis	ICD-10-CM
C44.591	Other specified malignant neoplasm of skin of breast	Diagnosis	ICD-10-CM
C44.599	Other specified malignant neoplasm of skin of other part of trunk	Diagnosis	ICD-10-CM
C44.6	Other and unspecified malignant neoplasm of skin of upper limb, including shoulder	Diagnosis	ICD-10-CM
C44.60	Unspecified malignant neoplasm of skin of upper limb, including shoulder	Diagnosis	ICD-10-CM
C44.601	Unspecified malignant neoplasm of skin of unspecified upper limb, including should	Diagnosis	ICD-10-CM
C44.602	Unspecified malignant neoplasm of skin of right upper limb, including shoulder	Diagnosis	ICD-10-CM
C44.609	Unspecified malignant neoplasm of skin of left upper limb, including shoulder	Diagnosis	ICD-10-CM
C44.69	Other specified malignant neoplasm of skin of upper limb, including shoulder	Diagnosis	ICD-10-CM
C44.691	Other specified malignant neoplasm of skin of unspecified upper limb, including shoulder	Diagnosis	ICD-10-CM
C44.692	Other specified malignant neoplasm of skin of right upper limb, including shoulder	Diagnosis	ICD-10-CM
C44.699	Other specified malignant neoplasm of skin of left upper limb, including shoulder	Diagnosis	ICD-10-CM
C44.7	Other and unspecified malignant neoplasm of skin of lower limb, including hip	Diagnosis	ICD-10-CM
C44.70	Unspecified malignant neoplasm of skin of lower limb, including hip	Diagnosis	ICD-10-CM
C44.701	Unspecified malignant neoplasm of skin of unspecified lower limb, including hip	Diagnosis	ICD-10-CM
C44.702	Unspecified malignant neoplasm of skin of right lower limb, including hip	Diagnosis	ICD-10-CM
C44.709	Unspecified malignant neoplasm of skin of left lower limb, including hip	Diagnosis	ICD-10-CM
C44.79	Other specified malignant neoplasm of skin of lower limb, including hip	Diagnosis	ICD-10-CM
C44.791	Other specified malignant neoplasm of skin of unspecified lower limb, including hip	Diagnosis	ICD-10-CM
C44.792	Other specified malignant neoplasm of skin of right lower limb, including hip	Diagnosis	ICD-10-CM
C44.799	Other specified malignant neoplasm of skin of left lower limb, including hip	Diagnosis	ICD-10-CM
C44.8	Other and unspecified malignant neoplasm of overlapping sites of skin	Diagnosis	ICD-10-CM
C44.80	Unspecified malignant neoplasm of overlapping sites of skin	Diagnosis	ICD-10-CM
C44.89	Other specified malignant neoplasm of overlapping sites of skin	Diagnosis	ICD-10-CM
C44.9	Other and unspecified malignant neoplasm of skin, unspecified	Diagnosis	ICD-10-CM
C44.90	Unspecified malignant neoplasm of skin, unspecified	Diagnosis	ICD-10-CM
C44.99	Other specified malignant neoplasm of skin, unspecified	Diagnosis	ICD-10-CM
C45	Mesothelioma	Diagnosis	ICD-10-CM
C46	Kaposi's sarcoma	Diagnosis	ICD-10-CM
C46.5	Kaposi's sarcoma of lung	Diagnosis	ICD-10-CM
C47	Malignant neoplasm of peripheral nerves and autonomic nervous system	Diagnosis	ICD-10-CM
C47.1	Malignant neoplasm of peripheral nerves of upper limb, including shoulder	Diagnosis	ICD-10-CM
C47.2	Malignant neoplasm of peripheral nerves of lower limb, including hip	Diagnosis	ICD-10-CM
C48	Malignant neoplasm of retroperitoneum and peritoneum	Diagnosis	ICD-10-CM
C49	Malignant neoplasm of other connective and soft tissue	Diagnosis	ICD-10-CM
C49.1	Malignant neoplasm of connective and soft tissue of upper limb, including shoulder	Diagnosis	ICD-10-CM
C49.2	Malignant neoplasm of connective and soft tissue of lower limb, including hip	Diagnosis	ICD-10-CM
C49.A	Gastrointestinal stromal tumor	Diagnosis	ICD-10-CM
C4A	Merkel cell carcinoma	Diagnosis	ICD-10-CM

Code	Description	Code Category	Code Type
C4A.0	Merkel cell carcinoma of lip	Diagnosis	ICD-10-CM
C4A.1	Merkel cell carcinoma of eyelid, including canthus	Diagnosis	ICD-10-CM
C4A.10	Merkel cell carcinoma of unspecified eyelid, including canthus	Diagnosis	ICD-10-CM
C4A.11	Merkel cell carcinoma of right eyelid, including canthus	Diagnosis	ICD-10-CM
C4A.111	Merkel cell carcinoma of right upper eyelid, including canthus	Diagnosis	ICD-10-CM
C4A.112	Merkel cell carcinoma of right lower eyelid, including canthus	Diagnosis	ICD-10-CM
C4A.12	Merkel cell carcinoma of left eyelid, including canthus	Diagnosis	ICD-10-CM
C4A.121	Merkel cell carcinoma of left upper eyelid, including canthus	Diagnosis	ICD-10-CM
C4A.122	Merkel cell carcinoma of left lower eyelid, including canthus	Diagnosis	ICD-10-CM
C4A.2	Merkel cell carcinoma of ear and external auricular canal	Diagnosis	ICD-10-CM
C4A.20	Merkel cell carcinoma of unspecified ear and external auricular canal	Diagnosis	ICD-10-CM
C4A.21	Merkel cell carcinoma of right ear and external auricular canal	Diagnosis	ICD-10-CM
C4A.22	Merkel cell carcinoma of left ear and external auricular canal	Diagnosis	ICD-10-CM
C4A.3	Merkel cell carcinoma of other and unspecified parts of face	Diagnosis	ICD-10-CM
C4A.30	Merkel cell carcinoma of unspecified part of face	Diagnosis	ICD-10-CM
C4A.31	Merkel cell carcinoma of nose	Diagnosis	ICD-10-CM
C4A.39	Merkel cell carcinoma of other parts of face	Diagnosis	ICD-10-CM
C4A.4	Merkel cell carcinoma of scalp and neck	Diagnosis	ICD-10-CM
C4A.5	Merkel cell carcinoma of trunk	Diagnosis	ICD-10-CM
C4A.51	Merkel cell carcinoma of anal skin	Diagnosis	ICD-10-CM
C4A.52	Merkel cell carcinoma of skin of breast	Diagnosis	ICD-10-CM
C4A.59	Merkel cell carcinoma of other part of trunk	Diagnosis	ICD-10-CM
C4A.6	Merkel cell carcinoma of upper limb, including shoulder	Diagnosis	ICD-10-CM
C4A.60	Merkel cell carcinoma of unspecified upper limb, including shoulder	Diagnosis	ICD-10-CM
C4A.61	Merkel cell carcinoma of right upper limb, including shoulder	Diagnosis	ICD-10-CM
C4A.62	Merkel cell carcinoma of left upper limb, including shoulder	Diagnosis	ICD-10-CM
C4A.7	Merkel cell carcinoma of lower limb, including hip	Diagnosis	ICD-10-CM
C4A.70	Merkel cell carcinoma of unspecified lower limb, including hip	Diagnosis	ICD-10-CM
C4A.71	Merkel cell carcinoma of right lower limb, including hip	Diagnosis	ICD-10-CM
C4A.72	Merkel cell carcinoma of left lower limb, including hip	Diagnosis	ICD-10-CM
C4A.8	Merkel cell carcinoma of overlapping sites	Diagnosis	ICD-10-CM
C4A.9	Merkel cell carcinoma, unspecified	Diagnosis	ICD-10-CM
C50	Malignant neoplasm of breast	Diagnosis	ICD-10-CM
C50.0	Malignant neoplasm of nipple and areola	Diagnosis	ICD-10-CM
C50.01	Malignant neoplasm of nipple and areola, female	Diagnosis	ICD-10-CM
C50.02	Malignant neoplasm of nipple and areola, male	Diagnosis	ICD-10-CM
C50.1	Malignant neoplasm of central portion of breast	Diagnosis	ICD-10-CM
C50.11	Malignant neoplasm of central portion of breast, female	Diagnosis	ICD-10-CM
C50.12	Malignant neoplasm of central portion of breast, male	Diagnosis	ICD-10-CM
C50.2	Malignant neoplasm of upper-inner quadrant of breast	Diagnosis	ICD-10-CM
C50.21	Malignant neoplasm of upper-inner quadrant of breast, female	Diagnosis	ICD-10-CM
C50.22	Malignant neoplasm of upper-inner quadrant of breast, male	Diagnosis	ICD-10-CM
C50.3	Malignant neoplasm of lower-inner quadrant of breast	Diagnosis	ICD-10-CM
C50.31	Malignant neoplasm of lower-inner quadrant of breast, female	Diagnosis	ICD-10-CM
C50.32	Malignant neoplasm of lower-inner quadrant of breast, male	Diagnosis	ICD-10-CM

Code	Description	Code Category	Code Type
C50.4	Malignant neoplasm of upper-outer quadrant of breast	Diagnosis	ICD-10-CM
C50.41	Malignant neoplasm of upper-outer quadrant of breast, female	Diagnosis	ICD-10-CM
C50.42	Malignant neoplasm of upper-outer quadrant of breast, male	Diagnosis	ICD-10-CM
C50.5	Malignant neoplasm of lower-outer quadrant of breast	Diagnosis	ICD-10-CM
C50.51	Malignant neoplasm of lower-outer quadrant of breast, female	Diagnosis	ICD-10-CM
C50.52	Malignant neoplasm of lower-outer quadrant of breast, male	Diagnosis	ICD-10-CM
C50.6	Malignant neoplasm of axillary tail of breast	Diagnosis	ICD-10-CM
C50.61	Malignant neoplasm of axillary tail of breast, female	Diagnosis	ICD-10-CM
C50.62	Malignant neoplasm of axillary tail of breast, male	Diagnosis	ICD-10-CM
C50.8	Malignant neoplasm of overlapping sites of breast	Diagnosis	ICD-10-CM
C50.81	Malignant neoplasm of overlapping sites of breast, female	Diagnosis	ICD-10-CM
C50.82	Malignant neoplasm of overlapping sites of breast, male	Diagnosis	ICD-10-CM
C50.9	Malignant neoplasm of breast of unspecified site	Diagnosis	ICD-10-CM
C50.91	Malignant neoplasm of breast of unspecified site, female	Diagnosis	ICD-10-CM
C50.92	Malignant neoplasm of breast of unspecified site, male	Diagnosis	ICD-10-CM
C51	Malignant neoplasm of vulva	Diagnosis	ICD-10-CM
C53	Malignant neoplasm of cervix uteri	Diagnosis	ICD-10-CM
C54	Malignant neoplasm of corpus uteri	Diagnosis	ICD-10-CM
C56	Malignant neoplasm of ovary	Diagnosis	ICD-10-CM
C57	Malignant neoplasm of other and unspecified female genital organs	Diagnosis	ICD-10-CM
C57.0	Malignant neoplasm of fallopian tube	Diagnosis	ICD-10-CM
C57.1	Malignant neoplasm of broad ligament	Diagnosis	ICD-10-CM
C57.2	Malignant neoplasm of round ligament	Diagnosis	ICD-10-CM
C60	Malignant neoplasm of penis	Diagnosis	ICD-10-CM
C62	Malignant neoplasm of testis	Diagnosis	ICD-10-CM
C62.0	Malignant neoplasm of undescended testis	Diagnosis	ICD-10-CM
C62.1	Malignant neoplasm of descended testis	Diagnosis	ICD-10-CM
C62.9	Malignant neoplasm of testis, unspecified whether descended or undescended	Diagnosis	ICD-10-CM
C63	Malignant neoplasm of other and unspecified male genital organs	Diagnosis	ICD-10-CM
C63.0	Malignant neoplasm of epididymis	Diagnosis	ICD-10-CM
C63.1	Malignant neoplasm of spermatic cord	Diagnosis	ICD-10-CM
C64	Malignant neoplasm of kidney, except renal pelvis	Diagnosis	ICD-10-CM
C65	Malignant neoplasm of renal pelvis	Diagnosis	ICD-10-CM
C66	Malignant neoplasm of ureter	Diagnosis	ICD-10-CM
C67	Malignant neoplasm of bladder	Diagnosis	ICD-10-CM
C68	Malignant neoplasm of other and unspecified urinary organs	Diagnosis	ICD-10-CM
C69	Malignant neoplasm of eye and adnexa	Diagnosis	ICD-10-CM
C69.0	Malignant neoplasm of conjunctiva	Diagnosis	ICD-10-CM
C69.1	Malignant neoplasm of cornea	Diagnosis	ICD-10-CM
C69.2	Malignant neoplasm of retina	Diagnosis	ICD-10-CM
C69.3	Malignant neoplasm of choroid	Diagnosis	ICD-10-CM
C69.4	Malignant neoplasm of ciliary body	Diagnosis	ICD-10-CM
C69.5	Malignant neoplasm of lacrimal gland and duct	Diagnosis	ICD-10-CM
C69.6	Malignant neoplasm of orbit	Diagnosis	ICD-10-CM
C69.8	Malignant neoplasm of overlapping sites of eye and adnexa	Diagnosis	ICD-10-CM

Code	Description	Code Category	Code Type
C69.9	Malignant neoplasm of unspecified site of eye	Diagnosis	ICD-10-CM
C70	Malignant neoplasm of meninges	Diagnosis	ICD-10-CM
C71	Malignant neoplasm of brain	Diagnosis	ICD-10-CM
C72	Malignant neoplasm of spinal cord, cranial nerves and other parts of central nervous system	Diagnosis	ICD-10-CM
C72.2	Malignant neoplasm of olfactory nerve	Diagnosis	ICD-10-CM
C72.3	Malignant neoplasm of optic nerve	Diagnosis	ICD-10-CM
C72.4	Malignant neoplasm of acoustic nerve	Diagnosis	ICD-10-CM
C72.5	Malignant neoplasm of other and unspecified cranial nerves	Diagnosis	ICD-10-CM
C74	Malignant neoplasm of adrenal gland	Diagnosis	ICD-10-CM
C74.0	Malignant neoplasm of cortex of adrenal gland	Diagnosis	ICD-10-CM
C74.1	Malignant neoplasm of medulla of adrenal gland	Diagnosis	ICD-10-CM
C74.9	Malignant neoplasm of unspecified part of adrenal gland	Diagnosis	ICD-10-CM
C75	Malignant neoplasm of other endocrine glands and related structures	Diagnosis	ICD-10-CM
C76	Malignant neoplasm of other and ill-defined sites	Diagnosis	ICD-10-CM
C76.4	Malignant neoplasm of upper limb	Diagnosis	ICD-10-CM
C76.5	Malignant neoplasm of lower limb	Diagnosis	ICD-10-CM
C77	Secondary and unspecified malignant neoplasm of lymph nodes	Diagnosis	ICD-10-CM
C78	Secondary malignant neoplasm of respiratory and digestive organs	Diagnosis	ICD-10-CM
C78.0	Secondary malignant neoplasm of lung	Diagnosis	ICD-10-CM
C78.3	Secondary malignant neoplasm of other and unspecified respiratory organs	Diagnosis	ICD-10-CM
C78.8	Secondary malignant neoplasm of other and unspecified digestive organs	Diagnosis	ICD-10-CM
C79	Secondary malignant neoplasm of other and unspecified sites	Diagnosis	ICD-10-CM
C79.0	Secondary malignant neoplasm of kidney and renal pelvis	Diagnosis	ICD-10-CM
C79.1	Secondary malignant neoplasm of bladder and other and unspecified urinary organs	Diagnosis	ICD-10-CM
C79.3	Secondary malignant neoplasm of brain and cerebral meninges	Diagnosis	ICD-10-CM
C79.4	Secondary malignant neoplasm of other and unspecified parts of nervous system	Diagnosis	ICD-10-CM
C79.5	Secondary malignant neoplasm of bone and bone marrow	Diagnosis	ICD-10-CM
C79.6	Secondary malignant neoplasm of ovary	Diagnosis	ICD-10-CM
C79.7	Secondary malignant neoplasm of adrenal gland	Diagnosis	ICD-10-CM
C79.8	Secondary malignant neoplasm of other specified sites	Diagnosis	ICD-10-CM
C7A	Malignant neuroendocrine tumors	Diagnosis	ICD-10-CM
C7A.0	Malignant carcinoid tumors	Diagnosis	ICD-10-CM
C7A.00	Malignant carcinoid tumor of unspecified site	Diagnosis	ICD-10-CM
C7A.01	Malignant carcinoid tumors of the small intestine	Diagnosis	ICD-10-CM
C7A.010	Malignant carcinoid tumor of the duodenum	Diagnosis	ICD-10-CM
C7A.011	Malignant carcinoid tumor of the jejunum	Diagnosis	ICD-10-CM
C7A.012	Malignant carcinoid tumor of the ileum	Diagnosis	ICD-10-CM
C7A.019	Malignant carcinoid tumor of the small intestine, unspecified portion	Diagnosis	ICD-10-CM
C7A.02	Malignant carcinoid tumors of the appendix, large intestine, and rectum	Diagnosis	ICD-10-CM
C7A.020	Malignant carcinoid tumor of the appendix	Diagnosis	ICD-10-CM
C7A.021	Malignant carcinoid tumor of the cecum	Diagnosis	ICD-10-CM
C7A.022	Malignant carcinoid tumor of the ascending colon	Diagnosis	ICD-10-CM
C7A.023	Malignant carcinoid tumor of the transverse colon	Diagnosis	ICD-10-CM

Code	Description	Code Category	Code Type
C7A.024	Malignant carcinoid tumor of the descending colon	Diagnosis	ICD-10-CM
C7A.025	Malignant carcinoid tumor of the sigmoid colon	Diagnosis	ICD-10-CM
C7A.026	Malignant carcinoid tumor of the rectum	Diagnosis	ICD-10-CM
C7A.029	Malignant carcinoid tumor of the large intestine, unspecified portion	Diagnosis	ICD-10-CM
C7A.09	Malignant carcinoid tumors of other sites	Diagnosis	ICD-10-CM
C7A.090	Malignant carcinoid tumor of the bronchus and lung	Diagnosis	ICD-10-CM
C7A.091	Malignant carcinoid tumor of the thymus	Diagnosis	ICD-10-CM
C7A.092	Malignant carcinoid tumor of the stomach	Diagnosis	ICD-10-CM
C7A.093	Malignant carcinoid tumor of the kidney	Diagnosis	ICD-10-CM
C7A.094	Malignant carcinoid tumor of the foregut, unspecified	Diagnosis	ICD-10-CM
C7A.095	Malignant carcinoid tumor of the midgut, unspecified	Diagnosis	ICD-10-CM
C7A.096	Malignant carcinoid tumor of the hindgut, unspecified	Diagnosis	ICD-10-CM
C7A.098	Malignant carcinoid tumors of other sites	Diagnosis	ICD-10-CM
C7A.1	Malignant poorly differentiated neuroendocrine tumors	Diagnosis	ICD-10-CM
C7A.8	Other malignant neuroendocrine tumors	Diagnosis	ICD-10-CM
C7B	Secondary neuroendocrine tumors	Diagnosis	ICD-10-CM
C7B.0	Secondary carcinoid tumors	Diagnosis	ICD-10-CM
C7B.00	Secondary carcinoid tumors, unspecified site	Diagnosis	ICD-10-CM
C7B.01	Secondary carcinoid tumors of distant lymph nodes	Diagnosis	ICD-10-CM
C7B.02	Secondary carcinoid tumors of liver	Diagnosis	ICD-10-CM
C7B.03	Secondary carcinoid tumors of bone	Diagnosis	ICD-10-CM
C7B.04	Secondary carcinoid tumors of peritoneum	Diagnosis	ICD-10-CM
C7B.09	Secondary carcinoid tumors of other sites	Diagnosis	ICD-10-CM
C7B.1	Secondary Merkel cell carcinoma	Diagnosis	ICD-10-CM
C7B.8	Other secondary neuroendocrine tumors	Diagnosis	ICD-10-CM
C80	Malignant neoplasm without specification of site	Diagnosis	ICD-10-CM
C81	Hodgkin lymphoma	Diagnosis	ICD-10-CM
C81.0	Nodular lymphocyte predominant Hodgkin lymphoma	Diagnosis	ICD-10-CM
C81.1	Nodular sclerosis Hodgkin lymphoma	Diagnosis	ICD-10-CM
C81.2	Mixed cellularity Hodgkin lymphoma	Diagnosis	ICD-10-CM
C81.3	Lymphocyte depleted Hodgkin lymphoma	Diagnosis	ICD-10-CM
C81.4	Lymphocyte-rich Hodgkin lymphoma	Diagnosis	ICD-10-CM
C81.7	Other Hodgkin lymphoma	Diagnosis	ICD-10-CM
C81.9	Hodgkin lymphoma, unspecified	Diagnosis	ICD-10-CM
C82	Follicular lymphoma	Diagnosis	ICD-10-CM
C82.0	Follicular lymphoma grade I	Diagnosis	ICD-10-CM
C82.1	Follicular lymphoma grade II	Diagnosis	ICD-10-CM
C82.2	Follicular lymphoma grade III, unspecified	Diagnosis	ICD-10-CM
C82.3	Follicular lymphoma grade IIIa	Diagnosis	ICD-10-CM
C82.4	Follicular lymphoma grade IIIb	Diagnosis	ICD-10-CM
C82.5	Diffuse follicle center lymphoma	Diagnosis	ICD-10-CM
C82.6	Cutaneous follicle center lymphoma	Diagnosis	ICD-10-CM
C82.8	Other types of follicular lymphoma	Diagnosis	ICD-10-CM
C82.9	Follicular lymphoma, unspecified	Diagnosis	ICD-10-CM
C83	Non-follicular lymphoma	Diagnosis	ICD-10-CM

Code	Description	Code Category	Code Type
C83.0	Small cell B-cell lymphoma	Diagnosis	ICD-10-CM
C83.1	Mantle cell lymphoma	Diagnosis	ICD-10-CM
C83.3	Diffuse large B-cell lymphoma	Diagnosis	ICD-10-CM
C83.5	Lymphoblastic (diffuse) lymphoma	Diagnosis	ICD-10-CM
C83.7	Burkitt lymphoma	Diagnosis	ICD-10-CM
C83.8	Other non-follicular lymphoma	Diagnosis	ICD-10-CM
C83.9	Non-follicular (diffuse) lymphoma, unspecified	Diagnosis	ICD-10-CM
C84	Mature T/NK-cell lymphomas	Diagnosis	ICD-10-CM
C84.0	Mycosis fungoides	Diagnosis	ICD-10-CM
C84.1	Sezary disease	Diagnosis	ICD-10-CM
C84.4	Peripheral T-cell lymphoma, not classified	Diagnosis	ICD-10-CM
C84.6	Anaplastic large cell lymphoma, ALK-positive	Diagnosis	ICD-10-CM
C84.7	Anaplastic large cell lymphoma, ALK-negative	Diagnosis	ICD-10-CM
C84.9	Mature T/NK-cell lymphomas, unspecified	Diagnosis	ICD-10-CM
C84.A	Cutaneous T-cell lymphoma, unspecified	Diagnosis	ICD-10-CM
C84.Z	Other mature T/NK-cell lymphomas	Diagnosis	ICD-10-CM
C85	Other specified and unspecified types of non-Hodgkin lymphoma	Diagnosis	ICD-10-CM
C85.1	Unspecified B-cell lymphoma	Diagnosis	ICD-10-CM
C85.2	Mediastinal (thymic) large B-cell lymphoma	Diagnosis	ICD-10-CM
C85.8	Other specified types of non-Hodgkin lymphoma	Diagnosis	ICD-10-CM
C85.9	Non-Hodgkin lymphoma, unspecified	Diagnosis	ICD-10-CM
C86	Other specified types of T/NK-cell lymphoma	Diagnosis	ICD-10-CM
C88	Malignant immunoproliferative diseases and certain other B-cell lymphomas	Diagnosis	ICD-10-CM
C88.0	Waldenstrom macroglobulinemia	Diagnosis	ICD-10-CM
C90	Multiple myeloma and malignant plasma cell neoplasms	Diagnosis	ICD-10-CM
C90.0	Multiple myeloma	Diagnosis	ICD-10-CM
C90.1	Plasma cell leukemia	Diagnosis	ICD-10-CM
C90.2	Extramedullary plasmacytoma	Diagnosis	ICD-10-CM
C90.3	Solitary plasmacytoma	Diagnosis	ICD-10-CM
C91	Lymphoid leukemia	Diagnosis	ICD-10-CM
C91.0	Acute lymphoblastic leukemia [ALL]	Diagnosis	ICD-10-CM
C91.1	Chronic lymphocytic leukemia of B-cell type	Diagnosis	ICD-10-CM
C91.3	Prolymphocytic leukemia of B-cell type	Diagnosis	ICD-10-CM
C91.4	Hairy cell leukemia	Diagnosis	ICD-10-CM
C91.5	Adult T-cell lymphoma/leukemia (HTLV-1-associated)	Diagnosis	ICD-10-CM
C91.6	Prolymphocytic leukemia of T-cell type	Diagnosis	ICD-10-CM
C91.9	Lymphoid leukemia, unspecified	Diagnosis	ICD-10-CM
C91.A	Mature B-cell leukemia Burkitt-type	Diagnosis	ICD-10-CM
C91.Z	Other lymphoid leukemia	Diagnosis	ICD-10-CM
C92	Myeloid leukemia	Diagnosis	ICD-10-CM
C92.0	Acute myeloblastic leukemia	Diagnosis	ICD-10-CM
C92.1	Chronic myeloid leukemia, BCR/ABL-positive	Diagnosis	ICD-10-CM
C92.2	Atypical chronic myeloid leukemia, BCR/ABL-negative	Diagnosis	ICD-10-CM
C92.3	Myeloid sarcoma	Diagnosis	ICD-10-CM
C92.4	Acute promyelocytic leukemia	Diagnosis	ICD-10-CM

Code	Description	Code Category	Code Type
C92.5	Acute myelomonocytic leukemia	Diagnosis	ICD-10-CM
C92.6	Acute myeloid leukemia with 11q23-abnormality	Diagnosis	ICD-10-CM
C92.9	Myeloid leukemia, unspecified	Diagnosis	ICD-10-CM
C92.A	Acute myeloid leukemia with multilineage dysplasia	Diagnosis	ICD-10-CM
C92.Z	Other myeloid leukemia	Diagnosis	ICD-10-CM
C93	Monocytic leukemia	Diagnosis	ICD-10-CM
C93.0	Acute monoblastic/monocytic leukemia	Diagnosis	ICD-10-CM
C93.1	Chronic myelomonocytic leukemia	Diagnosis	ICD-10-CM
C93.3	Juvenile myelomonocytic leukemia	Diagnosis	ICD-10-CM
C93.9	Monocytic leukemia, unspecified	Diagnosis	ICD-10-CM
C93.Z	Other monocytic leukemia	Diagnosis	ICD-10-CM
C94	Other leukemias of specified cell type	Diagnosis	ICD-10-CM
C94.0	Acute erythroid leukemia	Diagnosis	ICD-10-CM
C94.2	Acute megakaryoblastic leukemia	Diagnosis	ICD-10-CM
C94.3	Mast cell leukemia	Diagnosis	ICD-10-CM
C94.4	Acute panmyelosis with myelofibrosis	Diagnosis	ICD-10-CM
C94.40	Acute panmyelosis with myelofibrosis not having achieved remission	Diagnosis	ICD-10-CM
C94.41	Acute panmyelosis with myelofibrosis, in remission	Diagnosis	ICD-10-CM
C94.42	Acute panmyelosis with myelofibrosis, in relapse	Diagnosis	ICD-10-CM
C94.6	Myelodysplastic disease, not classified	Diagnosis	ICD-10-CM
C94.8	Other specified leukemias	Diagnosis	ICD-10-CM
C95	Leukemia of unspecified cell type	Diagnosis	ICD-10-CM
C95.0	Acute leukemia of unspecified cell type	Diagnosis	ICD-10-CM
C95.1	Chronic leukemia of unspecified cell type	Diagnosis	ICD-10-CM
C95.9	Leukemia, unspecified	Diagnosis	ICD-10-CM
C96	Other and unspecified malignant neoplasms of lymphoid, hematopoietic and related tissue	Diagnosis	ICD-10-CM
C96.2	Malignant mast cell neoplasm	Diagnosis	ICD-10-CM
C96.5	Multifocal and unisystemic Langerhans-cell histiocytosis	Diagnosis	ICD-10-CM
C96.6	Unifocal Langerhans-cell histiocytosis	Diagnosis	ICD-10-CM
3E0600P	Introduction of Clofarabine into Central Artery, Open Approach	Procedure	ICD-10-PCS
3E0630P	Introduction of Clofarabine into Central Artery, Percutaneous Approach	Procedure	ICD-10-PCS
3E0400P	Introduction of Clofarabine into Central Vein, Open Approach	Procedure	ICD-10-PCS
3E0430P	Introduction of Clofarabine into Central Vein, Percutaneous Approach	Procedure	ICD-10-PCS
3E0500P	Introduction of Clofarabine into Peripheral Artery, Open Approach	Procedure	ICD-10-PCS
3E0530P	Introduction of Clofarabine into Peripheral Artery, Percutaneous Approach	Procedure	ICD-10-PCS
3E0300P	Introduction of Clofarabine into Peripheral Vein, Open Approach	Procedure	ICD-10-PCS
3E0330P	Introduction of Clofarabine into Peripheral Vein, Percutaneous Approach	Procedure	ICD-10-PCS
96415	Chemotherapy administration, intravenous infusion technique; each additional hour (List separately in addition to code for primary procedure)	Procedure	CPT-4
96417	Chemotherapy administration, intravenous infusion technique; each additional sequential infusion (different substance/drug), up to 1 hour (List separately in addition to code for primary procedure)	Procedure	CPT-4

Code	Description	Code Category	Code Type
96416	Chemotherapy administration, intravenous infusion technique; initiation of prolonged chemotherapy infusion (more than 8 hours), requiring use of a portable or implantable pump	Procedure	CPT-4
96413	Chemotherapy administration, intravenous infusion technique; up to 1 hour, single or initial substance/drug	Procedure	CPT-4
96414	Chemotherapy administration, intravenous; infusion technique, initiation of prolonged infusion (more than 8 hours), requiring the use of a portable or implantable pump	Procedure	CPT-4
96412	Chemotherapy administration, intravenous; infusion technique, one to 8 hours, each additional hour (List separately in addition to code for primary procedure)	Procedure	CPT-4
96410	Chemotherapy administration, intravenous; infusion technique, up to one hour	Procedure	CPT-4
96408	Chemotherapy administration, intravenous; push technique	Procedure	CPT-4
96411	Chemotherapy administration; intravenous, push technique, each additional substance/drug (List separately in addition to code for primary procedure)	Procedure	CPT-4
96409	Chemotherapy administration; intravenous, push technique, single or initial substance/drug	Procedure	CPT-4
96508	Chemotherapy Injection, Intravenous, Complex, Using One Or	Procedure	CPT-4
96509	Chemotherapy Injection, Intravenous, Complex, Using One Or	Procedure	CPT-4
96510	Chemotherapy Injection, Intravenous, Complex, Using One Or	Procedure	CPT-4
96511	Chemotherapy Injection, Intravenous, Complex, Using One Or	Procedure	CPT-4
96512	Chemotherapy Injection, Intravenous, Complex, Using One Or	Procedure	CPT-4
96504	Chemotherapy Injection, Intravenous, Multiple Premixed Agents,	Procedure	CPT-4
96505	Chemotherapy Injection, Intravenous, Multiple Premixed Agents,	Procedure	CPT-4
96500	Chemotherapy Injection, Intravenous, Single Premixed Agent,	Procedure	CPT-4
96501	Chemotherapy Injection, Intravenous, Single Premixed Agent,	Procedure	CPT-4
99555	Home infusion for chemotherapy, per visit	Procedure	CPT-4
79100	Radiopharmaceutical therapy, polycythemia vera, chronic leukemia, each treatment by intravenous injection	Procedure	CPT-4
G0498	Chemotherapy administration, intravenous infusion technique; initiation of infusion in the office/clinic setting using office/clinic pump/supplies, with continuation of the infusion in the community setting (e.g., home, domiciliary, rest home or assisted living) using a portable pump provided by the office/clinic, includes follow up office/clinic visit at the conclusion of the infusion	Procedure	HCPCS
G0359	Chemotherapy administration, intravenous infusion technique; up to one hour, single or initial substance/drug	Procedure	HCPCS
C8955	Chemotherapy administration, intravenous; infusion technique, each additional hour (List separately in addition to C8954)	Procedure	HCPCS
C8954	Chemotherapy administration, intravenous; infusion technique, up to one hour	Procedure	HCPCS
C8953	Chemotherapy administration, intravenous; push technique	Procedure	HCPCS
S9329	Home infusion therapy, chemotherapy infusion; administrative services, professional pharmacy services, care coordination, and all necessary supplies and equipment (drugs and nursing visits coded separately), per diem (do not use this code with S9330 or S9331)	Procedure	HCPCS

Code	Description	Code Category	Code Type
S9330	Home infusion therapy, continuous (24 hours or more) chemotherapy infusion; administrative services, professional pharmacy services, care coordination, and all necessary supplies and equipment (drugs and nursing visits coded separately), per diem	Procedure	HCPCS
S9331	Home infusion therapy, intermittent (less than 24 hours) chemotherapy infusion; administrative services, professional pharmacy services, care coordination, and all necessary supplies and equipment (drugs and nursing visits coded separately), per diem	Procedure	HCPCS
	**Viral Pneumonia**		
480	Viral pneumonia	Diagnosis	ICD-9-CM
480.0	Pneumonia due to adenovirus	Diagnosis	ICD-9-CM
480.1	Pneumonia due to respiratory syncytial virus	Diagnosis	ICD-9-CM
480.2	Pneumonia due to parainfluenza virus	Diagnosis	ICD-9-CM
480.3	Pneumonia due to SARS-associated coronavirus	Diagnosis	ICD-9-CM
480.8	Pneumonia due to other virus not elsewhere classified	Diagnosis	ICD-9-CM
480.9	Unspecified viral pneumonia	Diagnosis	ICD-9-CM
481	Pneumococcal pneumonia (streptococcus pneumoniae pneumonia)	Diagnosis	ICD-9-CM
482	Other bacterial pneumonia	Diagnosis	ICD-9-CM
482.0	Pneumonia due to Klebsiella pneumoniae	Diagnosis	ICD-9-CM
482.1	Pneumonia due to Pseudomonas	Diagnosis	ICD-9-CM
482.2	Pneumonia due to Hemophilus influenzae (H. influenzae)	Diagnosis	ICD-9-CM
482.3	Pneumonia due to Streptococcus	Diagnosis	ICD-9-CM
482.30	Pneumonia due to unspecified Streptococcus	Diagnosis	ICD-9-CM
482.31	Pneumonia due to Streptococcus, group A	Diagnosis	ICD-9-CM
482.32	Pneumonia due to Streptococcus, group B	Diagnosis	ICD-9-CM
482.39	Pneumonia due to other Streptococcus	Diagnosis	ICD-9-CM
482.4	Pneumonia due to Staphylococcus	Diagnosis	ICD-9-CM
482.40	Pneumonia due to Staphylococcus, unspecified	Diagnosis	ICD-9-CM
482.41	Methicillin susceptible pneumonia due to Staphylococcus aureus	Diagnosis	ICD-9-CM
482.42	Methicillin resistant pneumonia due to Staphylococcus aureus	Diagnosis	ICD-9-CM
482.49	Other Staphylococcus pneumonia	Diagnosis	ICD-9-CM
482.8	Pneumonia due to other specified bacteria	Diagnosis	ICD-9-CM
482.81	Pneumonia due to anaerobes	Diagnosis	ICD-9-CM
482.82	Pneumonia due to Escherichia coli (E. coli)	Diagnosis	ICD-9-CM
482.83	Pneumonia due to other gram-negative bacteria	Diagnosis	ICD-9-CM
482.84	Legionnaires' disease	Diagnosis	ICD-9-CM
482.89	Pneumonia due to other specified bacteria	Diagnosis	ICD-9-CM
482.9	Unspecified bacterial pneumonia	Diagnosis	ICD-9-CM
483	Pneumonia due to other specified organism	Diagnosis	ICD-9-CM
483.0	Pneumonia due to Mycoplasma pneumoniae	Diagnosis	ICD-9-CM
483.1	Pneumonia due to Chlamydia	Diagnosis	ICD-9-CM
483.8	Pneumonia due to other specified organism	Diagnosis	ICD-9-CM
484	Pneumonia in infectious diseases classified elsewhere	Diagnosis	ICD-9-CM
484.1	Pneumonia in cytomegalic inclusion disease	Diagnosis	ICD-9-CM
484.3	Pneumonia in whooping cough	Diagnosis	ICD-9-CM

Code	Description	Code Category	Code Type
484.5	Pneumonia in anthrax	Diagnosis	ICD-9-CM
484.6	Pneumonia in aspergillosis	Diagnosis	ICD-9-CM
484.7	Pneumonia in other systemic mycoses	Diagnosis	ICD-9-CM
484.8	Pneumonia in other infectious diseases classified elsewhere	Diagnosis	ICD-9-CM
485	Bronchopneumonia, organism unspecified	Diagnosis	ICD-9-CM
486	Pneumonia, organism unspecified	Diagnosis	ICD-9-CM
507	Pneumonitis due to solids and liquids	Diagnosis	ICD-9-CM
507.0	Pneumonitis due to inhalation of food or vomitus	Diagnosis	ICD-9-CM
507.1	Pneumonitis due to inhalation of oils and essences	Diagnosis	ICD-9-CM
507.8	Pneumonitis due to other solids and liquids	Diagnosis	ICD-9-CM
997.3	Respiratory complications	Diagnosis	ICD-9-CM
997.31	Ventilator associated pneumonia	Diagnosis	ICD-9-CM
997.32	Postprocedural aspiration pneumonia	Diagnosis	ICD-9-CM
997.39	Other respiratory complications	Diagnosis	ICD-9-CM
A22.1	Pulmonary anthrax	Diagnosis	ICD-10-CM
A37.01	Whooping cough due to Bordetella pertussis with pneumonia	Diagnosis	ICD-10-CM
A37.11	Whooping cough due to Bordetella parapertussis with pneumonia	Diagnosis	ICD-10-CM
A37.81	Whooping cough due to other Bordetella species with pneumonia	Diagnosis	ICD-10-CM
A37.91	Whooping cough, unspecified species with pneumonia	Diagnosis	ICD-10-CM
A48.1	Legionnaires' disease	Diagnosis	ICD-10-CM
B25.0	Cytomegaloviral pneumonitis	Diagnosis	ICD-10-CM
B44.0	Invasive pulmonary aspergillosis	Diagnosis	ICD-10-CM
B77.81	Ascariasis pneumonia	Diagnosis	ICD-10-CM
J12.0	Adenoviral pneumonia	Diagnosis	ICD-10-CM
J12.1	Respiratory syncytial virus pneumonia	Diagnosis	ICD-10-CM
J12.2	Parainfluenza virus pneumonia	Diagnosis	ICD-10-CM
J12.3	Human metapneumovirus pneumonia	Diagnosis	ICD-10-CM
J12.81	Pneumonia due to SARS-associated coronavirus	Diagnosis	ICD-10-CM
J12.89	Other viral pneumonia	Diagnosis	ICD-10-CM
J12.9	Viral pneumonia, unspecified	Diagnosis	ICD-10-CM
J13	Pneumonia due to Streptococcus pneumoniae	Diagnosis	ICD-10-CM
J14	Pneumonia due to Hemophilus influenzae	Diagnosis	ICD-10-CM
J15.0	Pneumonia due to Klebsiella pneumoniae	Diagnosis	ICD-10-CM
J15.1	Pneumonia due to Pseudomonas	Diagnosis	ICD-10-CM
J15.20	Pneumonia due to staphylococcus, unspecified	Diagnosis	ICD-10-CM
J15.211	Pneumonia due to Methicillin susceptible Staphylococcus aureus	Diagnosis	ICD-10-CM
J15.212	Pneumonia due to Methicillin resistant Staphylococcus aureus	Diagnosis	ICD-10-CM
J15.29	Pneumonia due to other staphylococcus	Diagnosis	ICD-10-CM
J15.3	Pneumonia due to streptococcus, group B	Diagnosis	ICD-10-CM
J15.4	Pneumonia due to other streptococci	Diagnosis	ICD-10-CM
J15.5	Pneumonia due to Escherichia coli	Diagnosis	ICD-10-CM
J15.6	Pneumonia due to other Gram-negative bacteria	Diagnosis	ICD-10-CM
J15.7	Pneumonia due to Mycoplasma pneumoniae	Diagnosis	ICD-10-CM
J15.8	Pneumonia due to other specified bacteria	Diagnosis	ICD-10-CM
J15.9	Unspecified bacterial pneumonia	Diagnosis	ICD-10-CM

Code	Description	Code Category	Code Type
J16.0	Chlamydial pneumonia	Diagnosis	ICD-10-CM
J16.8	Pneumonia due to other specified infectious organisms	Diagnosis	ICD-10-CM
J17	Pneumonia in diseases classified elsewhere	Diagnosis	ICD-10-CM
J18.0	Bronchopneumonia, unspecified organism	Diagnosis	ICD-10-CM
J18.1	Lobar pneumonia, unspecified organism	Diagnosis	ICD-10-CM
J18.8	Other pneumonia, unspecified organism	Diagnosis	ICD-10-CM
J18.9	Pneumonia, unspecified organism	Diagnosis	ICD-10-CM
J69.0	Pneumonitis due to inhalation of food and vomit	Diagnosis	ICD-10-CM
J69.1	Pneumonitis due to inhalation of oils and essences	Diagnosis	ICD-10-CM
J69.8	Pneumonitis due to inhalation of other solids and liquids	Diagnosis	ICD-10-CM
J95.4	Chemical pneumonitis due to anesthesia	Diagnosis	ICD-10-CM
J95.5	Postprocedural subglottic stenosis	Diagnosis	ICD-10-CM
J95.851	Ventilator associated pneumonia	Diagnosis	ICD-10-CM
J95.859	Other complication of respirator [ventilator]	Diagnosis	ICD-10-CM
J95.88	Other intraoperative complications of respiratory system, not elsewhere classified	Diagnosis	ICD-10-CM
J95.89	Other postprocedural complications and disorders of respiratory system, not elsewhere classified	Diagnosis	ICD-10-CM
487.0	Influenza with pneumonia	Diagnosis	ICD-10-CM
488.01	Influenza due to identified avian influenza virus with pneumonia	Diagnosis	ICD-10-CM
488.11	Influenza due to identified 2009 H1N1 influenza virus with pneumonia	Diagnosis	ICD-10-CM
488.81	Influenza due to identified novel influenza A virus with pneumonia	Diagnosis	ICD-10-CM
J09.X1	Influenza due to identified novel influenza A virus with pneumonia	Diagnosis	ICD-10-CM
J10.00	Influenza due to other identified influenza virus with unspecified type of pneumonia	Diagnosis	ICD-10-CM
J10.01	Influenza due to other identified influenza virus with the same other identified influenza virus pneumonia	Diagnosis	ICD-10-CM
J10.08	Influenza due to other identified influenza virus with other specified pneumonia	Diagnosis	ICD-10-CM
J11.00	Influenza due to unidentified influenza virus with unspecified type of pneumonia	Diagnosis	ICD-10-CM
J11.08	Influenza due to unidentified influenza virus with specified pneumonia	Diagnosis	ICD-10-CM
J12.82	Pneumonia due to coronavirus disease 2019	Diagnosis	ICD-10-CM
**Acute Respiratory Failure**			
518.81	Acute respiratory failure	Diagnosis	ICD-9-CM
J96.00	Acute respiratory failure, unspecified whether with hypoxia or hypercapnia	Diagnosis	ICD-10-CM
J96.01	Acute respiratory failure with hypoxia	Diagnosis	ICD-10-CM
J96.02	Acute respiratory failure with hypercapnia	Diagnosis	ICD-10-CM
J96.90	Respiratory failure, unspecified, unspecified whether with hypoxia or hypercapnia	Diagnosis	ICD-10-CM
J96.91	Respiratory failure, unspecified with hypoxia	Diagnosis	ICD-10-CM
J96.92	Respiratory failure, unspecified with hypercapnia	Diagnosis	ICD-10-CM
**Acute Respiratory Distress Syndrome**			
518.82	Other pulmonary insufficiency, not elsewhere classified	Diagnosis	ICD-9-CM
J80	Acute respiratory distress syndrome	Diagnosis	ICD-10-CM
**Sepsis**			
038.0	Streptococcal septicemia	Diagnosis	ICD-9-CM
038.1	Staphylococcal septicemia	Diagnosis	ICD-9-CM
038.10	Unspecified staphylococcal septicemia	Diagnosis	ICD-9-CM

Code	Description	Code Category	Code Type
038.11	Methicillin susceptible Staphylococcus aureus septicemia	Diagnosis	ICD-9-CM
038.12	Methicillin resistant Staphylococcus aureus septicemia	Diagnosis	ICD-9-CM
038.19	Other staphylococcal septicemia	Diagnosis	ICD-9-CM
038.2	Pneumococcal septicemia	Diagnosis	ICD-9-CM
038.3	Septicemia due to anaerobes	Diagnosis	ICD-9-CM
038.4	Septicemia due to other gram-negative organisms	Diagnosis	ICD-9-CM
038.40	Septicemia due to unspecified gram-negative organism	Diagnosis	ICD-9-CM
038.41	Septicemia due to hemophilus influenzae (H. influenzae)	Diagnosis	ICD-9-CM
038.42	Septicemia due to Escherichia coli (E. coli)	Diagnosis	ICD-9-CM
038.43	Septicemia due to pseudomonas	Diagnosis	ICD-9-CM
038.44	Septicemia due to serratia	Diagnosis	ICD-9-CM
038.49	Other septicemia due to gram-negative organism	Diagnosis	ICD-9-CM
038.8	Other specified septicemia	Diagnosis	ICD-9-CM
038.9	Unspecified septicemia	Diagnosis	ICD-9-CM
785.52	Septic shock	Diagnosis	ICD-9-CM
995.90	Systemic inflammatory response syndrome, unspecified	Diagnosis	ICD-9-CM
995.91	Sepsis	Diagnosis	ICD-9-CM
995.92	Severe sepsis	Diagnosis	ICD-9-CM
A02.1	Salmonella sepsis	Diagnosis	ICD-10-CM
A22.7	Anthrax sepsis	Diagnosis	ICD-10-CM
A26.7	Erysipelothrix sepsis	Diagnosis	ICD-10-CM
A32.7	Listerial sepsis	Diagnosis	ICD-10-CM
A40.0	Sepsis due to streptococcus, group A	Diagnosis	ICD-10-CM
A40.1	Sepsis due to streptococcus, group B	Diagnosis	ICD-10-CM
A40.3	Sepsis due to Streptococcus pneumoniae	Diagnosis	ICD-10-CM
A40.8	Other streptococcal sepsis	Diagnosis	ICD-10-CM
A40.9	Streptococcal sepsis, unspecified	Diagnosis	ICD-10-CM
A41.0	Sepsis due to Staphylococcus aureus	Diagnosis	ICD-10-CM
A41.01	Sepsis due to Methicillin susceptible Staphylococcus aureus	Diagnosis	ICD-10-CM
A41.02	Sepsis due to Methicillin resistant Staphylococcus aureus	Diagnosis	ICD-10-CM
A41.0Z16	Sepsis due to Staphylococcus aureusInfection with drug resistant microorganisms	Diagnosis	ICD-10-CM
A41.1	Sepsis due to other specified staphylococcus	Diagnosis	ICD-10-CM
A41.2	Sepsis due to unspecified staphylococcus	Diagnosis	ICD-10-CM
A41.3	Sepsis due to Hemophilus influenzae	Diagnosis	ICD-10-CM
A41.4	Sepsis due to anaerobes	Diagnosis	ICD-10-CM
A41.50	Gram-negative sepsis, unspecified	Diagnosis	ICD-10-CM
A41.51	Sepsis due to Escherichia coli [E. coli]	Diagnosis	ICD-10-CM
A41.52	Sepsis due to Pseudomonas	Diagnosis	ICD-10-CM
A41.53	Sepsis due to Serratia	Diagnosis	ICD-10-CM
A41.59	Other Gram-negative sepsis	Diagnosis	ICD-10-CM
A41.81	Sepsis due to Enterococcus	Diagnosis	ICD-10-CM
A41.89	Other specified sepsis	Diagnosis	ICD-10-CM
A41.9	Sepsis, unspecified organism	Diagnosis	ICD-10-CM
A42.7	Actinomycotic sepsis	Diagnosis	ICD-10-CM
A54.86	Gonococcal sepsis	Diagnosis	ICD-10-CM

Code	Description	Code Category	Code Type
B37.7	Candidal sepsis	Diagnosis	ICD-10-CM
R65.20	Severe sepsis without septic shock	Diagnosis	ICD-10-CM
R65.21	Severe sepsis with septic shock	Diagnosis	ICD-10-CM
003.1	Salmonella septicemia	Diagnosis	ICD-10-CM
022.3	Anthrax septicemia	Diagnosis	ICD-10-CM
670.20	Puerperal sepsis, unspecified as to episode of care or not applicable	Diagnosis	ICD-10-CM
670.22	Puerperal sepsis, delivered, with mention of postpartum complication	Diagnosis	ICD-10-CM
670.24	Puerperal sepsis, postpartum condition or complication	Diagnosis	ICD-10-CM
771.81	Septicemia (sepsis) of newborn	Diagnosis	ICD-10-CM
A40	Streptococcal sepsis	Diagnosis	ICD-10-CM
A41	Other sepsis	Diagnosis	ICD-10-CM
A41.5	Sepsis due to other Gram-negative organisms	Diagnosis	ICD-10-CM
A41.8	Other specified sepsis	Diagnosis	ICD-10-CM
O03.37	Sepsis following incomplete spontaneous abortion	Diagnosis	ICD-10-CM
O03.87	Sepsis following complete or unspecified spontaneous abortion	Diagnosis	ICD-10-CM
O04.87	Sepsis following (induced) termination of pregnancy	Diagnosis	ICD-10-CM
O07.37	Sepsis following failed attempted termination of pregnancy	Diagnosis	ICD-10-CM
O08.82	Sepsis following ectopic and molar pregnancy	Diagnosis	ICD-10-CM
O85	Puerperal sepsis	Diagnosis	ICD-10-CM
O86.04	Sepsis following an obstetrical procedure	Diagnosis	ICD-10-CM
P36	Bacterial sepsis of newborn	Diagnosis	ICD-10-CM
P36.0	Sepsis of newborn due to streptococcus, group B	Diagnosis	ICD-10-CM
P36.1	Sepsis of newborn due to other and unspecified streptococci	Diagnosis	ICD-10-CM
P36.10	Sepsis of newborn due to unspecified streptococci	Diagnosis	ICD-10-CM
P36.19	Sepsis of newborn due to other streptococci	Diagnosis	ICD-10-CM
P36.2	Sepsis of newborn due to Staphylococcus aureus	Diagnosis	ICD-10-CM
P36.3	Sepsis of newborn due to other and unspecified staphylococci	Diagnosis	ICD-10-CM
P36.30	Sepsis of newborn due to unspecified staphylococci	Diagnosis	ICD-10-CM
P36.39	Sepsis of newborn due to other staphylococci	Diagnosis	ICD-10-CM
P36.4	Sepsis of newborn due to Escherichia coli	Diagnosis	ICD-10-CM
P36.5	Sepsis of newborn due to anaerobes	Diagnosis	ICD-10-CM
P36.8	Other bacterial sepsis of newborn	Diagnosis	ICD-10-CM
P36.9	Bacterial sepsis of newborn, unspecified	Diagnosis	ICD-10-CM
R65.2	Severe sepsis	Diagnosis	ICD-10-CM
T81.44	Sepsis following a procedure	Diagnosis	ICD-10-CM
T81.44XA	Sepsis following a procedure, initial encounter	Diagnosis	ICD-10-CM
T81.44XD	Sepsis following a procedure, subsequent encounter	Diagnosis	ICD-10-CM
T81.44XS	Sepsis following a procedure, sequela	Diagnosis	ICD-10-CM
**Myocarditis/Pericarditis**			
I30	Acute pericarditis	Diagnosis	ICD-10-CM
I30.0	Acute nonspecific idiopathic pericarditis	Diagnosis	ICD-10-CM
I30.1	Infective pericarditis	Diagnosis	ICD-10-CM
I30.8	Other forms of acute pericarditis	Diagnosis	ICD-10-CM
I30.9	Acute pericarditis, unspecified	Diagnosis	ICD-10-CM
I31	Other diseases of pericardium	Diagnosis	ICD-10-CM

Code	Description	Code Category	Code Type
I31.0	Chronic adhesive pericarditis	Diagnosis	ICD-10-CM
I31.1	Chronic constrictive pericarditis	Diagnosis	ICD-10-CM
I31.2	Hemopericardium, not elsewhere classified	Diagnosis	ICD-10-CM
I31.3	Pericardial effusion (noninflammatory)	Diagnosis	ICD-10-CM
I31.4	Cardiac tamponade	Diagnosis	ICD-10-CM
I31.8	Other specified diseases of pericardium	Diagnosis	ICD-10-CM
I31.9	Disease of pericardium, unspecified	Diagnosis	ICD-10-CM
I32	Pericarditis in diseases classified elsewhere	Diagnosis	ICD-10-CM
I40	Acute myocarditis	Diagnosis	ICD-10-CM
I40.0	Infective myocarditis	Diagnosis	ICD-10-CM
I40.1	Isolated myocarditis	Diagnosis	ICD-10-CM
I40.8	Other acute myocarditis	Diagnosis	ICD-10-CM
I40.9	Acute myocarditis, unspecified	Diagnosis	ICD-10-CM
I41	Myocarditis in diseases classified elsewhere	Diagnosis	ICD-10-CM
**Bilevel Positive Airway Pressure (BiPAP)**			
A7027	Combination oral/nasal mask, used with continuous positive airway pressure device, each	Procedure	HCPCS
A7030	Full face mask used with positive airway pressure device, each	Procedure	HCPCS
A7034	Nasal interface (mask or cannula type) used with positive airway pressure device, with or without head strap	Procedure	HCPCS
A7044	Oral interface used with positive airway pressure device, each	Procedure	HCPCS
A7045	Exhalation port with or without swivel used with accessories for positive airway devices, replacement only	Procedure	HCPCS
A7046	Water chamber for humidifier, used with positive airway pressure device, replacement, each	Procedure	HCPCS
E0470	Respiratory assist device, bi-level pressure capability, without backup rate feature, used with noninvasive interface, e.g., nasal or facial mask (intermittent assist device with continuous positive airway pressure device)	Procedure	HCPCS
E0471	Respiratory assist device, bi-level pressure capability, with back-up rate feature, used with noninvasive interface, e.g., nasal or facial mask (intermittent assist device with continuous positive airway pressure device)	Procedure	HCPCS
E0472	Respiratory assist device, bi-level pressure capability, with backup rate feature, used with invasive interface, e.g., tracheostomy tube (intermittent assist device with continuous positive airway pressure device)	Procedure	HCPCS
E0561	Humidifier, nonheated, used with positive airway pressure device	Procedure	HCPCS
E0562	Humidifier, heated, used with positive airway pressure device	Procedure	HCPCS
**Extracorporeal Membrane Oxygenation (ECMO)**			
39.65	Extracorporeal membrane oxygenation (ECMO)	Procedure	ICD-9-PCS
5A15223	Extracorporeal Membrane Oxygenation, Continuous	Procedure	ICD-10-PCS
5A1522F	Extracorporeal Oxygenation, Membrane, Central	Procedure	ICD-10-PCS
5A1522G	Extracorporeal Oxygenation, Membrane, Peripheral Veno-arterial	Procedure	ICD-10-PCS
5A1522H	Extracorporeal Oxygenation, Membrane, Peripheral Veno-venous	Procedure	ICD-10-PCS
5A15A2F	Extracorporeal Oxygenation, Membrane, Central, Intraoperative	Procedure	ICD-10-PCS
5A15A2G	Extracorporeal Oxygenation, Membrane, Peripheral Veno-arterial, Intraoperative	Procedure	ICD-10-PCS
5A15A2H	Extracorporeal Oxygenation, Membrane, Peripheral Veno-venous, Intraoperative	Procedure	ICD-10-PCS

Code	Description	Code Category	Code Type
1022227	Extracorporeal membrane oxygenation (ecmo)/ extracorporeal life support (ecls) provided by physician	Procedure	CPT-4
33988	Insertion of left heart vent by thoracic incision (eg, sternotomy, thoracotomy) for ECMO/ECLS	Procedure	CPT-4
33947	Extracorporeal membrane oxygenation (ECMO)/extracorporeal life support (ECLS) provided by physician; initiation, veno-arterial	Procedure	CPT-4
33965	Extracorporeal membrane oxygenation (ECMO)/extracorporeal life support (ECLS) provided by physician; removal of peripheral (arterial and/or venous) cannula(e), percutaneous, birth through 5 years of age	Procedure	CPT-4
33955	Extracorporeal membrane oxygenation (ECMO)/extracorporeal life support (ECLS) provided by physician; insertion of central cannula(e) by sternotomy or thoracotomy, birth through 5 years of age	Procedure	CPT-4
33964	Extracorporeal membrane oxygenation (ECMO)/extracorporeal life support (ECLS) provided by physician; reposition central cannula(e) by sternotomy or thoracotomy, 6 years and older (includes fluoroscopic guidance, when performed)	Procedure	CPT-4
33953	Extracorporeal membrane oxygenation (ECMO)/extracorporeal life support (ECLS) provided by physician; insertion of peripheral (arterial and/or venous) cannula(e), open, birth through 5 years of age	Procedure	CPT-4
33959	Extracorporeal membrane oxygenation (ECMO)/extracorporeal life support (ECLS) provided by physician; reposition peripheral (arterial and/or venous) cannula(e), open, birth through 5 years of age (includes fluoroscopic guidance, when performed)	Procedure	CPT-4
33984	Extracorporeal membrane oxygenation (ECMO)/extracorporeal life support (ECLS) provided by physician; removal of peripheral (arterial and/or venous) cannula(e), open, 6 years and older	Procedure	CPT-4
33962	Extracorporeal membrane oxygenation (ECMO)/extracorporeal life support (ECLS) provided by physician; reposition peripheral (arterial and/or venous) cannula(e), open, 6 years and older (includes fluoroscopic guidance, when performed)	Procedure	CPT-4
33958	Extracorporeal membrane oxygenation (ECMO)/extracorporeal life support (ECLS) provided by physician; reposition peripheral (arterial and/or venous) cannula(e), percutaneous, 6 years and older (includes fluoroscopic guidance, when performed)	Procedure	CPT-4
33986	Extracorporeal membrane oxygenation (ECMO)/extracorporeal life support (ECLS) provided by physician; removal of central cannula(e) by sternotomy or thoracotomy, 6 years and older	Procedure	CPT-4
33952	Extracorporeal membrane oxygenation (ECMO)/extracorporeal life support (ECLS) provided by physician; insertion of peripheral (arterial and/or venous) cannula(e), percutaneous, 6 years and older (includes fluoroscopic guidance, when performed)	Procedure	CPT-4
33948	Extracorporeal membrane oxygenation (ECMO)/extracorporeal life support (ECLS) provided by physician; daily management, each day, veno-venous	Procedure	CPT-4
33989	Removal of left heart vent by thoracic incision (eg, sternotomy, thoracotomy) for ECMO/ECLS	Procedure	CPT-4
33946	Extracorporeal membrane oxygenation (ECMO)/extracorporeal life support (ECLS) provided by physician; initiation, veno-venous	Procedure	CPT-4

Code	Description	Code Category	Code Type
36822	Insertion of cannula(s) for prolonged extracorporeal circulation for cardiopulmonary insufficiency (ECMO) (separate procedure)	Procedure	CPT-4
33954	Extracorporeal membrane oxygenation (ECMO)/extracorporeal life support (ECLS) provided by physician; insertion of peripheral (arterial and/or venous) cannula(e), open, 6 years and older	Procedure	CPT-4
33951	Extracorporeal membrane oxygenation (ECMO)/extracorporeal life support (ECLS) provided by physician; insertion of peripheral (arterial and/or venous) cannula(e), percutaneous, birth through 5 years of age (includes fluoroscopic guidance, when performed)	Procedure	CPT-4
33963	Extracorporeal membrane oxygenation (ECMO)/extracorporeal life support (ECLS) provided by physician; reposition of central cannula(e) by sternotomy or thoracotomy, birth through 5 years of age (includes fluoroscopic guidance, when performed)	Procedure	CPT-4
33969	Extracorporeal membrane oxygenation (ECMO)/extracorporeal life support (ECLS) provided by physician; removal of peripheral (arterial and/or venous) cannula(e), open, birth through 5 years of age	Procedure	CPT-4
33966	Extracorporeal membrane oxygenation (ECMO)/extracorporeal life support (ECLS) provided by physician; removal of peripheral (arterial and/or venous) cannula(e), percutaneous, 6 years and older	Procedure	CPT-4
33987	Arterial exposure with creation of graft conduit (eg, chimney graft) to facilitate arterial perfusion for ECMO/ECLS (List separately in addition to code for primary procedure)	Procedure	CPT-4
33957	Extracorporeal membrane oxygenation (ECMO)/extracorporeal life support (ECLS) provided by physician; reposition peripheral (arterial and/or venous) cannula(e), percutaneous, birth through 5 years of age (includes fluoroscopic guidance, when performed)	Procedure	CPT-4
33985	Extracorporeal membrane oxygenation (ECMO)/extracorporeal life support (ECLS) provided by physician; removal of central cannula(e) by sternotomy or thoracotomy, birth through 5 years of age	Procedure	CPT-4
33949	Extracorporeal membrane oxygenation (ECMO)/extracorporeal life support (ECLS) provided by physician; daily management, each day, veno-arterial	Procedure	CPT-4
33956	Extracorporeal membrane oxygenation (ECMO)/extracorporeal life support (ECLS) provided by physician; insertion of central cannula(e) by sternotomy or thoracotomy, 6 years and older	Procedure	CPT-4
33960	Prolonged extracorporeal circulation for cardiopulmonary insufficiency; initial day	Procedure	CPT-4
33961	Prolonged extracorporeal circulation for cardiopulmonary insufficiency; each subsequent day	Procedure	CPT-4
	**Intensive Care Unit (ICU)**		
200	ICU general	Procedure	Revenue
201	ICU surgical	Procedure	Revenue
202	ICU medical	Procedure	Revenue
203	ICU pediatric	Procedure	Revenue
204	ICU psychiatric stay	Procedure	Revenue
206	ICU intermediate	Procedure	Revenue
207	ICU burn care	Procedure	Revenue

Code	Description	Code Category	Code Type
208	ICU trauma	Procedure	Revenue
209	ICU other	Procedure	Revenue
210	CCU	Procedure	Revenue
211	CCU myo infarc	Procedure	Revenue
212	CCU pulmonary	Procedure	Revenue
213	CCU transplant	Procedure	Revenue
214	CCU intermediate	Procedure	Revenue
219	CCU other	Procedure	Revenue
0581F	Patient transferred directly from anesthetizing location to critical care unit (Peri2)	Procedure	CPT-4
0582F	Patient not transferred directly from anesthetizing location to critical care unit (Peri	Procedure	CPT-4
0188T	Remote real-time interactive video-conferenced critical care, evaluation and management of the critically ill or critically injured patient; first 30-74 minutes	Procedure	CPT-4
0189T	Remote real-time interactive video-conferenced critical care, evaluation and management of the critically ill or critically injured patient; each additional 30 minutes (List separately in addition to code for primary service)	Procedure	CPT-4
99171	Critical Care, Subsequent Follow-up Visit; Brief Examination, Evaluation And/or Treatment For Same Illness	Procedure	CPT-4
99160	Critical Care, Initial, Including The Diagnostic And Therapeutic Services And Direction Of Care Of The Critically Ill Or Multiply Injured Or Comatose Patient, Requiring The Prolonged Presence Of The Physician; First Hour	Procedure	CPT-4
99162	Critical Care, Initial, Including The Diagnostic And Therapeutic Services And Direction Of Care Of The Critically Ill Or Multiply Injured Or Comatose Patient, Requiring The Prolonged Presence Of The Physician; Each Additional 30 Minutes	Procedure	CPT-4
99291	Critical care, evaluation and management of the critically ill or critically injured patient; first 30-74 minutes	Procedure	CPT-4
99292	Critical care, evaluation and management of the critically ill or critically injured patient; each additional 30 minutes (List separately in addition to code for primary service)	Procedure	CPT-4
G0390	Trauma response team associated with hospital critical care service	Procedure	HCPCS
G0509	Telehealth consultation, critical care, subsequent, physicians typically spend 50 minutes communicating with the patient and providers via telehealth	Procedure	HCPCS
G0508	Telehealth consultation, critical care, initial, physicians typically spend 60 minutes communicating with the patient and providers via telehealth	Procedure	HCPCS
G0240	Critical care service delivered by a physician, face to face; during interfacility transport of a critically ill or critically injured patient; first 30-74 minutes of active transport	Procedure	HCPCS
**Invasive Mechanical Ventilation**			
Z99.1	Dependence on respirator	Diagnosis	ICD-10-CM
Z99.11	Dependence on respirator [ventilator] status	Diagnosis	ICD-10-CM
09HN7BZ	Insertion of Airway into Nasopharynx, Via Natural or Artificial Opening	Procedure	ICD-10-PCS
09HN8BZ	Insertion of Airway into Nasopharynx, Via Natural or Artificial Opening Endoscopic	Procedure	ICD-10-PCS
0BH13EZ	Insertion of Endotracheal Airway into Trachea, Percutaneous Approach	Procedure	ICD-10-PCS
0BH17EZ	Insertion of Endotracheal Airway into Trachea, Via Natural or Artificial Opening	Procedure	ICD-10-PCS
0BH18EZ	Insertion of Endotracheal Airway into Trachea, Via Natural or Artificial Opening Endoscopic	Procedure	ICD-10-PCS

Code	Description	Code Category	Code Type
0CHY7BZ	Insertion of Airway into Mouth and Throat, Via Natural or Artificial Opening	Procedure	ICD-10-PCS
0CHY8BZ	Insertion of Airway into Mouth and Throat, Via Natural or Artificial Opening Endoscopic	Procedure	ICD-10-PCS
0DH57BZ	Insertion of Airway into Esophagus, Via Natural or Artificial Opening	Procedure	ICD-10-PCS
0DH58BZ	Insertion of Airway into Esophagus, Via Natural or Artificial Opening Endoscopic	Procedure	ICD-10-PCS
0WHQ73Z	Insertion of Infusion Device into Respiratory Tract, Via Natural or Artificial Opening	Procedure	ICD-10-PCS
0WHQ7YZ	Insertion of Other Device into Respiratory Tract, Via Natural or Artificial Opening	Procedure	ICD-10-PCS
5A0920Z	Assistance with Respiratory Filtration, Continuous	Procedure	ICD-10-PCS
5A09357	Assistance with Respiratory Ventilation, Less than 24 Consecutive Hours, Continuous Positive Airway Pressure	Procedure	ICD-10-PCS
5A09358	Assistance with Respiratory Ventilation, Less than 24 Consecutive Hours, Intermittent Positive Airway Pressure	Procedure	ICD-10-PCS
5A09359	Assistance with Respiratory Ventilation, Less than 24 Consecutive Hours, Continuous Negative Airway Pressure	Procedure	ICD-10-PCS
5A0935A	Assistance with Respiratory Ventilation, Less than 24 Consecutive Hours, High Nasal Flow/Velocity	Procedure	ICD-10-PCS
5A0935B	Assistance with Respiratory Ventilation, Less than 24 Consecutive Hours, Intermittent Negative Airway Pressure	Procedure	ICD-10-PCS
5A0935Z	Assistance with Respiratory Ventilation, Less than 24 Consecutive Hours	Procedure	ICD-10-PCS
5A09457	Assistance with Respiratory Ventilation, 24-96 Consecutive Hours, Continuous Positive Airway Pressure	Procedure	ICD-10-PCS
5A09458	Assistance with Respiratory Ventilation, 24-96 Consecutive Hours, Intermittent Positive Airway Pressure	Procedure	ICD-10-PCS
5A09459	Assistance with Respiratory Ventilation, 24-96 Consecutive Hours, Continuous Negative Airway Pressure	Procedure	ICD-10-PCS
5A0945A	Assistance with Respiratory Ventilation, 24-96 Consecutive Hours, High Nasal Flow/Velocity	Procedure	ICD-10-PCS
5A0945B	Assistance with Respiratory Ventilation, 24-96 Consecutive Hours, Intermittent Negative Airway Pressure	Procedure	ICD-10-PCS
5A0945Z	Assistance with Respiratory Ventilation, 24-96 Consecutive Hours	Procedure	ICD-10-PCS
5A09557	Assistance with Respiratory Ventilation, Greater than 96 Consecutive Hours, Continuous Positive Airway Pressure	Procedure	ICD-10-PCS
5A09558	Assistance with Respiratory Ventilation, Greater than 96 Consecutive Hours, Intermittent Positive Airway Pressure	Procedure	ICD-10-PCS
5A09559	Assistance with Respiratory Ventilation, Greater than 96 Consecutive Hours, Continuous Negative Airway Pressure	Procedure	ICD-10-PCS
5A0955A	Assistance with Respiratory Ventilation, Greater than 96 Consecutive Hours, High Nasal Flow/Velocity	Procedure	ICD-10-PCS
5A0955B	Assistance with Respiratory Ventilation, Greater than 96 Consecutive Hours, Intermittent Negative Airway Pressure	Procedure	ICD-10-PCS
5A0955Z	Assistance with Respiratory Ventilation, Greater than 96 Consecutive Hours	Procedure	ICD-10-PCS
31500	Intubation, endotracheal, emergency procedure	Procedure	CPT-4
94003	Ventilation assist and management, initiation of pressure or volume preset ventilators for assisted or controlled breathing; hospital inpatient/observation, each subsequent day	Procedure	CPT-4

Code	Description	Code Category	Code Type
94002	Ventilation assist and management, initiation of pressure or volume preset ventilators for assisted or controlled breathing; hospital inpatient/observation, initial day	Procedure	CPT-4
A0396	ALS specialized service disposable supplies; esophageal intubation	Procedure	HCPCS
E0481	Intrapulmonary percussive ventilation system and related accessories	Procedure	HCPCS
G8569	Prolonged postoperative intubation (> 24 hrs) required	Procedure	HCPCS
E0472	Respiratory assist device, bi-level pressure capability, with backup rate feature, used with invasive interface, e.g., tracheostomy tube (intermittent assist device with continuous positive airway pressure device)	Procedure	HCPCS
A4483	Moisture exchanger, disposable, for use with invasive mechanical ventilation	Procedure	HCPCS
**Potential COVID-19 Treatments**			
XW033E5	Introduction of Remdesivir Anti-infective into Peripheral Vein, Percutaneous Approach, New Technology Group 5	Procedure	ICD-10-PCS
XW043E5	Introduction of Remdesivir Anti-infective into Central Vein, Percutaneous Approach, New Technology Group 5	Procedure	ICD-10-PCS
C9264	Injection, tocilizumab, 1 mg	Procedure	HCPCS
J3262	Injection, tocilizumab, 1 mg	Procedure	HCPCS
C9236	Injection, eculizumab, 10 mg	Procedure	HCPCS
J1300	Injection, eculizumab, 10 mg	Procedure	HCPCS
J1020	Injection, methylprednisolone acetate, 20 mg	Procedure	HCPCS
J1030	Injection, methylprednisolone acetate, 40 mg	Procedure	HCPCS
J1040	Injection, methylprednisolone acetate, 80 mg	Procedure	HCPCS
J2920	Injection, methylprednisolone sodium succinate, up to 40 mg	Procedure	HCPCS
J2930	Injection, methylprednisolone sodium succinate, up to 125 mg	Procedure	HCPCS
J7509	Methylprednisolone, oral, per 4 mg	Procedure	HCPCS
J1094	Injection, dexamethasone acetate, 1 mg	Procedure	HCPCS
J1100	Injection, dexamethasone sodium phosphate, 1 mg	Procedure	HCPCS
J8540	Dexamethasone, oral, 0.25 mg	Procedure	HCPCS
S0173	Dexamethasone, oral, 4 mg	Procedure	HCPCS
XW0DXM6	Introduction of baricitinib into mouth and pharynx, external approach, new technology group 6	Procedure	ICD-10-PCS
XW0G7M6	Introduction of baricitinib into upper GI, via natural or artificial opening, new technology group 6	Procedure	ICD-10-PCS
XW0H7M6	Introduction of baricitinib into lower GI, via natural or artificial opening, new technology group 6	Procedure	ICD-10-PCS
Q0239	Injection, bamlanivimab-xxxx, 700 mg	Procedure	HCPCS
M0239	intravenous infusion, bamlanivimab-xxxx, includes infusion and post administration monitoring	Procedure	HCPCS
XW033F6	Introduction of Bamlanivimab Monoclonal Antibody into Peripheral Vein, Percutaneous Approach, New Technology Group 6	Procedure	ICD-10-PCS
XW043F6	Introduction of Bamlanivimab Monoclonal Antibody into Central Vein, Percutaneous Approach, New Technology Group 6	Procedure	ICD-10-PCS
Q0245	Injection, bamlanivimab and etesevimab, 2100 mg	Procedure	HCPCS
M0245	Intravenous infusion, bamlanivimab and etesevimab, includes infusion and post administration monitoring	Procedure	HCPCS

Code	Description	Code Category	Code Type
XW043E6	Introduction of Etesevimab Monoclonal Antibody into Central Vein, Percutaneous Approach, New Technology Group 6	Procedure	ICD-10-PCS
XW033E6	Introduction of Etesevimab Monoclonal Antibody into Peripheral Vein, Percutaneous Approach, New Technology Group 6	Procedure	ICD-10-PCS
Q0243	Injection, casirivimab and imdevimab, 2400 mg	Procedure	HCPCS
M0243	intravenous infusion, casirivimab and imdevimab includes infusion and post administration monitoring	Procedure	HCPCS
XW033G6	Introduction of REGN-COV2 monoclonal antibody into peripheral vein, percutaneous approach, new technology group 6	Procedure	ICD-10-PCS
XW043G6	Introduction of REGN-COV2 monoclonal antibody into central vein, percutaneous approach, new technology group 6	Procedure	ICD-10-PCS
J0456	Injection, azithromycin, 500 mg	Procedure	HCPCS
Q0144	Azithromycin dihydrate, oral, capsules/powder, 1 g	Procedure	HCPCS
