# Supplementary material for: Treatment and care received by children hospitalized with COVID-19 in a large hospital network in the United States, February 2020 to September 2021
Source: PLoS One. 2023 Jul 11;18(7):e0288284. doi: 10.1371/journal.pone.0288284 (PMC10335660; doi:10.1371/journal.pone.0288284)
Supplement: S3 Appendix — (PDF) [file pone.0288284.s003.pdf]

S3 Appendix. Generic and Brand Names of Medical Products Used in this Analysis

Generic Name	Brand Name
**Potential COVID-19 Treatments**	
remdesivir	remdesivir
tocilizumab	Actemra
tocilizumab	Actemra ACTPen
sarilumab	Kevzara
eculizumab	Soliris
methylprednisolone acetate	Depo-Medrol
methylprednisolone acetate	methylprednisolone acetate
methylprednisolone acetate	P-Care D40
methylprednisolone acetate	ReadySharp Methylprednisolone
methylprednisolone acetate	methylprednisolone acetate
methylprednisolone acetate in sodium chloride,iso-osmotic/PF	methylpred ac(PF)-NaCl,iso-osm
methylprednisolone acetate in sterile water for injection	methylprednisolone acet-water
methylprednisolone acetate/bupivacaine HCl in sterile water	methylprednisol ac-bupivac-wat
methylprednisolone sodium succinate	Solu-Medrol
methylprednisolone sodium succinate	methylprednisolone sodium succ
methylprednisolone sodium succinate/PF	Solu-Medrol (PF)
lopinavir/ritonavir	Kaletra
lopinavir/ritonavir	lopinavir-ritonavir
norepinephrine bitartrate	Levophed (bitartrate)
norepinephrine bitartrate	norepinephrine bitart (bulk)
norepinephrine bitartrate	norepinephrine bitartrate
norepinephrine bitartrate in 0.9 % sodium chloride	norepinephrine bitartrate-NaCl
norepinephrine bitartrate in 5 % dextrose in water	norepinephrine bitartrate-D5W
dexamethasone	Decadron
dexamethasone	Dexabliss
dexamethasone	dexamethasone
dexamethasone	Dexamethasone Intensol
dexamethasone	DexPak 10 day
dexamethasone	DexPak 13 Day
dexamethasone	DexPak 6 Day
dexamethasone	Dxevo
dexamethasone	HiDex
dexamethasone	TaperDex
dexamethasone acetate	dexamethasone acetate (bulk)
dexamethasone acetate, micronized	dexamethasone ac, micro (bulk)
dexamethasone isonicotinate	dexamethasone isonic (bulk)
dexamethasone sodium phosphate	dexamethasone sod phos (bulk)
dexamethasone sodium phosphate in 0.9 % sodium chloride	dexamethasone-0.9 % sod. chlor
dexamethasone, micronized	dexamethasone,micronized(bulk)
dexamethasone/PF/norflurane/pentafluoropropane (HFC 245fa)	DMT SUIK

Generic Name	Brand Name
baricitinib	Olumiant
Bamlanivimab	Bamlanivimab
Etesevimab	etesevimab
casirivimab	casirivimab (REGN10933)
imdevimab	imdevimab (REGN10987)
casirivimab (REGN10933)/imdevimab (REGN10987)	casirivimab-imdevimab
casirivimab (REGN10933)/imdevimab (REGN10987)	REGEN-COV (EUA)
casirivimab (REGN10933)/imdevimab (REGN10987)	casirivimab-imdevimab
**Antibiotics**	
azithromycin	Azasite
azithromycin	azithromycin
azithromycin	azithromycin (bulk)
azithromycin	Zithromax
azithromycin	Zithromax TRI-PAK
azithromycin	Zithromax Z-Pak
azithromycin/chondroitin sulfate A sodium/PF	Klarity-A (azithro-chondr)(PF)
doxycycline calcium	Vibramycin
doxycycline hyclate	Acticlate
doxycycline hyclate	Doryx
doxycycline hyclate	Doryx MPC
doxycycline hyclate	Doxy-100
doxycycline hyclate	doxycycline hyclate
doxycycline hyclate	doxycycline hyclate (bulk)
doxycycline hyclate	Morgidox
doxycycline hyclate	Soloxide
doxycycline hyclate	Targadox
doxycycline hyclate	Vibramycin
doxycycline monohydrate	Avidoxy
doxycycline monohydrate	doxycycline monohydrate
doxycycline monohydrate	doxycycline monohydrate (bulk)
doxycycline monohydrate	Mondoxyne NL
doxycycline monohydrate	Monodox
doxycycline monohydrate	Okebo
doxycycline monohydrate	Oracea
doxycycline monohydrate	Vibramycin
**Antithrombotic Medications**	
dalteparin sodium,porcine	Fragmin
enoxaparin sodium	enoxaparin
alteplase	Activase
alteplase	Cathflo Activase
reteplase	Retavase
tenecteplase	TNKase
warfarin sodium	Coumadin
warfarin sodium	Jantoven

Generic Name	Brand Name
warfarin sodium	warfarin
warfarin sodium	warfarin (bulk)
apixaban	Eliquis
apixaban	Eliquis DVT-PE Treat 30D Start
betrixaban maleate	Bevyxxa
edoxaban tosylate	Savaysa
fondaparinux sodium	Arixtra
fondaparinux sodium	fondaparinux
rivaroxaban	Xarelto
bivalirudin	bivalirudin
bivalirudin in 0.9 % sodium chloride	bivalirudin-0.9 % sodium chlor
dabigatran etexilate mesylate	Pradaxa
abciximab	Reopro
anagrelide HCl	Agrylin
anagrelide HCl	anagrelide
aspirin	Adult Aspirin Regimen
aspirin	Adult Low Dose Aspirin
aspirin	Aspir-81
aspirin	aspirin
aspirin	aspirin (bulk)
aspirin	Aspirin Childrens
aspirin	Aspirin Low Dose
aspirin	Aspir-Low
aspirin	Aspir-Trin
aspirin	Bayer Advanced
aspirin	Bayer Aspirin
aspirin	Bayer Chewable Aspirin
aspirin	Children's Aspirin
aspirin	Durlaza
aspirin	E.C. Prin
aspirin	Ecotrin
aspirin	Ecotrin Low Strength
aspirin	Enteric Coated Aspirin
aspirin	Extra Strength Bayer
aspirin	Lite Coat Aspirin
aspirin	Lo-Dose Aspirin
aspirin	St Joseph Aspirin
aspirin	St. Joseph Aspirin
aspirin/calcium carbonate	Women's Aspirin with Calcium
aspirin/dipyridamole	Aggrenox
aspirin/dipyridamole	aspirin-dipyridamole
aspirin/omeprazole	aspirin-omeprazole
aspirin/omeprazole	Yosprala
cangrelor tetrasodium	Kengreal

Generic Name	Brand Name
cilostazol	cilostazol
clopidogrel bisulfate	clopidogrel
clopidogrel bisulfate	clopidogrel bisulfate (bulk)
clopidogrel bisulfate	Plavix
dipyridamole	dipyridamole
dipyridamole	dipyridamole (bulk)
eptifibatide	eptifibatide
eptifibatide	Integrilin
prasugrel HCl	Effient
prasugrel HCl	prasugrel
ticagrelor	Brilinta
tirofiban HCl monohydrate	Aggrastat Concentrate
tirofiban HCl monohydrate in 0.9 % sodium chloride	Aggrastat in sodium chloride
vorapaxar sulfate	Zontivity
**Immune globulin (IVIg)**	
immune globulin,gamm(IgG)/glycine/glucose/IgA 0 to 50 mcg/mL	Gammagard S-D (IgA < 1 mcg/mL)
immune globulin,gamm(IgG)/glycine/IgA greater than 50 mcg/mL	Gammagard Liquid
immune globulin,gamm(IgG)/glycine/IgA greater than 50 mcg/mL	HyQvia IG Component
immune globulin,gamm(IgG)/glycine/IgA greater than 50 mcg/mL	Cuvitru
immune globulin,gamm(IgG)/glycine/IgA greater than 50 mcg/mL	Bivigam
immune globulin,gamm(IgG)/maltose/IgA greater than 50 mcg/mL	Octagam
immune globulin,gamm(IgG)/sorbitol/glycin/IgA 0 to 50 mcg/mL	Gammaplex (with sorbitol)
immune globulin,gamm(IgG)/sucrose/IgA greater than 50 mcg/mL	Carimune NF Nanofiltered
immune globulin,gamma (IgG)/glycine/IgA 0 to 50 mcg/mL	Gammaplex
immune globulin,gamma (IgG)/proline/IgA 0 to 50 mcg/mL	Privigen
immune globulin,gamma (IgG)/proline/IgA 0 to 50 mcg/mL	Hizentra
immune globulin,gamma (IgG)/sorbitol/IgA 0 to 50 mcg/mL	Flebogamma DIF
immune globulin,gamma (IgG)-klhw human	Xembify
immune globulin,gamma (IgG)-slra human	Asceniv
immune globulin,gamma(IgG) human/hyaluronidase, human recom	HyQvia
immune globulin,gamma(IgG)/glycine	Gamastan
immune globulin,gamma(IgG)/glycine	GamaSTAN S/D
immune globulin,gamma(IgG)/glycine/IgA average 46 mcg/mL	Gamunex-C
immune globulin,gamma(IgG)/glycine/IgA average 46 mcg/mL	Gammaked
immune globulin,gamma(IgG)-hipp human/maltose	Cutaquig
immune globulin,gamma(IgG)-ifas human/glycine	Panzyga
